# Competing endogenous RNA network mediated by circ_3205 in SARS-CoV-2 infected cells

**DOI:** 10.1007/s00018-021-04119-8

**Published:** 2022-01-17

**Authors:** Davide Barbagallo, Concetta Ilenia Palermo, Cristina Barbagallo, Rosalia Battaglia, Angela Caponnetto, Vittoria Spina, Marco Ragusa, Cinzia Di Pietro, Guido Scalia, Michele Purrello

**Affiliations:** 1 Department of Biomedical and Biotechnological Sciences, Section of Biology and Genetics Giovanni Sichel, University of Catania, 95123 Catania, Italy; 2 U.O.C. Laboratory Analysis Unit, A.O.U. Policlinico‑Vittorio Emanuele, 95123 Catania, Italy; 3 Department of Biomedical and Biotechnological Sciences, Section of Medical Biochemistry, University of Catania, 95123 Catania, Italy; 4 Department of Biomedical and Biotechnological Sciences, Section of Microbiology, University of Catania, 95123 Catania, Italy

**Keywords:** Coronavirus disease 2019, circRNA, miRNA sponge, microRNA, Gene expression regulatory network

## Abstract

Severe Acute Respiratory Syndrome Coronavirus 2 (SARS-CoV-2) is a new member of the *Betacoronaviridae* family, responsible for the recent pandemic outbreak of COVID-19. To start exploring the molecular events that follow host cell infection, we queried VirusCircBase and identified a circular RNA (circRNA) predicted to be synthesized by SARS-CoV-2, circ_3205, which we used to probe: (i) a training cohort comprised of two pools of cells from three nasopharyngeal swabs of SARS-CoV-2 infected (positive) or uninfected (negative, UCs) individuals; (ii) a validation cohort made up of 12 positive and 3 negative samples. The expression of circRNAs, miRNAs and miRNA targets was assayed through real-time PCR. CircRNA–miRNA interactions were predicted by TarpMiR, Analysis of Common Targets for circular RNAs (ACT), and STarMir tools. Enrichment of the biological processes and the list of predicted miRNA targets were retrieved from DIANA miRPath v3.0. Our results showed that the predicted SARS-CoV-2 circ_3205 was expressed only in positive samples and its amount positively correlated with that of SARS-CoV-2 Spike (S) mRNA and the viral load (*r* values = 0.80952 and 0.84867, Spearman’s correlation test, respectively). Human (hsa) miR-298 was predicted to interact with circ_3205 by all three predictive tools. KCNMB4 and PRKCE were predicted as hsa-miR-298 targets. Interestingly, the function of both is correlated with blood coagulation and immune response. *KCNMB4* and *PRKCE* mRNAs were upregulated in positive samples as compared to UCs (6 and 8.1-fold, *p* values = 0.049 and 0.02, Student’s *t* test, respectively) and their expression positively correlated with that of circ_3205 (*r* values = 0.6 and 0.25, Spearman’s correlation test, respectively). We propose that our results convincingly suggest that circ_3205 is a circRNA synthesized by SARS-CoV-2 upon host cell infection and that it may behave as a competitive endogenous RNA (ceRNA), sponging hsa-miR-298 and contributing to the upregulation of *KCNMB4* and *PRKCE* mRNAs.

**Supplementary Information:**

The online version contains supplementary material available at 10.1007/s00018-021-04119-8.

## Introduction

Severe Acute Respiratory Syndrome Coronavirus 2 (SARS-CoV-2) is an enveloped virus classified as a new member of the *Coronaviridae* family, *Betacoronavirus* genus, whose genome consists of a single stranded positive ( +) RNA molecule about 30 kilobases long [1, 2]. SARS-CoV-2 is etiologically involved in the life-threatening coronavirus disease 2019 (COVID-19) and is responsible for a pandemic outbreak in March, 2020 [3]. From December 2019 to September 2021, 3,508 SARS-CoV-2 genomes were sampled and 20 clades were identified around the world, according to the GISAID database (https://nextstrain.org/ncov/gisaid/global). Emergence of new SARS-CoV-2 genotypes alerts the scientific community to the possibility that some variants of concern may bypass the immune barrier given by the vaccines currently used and highlights the importance of the identification of therapeutic targets helpful for the management of this clinically very relevant disease [4].

Circular RNAs (circRNAs) are a recently discovered class of RNAs, mainly synthesized through backsplicing and characterized by the covalent bond between their 5’ and 3’ termini [5, 6]. CircRNAs follow tissue- and developmental-specific expression patterns and are mainly localized in the cell cytoplasm [7, 8]. The best characterized functions of circRNAs consist in sponging microRNAs (miRNAs) and RNA-binding proteins (RBPs) [5, 9]: in the first case, circRNAs may be typically involved in competitive endogenous RNA (ceRNA) networks [10–13]; in the second case, circRNAs may regulate biological processes within eukaryotic cells, such as assembly of preinitiation complex (PIC) at the beginning of transcription or also splicing [14, 15]. Moreover, circRNAs may function either as a template for the synthesis of generally short peptides, thanks to the presence of Internal Ribosomes Entry Sites (IRESs) within their sequences, or as a scaffold for the regulation of host gene transcription [16–18]. CircRNAs have been found aberrantly expressed in many cancers and degenerative diseases [19, 20] and are associated with several biological processes, both in physiological and pathological conditions [21–24]. Due to their intrinsic resistance to the activity of exoribonucleases and their presence in several human body fluids as well as within extracellular vesicles, circRNAs have been suggested as good candidate diagnostic and prognostic biomarkers for several diseases [25–28].

Recent evidence has shown the etiological involvement of circRNAs in viral infections. Most specifically, cross-talk between host cell circRNA biogenesis and RBPs linked to immune response (e.g.: immune factors NF90/NF110) has been described [29]. Influenza virus-infected A549 cells showed the induction of a circRNA that acts as a sponge for miRNAs regulating the expression of interferon beta (IFN-β) enhanceosome [30]. At the same time, circRNAs have been demonstrated to be synthesized from the genome of several DNA viruses (e.g.: Herpesviruses), contributing to the infection’s pathogenesis [31–33].

Recently, thanks to RNA-seq data analysis from cells infected with Middle East Respiratory Syndrome Coronavirus (MERS-CoV), Severe Acute Respiratory Syndrome Coronavirus (SARS-CoV) and SARS-CoV-2 RNA (+) betacoronaviruses, several circRNAs of viral origin have been identified and characterized [34, 35]. At the same time, analysis of human circRNAome revealed several differentially expressed (DE) circRNAs in lung epithelial cells as well as in the peripheral blood of individuals infected with SARS-CoV-2 [36, 37]. Based on gene expression datasets, perturbation of ceRNA networks (circRNAs-miRNAs-mRNAs) in host cells following SARS-CoV-2 infection has also been predicted [38]. To improve our knowledge of the molecular dynamics of SARS-CoV-2 infection and prospectively identify new candidate therapeutic targets, in this study we focused on circRNAs synthesized from the viral genome, suggesting their involvement in COVID-19 pathogenesis.

## Materials and methods

### Sample preparation and diagnosis

Three ml of universal transport medium (UTM™) (COPAN Italia SpA, Brescia, Italy) from nasopharyngeal swabs of individuals suspected to be infected by SARS-CoV-2 were used for diagnostic purposes. A residual 1 ml of UTM™ was centrifuged at 350×*g* for 5 min at 4 °C to pellet cell debris. Supernatants were discarded and cell pellets stored at − 80 °C until further processing. Nucleic acids were isolated directly from UTM™ through a ThermoFisher Flex apparatus by the MagMAXTM Viral Pathogen Kit (ThermoFisher Scientific, Monza, Italy) for diagnostic purposes. Diagnosis was performed by a multiplex real-time PCR, Allplex™ SARS-CoV-2 Master assay (Seegene Inc., Arrow Diagnostics, Genoa, Italy), following the instructions of the manufacturer. The method amplifies SARS-CoV-2 E, N, RdRp, and S genes, according to World Health Organization (WHO)’s guidelines. Amplification cycles were performed on a Bio-Rad CFX96 real-time PCR instrument (Bio-Rad, Segrate–Milan, Italy). Only samples showing cycle threshold (*Ct*) values ≤ 35 for all the transcripts assayed were considered positive. A total of 15 positive and 6 and negative samples were assayed in this retrospective study. Data on biological specimens anonymously collected in this study were processed in accordance with the ethical principles reported in the Declaration of Helsinki.

### RNA extraction, PCR amplification and Sanger sequencing

RNA was extracted from cells previously collected by nasopharyngeal swab using TRIzol^®^ (ThermoFisher Scientific, ThermoFisher Scientific, Waltham, MA, USA), according to the manufacturer’s instruction [39]. RNA was quantified by a GenQuant pro spectrophotometer (Biochrom, Cambridge, UK). CircRNAs and mRNAs were amplified using a Power SYBR^®^ Green RNA-to-CT™ 1-Step Kit (ThermoFisher Scientific). MiRNAs were reverse transcribed into cDNA by TaqMan™ MicroRNA Reverse Transcription Kit (ThermoFisher Scientific) and amplified through TaqMan™ Universal Master Mix II (ThermoFisher Scientific). PCRs were run on a 7900HT real-time PCR instrument (ThermoFisher Scientific). PCR products of the amplified circ_3205 were then sent to BMR Genomics, Padua, Italy (www.bmr-genomics.it) for purification through ExoSap (Applied Biosystems™, ThermoFischer Scientific). Sanger sequencing was performed with BigDyeTM Terminator v3.1 Cycle Sequencing Kit (ThermoFisher Scientific) on a ABI 3730xl DNA Analyzer (Applied Biosystems™, ThermoFisher Scientific). Sequences and IDs of specific primer pairs and TaqMan probes used in this manuscript are listed in Supplemental Table 1.

### Candidate circRNAs selection

SARS-CoV-2 candidate circRNA sequences were retrieved from VirusCircBase (v. 1.1) [40], based on RNA-Seq experiments performed on Calu3 cells infected with the virus for 12 h and 24 h (GSE148729). Candidate circRNAs were filtered through the following criteria: (i) backsplice junction reads counted by at least two circRNA predictive algorithms among CIRI2; circRNA_finder; find_circ [6, 41, 42], for the same circRNA; (ii) abundancy of circRNA (at least two backsplice junction reads for each predictive algorithm).

### Prediction of circRNA/miRNA interactions

Interactions between candidate circRNA and miRNAs were predicted by TarpMiR [43], Analysis of Common Targets for circular RNAs (ACT) [44], and STarMir [45]. FASTA sequences of human miRNAs (from miRBase 22 release [46]) and candidate circRNA were given as input to each of the three predictive algorithms. TarpMiR was set choosing human model and a probability cutoff of 0.5. Human V-CLIP data were used to train STarMir predictions [47]. Only miRNAs predicted to interact with SARS-CoV-2 candidate circRNA by all the three tools were considered as potentially implied in the ceRNA network.

### Gene Ontology (GO) analysis and miRNA target selection

MiRNAs predicted to interact with candidate circRNA were given as input to DIANA miRPath v3.0 [48] and Biological Process (BP) GO’s subcategory was analyzed. MicroT-CDS (MicroT and False Discovery Rate (FDR)-corrected *p* value thresholds = 0.8 and 0.05, respectively) was selected as the algorithm for the prediction of miRNA-mRNA interactions. BP GO analysis filtered miRNA targets based on: (i) their potential involvement in blood clotting and immune response pathways, known to be related to COVID-19; (ii) dysregulated expression in lung or nasopharyngeal cells from SARS-CoV-2 infected individuals.

### Protein–protein interaction (PPI) network analysis

First and second neighbor interactants of the candidate miRNA targets were retrieved from the HUman Reference protein Interactome (HuRI) database [49]. The list of interactions was given as input to Cytoscape (version 3.8.2) [50] and the network generated was analyzed through g:GOSt, within the g:Profiler web interface [51], and the Cytoscape plugin cytoHubba [52] to study gene functional enrichment and topological features, respectively. Topological analysis focused on the centrality parameters: betweenness; bottleneck; closeness; eccentricity; radiality and stress; for each of them, a corresponding subnetwork has been generated and analyzed for GO BP enrichment.

### Statistical analysis

Real-time PCR data were analyzed through the 2^−DD*Ct*^ method [53] and two-sided Student’s *t* test was applied to identify DE transcripts. Spearman’s correlation test was used to identify positive or negative correlations among transcripts. Modified Fisher’s exact test followed by false discovery rate methodology was used to calculate *p* values for GO analysis. *p* values ≤ 0.05 were considered statistically significant.

## Results

### circRNA_3205 is a candidate viral circRNA expressed in Calu3 infected with SARS-CoV-2

Based on VirusCircBase, a total of 3473 circRNAs were detected by at least one tool among CIRI2; circRNA_finder; find_circ (Supplemental Table 2). SARS-CoV-2 circRNAs 363, 368, 2667, 2670, 2685, 2795, 3058, 3205 were the top eight most expressed circRNAs in Calu-3 infected with SARS-CoV-2 at least in one time point, according at least to two out of three algorithms among CIRI2; circRNA_finder; find_circ (Table 1).

**Table 1:** Candidate SARS-CoV-2 circRNAs in Calu-3 infected with SARS-CoV-2 for 12 h and 24 h (data from GSE148729)

SARS-CoV-2 CircRNA ID (VirusCircBase)	Start	End	Strand	SARS-CoV-2 host gene	Mean JRC 12 h circRNA_finder	Mean JRC 12 h find_circ	Mean JRC 12 h CIRI2	Mean JRC 24 h circfinder	Mean JRC 24 h find_circ	Mean JRC 24 h CIRI2
circ_363	2571	3263	−	ORF1ab	N/A	2	9.5	N/A	4.5	30
circ_368	2571	3539	−	ORF1ab	N/A	2	31	N/A	2.5	76
circ_2667	25,672	26,212	−	ORF3a	N/A	2.5	26.5	N/A	3.5	33
circ_2670	25,672	26,241	−	ORF3a	N/A	10.5	25	N/A	10	104
circ_2685	25,672	26,570	−	ORF3a;M;E	N/A	3	26.5	N/A	12.5	60
circ_2795	26,825	27,225	+	ORF6;M	12	15	N/A	12.5	17.5	N/A
circ_3058	28,404	29,080	+	N	4.5	5.5	N/A	19	20.5	N/A
circ_3205	28,610	28,895	−	N	N/A	12	17	N/A	7	15.5

For each circRNA, the arithmetic mean of the junction read counts (JCR) calculated from each tool for two biological replicates, is reported

*N/A*  JCR not annotated by the tool

### Circ_3205 is expressed only in positive samples and its amount positively correlates with that of viral Spike (S) mRNA

**Fig. 1:**
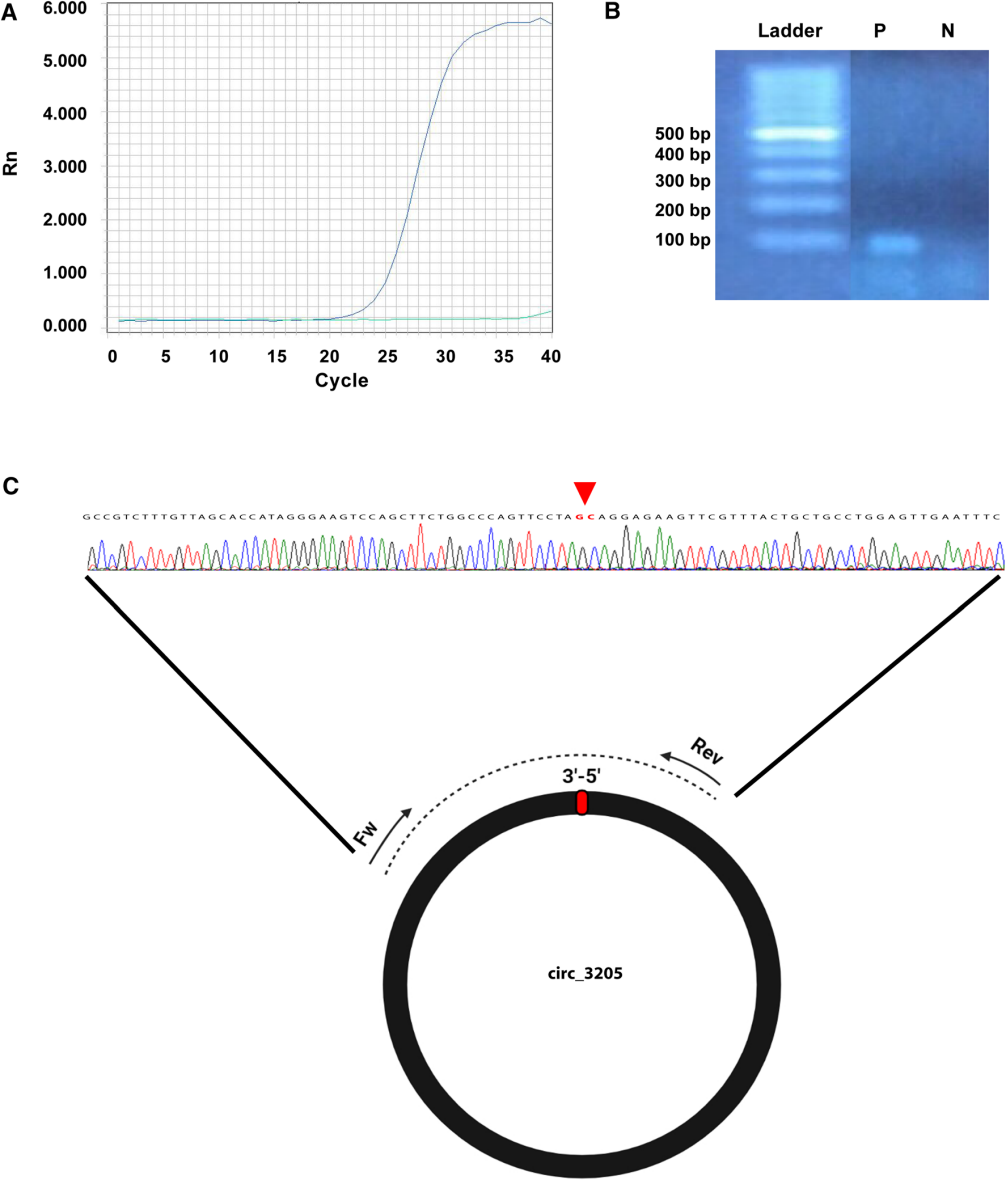
**A** Real-time PCR amplification plot of circ_3205 for a representative positive (blue curve) and negative (green curve) sample. **B** Agarose gel (2%) electrophoresis of the real-time PCR products in (A). Ladder = 100 bp DNA ladder (ThermoFisher Scientific); P and N = representative positive and negative sample, respectively. **C** Graphical representation of the divergent primers used to amplify SARS-CoV-2 circ_3205 and Sanger sequencing of the resulting PCR amplicon (from a representative positive sample). Dotted line represents the PCR amplicon obtained through the use of divergent primers; the red triangle above the electropherogram highlights the 3ʹ–5ʹ junction of circ_3205

**Fig. 2:**
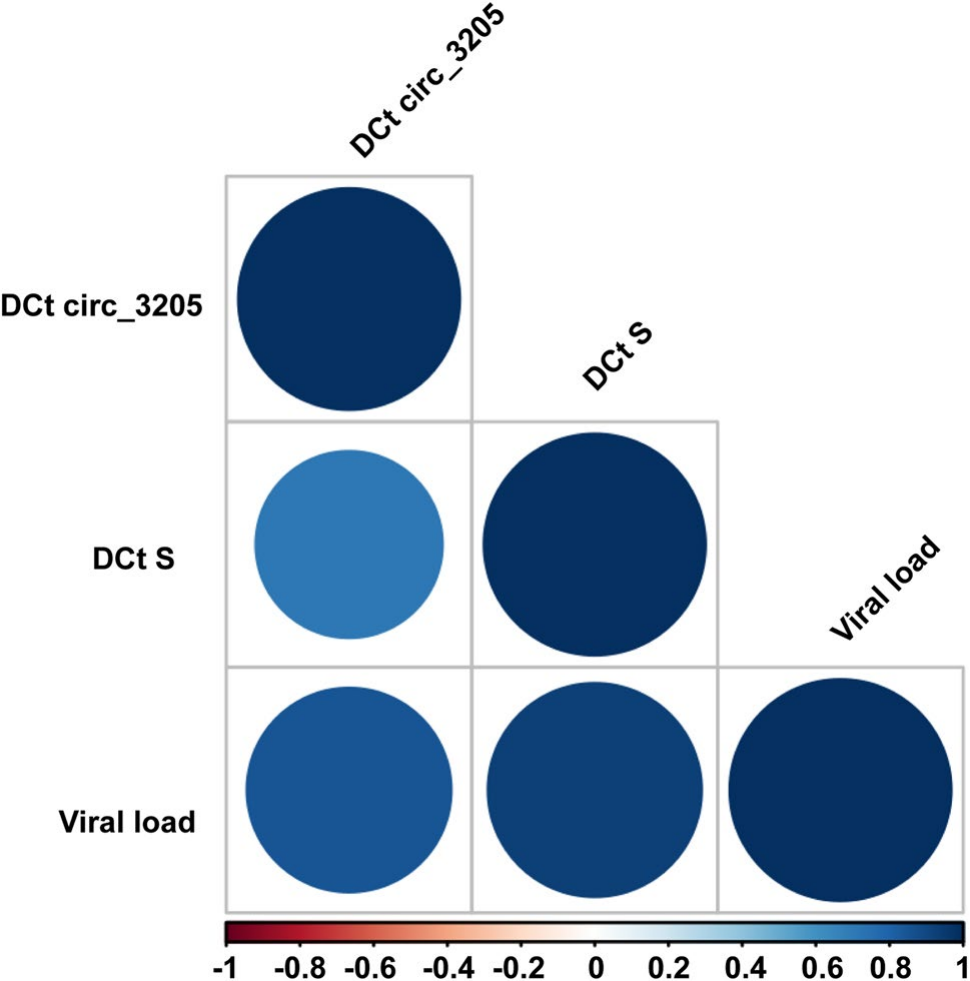
Correlogram showing correlations among the expression of circ_3205, S mRNA and the viral load. Expression of circ_3205 and S mRNA is reported as DCt (*Ct* of the transcript of interest – *Ct*  of *GAPDH*, used as endogenous control). Viral load was estimated on the basis of the mean of the *Cts* of SARS-CoV-2 E, N, RdRp, and S genes. The color of the circle is related to the correlation coefficient (*r* value), estimated through the Spearman correlation test: the deeper is the blue, the more positive is the correlation, the deeper is the red, the more negative is the correlation, as reported in the legend of the figure. The size of the circle is proportional to the significance of the correlation: the higher is the size of the circle, the lower is the *p* value

The expression of SARS-CoV-2 circRNAs 363, 368, 2667, 2670, 2685, 2795, 3058, and 3205 was assayed in a discovery cohort made of a pool of three positive samples and three UCs. Based on real-time PCR and gel electrophoresis data, we focused on circ_3205, a 286 nt-long circRNA whose sequence is embedded within the open reading frame (ORF) coding for the nucleocapside (N) protein of SARS-CoV-2 (nucleotide position 28,609–28,898, Wuhan-Hu-1 reference genome, NCBI Reference Sequence: NC_045512.2), clearly present and highly expressed only in positive samples (Fig. 1A and 1B). Sanger sequencing of the PCR amplification product of circ_3205 confirmed the presence of the 3’-5’ junction, specific of this circRNA (Fig. 1C). Gene expression assay in the validation cohort confirmed that circ_3205 was expressed only in positive samples and its amount positively correlated with that of Spike (S) mRNA and SARS-CoV-2 viral load (*r* values = 0.80952 and 0.84867, *p* values (two sided) = 0.015 and 0.016, respectively, Spearman’s correlation test) (Fig. 2). The expression of S mRNA positively correlated with SARS-CoV-2 viral load, too (*r* value = 0.92582, *p* values (two sided) = 0.003) (Fig. 2).

### Human (hsa) miRNAs 298, 3940-5p, 4640-5p, 6081, and 6133 are predicted to interact with circ_3205 and their targets are potentially involved in cell response to viral infection

**Fig. 3:**
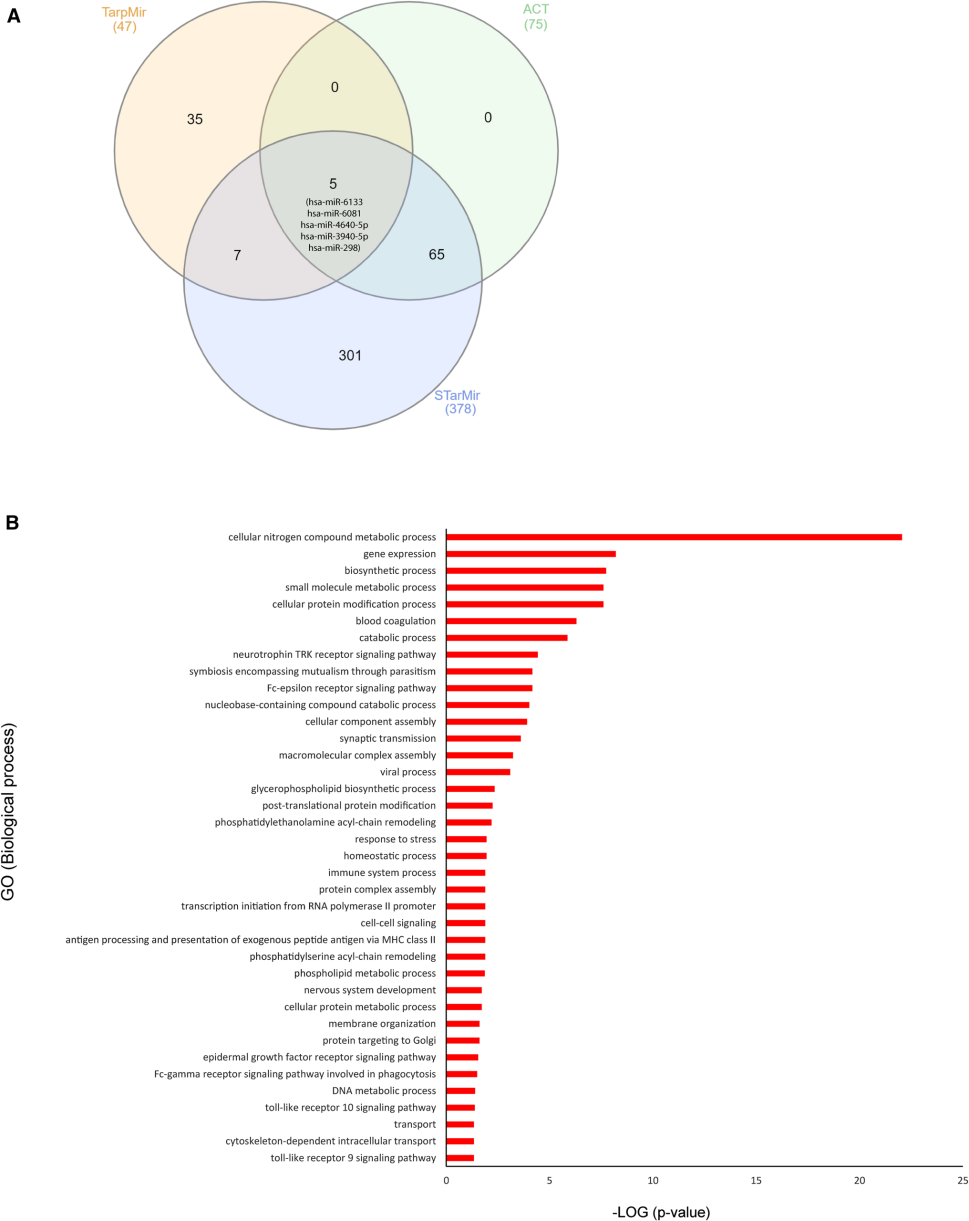
**A** Venn diagrams showing the number of miRNAs predicted to interact with circ_3205 by TarpMir, ACT and STarMiR predictive tools. The total number of miRNAs predicted by each tool is reported in brackets, while the number of miRNAs, either specifically predicted by only one or by more than one predictive tool, is reported (without brackets) within the Venn diagrams. The name of the five candidate miRNAs predicted to interact with circ_3205 by all the three predictive tools is reported in extenso. **B** Horizontal bar chart representing the enrichment of biological processes carried out by the predicted targets of hsa miRNAs 298, 3940-5p, 4640-5p, 6081, and 6133. Biological processes are reported in y-axis; statistical significance is reported as -LOG (*p* value) (x-axis)

Hsa miRNAs 298, 3940-5p, 4640-5p, 6081 and 6133 were predicted to interact with circ_3205 by all the three predictive algorithms queried (TarpMiR, ACT and STarMir) (Fig. 3A). BP GO analysis highlighted a potential involvement of the mRNA targets regulated by hsa miRNAs 298, 3940-5p, 4640-5p, 6081 and 6133 in SARS-CoV-2 infection-related processes (blood coagulation and immune response) (Fig. 3B).

### Hsa-miR-298 is predicted to target mRNAs coding for proteins involved in blood coagulation and immune response

**Fig. 4:**
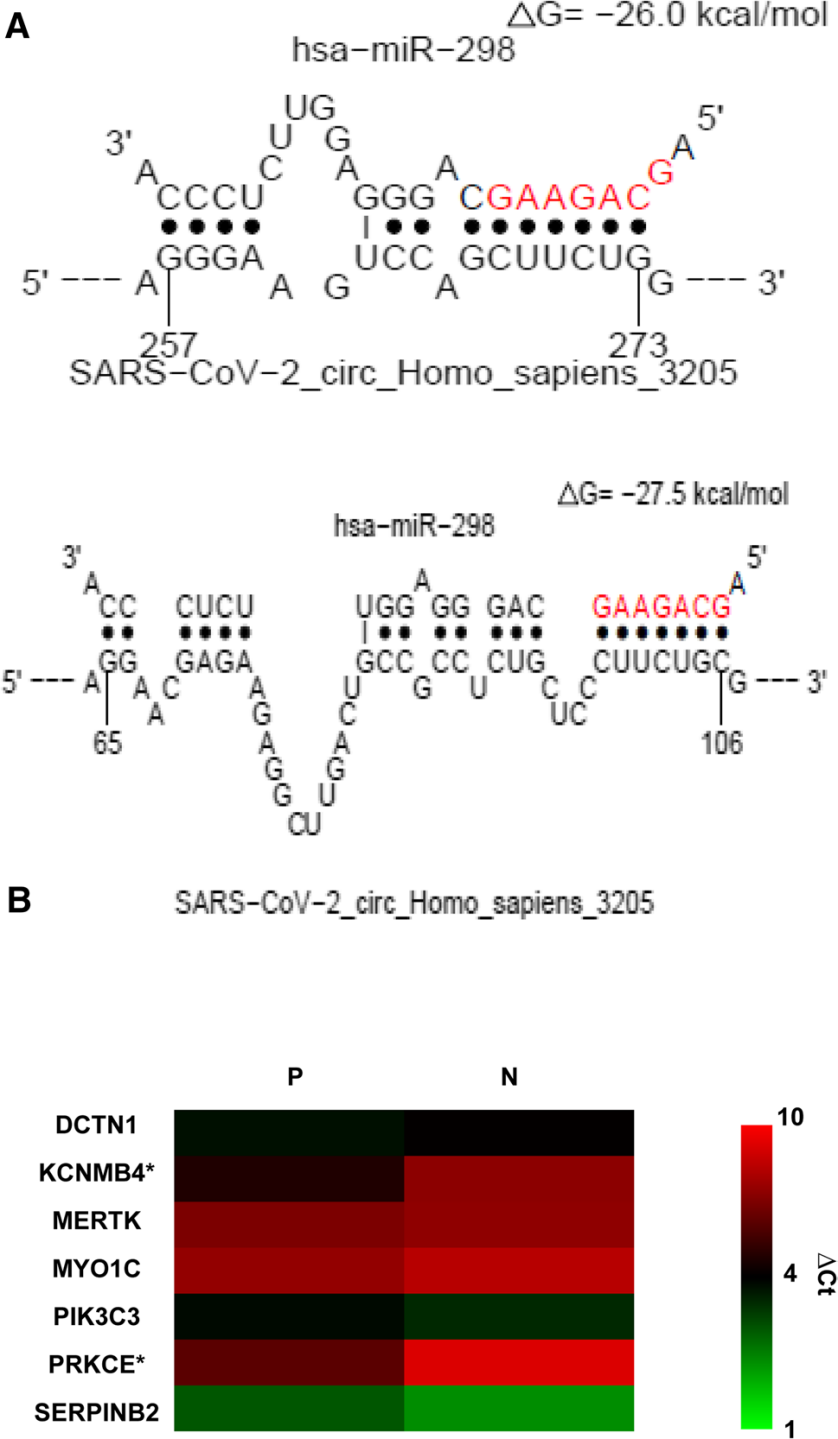
**A** Graphical representation of the interaction predicted by STarMiR between hsa-miR-298 and circ_3205. MiRNA seed region is showed in red. The numbers below vertical bars indicate the nucleotide position of circ_3205. **B** Heatmap showing the expression of seven predicted hsa-miR-298 targets whose expression was revealed in positive (P) and negative (N) samples by real-time PCR. Expression is reported as DCt: the lower is this value, the higher is the expression of the target and viceversa. * = statistically significant DE transcript (*p* value < 0.05, Student’s *t* test)

Based on (i) literature data, (ii) our GO analysis, and (iii) the high probability of interaction with SARS-CoV-2_circ_3205, we focused on hsa-mir-298 (Fig. 4A). Has-miR-298 was predicted to target 18 and 30 mRNAs involved in blood coagulation and immune response, respectively, based on DIANA miRPath analysis (Supplemental Table 3). Ten predicted mRNA targets (*CPB2; DCTN1; FN1; KCNMB4; MERTK; MYO1C; PDE1A; PIK3C3; PRKCE; SERPINB2*) were selected as candidates because of their known upregulation in biological specimens from SARS-CoV-2-infected individuals, as compared with UCs (Supplemental Table 4).

### Potassium calcium-activated channel subfamily M regulatory beta subunit 4 (*KCNMB4*) and protein kinase C epsilon (*PRKCE*) mRNAs are upregulated in positive samples as compared to UCs and their expression positively correlates with that of circ_3205

Quantitative real-time PCR detected the expression of seven (*DCTN1; KCNMB4; MERTK; MYO1C; PIK3C3; PRKCE; SERPINB2*) out of the ten predicted hsa-miR-298 targets in the analyzed samples. Among them, *KCNMB4* and *PRKCE* were 6 and 8.1-fold more expressed in positive samples as compared to UCs (*p* values = 0.049 and 0.02, Student’s *t* test, respectively) and their expression positively correlated with that of circ_3205 (*r* values = 0.6 and 0.25, Spearman’s correlation test, respectively) (Fig. 4B).

### PPI network of KCNMB4 and PRKCE is enriched in biological processes related to immune response and blood coagulation

**Fig. 5:**
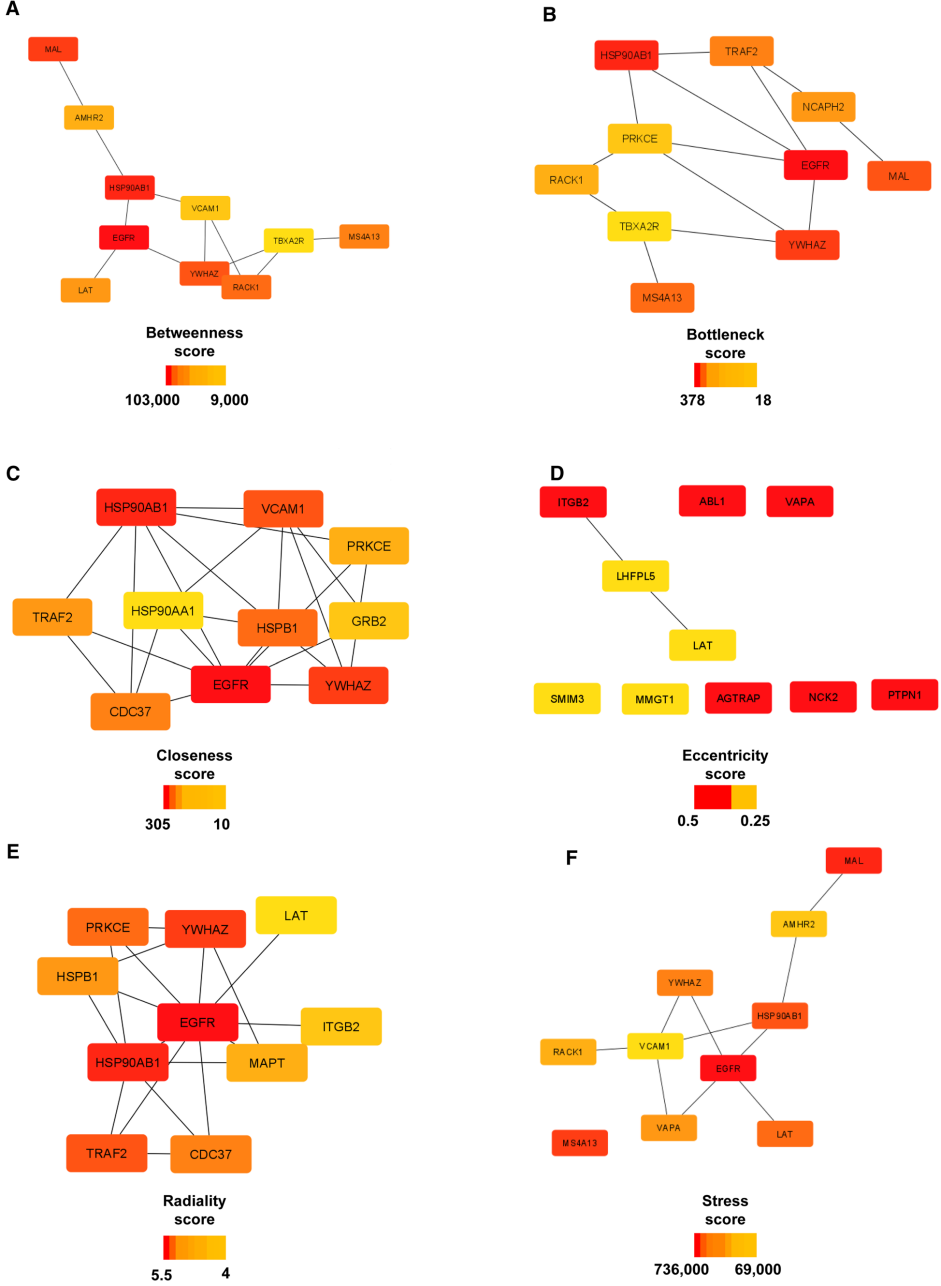
Analysis of centralities of KCNMB4 and PRKCE’s PPI network. Subnetworks show the top ten most central nodes calculated by **A** betweenness, **B** bottleneck, **C** closeness, **D** eccentricity, **E** radiality, **F** stress methods. For each subnetwork the scalebar reporting the scores of centrality is shown

**Fig. 6:**
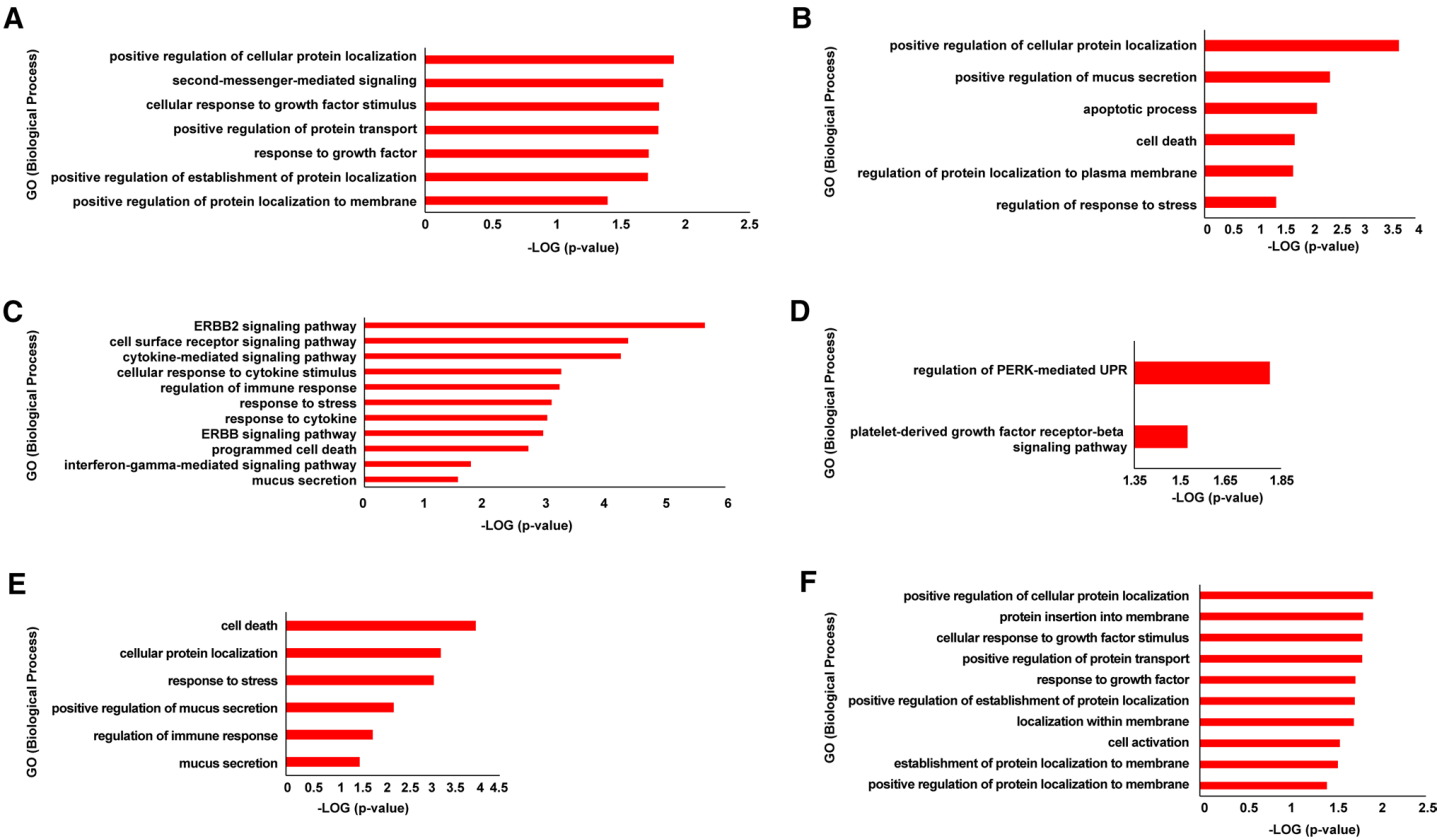
Functional enrichment of the subnetworks generated by **A** betweenness, **B** bottleneck, **C** closeness, **D** eccentricity, **E** radiality, **F** stress methods. Statistical significance of the functional enrichment within each subnetwork is reported as -LOG (*p* value) and BPs are arranged in y-axis from the higher to the lower -LOG (*p* value)

PPI network generated by HuRI consisted of 482 nodes and 1452 edges. GO analysis of the whole network revealed an over-representation of biological functions linked to blood coagulation, immune response, and inflammation (Supplemental Fig.1). The analysis of centralities of the network revealed a total of 27 most central proteins: among them, EGFR, HSP90AB1, YWHAZ occurred in five out of six subnetworks made of the most central nodes (Fig. 5). The generated subnetworks revealed an enrichment in BPs related to SARS-CoV-2 infection program (Fig. 6).

## Discussion

The capability of viruses to synthesize circRNAs upon infection has been ascertained, especially in DNA viruses [54–60]. As an example, Epstein Barr virus (EBV), a double stranded DNA virus belonging to the *Herpesviridae* family, is known to produce about 30 circRNAs during different phases of its infection [61] and some of them (e.g.: circBART2.2) contribute to virus-induced carcinogenesis through the immune escape of nasopharyngeal carcinoma cells [62]. Kaposi’s sarcoma-associated herpesvirus (KSHV) generates circRNAs found to be inserted into virions and implied in several steps of the infection [63].

**Fig. 7:**
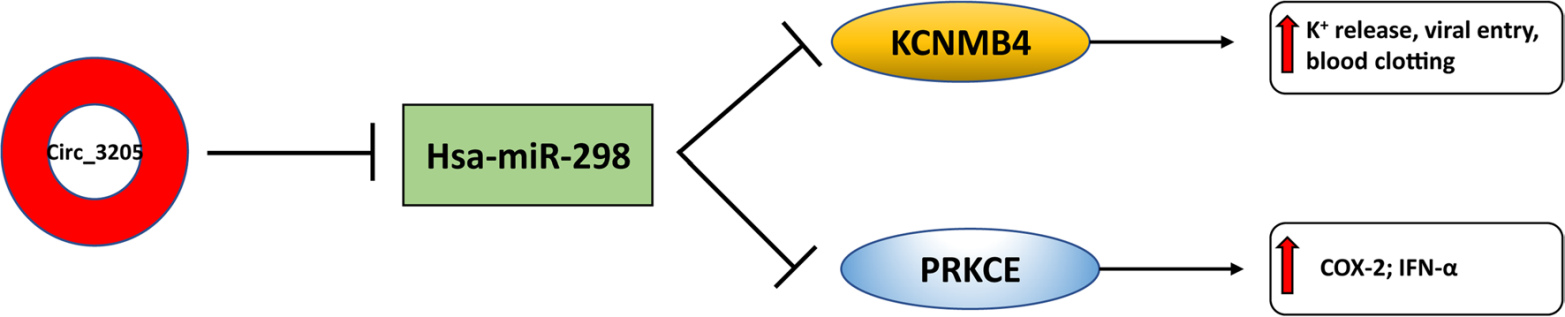
Schematic diagram of the ceRNA network mediated by SARS-CoV-2 circ_3205 in human infected cells

In this study, we first assayed the expression of eight circRNAs predicted to be synthesized by SARS-CoV-2 upon host cell infection. Based on our experimental results, we then focused on circRNA 3205. Based on data stored in VirusCircBase, circ_3205 was predicted to be synthesized from the negative RNA strand of SARS-CoV-2, specifically from a sequence embedded in the ORF coding for the N protein of the virus. Although the mechanism of circRNA biogenesis from RNA (+) viruses is under investigation, some hypotheses may be proposed: in a recent study, it was found that SARS-CoV-2 genome may be in part reverse transcribed and integrated as DNA into the host genome through a LINE1-mediated mechanism, leading to the production of chimeric viral-host cellular transcripts [64, 65]. Based on this study, it is conceivable that viral circRNAs could be generated through backsplicing from these chimeric viral-host transcripts. Nevertheless, the hypothesis of an integration of SARS-CoV-2 genome (or a part) into the host genome is debated [66]. An alternative path of circRNA biogenesis from RNA (+) viral genomes may consist in splicing-independent mechanisms occurring in the cytoplasm of host cells: this mechanism was previously described for IRE1alpha-mediated XBP1 mRNA splicing in mammalian cells, for IL1b transcripts in platelets and for the recently suggested miR-7-mediated circularization of the CDR1AS transcript [67–69]. Expression of viral circRNAs may perturb the ceRNA networks originally present within host cells or may create new ones [70]. Specifically, our data suggest that once synthesized within the host cell, circ_3205 may function as sponge for hsa-miR-298, allowing for the upregulation of targets involved in the progression through the infection (Fig. 7). MiR-298, together with miR-296, belongs to a genomic locus that is imprinted both in mice and humans [71, 72]. Interestingly, hsa-miR-298 has been predicted to bind the 5’-UTR of the SARS-CoV-2 genome, potentially altering its secondary structure and negatively impacting on its capability to be translated after infection of the host cell [73]. Chopra N. et al. identified hsa-miR-298 as a potential therapeutic agent for Alzheimer’s disease, because of its capability to negatively regulate the expression of human amyloid-β precursor protein (APP), β-site APP-converting enzyme 1 (BACE1) and specific tau protein isoforms [74]. Hsa-miR-298 has also been defined as oncomiRNA in several cancers, thanks to its ability to downregulate proapoptotic proteins such as BAX and PTEN [75, 76]. These literature data support our hypothesis that the circ_3205 sponge effect against hsa-miR-298 may contribute to the progression of the infection, by stabilizing the SARS-CoV-2 genome and triggering biological processes such as inflammation and apoptosis. Our data also shed light on two predicted targets of hsa-miR-298 (*KCNMB4* and *PRKCE*), which we found to be upregulated in positive samples and whose expression positively correlated with that of circ_3205. *KCNMB4* encodes a β4 subunit of a voltage-dependent K^+^ channel, belonging to the Ca2^+^-activated Slo subfamily (BK) [77, 78]. Upregulation of *KCNMB4* correlates with the increased intracellular concentration of Ca^2+^ observed during SARS-CoV-2 infection [79]. Even though the function exerted by BK channels during the SARS-CoV-2 infection needs further investigation, the role of K^+^ concentration and K^+^ channels in facilitating the entry of some viruses into the host cells has been convincingly ascertained [80, 81]. Furthermore, abnormalities in electrolyte serum concentrations (especially sodium, potassium, calcium and chloride) have been found to be related with the prognosis of COVID-19 patients and with the possibility to develop blood clots [82–84]. *PRKCE* encodes a Ca^2+^-independent protein kinase, belonging to the subfamily of nonconventional protein kinase C (PKCs); it has been described as involved in SARS-CoV infection, through the calcium-independent PI3K/PKCε/JNK/CREB pathway; this, in turn, induces COX-2 expression upon the interaction between viral S protein and cellular receptors [85]. *COX-2* has been found upregulated in several cell types also after SARS-CoV-2 infection [86]. *PRKCE* expression is further induced by Interferon-α (IFN-α), one of the first cytokines synthesized in infected cells on innate immune response [87]. Due to an abnormal recruitment of proinflammatory cells, IFN-α signaling over a prolonged period of time is known to cause an uncontrolled inflammatory response and potential organ failure in tissues infected by SARS-CoV-2 as well as other respiratory viruses [88, 89]. The study of the PPI network, generated starting from KCNMB4 and PRKCE, revealed Heat Shock Protein 90, Alpha family, class B member 1 (HSP90AB1) as one of the most central nodes, according to five out of six parameters of network centrality. HSP90AB1 is a first neighbor interactant of PRKCE and belongs to the Heat Shock Protein 90 (HSP90) family. Some members of HSP90 family have been recently suggested to foster MERS-CoV, SARS-CoV and SARS-CoV-2 replication and proinflammatory cytokine expression [90, 91]. Subnetworks generated by the study of centralities further revealed an enrichment in BPs strictly related to the local and systemic effects of SARS-CoV-2 infection, such as remodeling of protein trafficking within infected host cell [92], mucus hypersecretion [93], ErbB protein family and growth factor receptor signaling [94, 95], and unfolded protein response [96]. Collectively, these findings corroborate our hypothesis of a functional involvement of KCNMB4 and PRKCE in SARS-CoV-2 infection.

## Conclusions

Based on the integration of our experimental data and predictive analysis, we propose SARS-Cov-2_circ_3205/hsa-miR-298/KCNMB4 and SARS-Cov-2_circ_3205/hsa-miR-298/PRKCE molecular axes as involved in the progression of SARS-Cov-2 infection and, more in detail, in the related processes of blood clotting and immune response, respectively (Fig. 7).

## Supplementary Information

Below is the link to the electronic supplementary material.

Supplementary file1 (DOCX 15 KB)

Supplementary file2 (DOCX 712 KB)

Supplementary file3 (DOCX 18 KB)

Supplementary file4 (DOCX 14 KB)

Supplementary file5 (TIF 2275 KB)

## Data Availability

Data available on request from the authors.
