# Supplementary material for: Competing endogenous RNA network mediated by circ_3205 in SARS-CoV-2 infected cells
Source: Cell Mol Life Sci. 2022 Jan 17;79(2):75. doi: 10.1007/s00018-021-04119-8 (PMC8763136; doi:10.1007/s00018-021-04119-8)
Supplement: Supplementary file 1 — Supplementary file1 (DOCX 15 KB) [file 18_2021_4119_MOESM1_ESM.docx]

| **Transcript name** | **Fw** | **Rev** | **TaqMan Probe ID** |
| --- | --- | --- | --- |
| CPB2 | ATGCGAACAATCATTGCATCG | CAGTAGGTTTCCGAGCATGAG |  |
| DCTN1 | GGATGCGGGATCTTTCTTCCT | CAACTCGCGCACTTTCTCTTC |  |
| FN1 | CGGTGGCTGTCAGTCAAAG | AAACCTCGGCTTCCTCCATAA |  |
| GAPDH | GTCAGCCGCATCTTCTTTTG | GCGCCCAATACGACCAAATC |  |
| KCNMB4 | GGTCTACGTGAACAACTCTGAG | GGAGGGATATAGGAGCACTTGG |  |
| MERTK | CTCTGGCGTAGAGCTATCACT | AGGCTGGGTTGGTGAAAACA |  |
| MYO1C | ACGACAAGAGTGACTGGAAGG | GCAAAGTGGATGTTGCCCAAAT |  |
| PDE1A | ATGGGGTCTAGTGCCACAGAG | GCACAGATGCCGCATATTCAAT |  |
| PIK3C3 | TAGGAGGAACAACGGTTTCGC | GCTTCTACATTAGGCCAGACTTT |  |
| PRKCE | CAACGGACGCAAGATCGAG | CTGGCTCCAGATCAATCCAGT |  |
| S | CGGGTGTGACCGAAAGGTAA | CAGGGGGTAATTGAGTTCTGGT |  |
| SERPINB2 | TCCTGGGTCAAGACTCAAACC | CATCCTGGTATCCCCATCTACAG |  |
| Circ_363 | CGAGCAACATAAGCCCGTTA | AAGACGGCAGTGAGGACAAT |  |
| Circ_368 | CAACCAATGGAGCTTCAACAG | ACATGGAGGAGGTGTTGCAG |  |
| Circ_2667 | GCATAAAGATAGAGAAAAGGGGC | TGGAACCAATTTATGATGAACCG |  |
| Circ_2670 | GCATAAAGATAGAGAAAAGGGGC | GACTACTAGCGTGCCTTTGTAA |  |
| Circ_2685 | GCATAAAGATAGAGAAAAGGGGC | GGCAGATTCCAACGGTACTATTA |  |
| Circ_2795 | TGCTTTGCTTGTACAGTAAGTGA | AGTTTCTGGATTGAATGACCACA |  |
| Circ_3058 | ACAACAAGGCCAAACTGTCA | ATGTTGAGTGAGAGCGGTGA |  |
| Circ_3205 | GCCGTCTTTGTTAGCACCAT | GAAATTCAACTCCAGGCAGCA |  |
| Hsa-miR-298 |  |  | 002190 (TaqMan® MicroRNA Assays, ThermoFisher Scientific) |

**Supplemental table 1.** List of primers and TaqMan probe used in this study
