# Supplementary material for: Competing endogenous RNA network mediated by circ_3205 in SARS-CoV-2 infected cells
Source: Cell Mol Life Sci. 2022 Jan 17;79(2):75. doi: 10.1007/s00018-021-04119-8 (PMC8763136; doi:10.1007/s00018-021-04119-8)
Supplement: Supplementary file 2 — Supplementary file2 (DOCX 712 KB) [file 18_2021_4119_MOESM2_ESM.docx]

| **circRNA name** | **Viral genome** | **Start** | **End** | **Strand** | **Gene** | **Junction read counts** | **Detection method** | **Sample** |
| --- | --- | --- | --- | --- | --- | --- | --- | --- |
| SARS-CoV-2_circ_Homo_sapiens_2956 | MN908947.3 | 27644 | 28895 | - | ORF7a;ORF8;ORF7b;N | 193 | CIRI2 | SRR11550045 |
| SARS-CoV-2_circ_Homo_sapiens_2956 | MN908947.3 | 27644 | 28895 | - | ORF7a;ORF8;ORF7b;N | 164 | CIRI2 | SRR11550044 |
| SARS-CoV-2_circ_Homo_sapiens_2958 | MN908947.3 | 27644 | 29012 | - | ORF7a;ORF8;ORF7b;N | 156 | CIRI2 | SRR11550046 |
| SARS-CoV-2_circ_Homo_sapiens_877 | MN908947.3 | 8293 | 27130 | - | M;E;ORF3a;S;ORF1ab | 144 | CIRI2 | SRR11550043 |
| SARS-CoV-2_circ_Homo_sapiens_1284 | MN908947.3 | 11817 | 26570 | - | M;E;ORF3a;S;ORF1ab | 130 | CIRI2 | SRR11550043 |
| SARS-CoV-2_circ_Homo_sapiens_2670 | MN908947.3 | 25672 | 26241 | - | ORF3a | 114 | CIRI2 | SRR11550045 |
| SARS-CoV-2_circ_Homo_sapiens_630 | MN908947.3 | 4921 | 26241 | - | ORF3a;S;ORF1ab | 103 | CIRI2 | SRR11550045 |
| SARS-CoV-2_circ_Homo_sapiens_2670 | MN908947.3 | 25672 | 26241 | - | ORF3a | 94 | CIRI2 | SRR11550046 |
| SARS-CoV-2_circ_Homo_sapiens_223 | MN908947.3 | 935 | 29268 | - | ORF6;M;E;ORF3a;S;N;ORF8;ORF7a;ORF7b;ORF1ab | 87 | CIRI2 | SRR11550045 |
| SARS-CoV-2_circ_Homo_sapiens_2959 | MN908947.3 | 27644 | 29066 | - | ORF7a;ORF8;ORF7b;N | 82 | CIRI2 | SRR11550045 |
| SARS-CoV-2_circ_Homo_sapiens_385 | MN908947.3 | 2676 | 3542 | - | ORF1ab | 81 | CIRI2 | SRR11550045 |
| SARS-CoV-2_circ_Homo_sapiens_2685 | MN908947.3 | 25672 | 26570 | - | ORF3a;M;E | 77 | CIRI2 | SRR11550046 |
| SARS-CoV-2_circ_Homo_sapiens_368 | MN908947.3 | 2571 | 3539 | - | ORF1ab | 76 | CIRI2 | SRR11550045 |
| SARS-CoV-2_circ_Homo_sapiens_368 | MN908947.3 | 2571 | 3539 | - | ORF1ab | 76 | CIRI2 | SRR11550046 |
| SARS-CoV-2_circ_Homo_sapiens_2959 | MN908947.3 | 27644 | 29066 | - | ORF7a;ORF8;ORF7b;N | 69 | CIRI2 | SRR11550044 |
| SARS-CoV-2_circ_Homo_sapiens_34 | MN908947.3 | 8 | 29572 | + | ORF6;ORF10;M;E;ORF3a;S;N;ORF8;ORF7a;ORF7b;ORF1ab | 67 | CIRI2 | SRR11550045 |
| SARS-CoV-2_circ_Homo_sapiens_2641 | MN908947.3 | 25564 | 27225 | + | ORF6;ORF3a;M;E | 62 | CIRI2 | SRR11550046 |
| SARS-CoV-2_circ_Homo_sapiens_34 | MN908947.3 | 8 | 29572 | + | ORF6;ORF10;M;E;ORF3a;S;N;ORF8;ORF7a;ORF7b;ORF1ab | 60 | CIRI2 | SRR11550044 |
| SARS-CoV-2_circ_Homo_sapiens_2695 | MN908947.3 | 25672 | 26817 | - | ORF3a;M;E | 53 | CIRI2 | SRR11550045 |
| SARS-CoV-2_circ_Homo_sapiens_2244 | MN908947.3 | 22377 | 28895 | - | ORF6;M;E;ORF3a;S;N;ORF8;ORF7a;ORF7b | 52 | CIRI2 | SRR11550043 |
| SARS-CoV-2_circ_Homo_sapiens_1600 | MN908947.3 | 14178 | 29579 | - | ORF6;ORF10;M;E;ORF3a;S;N;ORF8;ORF7a;ORF7b;ORF1ab | 49 | CIRI2 | SRR11550044 |
| SARS-CoV-2_circ_Homo_sapiens_2484 | MN908947.3 | 24150 | 26570 | - | ORF3a;S;M;E | 48 | CIRI2 | SRR11550046 |
| SARS-CoV-2_circ_Homo_sapiens_2695 | MN908947.3 | 25672 | 26817 | - | ORF3a;M;E | 48 | CIRI2 | SRR11550046 |
| SARS-CoV-2_circ_Homo_sapiens_2294 | MN908947.3 | 22950 | 26780 | - | ORF3a;S;M;E | 47 | CIRI2 | SRR11550046 |
| SARS-CoV-2_circ_Homo_sapiens_2955 | MN908947.3 | 27644 | 28727 | - | ORF7a;ORF8;ORF7b;N | 47 | CIRI2 | SRR11550046 |
| SARS-CoV-2_circ_Homo_sapiens_2293 | MN908947.3 | 22950 | 26241 | - | ORF3a;S | 43 | CIRI2 | SRR11550044 |
| SARS-CoV-2_circ_Homo_sapiens_2685 | MN908947.3 | 25672 | 26570 | - | ORF3a;M;E | 43 | CIRI2 | SRR11550045 |
| SARS-CoV-2_circ_Homo_sapiens_5 | MN908947.3 | 8 | 442 | + | ORF1ab | 43 | CIRI2 | SRR11550046 |
| SARS-CoV-2_circ_Homo_sapiens_1105 | MN908947.3 | 10605 | 26780 | - | M;E;ORF3a;S;ORF1ab | 40 | CIRI2 | SRR11550045 |
| SARS-CoV-2_circ_Homo_sapiens_368 | MN908947.3 | 2571 | 3539 | - | ORF1ab | 39 | CIRI2 | SRR11550044 |
| SARS-CoV-2_circ_Homo_sapiens_363 | MN908947.3 | 2571 | 3263 | - | ORF1ab | 38 | CIRI2 | SRR11550045 |
| SARS-CoV-2_circ_Homo_sapiens_3134 | MN908947.3 | 28463 | 28895 | - | N | 35 | find_circ | SRR11550045 |
| SARS-CoV-2_circ_Homo_sapiens_2667 | MN908947.3 | 25672 | 26212 | - | ORF3a | 34 | CIRI2 | SRR11550045 |
| SARS-CoV-2_circ_Homo_sapiens_2667 | MN908947.3 | 25672 | 26212 | - | ORF3a | 34 | CIRI2 | SRR11550044 |
| SARS-CoV-2_circ_Homo_sapiens_2685 | MN908947.3 | 25672 | 26570 | - | ORF3a;M;E | 34 | CIRI2 | SRR11550043 |
| SARS-CoV-2_circ_Homo_sapiens_3237 | MN908947.3 | 28696 | 28904 | - | N | 34 | find_circ | SRR11550045 |
| SARS-CoV-2_circ_Homo_sapiens_2670 | MN908947.3 | 25672 | 26241 | - | ORF3a | 33 | CIRI2 | SRR11550043 |
| SARS-CoV-2_circ_Homo_sapiens_3058 | MN908947.3 | 28404 | 29080 | + | N | 33 | find_circ | SRR11550045 |
| SARS-CoV-2_circ_Homo_sapiens_3134 | MN908947.3 | 28463 | 28895 | - | N | 33 | find_circ | SRR11550044 |
| SARS-CoV-2_circ_Homo_sapiens_2667 | MN908947.3 | 25672 | 26212 | - | ORF3a | 32 | CIRI2 | SRR11550046 |
| SARS-CoV-2_circ_Homo_sapiens_3062 | MN908947.3 | 28404 | 29572 | + | ORF10;N | 32 | CIRI2 | SRR11550046 |
| SARS-CoV-2_circ_Homo_sapiens_1103 | MN908947.3 | 10605 | 26775 | - | M;E;ORF3a;S;ORF1ab | 31 | CIRI2 | SRR11550046 |
| SARS-CoV-2_circ_Homo_sapiens_2712 | MN908947.3 | 25672 | 27678 | - | ORF6;M;E;ORF3a;ORF7a | 30 | CIRI2 | SRR11550044 |
| SARS-CoV-2_circ_Homo_sapiens_3058 | MN908947.3 | 28404 | 29080 | + | N | 30 | circRNA_finder | SRR11550045 |
| SARS-CoV-2_circ_Homo_sapiens_3223 | MN908947.3 | 28652 | 29012 | - | N | 30 | find_circ | SRR11550045 |
| SARS-CoV-2_circ_Homo_sapiens_7 | MN908947.3 | 8 | 520 | + | ORF1ab | 30 | find_circ | SRR11550045 |
| SARS-CoV-2_circ_Homo_sapiens_21 | MN908947.3 | 8 | 9553 | + | ORF1ab | 29 | CIRI2 | SRR11550046 |
| SARS-CoV-2_circ_Homo_sapiens_2104 | MN908947.3 | 20405 | 25516 | - | ORF3a;S;ORF1ab | 29 | CIRI2 | SRR11550045 |
| SARS-CoV-2_circ_Homo_sapiens_2695 | MN908947.3 | 25672 | 26817 | - | ORF3a;M;E | 28 | CIRI2 | SRR11550043 |
| SARS-CoV-2_circ_Homo_sapiens_2294 | MN908947.3 | 22950 | 26780 | - | ORF3a;S;M;E | 27 | CIRI2 | SRR11550043 |
| SARS-CoV-2_circ_Homo_sapiens_2976 | MN908947.3 | 27780 | 29268 | - | ORF8;ORF7b;N | 27 | CIRI2 | SRR11550045 |
| SARS-CoV-2_circ_Homo_sapiens_3134 | MN908947.3 | 28463 | 28895 | - | N | 27 | find_circ | SRR11550046 |
| SARS-CoV-2_circ_Homo_sapiens_2710 | MN908947.3 | 25672 | 27218 | - | ORF6;ORF3a;M;E | 26 | CIRI2 | SRR11550045 |
| SARS-CoV-2_circ_Homo_sapiens_3134 | MN908947.3 | 28463 | 28895 | - | N | 26 | find_circ | SRR11550043 |
| SARS-CoV-2_circ_Homo_sapiens_3205 | MN908947.3 | 28610 | 28895 | - | N | 26 | CIRI2 | SRR11550044 |
| SARS-CoV-2_circ_Homo_sapiens_1963 | MN908947.3 | 18356 | 25548 | - | ORF3a;S;ORF1ab | 25 | CIRI2 | SRR11550045 |
| SARS-CoV-2_circ_Homo_sapiens_3082 | MN908947.3 | 28410 | 29012 | - | N | 25 | find_circ | SRR11550045 |
| SARS-CoV-2_circ_Homo_sapiens_3237 | MN908947.3 | 28696 | 28904 | - | N | 25 | find_circ | SRR11550046 |
| SARS-CoV-2_circ_Homo_sapiens_2976 | MN908947.3 | 27780 | 29268 | - | ORF8;ORF7b;N | 24 | CIRI2 | SRR11550046 |
| SARS-CoV-2_circ_Homo_sapiens_3192 | MN908947.3 | 28607 | 28904 | - | N | 24 | find_circ | SRR11550045 |
| SARS-CoV-2_circ_Homo_sapiens_3222 | MN908947.3 | 28652 | 28895 | - | N | 24 | find_circ | SRR11550045 |
| SARS-CoV-2_circ_Homo_sapiens_3222 | MN908947.3 | 28652 | 28895 | - | N | 24 | find_circ | SRR11550043 |
| SARS-CoV-2_circ_Homo_sapiens_7 | MN908947.3 | 8 | 520 | + | ORF1ab | 24 | find_circ | SRR11550046 |
| SARS-CoV-2_circ_Homo_sapiens_2976 | MN908947.3 | 27780 | 29268 | - | ORF8;ORF7b;N | 23 | CIRI2 | SRR11550044 |
| SARS-CoV-2_circ_Homo_sapiens_3135 | MN908947.3 | 28463 | 28895 | + | N | 23 | circRNA_finder | SRR11550043 |
| SARS-CoV-2_circ_Homo_sapiens_368 | MN908947.3 | 2571 | 3539 | - | ORF1ab | 23 | CIRI2 | SRR11550043 |
| SARS-CoV-2_circ_Homo_sapiens_1574 | MN908947.3 | 14178 | 14535 | - | ORF1ab | 22 | CIRI2 | SRR11550046 |
| SARS-CoV-2_circ_Homo_sapiens_2695 | MN908947.3 | 25672 | 26817 | - | ORF3a;M;E | 22 | CIRI2 | SRR11550044 |
| SARS-CoV-2_circ_Homo_sapiens_3135 | MN908947.3 | 28463 | 28895 | + | N | 22 | circRNA_finder | SRR11550044 |
| SARS-CoV-2_circ_Homo_sapiens_363 | MN908947.3 | 2571 | 3263 | - | ORF1ab | 22 | CIRI2 | SRR11550046 |
| SARS-CoV-2_circ_Homo_sapiens_928 | MN908947.3 | 8922 | 14575 | - | ORF1ab | 22 | CIRI2 | SRR11550045 |
| SARS-CoV-2_circ_Homo_sapiens_2795 | MN908947.3 | 26825 | 27225 | + | ORF6;M | 21 | find_circ | SRR11550045 |
| SARS-CoV-2_circ_Homo_sapiens_3120 | MN908947.3 | 28463 | 28727 | - | N | 21 | find_circ | SRR11550043 |
| SARS-CoV-2_circ_Homo_sapiens_3317 | MN908947.3 | 29086 | 29534 | - | N | 21 | find_circ | SRR11550044 |
| SARS-CoV-2_circ_Homo_sapiens_385 | MN908947.3 | 2676 | 3542 | - | ORF1ab | 21 | CIRI2 | SRR11550044 |
| SARS-CoV-2_circ_Homo_sapiens_1412 | MN908947.3 | 12624 | 13139 | - | ORF1ab | 20 | CIRI2 | SRR11550046 |
| SARS-CoV-2_circ_Homo_sapiens_2291 | MN908947.3 | 22950 | 25516 | - | ORF3a;S | 20 | CIRI2 | SRR11550043 |
| SARS-CoV-2_circ_Homo_sapiens_2843 | MN908947.3 | 27060 | 28146 | - | ORF6;M;ORF8;ORF7a;ORF7b | 20 | CIRI2 | SRR11550043 |
| SARS-CoV-2_circ_Homo_sapiens_3096 | MN908947.3 | 28420 | 28609 | - | N | 20 | find_circ | SRR11550046 |
| SARS-CoV-2_circ_Homo_sapiens_874 | MN908947.3 | 8293 | 13139 | - | ORF1ab | 20 | CIRI2 | SRR11550046 |
| SARS-CoV-2_circ_Homo_sapiens_2518 | MN908947.3 | 24463 | 25061 | + | S | 19 | CIRI2 | SRR11550044 |
| SARS-CoV-2_circ_Homo_sapiens_2667 | MN908947.3 | 25672 | 26212 | - | ORF3a | 19 | CIRI2 | SRR11550043 |
| SARS-CoV-2_circ_Homo_sapiens_2685 | MN908947.3 | 25672 | 26570 | - | ORF3a;M;E | 19 | CIRI2 | SRR11550044 |
| SARS-CoV-2_circ_Homo_sapiens_2692 | MN908947.3 | 25672 | 26780 | - | ORF3a;M;E | 19 | CIRI2 | SRR11550044 |
| SARS-CoV-2_circ_Homo_sapiens_3120 | MN908947.3 | 28463 | 28727 | - | N | 19 | find_circ | SRR11550044 |
| SARS-CoV-2_circ_Homo_sapiens_3205 | MN908947.3 | 28610 | 28895 | - | N | 19 | CIRI2 | SRR11550045 |
| SARS-CoV-2_circ_Homo_sapiens_5 | MN908947.3 | 8 | 442 | + | ORF1ab | 19 | CIRI2 | SRR11550043 |
| SARS-CoV-2_circ_Homo_sapiens_1461 | MN908947.3 | 12974 | 14598 | - | ORF1ab | 18 | CIRI2 | SRR11550045 |
| SARS-CoV-2_circ_Homo_sapiens_2710 | MN908947.3 | 25672 | 27218 | - | ORF6;ORF3a;M;E | 18 | CIRI2 | SRR11550044 |
| SARS-CoV-2_circ_Homo_sapiens_2795 | MN908947.3 | 26825 | 27225 | + | ORF6;M | 18 | find_circ | SRR11550044 |
| SARS-CoV-2_circ_Homo_sapiens_2670 | MN908947.3 | 25672 | 26241 | - | ORF3a | 17 | CIRI2 | SRR11550044 |
| SARS-CoV-2_circ_Homo_sapiens_2685 | MN908947.3 | 25672 | 26570 | - | ORF3a;M;E | 17 | find_circ | SRR11550046 |
| SARS-CoV-2_circ_Homo_sapiens_3193 | MN908947.3 | 28607 | 28904 | + | N | 17 | circRNA_finder | SRR11550045 |
| SARS-CoV-2_circ_Homo_sapiens_2291 | MN908947.3 | 22950 | 25516 | - | ORF3a;S | 16 | CIRI2 | SRR11550044 |
| SARS-CoV-2_circ_Homo_sapiens_2518 | MN908947.3 | 24463 | 25061 | + | S | 16 | CIRI2 | SRR11550043 |
| SARS-CoV-2_circ_Homo_sapiens_2670 | MN908947.3 | 25672 | 26241 | - | ORF3a | 16 | find_circ | SRR11550044 |
| SARS-CoV-2_circ_Homo_sapiens_2730 | MN908947.3 | 25758 | 26276 | + | ORF3a;E | 16 | find_circ | SRR11550045 |
| SARS-CoV-2_circ_Homo_sapiens_2752 | MN908947.3 | 26156 | 26778 | + | ORF3a;M;E | 16 | find_circ | SRR11550046 |
| SARS-CoV-2_circ_Homo_sapiens_3026 | MN908947.3 | 28321 | 29012 | - | N | 16 | find_circ | SRR11550045 |
| SARS-CoV-2_circ_Homo_sapiens_3135 | MN908947.3 | 28463 | 28895 | + | N | 16 | circRNA_finder | SRR11550045 |
| SARS-CoV-2_circ_Homo_sapiens_3205 | MN908947.3 | 28610 | 28895 | - | N | 16 | find_circ | SRR11550044 |
| SARS-CoV-2_circ_Homo_sapiens_3223 | MN908947.3 | 28652 | 29012 | - | N | 16 | find_circ | SRR11550046 |
| SARS-CoV-2_circ_Homo_sapiens_1101 | MN908947.3 | 10605 | 25516 | - | ORF3a;S;ORF1ab | 15 | CIRI2 | SRR11550046 |
| SARS-CoV-2_circ_Homo_sapiens_1461 | MN908947.3 | 12974 | 14598 | - | ORF1ab | 15 | CIRI2 | SRR11550046 |
| SARS-CoV-2_circ_Homo_sapiens_1958 | MN908947.3 | 18356 | 20198 | - | ORF1ab | 15 | CIRI2 | SRR11550043 |
| SARS-CoV-2_circ_Homo_sapiens_3026 | MN908947.3 | 28321 | 29012 | - | N | 15 | find_circ | SRR11550046 |
| SARS-CoV-2_circ_Homo_sapiens_3026 | MN908947.3 | 28321 | 29012 | - | N | 15 | find_circ | SRR11550044 |
| SARS-CoV-2_circ_Homo_sapiens_3135 | MN908947.3 | 28463 | 28895 | + | N | 15 | circRNA_finder | SRR11550046 |
| SARS-CoV-2_circ_Homo_sapiens_3212 | MN908947.3 | 28610 | 29012 | - | N | 15 | CIRI2 | SRR11550045 |
| SARS-CoV-2_circ_Homo_sapiens_3225 | MN908947.3 | 28652 | 29066 | - | N | 15 | find_circ | SRR11550044 |
| SARS-CoV-2_circ_Homo_sapiens_3235 | MN908947.3 | 28658 | 29268 | - | N | 15 | CIRI2 | SRR11550044 |
| SARS-CoV-2_circ_Homo_sapiens_3309 | MN908947.3 | 29086 | 29329 | - | N | 15 | find_circ | SRR11550046 |
| SARS-CoV-2_circ_Homo_sapiens_3313 | MN908947.3 | 29086 | 29450 | - | N | 15 | find_circ | SRR11550046 |
| SARS-CoV-2_circ_Homo_sapiens_627 | MN908947.3 | 4921 | 5821 | - | ORF1ab | 15 | CIRI2 | SRR11550046 |
| SARS-CoV-2_circ_Homo_sapiens_757 | MN908947.3 | 5827 | 26078 | - | ORF3a;S;ORF1ab | 15 | CIRI2 | SRR11550046 |
| SARS-CoV-2_circ_Homo_sapiens_2 | MN908947.3 | 8 | 314 | + | ORF1ab | 14 | CIRI2 | SRR11550045 |
| SARS-CoV-2_circ_Homo_sapiens_2053 | MN908947.3 | 19605 | 19982 | - | ORF1ab | 14 | find_circ | SRR11550046 |
| SARS-CoV-2_circ_Homo_sapiens_2054 | MN908947.3 | 19605 | 19982 | + | ORF1ab | 14 | circRNA_finder | SRR11550046 |
| SARS-CoV-2_circ_Homo_sapiens_2397 | MN908947.3 | 23568 | 27187 | + | ORF3a;S;M;E | 14 | CIRI2 | SRR11550046 |
| SARS-CoV-2_circ_Homo_sapiens_2672 | MN908947.3 | 25672 | 26298 | - | ORF3a;E | 14 | CIRI2 | SRR11550043 |
| SARS-CoV-2_circ_Homo_sapiens_2795 | MN908947.3 | 26825 | 27225 | + | ORF6;M | 14 | find_circ | SRR11550046 |
| SARS-CoV-2_circ_Homo_sapiens_3051 | MN908947.3 | 28404 | 28850 | + | N | 14 | CIRI2 | SRR11550045 |
| SARS-CoV-2_circ_Homo_sapiens_3120 | MN908947.3 | 28463 | 28727 | - | N | 14 | find_circ | SRR11550046 |
| SARS-CoV-2_circ_Homo_sapiens_3121 | MN908947.3 | 28463 | 28727 | + | N | 14 | circRNA_finder | SRR11550044 |
| SARS-CoV-2_circ_Homo_sapiens_3146 | MN908947.3 | 28463 | 29012 | - | N | 14 | find_circ | SRR11550044 |
| SARS-CoV-2_circ_Homo_sapiens_3192 | MN908947.3 | 28607 | 28904 | - | N | 14 | find_circ | SRR11550044 |
| SARS-CoV-2_circ_Homo_sapiens_3206 | MN908947.3 | 28610 | 28895 | + | N | 14 | circRNA_finder | SRR11550044 |
| SARS-CoV-2_circ_Homo_sapiens_3222 | MN908947.3 | 28652 | 28895 | - | N | 14 | find_circ | SRR11550044 |
| SARS-CoV-2_circ_Homo_sapiens_3225 | MN908947.3 | 28652 | 29066 | - | N | 14 | find_circ | SRR11550045 |
| SARS-CoV-2_circ_Homo_sapiens_3309 | MN908947.3 | 29086 | 29329 | - | N | 14 | find_circ | SRR11550043 |
| SARS-CoV-2_circ_Homo_sapiens_3309 | MN908947.3 | 29086 | 29329 | - | N | 14 | find_circ | SRR11550045 |
| SARS-CoV-2_circ_Homo_sapiens_7 | MN908947.3 | 8 | 520 | + | ORF1ab | 14 | find_circ | SRR11550043 |
| SARS-CoV-2_circ_Homo_sapiens_1810 | MN908947.3 | 16751 | 27015 | - | M;E;ORF3a;S;ORF1ab | 13 | CIRI2 | SRR11550043 |
| SARS-CoV-2_circ_Homo_sapiens_2686 | MN908947.3 | 25672 | 26570 | + | ORF3a;M;E | 13 | circRNA_finder | SRR11550046 |
| SARS-CoV-2_circ_Homo_sapiens_2795 | MN908947.3 | 26825 | 27225 | + | ORF6;M | 13 | circRNA_finder | SRR11550045 |
| SARS-CoV-2_circ_Homo_sapiens_3051 | MN908947.3 | 28404 | 28850 | + | N | 13 | CIRI2 | SRR11550044 |
| SARS-CoV-2_circ_Homo_sapiens_3121 | MN908947.3 | 28463 | 28727 | + | N | 13 | circRNA_finder | SRR11550043 |
| SARS-CoV-2_circ_Homo_sapiens_3193 | MN908947.3 | 28607 | 28904 | + | N | 13 | circRNA_finder | SRR11550044 |
| SARS-CoV-2_circ_Homo_sapiens_3222 | MN908947.3 | 28652 | 28895 | - | N | 13 | find_circ | SRR11550046 |
| SARS-CoV-2_circ_Homo_sapiens_3252 | MN908947.3 | 28709 | 29066 | - | N | 13 | find_circ | SRR11550044 |
| SARS-CoV-2_circ_Homo_sapiens_3317 | MN908947.3 | 29086 | 29534 | - | N | 13 | find_circ | SRR11550045 |
| SARS-CoV-2_circ_Homo_sapiens_3318 | MN908947.3 | 29086 | 29534 | + | N | 13 | circRNA_finder | SRR11550044 |
| SARS-CoV-2_circ_Homo_sapiens_1169 | MN908947.3 | 11190 | 26780 | - | M;E;ORF3a;S;ORF1ab | 12 | CIRI2 | SRR11550045 |
| SARS-CoV-2_circ_Homo_sapiens_1351 | MN908947.3 | 12153 | 13385 | + | ORF1ab | 12 | find_circ | SRR11550046 |
| SARS-CoV-2_circ_Homo_sapiens_1548 | MN908947.3 | 13736 | 14177 | - | ORF1ab | 12 | CIRI2 | SRR11550045 |
| SARS-CoV-2_circ_Homo_sapiens_2671 | MN908947.3 | 25672 | 26241 | + | ORF3a | 12 | circRNA_finder | SRR11550044 |
| SARS-CoV-2_circ_Homo_sapiens_2692 | MN908947.3 | 25672 | 26780 | - | ORF3a;M;E | 12 | find_circ | SRR11550045 |
| SARS-CoV-2_circ_Homo_sapiens_2795 | MN908947.3 | 26825 | 27225 | + | ORF6;M | 12 | find_circ | SRR11550043 |
| SARS-CoV-2_circ_Homo_sapiens_2795 | MN908947.3 | 26825 | 27225 | + | ORF6;M | 12 | circRNA_finder | SRR11550043 |
| SARS-CoV-2_circ_Homo_sapiens_2795 | MN908947.3 | 26825 | 27225 | + | ORF6;M | 12 | circRNA_finder | SRR11550046 |
| SARS-CoV-2_circ_Homo_sapiens_3166 | MN908947.3 | 28463 | 29658 | - | ORF10;N | 12 | CIRI2 | SRR11550044 |
| SARS-CoV-2_circ_Homo_sapiens_3192 | MN908947.3 | 28607 | 28904 | - | N | 12 | find_circ | SRR11550046 |
| SARS-CoV-2_circ_Homo_sapiens_3193 | MN908947.3 | 28607 | 28904 | + | N | 12 | circRNA_finder | SRR11550046 |
| SARS-CoV-2_circ_Homo_sapiens_3205 | MN908947.3 | 28610 | 28895 | - | N | 12 | CIRI2 | SRR11550046 |
| SARS-CoV-2_circ_Homo_sapiens_3225 | MN908947.3 | 28652 | 29066 | - | N | 12 | find_circ | SRR11550043 |
| SARS-CoV-2_circ_Homo_sapiens_3348 | MN908947.3 | 29172 | 29529 | - | N | 12 | find_circ | SRR11550045 |
| SARS-CoV-2_circ_Homo_sapiens_893 | MN908947.3 | 8640 | 15724 | - | ORF1ab | 12 | CIRI2 | SRR11550043 |
| SARS-CoV-2_circ_Homo_sapiens_909 | MN908947.3 | 8773 | 10043 | - | ORF1ab | 12 | find_circ | SRR11550045 |
| SARS-CoV-2_circ_Homo_sapiens_1351 | MN908947.3 | 12153 | 13385 | + | ORF1ab | 11 | find_circ | SRR11550045 |
| SARS-CoV-2_circ_Homo_sapiens_1379 | MN908947.3 | 12266 | 18820 | + | ORF1ab | 11 | find_circ | SRR11550045 |
| SARS-CoV-2_circ_Homo_sapiens_1690 | MN908947.3 | 15333 | 15593 | + | ORF1ab | 11 | circRNA_finder | SRR11550046 |
| SARS-CoV-2_circ_Homo_sapiens_1690 | MN908947.3 | 15333 | 15593 | + | ORF1ab | 11 | find_circ | SRR11550046 |
| SARS-CoV-2_circ_Homo_sapiens_1729 | MN908947.3 | 15721 | 18355 | - | ORF1ab | 11 | CIRI2 | SRR11550046 |
| SARS-CoV-2_circ_Homo_sapiens_1829 | MN908947.3 | 17197 | 18214 | + | ORF1ab | 11 | find_circ | SRR11550043 |
| SARS-CoV-2_circ_Homo_sapiens_1854 | MN908947.3 | 17411 | 28146 | - | ORF6;M;E;ORF3a;S;ORF8;ORF7a;ORF7b;ORF1ab | 11 | CIRI2 | SRR11550046 |
| SARS-CoV-2_circ_Homo_sapiens_1948 | MN908947.3 | 18282 | 18661 | + | ORF1ab | 11 | find_circ | SRR11550044 |
| SARS-CoV-2_circ_Homo_sapiens_2412 | MN908947.3 | 23588 | 25428 | + | ORF3a;S | 11 | CIRI2 | SRR11550045 |
| SARS-CoV-2_circ_Homo_sapiens_2634 | MN908947.3 | 25514 | 26567 | - | ORF3a;M;E | 11 | find_circ | SRR11550046 |
| SARS-CoV-2_circ_Homo_sapiens_2634 | MN908947.3 | 25514 | 26567 | - | ORF3a;M;E | 11 | find_circ | SRR11550043 |
| SARS-CoV-2_circ_Homo_sapiens_2670 | MN908947.3 | 25672 | 26241 | - | ORF3a | 11 | find_circ | SRR11550046 |
| SARS-CoV-2_circ_Homo_sapiens_2687 | MN908947.3 | 25672 | 26606 | - | ORF3a;M;E | 11 | CIRI2 | SRR11550046 |
| SARS-CoV-2_circ_Homo_sapiens_2981 | MN908947.3 | 27799 | 28403 | + | ORF8;ORF7b;N | 11 | find_circ | SRR11550045 |
| SARS-CoV-2_circ_Homo_sapiens_3082 | MN908947.3 | 28410 | 29012 | - | N | 11 | find_circ | SRR11550046 |
| SARS-CoV-2_circ_Homo_sapiens_3142 | MN908947.3 | 28463 | 28960 | - | N | 11 | CIRI2 | SRR11550043 |
| SARS-CoV-2_circ_Homo_sapiens_3142 | MN908947.3 | 28463 | 28960 | - | N | 11 | CIRI2 | SRR11550044 |
| SARS-CoV-2_circ_Homo_sapiens_3152 | MN908947.3 | 28463 | 29090 | - | N | 11 | CIRI2 | SRR11550044 |
| SARS-CoV-2_circ_Homo_sapiens_3216 | MN908947.3 | 28610 | 29066 | - | N | 11 | CIRI2 | SRR11550046 |
| SARS-CoV-2_circ_Homo_sapiens_3223 | MN908947.3 | 28652 | 29012 | - | N | 11 | find_circ | SRR11550043 |
| SARS-CoV-2_circ_Homo_sapiens_3313 | MN908947.3 | 29086 | 29450 | - | N | 11 | find_circ | SRR11550045 |
| SARS-CoV-2_circ_Homo_sapiens_363 | MN908947.3 | 2571 | 3263 | - | ORF1ab | 11 | CIRI2 | SRR11550044 |
| SARS-CoV-2_circ_Homo_sapiens_573 | MN908947.3 | 4597 | 5090 | - | ORF1ab | 11 | CIRI2 | SRR11550046 |
| SARS-CoV-2_circ_Homo_sapiens_621 | MN908947.3 | 4921 | 5525 | - | ORF1ab | 11 | CIRI2 | SRR11550045 |
| SARS-CoV-2_circ_Homo_sapiens_667 | MN908947.3 | 5130 | 29375 | - | ORF6;M;E;ORF3a;S;N;ORF8;ORF7a;ORF7b;ORF1ab | 11 | CIRI2 | SRR11550046 |
| SARS-CoV-2_circ_Homo_sapiens_753 | MN908947.3 | 5827 | 8921 | - | ORF1ab | 11 | CIRI2 | SRR11550043 |
| SARS-CoV-2_circ_Homo_sapiens_1167 | MN908947.3 | 11190 | 12084 | - | ORF1ab | 10 | CIRI2 | SRR11550044 |
| SARS-CoV-2_circ_Homo_sapiens_1255 | MN908947.3 | 11705 | 12084 | - | ORF1ab | 10 | find_circ | SRR11550045 |
| SARS-CoV-2_circ_Homo_sapiens_1579 | MN908947.3 | 14178 | 14575 | - | ORF1ab | 10 | find_circ | SRR11550046 |
| SARS-CoV-2_circ_Homo_sapiens_1681 | MN908947.3 | 15280 | 16307 | - | ORF1ab | 10 | CIRI2 | SRR11550043 |
| SARS-CoV-2_circ_Homo_sapiens_1839 | MN908947.3 | 17406 | 18016 | + | ORF1ab | 10 | find_circ | SRR11550046 |
| SARS-CoV-2_circ_Homo_sapiens_2050 | MN908947.3 | 19603 | 19982 | - | ORF1ab | 10 | find_circ | SRR11550046 |
| SARS-CoV-2_circ_Homo_sapiens_2692 | MN908947.3 | 25672 | 26780 | - | ORF3a;M;E | 10 | CIRI2 | SRR11550045 |
| SARS-CoV-2_circ_Homo_sapiens_2893 | MN908947.3 | 27264 | 27558 | - | ORF7a;ORF6 | 10 | find_circ | SRR11550044 |
| SARS-CoV-2_circ_Homo_sapiens_3027 | MN908947.3 | 28321 | 29066 | - | N | 10 | find_circ | SRR11550045 |
| SARS-CoV-2_circ_Homo_sapiens_3060 | MN908947.3 | 28404 | 29262 | + | N | 10 | CIRI2 | SRR11550046 |
| SARS-CoV-2_circ_Homo_sapiens_3086 | MN908947.3 | 28410 | 29268 | - | N | 10 | CIRI2 | SRR11550043 |
| SARS-CoV-2_circ_Homo_sapiens_3096 | MN908947.3 | 28420 | 28609 | - | N | 10 | find_circ | SRR11550043 |
| SARS-CoV-2_circ_Homo_sapiens_3120 | MN908947.3 | 28463 | 28727 | - | N | 10 | find_circ | SRR11550045 |
| SARS-CoV-2_circ_Homo_sapiens_3142 | MN908947.3 | 28463 | 28960 | - | N | 10 | CIRI2 | SRR11550045 |
| SARS-CoV-2_circ_Homo_sapiens_3237 | MN908947.3 | 28696 | 28904 | - | N | 10 | find_circ | SRR11550043 |
| SARS-CoV-2_circ_Homo_sapiens_3282 | MN908947.3 | 28949 | 29268 | - | N | 10 | CIRI2 | SRR11550045 |
| SARS-CoV-2_circ_Homo_sapiens_3311 | MN908947.3 | 29086 | 29408 | + | N | 10 | circRNA_finder | SRR11550043 |
| SARS-CoV-2_circ_Homo_sapiens_3317 | MN908947.3 | 29086 | 29534 | - | N | 10 | find_circ | SRR11550043 |
| SARS-CoV-2_circ_Homo_sapiens_370 | MN908947.3 | 2571 | 3542 | - | ORF1ab | 10 | find_circ | SRR11550046 |
| SARS-CoV-2_circ_Homo_sapiens_6 | MN908947.3 | 8 | 514 | + | ORF1ab | 10 | CIRI2 | SRR11550046 |
| SARS-CoV-2_circ_Homo_sapiens_950 | MN908947.3 | 9123 | 12052 | - | ORF1ab | 10 | find_circ | SRR11550045 |
| SARS-CoV-2_circ_Homo_sapiens_1200 | MN908947.3 | 11488 | 14177 | - | ORF1ab | 9 | CIRI2 | SRR11550045 |
| SARS-CoV-2_circ_Homo_sapiens_1207 | MN908947.3 | 11488 | 18355 | - | ORF1ab | 9 | CIRI2 | SRR11550043 |
| SARS-CoV-2_circ_Homo_sapiens_1207 | MN908947.3 | 11488 | 18355 | - | ORF1ab | 9 | CIRI2 | SRR11550045 |
| SARS-CoV-2_circ_Homo_sapiens_1459 | MN908947.3 | 12974 | 14575 | - | ORF1ab | 9 | CIRI2 | SRR11550044 |
| SARS-CoV-2_circ_Homo_sapiens_1510 | MN908947.3 | 13398 | 15242 | + | ORF1ab | 9 | CIRI2 | SRR11550046 |
| SARS-CoV-2_circ_Homo_sapiens_1548 | MN908947.3 | 13736 | 14177 | - | ORF1ab | 9 | CIRI2 | SRR11550046 |
| SARS-CoV-2_circ_Homo_sapiens_1584 | MN908947.3 | 14178 | 14598 | - | ORF1ab | 9 | find_circ | SRR11550045 |
| SARS-CoV-2_circ_Homo_sapiens_1851 | MN908947.3 | 17411 | 18355 | - | ORF1ab | 9 | CIRI2 | SRR11550045 |
| SARS-CoV-2_circ_Homo_sapiens_1922 | MN908947.3 | 18021 | 18744 | + | ORF1ab | 9 | circRNA_finder | SRR11550046 |
| SARS-CoV-2_circ_Homo_sapiens_1922 | MN908947.3 | 18021 | 18744 | + | ORF1ab | 9 | find_circ | SRR11550046 |
| SARS-CoV-2_circ_Homo_sapiens_204 | MN908947.3 | 897 | 1244 | + | ORF1ab | 9 | find_circ | SRR11550043 |
| SARS-CoV-2_circ_Homo_sapiens_2670 | MN908947.3 | 25672 | 26241 | - | ORF3a | 9 | find_circ | SRR11550045 |
| SARS-CoV-2_circ_Homo_sapiens_2676 | MN908947.3 | 25672 | 26343 | - | ORF3a;E | 9 | CIRI2 | SRR11550043 |
| SARS-CoV-2_circ_Homo_sapiens_2730 | MN908947.3 | 25758 | 26276 | + | ORF3a;E | 9 | find_circ | SRR11550044 |
| SARS-CoV-2_circ_Homo_sapiens_3105 | MN908947.3 | 28435 | 28976 | - | N | 9 | find_circ | SRR11550046 |
| SARS-CoV-2_circ_Homo_sapiens_3121 | MN908947.3 | 28463 | 28727 | + | N | 9 | circRNA_finder | SRR11550046 |
| SARS-CoV-2_circ_Homo_sapiens_3147 | MN908947.3 | 28463 | 29012 | + | N | 9 | circRNA_finder | SRR11550044 |
| SARS-CoV-2_circ_Homo_sapiens_3192 | MN908947.3 | 28607 | 28904 | - | N | 9 | find_circ | SRR11550043 |
| SARS-CoV-2_circ_Homo_sapiens_3205 | MN908947.3 | 28610 | 28895 | - | N | 9 | find_circ | SRR11550045 |
| SARS-CoV-2_circ_Homo_sapiens_3225 | MN908947.3 | 28652 | 29066 | - | N | 9 | find_circ | SRR11550046 |
| SARS-CoV-2_circ_Homo_sapiens_3308 | MN908947.3 | 29086 | 29268 | - | N | 9 | CIRI2 | SRR11550045 |
| SARS-CoV-2_circ_Homo_sapiens_3313 | MN908947.3 | 29086 | 29450 | - | N | 9 | find_circ | SRR11550044 |
| SARS-CoV-2_circ_Homo_sapiens_3317 | MN908947.3 | 29086 | 29534 | - | N | 9 | find_circ | SRR11550046 |
| SARS-CoV-2_circ_Homo_sapiens_3318 | MN908947.3 | 29086 | 29534 | + | N | 9 | circRNA_finder | SRR11550045 |
| SARS-CoV-2_circ_Homo_sapiens_3318 | MN908947.3 | 29086 | 29534 | + | N | 9 | circRNA_finder | SRR11550043 |
| SARS-CoV-2_circ_Homo_sapiens_3320 | MN908947.3 | 29086 | 29665 | - | ORF10;N | 9 | find_circ | SRR11550045 |
| SARS-CoV-2_circ_Homo_sapiens_3320 | MN908947.3 | 29086 | 29665 | - | ORF10;N | 9 | find_circ | SRR11550043 |
| SARS-CoV-2_circ_Homo_sapiens_3393 | MN908947.3 | 29269 | 29529 | - | N | 9 | CIRI2 | SRR11550046 |
| SARS-CoV-2_circ_Homo_sapiens_38 | MN908947.3 | 16 | 449 | - | ORF1ab | 9 | find_circ | SRR11550046 |
| SARS-CoV-2_circ_Homo_sapiens_573 | MN908947.3 | 4597 | 5090 | - | ORF1ab | 9 | CIRI2 | SRR11550045 |
| SARS-CoV-2_circ_Homo_sapiens_66 | MN908947.3 | 24 | 29644 | + | ORF6;ORF10;M;E;ORF3a;S;N;ORF8;ORF7a;ORF7b;ORF1ab | 9 | CIRI2 | SRR11550043 |
| SARS-CoV-2_circ_Homo_sapiens_719 | MN908947.3 | 5622 | 26241 | - | ORF3a;S;ORF1ab | 9 | CIRI2 | SRR11550043 |
| SARS-CoV-2_circ_Homo_sapiens_745 | MN908947.3 | 5827 | 6379 | - | ORF1ab | 9 | find_circ | SRR11550046 |
| SARS-CoV-2_circ_Homo_sapiens_745 | MN908947.3 | 5827 | 6379 | - | ORF1ab | 9 | find_circ | SRR11550045 |
| SARS-CoV-2_circ_Homo_sapiens_826 | MN908947.3 | 7125 | 10009 | - | ORF1ab | 9 | CIRI2 | SRR11550046 |
| SARS-CoV-2_circ_Homo_sapiens_840 | MN908947.3 | 7859 | 9591 | - | ORF1ab | 9 | find_circ | SRR11550045 |
| SARS-CoV-2_circ_Homo_sapiens_855 | MN908947.3 | 8293 | 8654 | - | ORF1ab | 9 | CIRI2 | SRR11550043 |
| SARS-CoV-2_circ_Homo_sapiens_1230 | MN908947.3 | 11645 | 11966 | - | ORF1ab | 8 | find_circ | SRR11550046 |
| SARS-CoV-2_circ_Homo_sapiens_1231 | MN908947.3 | 11645 | 11966 | + | ORF1ab | 8 | circRNA_finder | SRR11550046 |
| SARS-CoV-2_circ_Homo_sapiens_1579 | MN908947.3 | 14178 | 14575 | - | ORF1ab | 8 | find_circ | SRR11550045 |
| SARS-CoV-2_circ_Homo_sapiens_1580 | MN908947.3 | 14178 | 14575 | + | ORF1ab | 8 | circRNA_finder | SRR11550046 |
| SARS-CoV-2_circ_Homo_sapiens_1580 | MN908947.3 | 14178 | 14575 | + | ORF1ab | 8 | circRNA_finder | SRR11550045 |
| SARS-CoV-2_circ_Homo_sapiens_1585 | MN908947.3 | 14178 | 14598 | + | ORF1ab | 8 | circRNA_finder | SRR11550045 |
| SARS-CoV-2_circ_Homo_sapiens_18 | MN908947.3 | 8 | 6960 | + | ORF1ab | 8 | find_circ | SRR11550046 |
| SARS-CoV-2_circ_Homo_sapiens_1901 | MN908947.3 | 18017 | 18876 | - | ORF1ab | 8 | find_circ | SRR11550043 |
| SARS-CoV-2_circ_Homo_sapiens_1960 | MN908947.3 | 18356 | 21653 | - | S;ORF1ab | 8 | CIRI2 | SRR11550044 |
| SARS-CoV-2_circ_Homo_sapiens_2056 | MN908947.3 | 19605 | 20103 | - | ORF1ab | 8 | find_circ | SRR11550045 |
| SARS-CoV-2_circ_Homo_sapiens_2302 | MN908947.3 | 22998 | 25516 | - | ORF3a;S | 8 | CIRI2 | SRR11550044 |
| SARS-CoV-2_circ_Homo_sapiens_2397 | MN908947.3 | 23568 | 27187 | + | ORF3a;S;M;E | 8 | CIRI2 | SRR11550044 |
| SARS-CoV-2_circ_Homo_sapiens_2411 | MN908947.3 | 23588 | 24200 | + | S | 8 | find_circ | SRR11550046 |
| SARS-CoV-2_circ_Homo_sapiens_2573 | MN908947.3 | 24990 | 27595 | - | ORF6;M;E;ORF3a;S;ORF7a | 8 | CIRI2 | SRR11550045 |
| SARS-CoV-2_circ_Homo_sapiens_2671 | MN908947.3 | 25672 | 26241 | + | ORF3a | 8 | circRNA_finder | SRR11550045 |
| SARS-CoV-2_circ_Homo_sapiens_2671 | MN908947.3 | 25672 | 26241 | + | ORF3a | 8 | circRNA_finder | SRR11550046 |
| SARS-CoV-2_circ_Homo_sapiens_2685 | MN908947.3 | 25672 | 26570 | - | ORF3a;M;E | 8 | find_circ | SRR11550045 |
| SARS-CoV-2_circ_Homo_sapiens_2690 | MN908947.3 | 25672 | 26775 | - | ORF3a;M;E | 8 | CIRI2 | SRR11550045 |
| SARS-CoV-2_circ_Homo_sapiens_2690 | MN908947.3 | 25672 | 26775 | - | ORF3a;M;E | 8 | CIRI2 | SRR11550046 |
| SARS-CoV-2_circ_Homo_sapiens_2730 | MN908947.3 | 25758 | 26276 | + | ORF3a;E | 8 | find_circ | SRR11550046 |
| SARS-CoV-2_circ_Homo_sapiens_2752 | MN908947.3 | 26156 | 26778 | + | ORF3a;M;E | 8 | find_circ | SRR11550045 |
| SARS-CoV-2_circ_Homo_sapiens_2795 | MN908947.3 | 26825 | 27225 | + | ORF6;M | 8 | circRNA_finder | SRR11550044 |
| SARS-CoV-2_circ_Homo_sapiens_2981 | MN908947.3 | 27799 | 28403 | + | ORF8;ORF7b;N | 8 | find_circ | SRR11550046 |
| SARS-CoV-2_circ_Homo_sapiens_3058 | MN908947.3 | 28404 | 29080 | + | N | 8 | find_circ | SRR11550046 |
| SARS-CoV-2_circ_Homo_sapiens_3058 | MN908947.3 | 28404 | 29080 | + | N | 8 | circRNA_finder | SRR11550046 |
| SARS-CoV-2_circ_Homo_sapiens_3086 | MN908947.3 | 28410 | 29268 | - | N | 8 | CIRI2 | SRR11550046 |
| SARS-CoV-2_circ_Homo_sapiens_3118 | MN908947.3 | 28463 | 28686 | - | N | 8 | CIRI2 | SRR11550045 |
| SARS-CoV-2_circ_Homo_sapiens_3152 | MN908947.3 | 28463 | 29090 | - | N | 8 | find_circ | SRR11550044 |
| SARS-CoV-2_circ_Homo_sapiens_3153 | MN908947.3 | 28463 | 29090 | + | N | 8 | circRNA_finder | SRR11550044 |
| SARS-CoV-2_circ_Homo_sapiens_3205 | MN908947.3 | 28610 | 28895 | - | N | 8 | CIRI2 | SRR11550043 |
| SARS-CoV-2_circ_Homo_sapiens_3205 | MN908947.3 | 28610 | 28895 | - | N | 8 | find_circ | SRR11550043 |
| SARS-CoV-2_circ_Homo_sapiens_3252 | MN908947.3 | 28709 | 29066 | - | N | 8 | find_circ | SRR11550043 |
| SARS-CoV-2_circ_Homo_sapiens_3362 | MN908947.3 | 29190 | 29579 | - | ORF10;N | 8 | find_circ | SRR11550043 |
| SARS-CoV-2_circ_Homo_sapiens_363 | MN908947.3 | 2571 | 3263 | - | ORF1ab | 8 | CIRI2 | SRR11550043 |
| SARS-CoV-2_circ_Homo_sapiens_37 | MN908947.3 | 16 | 320 | - | ORF1ab | 8 | find_circ | SRR11550045 |
| SARS-CoV-2_circ_Homo_sapiens_38 | MN908947.3 | 16 | 449 | - | ORF1ab | 8 | find_circ | SRR11550045 |
| SARS-CoV-2_circ_Homo_sapiens_397 | MN908947.3 | 2799 | 3542 | - | ORF1ab | 8 | find_circ | SRR11550045 |
| SARS-CoV-2_circ_Homo_sapiens_5 | MN908947.3 | 8 | 442 | + | ORF1ab | 8 | find_circ | SRR11550045 |
| SARS-CoV-2_circ_Homo_sapiens_553 | MN908947.3 | 4535 | 7278 | - | ORF1ab | 8 | find_circ | SRR11550043 |
| SARS-CoV-2_circ_Homo_sapiens_6 | MN908947.3 | 8 | 514 | + | ORF1ab | 8 | find_circ | SRR11550045 |
| SARS-CoV-2_circ_Homo_sapiens_657 | MN908947.3 | 5130 | 5525 | - | ORF1ab | 8 | find_circ | SRR11550045 |
| SARS-CoV-2_circ_Homo_sapiens_758 | MN908947.3 | 5904 | 28596 | + | ORF6;M;E;ORF3a;S;N;ORF8;ORF7a;ORF7b;ORF1ab | 8 | find_circ | SRR11550046 |
| SARS-CoV-2_circ_Homo_sapiens_809 | MN908947.3 | 6681 | 7621 | + | ORF1ab | 8 | find_circ | SRR11550045 |
| SARS-CoV-2_circ_Homo_sapiens_1012 | MN908947.3 | 10016 | 10349 | - | ORF1ab | 7 | find_circ | SRR11550044 |
| SARS-CoV-2_circ_Homo_sapiens_1013 | MN908947.3 | 10016 | 10349 | + | ORF1ab | 7 | circRNA_finder | SRR11550044 |
| SARS-CoV-2_circ_Homo_sapiens_1117 | MN908947.3 | 10659 | 11872 | + | ORF1ab | 7 | find_circ | SRR11550045 |
| SARS-CoV-2_circ_Homo_sapiens_1256 | MN908947.3 | 11705 | 12131 | - | ORF1ab | 7 | find_circ | SRR11550045 |
| SARS-CoV-2_circ_Homo_sapiens_1333 | MN908947.3 | 12132 | 13385 | + | ORF1ab | 7 | find_circ | SRR11550045 |
| SARS-CoV-2_circ_Homo_sapiens_1343 | MN908947.3 | 12153 | 12599 | + | ORF1ab | 7 | find_circ | SRR11550046 |
| SARS-CoV-2_circ_Homo_sapiens_1640 | MN908947.3 | 14689 | 15112 | - | ORF1ab | 7 | find_circ | SRR11550044 |
| SARS-CoV-2_circ_Homo_sapiens_1641 | MN908947.3 | 14689 | 15112 | + | ORF1ab | 7 | circRNA_finder | SRR11550044 |
| SARS-CoV-2_circ_Homo_sapiens_1681 | MN908947.3 | 15280 | 16307 | - | ORF1ab | 7 | CIRI2 | SRR11550045 |
| SARS-CoV-2_circ_Homo_sapiens_1825 | MN908947.3 | 17003 | 18421 | - | ORF1ab | 7 | find_circ | SRR11550043 |
| SARS-CoV-2_circ_Homo_sapiens_1901 | MN908947.3 | 18017 | 18876 | - | ORF1ab | 7 | find_circ | SRR11550046 |
| SARS-CoV-2_circ_Homo_sapiens_1902 | MN908947.3 | 18017 | 18876 | + | ORF1ab | 7 | circRNA_finder | SRR11550043 |
| SARS-CoV-2_circ_Homo_sapiens_1918 | MN908947.3 | 18021 | 18379 | + | ORF1ab | 7 | CIRI2 | SRR11550045 |
| SARS-CoV-2_circ_Homo_sapiens_1960 | MN908947.3 | 18356 | 21653 | - | S;ORF1ab | 7 | find_circ | SRR11550044 |
| SARS-CoV-2_circ_Homo_sapiens_1961 | MN908947.3 | 18356 | 21653 | + | S;ORF1ab | 7 | circRNA_finder | SRR11550044 |
| SARS-CoV-2_circ_Homo_sapiens_1975 | MN908947.3 | 18407 | 18897 | + | ORF1ab | 7 | CIRI2 | SRR11550043 |
| SARS-CoV-2_circ_Homo_sapiens_2034 | MN908947.3 | 19007 | 19347 | + | ORF1ab | 7 | find_circ | SRR11550045 |
| SARS-CoV-2_circ_Homo_sapiens_2053 | MN908947.3 | 19605 | 19982 | - | ORF1ab | 7 | find_circ | SRR11550045 |
| SARS-CoV-2_circ_Homo_sapiens_26 | MN908947.3 | 8 | 23350 | + | S;ORF1ab | 7 | CIRI2 | SRR11550044 |
| SARS-CoV-2_circ_Homo_sapiens_2634 | MN908947.3 | 25514 | 26567 | - | ORF3a;M;E | 7 | find_circ | SRR11550045 |
| SARS-CoV-2_circ_Homo_sapiens_2672 | MN908947.3 | 25672 | 26298 | - | ORF3a;E | 7 | CIRI2 | SRR11550045 |
| SARS-CoV-2_circ_Homo_sapiens_2690 | MN908947.3 | 25672 | 26775 | - | ORF3a;M;E | 7 | CIRI2 | SRR11550044 |
| SARS-CoV-2_circ_Homo_sapiens_2707 | MN908947.3 | 25672 | 27051 | - | ORF3a;M;E | 7 | CIRI2 | SRR11550045 |
| SARS-CoV-2_circ_Homo_sapiens_2836 | MN908947.3 | 27060 | 27583 | - | ORF7a;ORF6;M | 7 | find_circ | SRR11550045 |
| SARS-CoV-2_circ_Homo_sapiens_2933 | MN908947.3 | 27576 | 28092 | - | ORF7a;ORF8;ORF7b | 7 | find_circ | SRR11550046 |
| SARS-CoV-2_circ_Homo_sapiens_2966 | MN908947.3 | 27726 | 28609 | - | ORF7a;ORF8;ORF7b;N | 7 | find_circ | SRR11550045 |
| SARS-CoV-2_circ_Homo_sapiens_2999 | MN908947.3 | 28147 | 28462 | - | ORF8;N | 7 | CIRI2 | SRR11550045 |
| SARS-CoV-2_circ_Homo_sapiens_3026 | MN908947.3 | 28321 | 29012 | - | N | 7 | find_circ | SRR11550043 |
| SARS-CoV-2_circ_Homo_sapiens_3027 | MN908947.3 | 28321 | 29066 | - | N | 7 | find_circ | SRR11550046 |
| SARS-CoV-2_circ_Homo_sapiens_3028 | MN908947.3 | 28321 | 29268 | - | N | 7 | CIRI2 | SRR11550045 |
| SARS-CoV-2_circ_Homo_sapiens_3051 | MN908947.3 | 28404 | 28850 | + | N | 7 | find_circ | SRR11550044 |
| SARS-CoV-2_circ_Homo_sapiens_3055 | MN908947.3 | 28404 | 28979 | + | N | 7 | find_circ | SRR11550046 |
| SARS-CoV-2_circ_Homo_sapiens_3058 | MN908947.3 | 28404 | 29080 | + | N | 7 | find_circ | SRR11550044 |
| SARS-CoV-2_circ_Homo_sapiens_3096 | MN908947.3 | 28420 | 28609 | - | N | 7 | find_circ | SRR11550044 |
| SARS-CoV-2_circ_Homo_sapiens_3146 | MN908947.3 | 28463 | 29012 | - | N | 7 | find_circ | SRR11550043 |
| SARS-CoV-2_circ_Homo_sapiens_3158 | MN908947.3 | 28463 | 29415 | - | N | 7 | CIRI2 | SRR11550044 |
| SARS-CoV-2_circ_Homo_sapiens_3168 | MN908947.3 | 28463 | 29678 | - | ORF10;N | 7 | CIRI2 | SRR11550046 |
| SARS-CoV-2_circ_Homo_sapiens_3206 | MN908947.3 | 28610 | 28895 | + | N | 7 | circRNA_finder | SRR11550045 |
| SARS-CoV-2_circ_Homo_sapiens_3212 | MN908947.3 | 28610 | 29012 | - | N | 7 | CIRI2 | SRR11550043 |
| SARS-CoV-2_circ_Homo_sapiens_3212 | MN908947.3 | 28610 | 29012 | - | N | 7 | find_circ | SRR11550045 |
| SARS-CoV-2_circ_Homo_sapiens_3212 | MN908947.3 | 28610 | 29012 | - | N | 7 | CIRI2 | SRR11550046 |
| SARS-CoV-2_circ_Homo_sapiens_3237 | MN908947.3 | 28696 | 28904 | - | N | 7 | find_circ | SRR11550044 |
| SARS-CoV-2_circ_Homo_sapiens_3318 | MN908947.3 | 29086 | 29534 | + | N | 7 | circRNA_finder | SRR11550046 |
| SARS-CoV-2_circ_Homo_sapiens_3332 | MN908947.3 | 29124 | 29320 | + | N | 7 | find_circ | SRR11550046 |
| SARS-CoV-2_circ_Homo_sapiens_3348 | MN908947.3 | 29172 | 29529 | - | N | 7 | find_circ | SRR11550046 |
| SARS-CoV-2_circ_Homo_sapiens_3350 | MN908947.3 | 29172 | 29579 | - | ORF10;N | 7 | find_circ | SRR11550046 |
| SARS-CoV-2_circ_Homo_sapiens_3362 | MN908947.3 | 29190 | 29579 | - | ORF10;N | 7 | find_circ | SRR11550044 |
| SARS-CoV-2_circ_Homo_sapiens_38 | MN908947.3 | 16 | 449 | - | ORF1ab | 7 | find_circ | SRR11550043 |
| SARS-CoV-2_circ_Homo_sapiens_43 | MN908947.3 | 16 | 2700 | - | ORF1ab | 7 | find_circ | SRR11550046 |
| SARS-CoV-2_circ_Homo_sapiens_658 | MN908947.3 | 5130 | 5525 | + | ORF1ab | 7 | circRNA_finder | SRR11550045 |
| SARS-CoV-2_circ_Homo_sapiens_746 | MN908947.3 | 5827 | 6379 | + | ORF1ab | 7 | circRNA_finder | SRR11550045 |
| SARS-CoV-2_circ_Homo_sapiens_856 | MN908947.3 | 8293 | 8654 | + | ORF1ab | 7 | circRNA_finder | SRR11550043 |
| SARS-CoV-2_circ_Homo_sapiens_918 | MN908947.3 | 8782 | 24276 | + | S;ORF1ab | 7 | CIRI2 | SRR11550046 |
| SARS-CoV-2_circ_Homo_sapiens_1026 | MN908947.3 | 10044 | 10499 | + | ORF1ab | 6 | CIRI2 | SRR11550045 |
| SARS-CoV-2_circ_Homo_sapiens_1029 | MN908947.3 | 10055 | 10495 | + | ORF1ab | 6 | find_circ | SRR11550046 |
| SARS-CoV-2_circ_Homo_sapiens_1031 | MN908947.3 | 10066 | 10499 | + | ORF1ab | 6 | CIRI2 | SRR11550046 |
| SARS-CoV-2_circ_Homo_sapiens_107 | MN908947.3 | 312 | 682 | - | ORF1ab | 6 | find_circ | SRR11550046 |
| SARS-CoV-2_circ_Homo_sapiens_107 | MN908947.3 | 312 | 682 | - | ORF1ab | 6 | find_circ | SRR11550045 |
| SARS-CoV-2_circ_Homo_sapiens_1252 | MN908947.3 | 11705 | 12004 | - | ORF1ab | 6 | find_circ | SRR11550046 |
| SARS-CoV-2_circ_Homo_sapiens_1255 | MN908947.3 | 11705 | 12084 | - | ORF1ab | 6 | find_circ | SRR11550046 |
| SARS-CoV-2_circ_Homo_sapiens_1368 | MN908947.3 | 12203 | 14862 | + | ORF1ab | 6 | find_circ | SRR11550046 |
| SARS-CoV-2_circ_Homo_sapiens_151 | MN908947.3 | 689 | 1244 | + | ORF1ab | 6 | find_circ | SRR11550046 |
| SARS-CoV-2_circ_Homo_sapiens_151 | MN908947.3 | 689 | 1244 | + | ORF1ab | 6 | find_circ | SRR11550043 |
| SARS-CoV-2_circ_Homo_sapiens_151 | MN908947.3 | 689 | 1244 | + | ORF1ab | 6 | find_circ | SRR11550045 |
| SARS-CoV-2_circ_Homo_sapiens_1534 | MN908947.3 | 13518 | 14572 | - | ORF1ab | 6 | find_circ | SRR11550045 |
| SARS-CoV-2_circ_Homo_sapiens_1577 | MN908947.3 | 14178 | 14572 | - | ORF1ab | 6 | find_circ | SRR11550045 |
| SARS-CoV-2_circ_Homo_sapiens_1578 | MN908947.3 | 14178 | 14572 | + | ORF1ab | 6 | circRNA_finder | SRR11550045 |
| SARS-CoV-2_circ_Homo_sapiens_1821 | MN908947.3 | 16949 | 29036 | - | ORF6;M;E;ORF3a;S;N;ORF8;ORF7a;ORF7b;ORF1ab | 6 | CIRI2 | SRR11550045 |
| SARS-CoV-2_circ_Homo_sapiens_1839 | MN908947.3 | 17406 | 18016 | + | ORF1ab | 6 | find_circ | SRR11550043 |
| SARS-CoV-2_circ_Homo_sapiens_1918 | MN908947.3 | 18021 | 18379 | + | ORF1ab | 6 | find_circ | SRR11550045 |
| SARS-CoV-2_circ_Homo_sapiens_1927 | MN908947.3 | 18021 | 19299 | + | ORF1ab | 6 | find_circ | SRR11550045 |
| SARS-CoV-2_circ_Homo_sapiens_204 | MN908947.3 | 897 | 1244 | + | ORF1ab | 6 | find_circ | SRR11550045 |
| SARS-CoV-2_circ_Homo_sapiens_205 | MN908947.3 | 897 | 1337 | + | ORF1ab | 6 | find_circ | SRR11550046 |
| SARS-CoV-2_circ_Homo_sapiens_2054 | MN908947.3 | 19605 | 19982 | + | ORF1ab | 6 | circRNA_finder | SRR11550045 |
| SARS-CoV-2_circ_Homo_sapiens_2058 | MN908947.3 | 19605 | 20198 | - | ORF1ab | 6 | find_circ | SRR11550043 |
| SARS-CoV-2_circ_Homo_sapiens_2058 | MN908947.3 | 19605 | 20198 | - | ORF1ab | 6 | find_circ | SRR11550046 |
| SARS-CoV-2_circ_Homo_sapiens_2058 | MN908947.3 | 19605 | 20198 | - | ORF1ab | 6 | find_circ | SRR11550045 |
| SARS-CoV-2_circ_Homo_sapiens_2059 | MN908947.3 | 19605 | 20198 | + | ORF1ab | 6 | circRNA_finder | SRR11550043 |
| SARS-CoV-2_circ_Homo_sapiens_2063 | MN908947.3 | 19611 | 20015 | - | ORF1ab | 6 | find_circ | SRR11550046 |
| SARS-CoV-2_circ_Homo_sapiens_2065 | MN908947.3 | 19611 | 20203 | - | ORF1ab | 6 | find_circ | SRR11550044 |
| SARS-CoV-2_circ_Homo_sapiens_2065 | MN908947.3 | 19611 | 20203 | - | ORF1ab | 6 | find_circ | SRR11550046 |
| SARS-CoV-2_circ_Homo_sapiens_2090 | MN908947.3 | 20366 | 20767 | - | ORF1ab | 6 | CIRI2 | SRR11550043 |
| SARS-CoV-2_circ_Homo_sapiens_2190 | MN908947.3 | 21768 | 23060 | - | S | 6 | find_circ | SRR11550045 |
| SARS-CoV-2_circ_Homo_sapiens_2333 | MN908947.3 | 23142 | 25602 | - | ORF3a;S | 6 | CIRI2 | SRR11550046 |
| SARS-CoV-2_circ_Homo_sapiens_2349 | MN908947.3 | 23307 | 24022 | - | S | 6 | find_circ | SRR11550043 |
| SARS-CoV-2_circ_Homo_sapiens_2370 | MN908947.3 | 23552 | 24457 | - | S | 6 | find_circ | SRR11550046 |
| SARS-CoV-2_circ_Homo_sapiens_2379 | MN908947.3 | 23568 | 24192 | + | S | 6 | find_circ | SRR11550043 |
| SARS-CoV-2_circ_Homo_sapiens_2379 | MN908947.3 | 23568 | 24192 | + | S | 6 | circRNA_finder | SRR11550043 |
| SARS-CoV-2_circ_Homo_sapiens_2386 | MN908947.3 | 23568 | 25428 | + | ORF3a;S | 6 | CIRI2 | SRR11550046 |
| SARS-CoV-2_circ_Homo_sapiens_2394 | MN908947.3 | 23568 | 26783 | + | ORF3a;S;M;E | 6 | CIRI2 | SRR11550044 |
| SARS-CoV-2_circ_Homo_sapiens_2397 | MN908947.3 | 23568 | 27187 | + | ORF3a;S;M;E | 6 | CIRI2 | SRR11550045 |
| SARS-CoV-2_circ_Homo_sapiens_242 | MN908947.3 | 1009 | 3517 | + | ORF1ab | 6 | find_circ | SRR11550044 |
| SARS-CoV-2_circ_Homo_sapiens_2672 | MN908947.3 | 25672 | 26298 | - | ORF3a;E | 6 | CIRI2 | SRR11550044 |
| SARS-CoV-2_circ_Homo_sapiens_2693 | MN908947.3 | 25672 | 26780 | + | ORF3a;M;E | 6 | circRNA_finder | SRR11550045 |
| SARS-CoV-2_circ_Homo_sapiens_2731 | MN908947.3 | 25770 | 26157 | + | ORF3a | 6 | find_circ | SRR11550045 |
| SARS-CoV-2_circ_Homo_sapiens_2747 | MN908947.3 | 25990 | 27011 | - | ORF3a;M;E | 6 | find_circ | SRR11550046 |
| SARS-CoV-2_circ_Homo_sapiens_2793 | MN908947.3 | 26788 | 27225 | + | ORF6;M | 6 | find_circ | SRR11550044 |
| SARS-CoV-2_circ_Homo_sapiens_2812 | MN908947.3 | 26874 | 27218 | - | ORF6;M | 6 | find_circ | SRR11550045 |
| SARS-CoV-2_circ_Homo_sapiens_2855 | MN908947.3 | 27144 | 27685 | - | ORF7a;ORF6;M | 6 | find_circ | SRR11550043 |
| SARS-CoV-2_circ_Homo_sapiens_2884 | MN908947.3 | 27226 | 29262 | + | ORF6;N;ORF8;ORF7a;ORF7b | 6 | CIRI2 | SRR11550044 |
| SARS-CoV-2_circ_Homo_sapiens_2998 | MN908947.3 | 28102 | 28472 | - | ORF8;N | 6 | find_circ | SRR11550043 |
| SARS-CoV-2_circ_Homo_sapiens_3041 | MN908947.3 | 28338 | 28979 | + | N | 6 | find_circ | SRR11550045 |
| SARS-CoV-2_circ_Homo_sapiens_3047 | MN908947.3 | 28404 | 28596 | + | N | 6 | find_circ | SRR11550044 |
| SARS-CoV-2_circ_Homo_sapiens_3055 | MN908947.3 | 28404 | 28979 | + | N | 6 | circRNA_finder | SRR11550046 |
| SARS-CoV-2_circ_Homo_sapiens_3060 | MN908947.3 | 28404 | 29262 | + | N | 6 | CIRI2 | SRR11550045 |
| SARS-CoV-2_circ_Homo_sapiens_3121 | MN908947.3 | 28463 | 28727 | + | N | 6 | circRNA_finder | SRR11550045 |
| SARS-CoV-2_circ_Homo_sapiens_3125 | MN908947.3 | 28463 | 28753 | - | N | 6 | CIRI2 | SRR11550044 |
| SARS-CoV-2_circ_Homo_sapiens_3132 | MN908947.3 | 28463 | 28886 | - | N | 6 | CIRI2 | SRR11550046 |
| SARS-CoV-2_circ_Homo_sapiens_3142 | MN908947.3 | 28463 | 28960 | - | N | 6 | CIRI2 | SRR11550046 |
| SARS-CoV-2_circ_Homo_sapiens_3150 | MN908947.3 | 28463 | 29066 | - | N | 6 | find_circ | SRR11550044 |
| SARS-CoV-2_circ_Homo_sapiens_32 | MN908947.3 | 8 | 28850 | + | ORF6;M;E;ORF3a;S;N;ORF8;ORF7a;ORF7b;ORF1ab | 6 | CIRI2 | SRR11550043 |
| SARS-CoV-2_circ_Homo_sapiens_3217 | MN908947.3 | 28610 | 29787 | - | ORF10;N | 6 | CIRI2 | SRR11550045 |
| SARS-CoV-2_circ_Homo_sapiens_3240 | MN908947.3 | 28696 | 29012 | - | N | 6 | find_circ | SRR11550044 |
| SARS-CoV-2_circ_Homo_sapiens_3249 | MN908947.3 | 28709 | 29012 | - | N | 6 | find_circ | SRR11550045 |
| SARS-CoV-2_circ_Homo_sapiens_3321 | MN908947.3 | 29086 | 29665 | + | ORF10;N | 6 | circRNA_finder | SRR11550045 |
| SARS-CoV-2_circ_Homo_sapiens_3348 | MN908947.3 | 29172 | 29529 | - | N | 6 | find_circ | SRR11550044 |
| SARS-CoV-2_circ_Homo_sapiens_3350 | MN908947.3 | 29172 | 29579 | - | ORF10;N | 6 | find_circ | SRR11550043 |
| SARS-CoV-2_circ_Homo_sapiens_3350 | MN908947.3 | 29172 | 29579 | - | ORF10;N | 6 | find_circ | SRR11550045 |
| SARS-CoV-2_circ_Homo_sapiens_3358 | MN908947.3 | 29172 | 29707 | - | ORF10;N | 6 | find_circ | SRR11550045 |
| SARS-CoV-2_circ_Homo_sapiens_3368 | MN908947.3 | 29250 | 29444 | - | N | 6 | find_circ | SRR11550046 |
| SARS-CoV-2_circ_Homo_sapiens_379 | MN908947.3 | 2592 | 3337 | + | ORF1ab | 6 | find_circ | SRR11550045 |
| SARS-CoV-2_circ_Homo_sapiens_417 | MN908947.3 | 3048 | 3517 | + | ORF1ab | 6 | find_circ | SRR11550044 |
| SARS-CoV-2_circ_Homo_sapiens_424 | MN908947.3 | 3066 | 29259 | + | ORF6;M;E;ORF3a;S;N;ORF8;ORF7a;ORF7b;ORF1ab | 6 | CIRI2 | SRR11550043 |
| SARS-CoV-2_circ_Homo_sapiens_5 | MN908947.3 | 8 | 442 | + | ORF1ab | 6 | find_circ | SRR11550046 |
| SARS-CoV-2_circ_Homo_sapiens_535 | MN908947.3 | 4328 | 4523 | - | ORF1ab | 6 | find_circ | SRR11550046 |
| SARS-CoV-2_circ_Homo_sapiens_57 | MN908947.3 | 16 | 28609 | - | ORF6;M;E;ORF3a;S;N;ORF8;ORF7a;ORF7b;ORF1ab | 6 | find_circ | SRR11550045 |
| SARS-CoV-2_circ_Homo_sapiens_590 | MN908947.3 | 4836 | 6562 | + | ORF1ab | 6 | CIRI2 | SRR11550046 |
| SARS-CoV-2_circ_Homo_sapiens_613 | MN908947.3 | 4901 | 29143 | - | ORF6;M;E;ORF3a;S;N;ORF8;ORF7a;ORF7b;ORF1ab | 6 | CIRI2 | SRR11550043 |
| SARS-CoV-2_circ_Homo_sapiens_670 | MN908947.3 | 5148 | 5821 | - | ORF1ab | 6 | find_circ | SRR11550046 |
| SARS-CoV-2_circ_Homo_sapiens_671 | MN908947.3 | 5148 | 5821 | + | ORF1ab | 6 | circRNA_finder | SRR11550046 |
| SARS-CoV-2_circ_Homo_sapiens_855 | MN908947.3 | 8293 | 8654 | - | ORF1ab | 6 | find_circ | SRR11550043 |
| SARS-CoV-2_circ_Homo_sapiens_880 | MN908947.3 | 8300 | 8921 | - | ORF1ab | 6 | CIRI2 | SRR11550045 |
| SARS-CoV-2_circ_Homo_sapiens_889 | MN908947.3 | 8345 | 29268 | - | ORF6;M;E;ORF3a;S;N;ORF8;ORF7a;ORF7b;ORF1ab | 6 | CIRI2 | SRR11550045 |
| SARS-CoV-2_circ_Homo_sapiens_902 | MN908947.3 | 8771 | 10038 | - | ORF1ab | 6 | find_circ | SRR11550043 |
| SARS-CoV-2_circ_Homo_sapiens_1025 | MN908947.3 | 10044 | 10493 | + | ORF1ab | 5 | circRNA_finder | SRR11550046 |
| SARS-CoV-2_circ_Homo_sapiens_1025 | MN908947.3 | 10044 | 10493 | + | ORF1ab | 5 | find_circ | SRR11550046 |
| SARS-CoV-2_circ_Homo_sapiens_1152 | MN908947.3 | 10948 | 11178 | + | ORF1ab | 5 | circRNA_finder | SRR11550046 |
| SARS-CoV-2_circ_Homo_sapiens_1152 | MN908947.3 | 10948 | 11178 | + | ORF1ab | 5 | find_circ | SRR11550046 |
| SARS-CoV-2_circ_Homo_sapiens_1167 | MN908947.3 | 11190 | 12084 | - | ORF1ab | 5 | find_circ | SRR11550044 |
| SARS-CoV-2_circ_Homo_sapiens_1168 | MN908947.3 | 11190 | 12084 | + | ORF1ab | 5 | circRNA_finder | SRR11550044 |
| SARS-CoV-2_circ_Homo_sapiens_1226 | MN908947.3 | 11638 | 12131 | - | ORF1ab | 5 | find_circ | SRR11550045 |
| SARS-CoV-2_circ_Homo_sapiens_132 | MN908947.3 | 527 | 1244 | + | ORF1ab | 5 | circRNA_finder | SRR11550044 |
| SARS-CoV-2_circ_Homo_sapiens_132 | MN908947.3 | 527 | 1244 | + | ORF1ab | 5 | find_circ | SRR11550044 |
| SARS-CoV-2_circ_Homo_sapiens_140 | MN908947.3 | 527 | 3353 | + | ORF1ab | 5 | circRNA_finder | SRR11550046 |
| SARS-CoV-2_circ_Homo_sapiens_140 | MN908947.3 | 527 | 3353 | + | ORF1ab | 5 | find_circ | SRR11550046 |
| SARS-CoV-2_circ_Homo_sapiens_1434 | MN908947.3 | 12831 | 18355 | - | ORF1ab | 5 | CIRI2 | SRR11550043 |
| SARS-CoV-2_circ_Homo_sapiens_1458 | MN908947.3 | 12974 | 14167 | - | ORF1ab | 5 | find_circ | SRR11550043 |
| SARS-CoV-2_circ_Homo_sapiens_1525 | MN908947.3 | 13500 | 18355 | - | ORF1ab | 5 | CIRI2 | SRR11550043 |
| SARS-CoV-2_circ_Homo_sapiens_1525 | MN908947.3 | 13500 | 18355 | - | ORF1ab | 5 | CIRI2 | SRR11550044 |
| SARS-CoV-2_circ_Homo_sapiens_154 | MN908947.3 | 689 | 1598 | + | ORF1ab | 5 | CIRI2 | SRR11550046 |
| SARS-CoV-2_circ_Homo_sapiens_1633 | MN908947.3 | 14609 | 15242 | + | ORF1ab | 5 | find_circ | SRR11550046 |
| SARS-CoV-2_circ_Homo_sapiens_1700 | MN908947.3 | 15333 | 23561 | + | S;ORF1ab | 5 | CIRI2 | SRR11550045 |
| SARS-CoV-2_circ_Homo_sapiens_1795 | MN908947.3 | 16630 | 16974 | + | ORF1ab | 5 | find_circ | SRR11550046 |
| SARS-CoV-2_circ_Homo_sapiens_1839 | MN908947.3 | 17406 | 18016 | + | ORF1ab | 5 | find_circ | SRR11550045 |
| SARS-CoV-2_circ_Homo_sapiens_1868 | MN908947.3 | 17717 | 25476 | + | ORF3a;S;ORF1ab | 5 | circRNA_finder | SRR11550043 |
| SARS-CoV-2_circ_Homo_sapiens_1868 | MN908947.3 | 17717 | 25476 | + | ORF3a;S;ORF1ab | 5 | find_circ | SRR11550043 |
| SARS-CoV-2_circ_Homo_sapiens_1922 | MN908947.3 | 18021 | 18744 | + | ORF1ab | 5 | find_circ | SRR11550045 |
| SARS-CoV-2_circ_Homo_sapiens_1922 | MN908947.3 | 18021 | 18744 | + | ORF1ab | 5 | circRNA_finder | SRR11550045 |
| SARS-CoV-2_circ_Homo_sapiens_1941 | MN908947.3 | 18176 | 18813 | - | ORF1ab | 5 | find_circ | SRR11550043 |
| SARS-CoV-2_circ_Homo_sapiens_1988 | MN908947.3 | 18494 | 29006 | - | ORF6;M;E;ORF3a;S;N;ORF8;ORF7a;ORF7b;ORF1ab | 5 | find_circ | SRR11550043 |
| SARS-CoV-2_circ_Homo_sapiens_1989 | MN908947.3 | 18494 | 29006 | + | ORF6;M;E;ORF3a;S;N;ORF8;ORF7a;ORF7b;ORF1ab | 5 | circRNA_finder | SRR11550043 |
| SARS-CoV-2_circ_Homo_sapiens_201 | MN908947.3 | 882 | 2392 | + | ORF1ab | 5 | find_circ | SRR11550043 |
| SARS-CoV-2_circ_Homo_sapiens_2059 | MN908947.3 | 19605 | 20198 | + | ORF1ab | 5 | circRNA_finder | SRR11550046 |
| SARS-CoV-2_circ_Homo_sapiens_2208 | MN908947.3 | 21894 | 23561 | + | S | 5 | circRNA_finder | SRR11550045 |
| SARS-CoV-2_circ_Homo_sapiens_2208 | MN908947.3 | 21894 | 23561 | + | S | 5 | find_circ | SRR11550045 |
| SARS-CoV-2_circ_Homo_sapiens_2208 | MN908947.3 | 21894 | 23561 | + | S | 5 | CIRI2 | SRR11550045 |
| SARS-CoV-2_circ_Homo_sapiens_2386 | MN908947.3 | 23568 | 25428 | + | ORF3a;S | 5 | find_circ | SRR11550046 |
| SARS-CoV-2_circ_Homo_sapiens_240 | MN908947.3 | 1009 | 3344 | + | ORF1ab | 5 | find_circ | SRR11550045 |
| SARS-CoV-2_circ_Homo_sapiens_2411 | MN908947.3 | 23588 | 24200 | + | S | 5 | find_circ | SRR11550045 |
| SARS-CoV-2_circ_Homo_sapiens_2412 | MN908947.3 | 23588 | 25428 | + | ORF3a;S | 5 | find_circ | SRR11550045 |
| SARS-CoV-2_circ_Homo_sapiens_2412 | MN908947.3 | 23588 | 25428 | + | ORF3a;S | 5 | circRNA_finder | SRR11550045 |
| SARS-CoV-2_circ_Homo_sapiens_2420 | MN908947.3 | 23625 | 24192 | + | S | 5 | find_circ | SRR11550043 |
| SARS-CoV-2_circ_Homo_sapiens_2470 | MN908947.3 | 24027 | 25641 | - | ORF3a;S | 5 | find_circ | SRR11550044 |
| SARS-CoV-2_circ_Homo_sapiens_2471 | MN908947.3 | 24027 | 25641 | + | ORF3a;S | 5 | circRNA_finder | SRR11550044 |
| SARS-CoV-2_circ_Homo_sapiens_2498 | MN908947.3 | 24277 | 25061 | + | S | 5 | CIRI2 | SRR11550043 |
| SARS-CoV-2_circ_Homo_sapiens_2554 | MN908947.3 | 24954 | 25476 | + | ORF3a;S | 5 | circRNA_finder | SRR11550046 |
| SARS-CoV-2_circ_Homo_sapiens_2561 | MN908947.3 | 24990 | 25516 | - | ORF3a;S | 5 | find_circ | SRR11550044 |
| SARS-CoV-2_circ_Homo_sapiens_2563 | MN908947.3 | 24990 | 25548 | - | ORF3a;S | 5 | CIRI2 | SRR11550045 |
| SARS-CoV-2_circ_Homo_sapiens_2634 | MN908947.3 | 25514 | 26567 | - | ORF3a;M;E | 5 | find_circ | SRR11550044 |
| SARS-CoV-2_circ_Homo_sapiens_266 | MN908947.3 | 1404 | 1918 | - | ORF1ab | 5 | find_circ | SRR11550046 |
| SARS-CoV-2_circ_Homo_sapiens_267 | MN908947.3 | 1404 | 1918 | + | ORF1ab | 5 | circRNA_finder | SRR11550046 |
| SARS-CoV-2_circ_Homo_sapiens_2670 | MN908947.3 | 25672 | 26241 | - | ORF3a | 5 | find_circ | SRR11550043 |
| SARS-CoV-2_circ_Homo_sapiens_2671 | MN908947.3 | 25672 | 26241 | + | ORF3a | 5 | circRNA_finder | SRR11550043 |
| SARS-CoV-2_circ_Homo_sapiens_2680 | MN908947.3 | 25672 | 26404 | - | ORF3a;E | 5 | find_circ | SRR11550046 |
| SARS-CoV-2_circ_Homo_sapiens_2680 | MN908947.3 | 25672 | 26404 | - | ORF3a;E | 5 | CIRI2 | SRR11550046 |
| SARS-CoV-2_circ_Homo_sapiens_2681 | MN908947.3 | 25672 | 26404 | + | ORF3a;E | 5 | circRNA_finder | SRR11550046 |
| SARS-CoV-2_circ_Homo_sapiens_2690 | MN908947.3 | 25672 | 26775 | - | ORF3a;M;E | 5 | CIRI2 | SRR11550043 |
| SARS-CoV-2_circ_Homo_sapiens_2690 | MN908947.3 | 25672 | 26775 | - | ORF3a;M;E | 5 | find_circ | SRR11550046 |
| SARS-CoV-2_circ_Homo_sapiens_2691 | MN908947.3 | 25672 | 26775 | + | ORF3a;M;E | 5 | circRNA_finder | SRR11550046 |
| SARS-CoV-2_circ_Homo_sapiens_2692 | MN908947.3 | 25672 | 26780 | - | ORF3a;M;E | 5 | CIRI2 | SRR11550046 |
| SARS-CoV-2_circ_Homo_sapiens_2695 | MN908947.3 | 25672 | 26817 | - | ORF3a;M;E | 5 | find_circ | SRR11550045 |
| SARS-CoV-2_circ_Homo_sapiens_2696 | MN908947.3 | 25672 | 26817 | + | ORF3a;M;E | 5 | circRNA_finder | SRR11550045 |
| SARS-CoV-2_circ_Homo_sapiens_2705 | MN908947.3 | 25672 | 27047 | - | ORF3a;M;E | 5 | CIRI2 | SRR11550044 |
| SARS-CoV-2_circ_Homo_sapiens_2738 | MN908947.3 | 25811 | 26570 | + | ORF3a;M;E | 5 | circRNA_finder | SRR11550046 |
| SARS-CoV-2_circ_Homo_sapiens_2797 | MN908947.3 | 26825 | 27270 | + | ORF6;M | 5 | find_circ | SRR11550046 |
| SARS-CoV-2_circ_Homo_sapiens_280 | MN908947.3 | 1520 | 2155 | - | ORF1ab | 5 | find_circ | SRR11550045 |
| SARS-CoV-2_circ_Homo_sapiens_2803 | MN908947.3 | 26827 | 27480 | - | ORF7a;ORF6;M | 5 | find_circ | SRR11550043 |
| SARS-CoV-2_circ_Homo_sapiens_2830 | MN908947.3 | 26983 | 27130 | - | M | 5 | find_circ | SRR11550046 |
| SARS-CoV-2_circ_Homo_sapiens_2929 | MN908947.3 | 27494 | 28960 | - | ORF7a;ORF8;ORF7b;N | 5 | CIRI2 | SRR11550044 |
| SARS-CoV-2_circ_Homo_sapiens_2933 | MN908947.3 | 27576 | 28092 | - | ORF7a;ORF8;ORF7b | 5 | find_circ | SRR11550044 |
| SARS-CoV-2_circ_Homo_sapiens_2950 | MN908947.3 | 27626 | 29534 | - | ORF7a;ORF8;ORF7b;N | 5 | find_circ | SRR11550046 |
| SARS-CoV-2_circ_Homo_sapiens_2951 | MN908947.3 | 27626 | 29534 | + | ORF7a;ORF8;ORF7b;N | 5 | circRNA_finder | SRR11550046 |
| SARS-CoV-2_circ_Homo_sapiens_2979 | MN908947.3 | 27799 | 28320 | + | ORF8;ORF7b;N | 5 | find_circ | SRR11550046 |
| SARS-CoV-2_circ_Homo_sapiens_2981 | MN908947.3 | 27799 | 28403 | + | ORF8;ORF7b;N | 5 | find_circ | SRR11550044 |
| SARS-CoV-2_circ_Homo_sapiens_2998 | MN908947.3 | 28102 | 28472 | - | ORF8;N | 5 | find_circ | SRR11550046 |
| SARS-CoV-2_circ_Homo_sapiens_3014 | MN908947.3 | 28301 | 28556 | + | N | 5 | circRNA_finder | SRR11550046 |
| SARS-CoV-2_circ_Homo_sapiens_3014 | MN908947.3 | 28301 | 28556 | + | N | 5 | find_circ | SRR11550046 |
| SARS-CoV-2_circ_Homo_sapiens_3048 | MN908947.3 | 28404 | 28669 | + | N | 5 | CIRI2 | SRR11550045 |
| SARS-CoV-2_circ_Homo_sapiens_3048 | MN908947.3 | 28404 | 28669 | + | N | 5 | find_circ | SRR11550045 |
| SARS-CoV-2_circ_Homo_sapiens_3051 | MN908947.3 | 28404 | 28850 | + | N | 5 | CIRI2 | SRR11550046 |
| SARS-CoV-2_circ_Homo_sapiens_3051 | MN908947.3 | 28404 | 28850 | + | N | 5 | CIRI2 | SRR11550043 |
| SARS-CoV-2_circ_Homo_sapiens_3058 | MN908947.3 | 28404 | 29080 | + | N | 5 | circRNA_finder | SRR11550044 |
| SARS-CoV-2_circ_Homo_sapiens_3062 | MN908947.3 | 28404 | 29572 | + | ORF10;N | 5 | find_circ | SRR11550046 |
| SARS-CoV-2_circ_Homo_sapiens_3065 | MN908947.3 | 28410 | 28646 | - | N | 5 | find_circ | SRR11550044 |
| SARS-CoV-2_circ_Homo_sapiens_3076 | MN908947.3 | 28410 | 28895 | - | N | 5 | find_circ | SRR11550045 |
| SARS-CoV-2_circ_Homo_sapiens_3077 | MN908947.3 | 28410 | 28895 | + | N | 5 | circRNA_finder | SRR11550045 |
| SARS-CoV-2_circ_Homo_sapiens_3083 | MN908947.3 | 28410 | 29012 | + | N | 5 | circRNA_finder | SRR11550046 |
| SARS-CoV-2_circ_Homo_sapiens_3088 | MN908947.3 | 28410 | 29329 | - | N | 5 | find_circ | SRR11550045 |
| SARS-CoV-2_circ_Homo_sapiens_3096 | MN908947.3 | 28420 | 28609 | - | N | 5 | find_circ | SRR11550045 |
| SARS-CoV-2_circ_Homo_sapiens_3111 | MN908947.3 | 28449 | 28895 | - | N | 5 | find_circ | SRR11550044 |
| SARS-CoV-2_circ_Homo_sapiens_3136 | MN908947.3 | 28463 | 28927 | - | N | 5 | CIRI2 | SRR11550044 |
| SARS-CoV-2_circ_Homo_sapiens_3138 | MN908947.3 | 28463 | 28931 | - | N | 5 | CIRI2 | SRR11550045 |
| SARS-CoV-2_circ_Homo_sapiens_3142 | MN908947.3 | 28463 | 28960 | - | N | 5 | find_circ | SRR11550043 |
| SARS-CoV-2_circ_Homo_sapiens_3150 | MN908947.3 | 28463 | 29066 | - | N | 5 | find_circ | SRR11550045 |
| SARS-CoV-2_circ_Homo_sapiens_3164 | MN908947.3 | 28463 | 29607 | - | ORF10;N | 5 | CIRI2 | SRR11550046 |
| SARS-CoV-2_circ_Homo_sapiens_3184 | MN908947.3 | 28551 | 29066 | - | N | 5 | find_circ | SRR11550045 |
| SARS-CoV-2_circ_Homo_sapiens_3205 | MN908947.3 | 28610 | 28895 | - | N | 5 | find_circ | SRR11550046 |
| SARS-CoV-2_circ_Homo_sapiens_3206 | MN908947.3 | 28610 | 28895 | + | N | 5 | circRNA_finder | SRR11550043 |
| SARS-CoV-2_circ_Homo_sapiens_3212 | MN908947.3 | 28610 | 29012 | - | N | 5 | CIRI2 | SRR11550044 |
| SARS-CoV-2_circ_Homo_sapiens_3213 | MN908947.3 | 28610 | 29012 | + | N | 5 | circRNA_finder | SRR11550045 |
| SARS-CoV-2_circ_Homo_sapiens_3223 | MN908947.3 | 28652 | 29012 | - | N | 5 | find_circ | SRR11550044 |
| SARS-CoV-2_circ_Homo_sapiens_3241 | MN908947.3 | 28696 | 29012 | + | N | 5 | circRNA_finder | SRR11550044 |
| SARS-CoV-2_circ_Homo_sapiens_3252 | MN908947.3 | 28709 | 29066 | - | N | 5 | find_circ | SRR11550045 |
| SARS-CoV-2_circ_Homo_sapiens_3252 | MN908947.3 | 28709 | 29066 | - | N | 5 | find_circ | SRR11550046 |
| SARS-CoV-2_circ_Homo_sapiens_3269 | MN908947.3 | 28771 | 29066 | - | N | 5 | find_circ | SRR11550045 |
| SARS-CoV-2_circ_Homo_sapiens_3294 | MN908947.3 | 28967 | 29600 | + | ORF10;N | 5 | find_circ | SRR11550046 |
| SARS-CoV-2_circ_Homo_sapiens_3301 | MN908947.3 | 29073 | 29650 | + | ORF10;N | 5 | find_circ | SRR11550046 |
| SARS-CoV-2_circ_Homo_sapiens_3320 | MN908947.3 | 29086 | 29665 | - | ORF10;N | 5 | find_circ | SRR11550044 |
| SARS-CoV-2_circ_Homo_sapiens_3343 | MN908947.3 | 29172 | 29444 | - | N | 5 | find_circ | SRR11550043 |
| SARS-CoV-2_circ_Homo_sapiens_3351 | MN908947.3 | 29172 | 29579 | + | ORF10;N | 5 | circRNA_finder | SRR11550045 |
| SARS-CoV-2_circ_Homo_sapiens_3362 | MN908947.3 | 29190 | 29579 | - | ORF10;N | 5 | find_circ | SRR11550045 |
| SARS-CoV-2_circ_Homo_sapiens_3363 | MN908947.3 | 29190 | 29658 | - | ORF10;N | 5 | find_circ | SRR11550044 |
| SARS-CoV-2_circ_Homo_sapiens_3372 | MN908947.3 | 29250 | 29579 | - | ORF10;N | 5 | CIRI2 | SRR11550046 |
| SARS-CoV-2_circ_Homo_sapiens_3372 | MN908947.3 | 29250 | 29579 | - | ORF10;N | 5 | find_circ | SRR11550046 |
| SARS-CoV-2_circ_Homo_sapiens_3421 | MN908947.3 | 29348 | 29534 | - | N | 5 | find_circ | SRR11550043 |
| SARS-CoV-2_circ_Homo_sapiens_3421 | MN908947.3 | 29348 | 29534 | - | N | 5 | find_circ | SRR11550046 |
| SARS-CoV-2_circ_Homo_sapiens_347 | MN908947.3 | 2495 | 3353 | + | ORF1ab | 5 | find_circ | SRR11550046 |
| SARS-CoV-2_circ_Homo_sapiens_363 | MN908947.3 | 2571 | 3263 | - | ORF1ab | 5 | find_circ | SRR11550046 |
| SARS-CoV-2_circ_Homo_sapiens_370 | MN908947.3 | 2571 | 3542 | - | ORF1ab | 5 | find_circ | SRR11550045 |
| SARS-CoV-2_circ_Homo_sapiens_370 | MN908947.3 | 2571 | 3542 | - | ORF1ab | 5 | find_circ | SRR11550044 |
| SARS-CoV-2_circ_Homo_sapiens_397 | MN908947.3 | 2799 | 3542 | - | ORF1ab | 5 | find_circ | SRR11550046 |
| SARS-CoV-2_circ_Homo_sapiens_428 | MN908947.3 | 3177 | 3542 | - | ORF1ab | 5 | find_circ | SRR11550043 |
| SARS-CoV-2_circ_Homo_sapiens_429 | MN908947.3 | 3177 | 3542 | + | ORF1ab | 5 | circRNA_finder | SRR11550043 |
| SARS-CoV-2_circ_Homo_sapiens_452 | MN908947.3 | 3447 | 9104 | + | ORF1ab | 5 | find_circ | SRR11550046 |
| SARS-CoV-2_circ_Homo_sapiens_454 | MN908947.3 | 3458 | 5078 | - | ORF1ab | 5 | find_circ | SRR11550045 |
| SARS-CoV-2_circ_Homo_sapiens_456 | MN908947.3 | 3540 | 4487 | + | ORF1ab | 5 | find_circ | SRR11550046 |
| SARS-CoV-2_circ_Homo_sapiens_57 | MN908947.3 | 16 | 28609 | - | ORF6;M;E;ORF3a;S;N;ORF8;ORF7a;ORF7b;ORF1ab | 5 | find_circ | SRR11550044 |
| SARS-CoV-2_circ_Homo_sapiens_589 | MN908947.3 | 4836 | 5557 | + | ORF1ab | 5 | CIRI2 | SRR11550045 |
| SARS-CoV-2_circ_Homo_sapiens_637 | MN908947.3 | 4966 | 5816 | - | ORF1ab | 5 | find_circ | SRR11550045 |
| SARS-CoV-2_circ_Homo_sapiens_664 | MN908947.3 | 5130 | 5821 | - | ORF1ab | 5 | find_circ | SRR11550045 |
| SARS-CoV-2_circ_Homo_sapiens_665 | MN908947.3 | 5130 | 5821 | + | ORF1ab | 5 | circRNA_finder | SRR11550045 |
| SARS-CoV-2_circ_Homo_sapiens_7 | MN908947.3 | 8 | 520 | + | ORF1ab | 5 | find_circ | SRR11550044 |
| SARS-CoV-2_circ_Homo_sapiens_746 | MN908947.3 | 5827 | 6379 | + | ORF1ab | 5 | circRNA_finder | SRR11550046 |
| SARS-CoV-2_circ_Homo_sapiens_799 | MN908947.3 | 6526 | 12694 | - | ORF1ab | 5 | find_circ | SRR11550045 |
| SARS-CoV-2_circ_Homo_sapiens_839 | MN908947.3 | 7839 | 8188 | + | ORF1ab | 5 | circRNA_finder | SRR11550046 |
| SARS-CoV-2_circ_Homo_sapiens_855 | MN908947.3 | 8293 | 8654 | - | ORF1ab | 5 | CIRI2 | SRR11550045 |
| SARS-CoV-2_circ_Homo_sapiens_855 | MN908947.3 | 8293 | 8654 | - | ORF1ab | 5 | CIRI2 | SRR11550044 |
| SARS-CoV-2_circ_Homo_sapiens_903 | MN908947.3 | 8771 | 10038 | + | ORF1ab | 5 | circRNA_finder | SRR11550043 |
| SARS-CoV-2_circ_Homo_sapiens_91 | MN908947.3 | 72 | 449 | - | ORF1ab | 5 | find_circ | SRR11550045 |
| SARS-CoV-2_circ_Homo_sapiens_97 | MN908947.3 | 237 | 442 | + | ORF1ab | 5 | find_circ | SRR11550046 |
| SARS-CoV-2_circ_Homo_sapiens_1010 | MN908947.3 | 9951 | 10589 | + | ORF1ab | 4 | CIRI2 | SRR11550044 |
| SARS-CoV-2_circ_Homo_sapiens_1036 | MN908947.3 | 10066 | 28556 | + | ORF6;M;E;ORF3a;S;N;ORF8;ORF7a;ORF7b;ORF1ab | 4 | circRNA_finder | SRR11550046 |
| SARS-CoV-2_circ_Homo_sapiens_1036 | MN908947.3 | 10066 | 28556 | + | ORF6;M;E;ORF3a;S;N;ORF8;ORF7a;ORF7b;ORF1ab | 4 | find_circ | SRR11550046 |
| SARS-CoV-2_circ_Homo_sapiens_1079 | MN908947.3 | 10590 | 12220 | + | ORF1ab | 4 | find_circ | SRR11550045 |
| SARS-CoV-2_circ_Homo_sapiens_110 | MN908947.3 | 312 | 3542 | - | ORF1ab | 4 | find_circ | SRR11550046 |
| SARS-CoV-2_circ_Homo_sapiens_1107 | MN908947.3 | 10616 | 11102 | - | ORF1ab | 4 | CIRI2 | SRR11550045 |
| SARS-CoV-2_circ_Homo_sapiens_1107 | MN908947.3 | 10616 | 11102 | - | ORF1ab | 4 | find_circ | SRR11550045 |
| SARS-CoV-2_circ_Homo_sapiens_1108 | MN908947.3 | 10616 | 11102 | + | ORF1ab | 4 | circRNA_finder | SRR11550045 |
| SARS-CoV-2_circ_Homo_sapiens_1114 | MN908947.3 | 10659 | 11174 | + | ORF1ab | 4 | find_circ | SRR11550044 |
| SARS-CoV-2_circ_Homo_sapiens_1128 | MN908947.3 | 10671 | 10939 | + | ORF1ab | 4 | circRNA_finder | SRR11550046 |
| SARS-CoV-2_circ_Homo_sapiens_1163 | MN908947.3 | 11190 | 11816 | - | ORF1ab | 4 | CIRI2 | SRR11550046 |
| SARS-CoV-2_circ_Homo_sapiens_1190 | MN908947.3 | 11488 | 12007 | - | ORF1ab | 4 | find_circ | SRR11550045 |
| SARS-CoV-2_circ_Homo_sapiens_1191 | MN908947.3 | 11488 | 12007 | + | ORF1ab | 4 | circRNA_finder | SRR11550045 |
| SARS-CoV-2_circ_Homo_sapiens_1194 | MN908947.3 | 11488 | 12137 | - | ORF1ab | 4 | find_circ | SRR11550046 |
| SARS-CoV-2_circ_Homo_sapiens_1194 | MN908947.3 | 11488 | 12137 | - | ORF1ab | 4 | find_circ | SRR11550045 |
| SARS-CoV-2_circ_Homo_sapiens_12 | MN908947.3 | 8 | 2080 | + | ORF1ab | 4 | find_circ | SRR11550046 |
| SARS-CoV-2_circ_Homo_sapiens_1208 | MN908947.3 | 11502 | 13145 | + | ORF1ab | 4 | CIRI2 | SRR11550045 |
| SARS-CoV-2_circ_Homo_sapiens_1236 | MN908947.3 | 11645 | 12137 | - | ORF1ab | 4 | find_circ | SRR11550046 |
| SARS-CoV-2_circ_Homo_sapiens_1262 | MN908947.3 | 11705 | 12749 | - | ORF1ab | 4 | find_circ | SRR11550044 |
| SARS-CoV-2_circ_Homo_sapiens_1270 | MN908947.3 | 11709 | 12013 | + | ORF1ab | 4 | circRNA_finder | SRR11550046 |
| SARS-CoV-2_circ_Homo_sapiens_1270 | MN908947.3 | 11709 | 12013 | + | ORF1ab | 4 | find_circ | SRR11550046 |
| SARS-CoV-2_circ_Homo_sapiens_1323 | MN908947.3 | 12093 | 13458 | + | ORF1ab | 4 | find_circ | SRR11550045 |
| SARS-CoV-2_circ_Homo_sapiens_1344 | MN908947.3 | 12153 | 12646 | + | ORF1ab | 4 | find_circ | SRR11550045 |
| SARS-CoV-2_circ_Homo_sapiens_1345 | MN908947.3 | 12153 | 12768 | + | ORF1ab | 4 | find_circ | SRR11550044 |
| SARS-CoV-2_circ_Homo_sapiens_1355 | MN908947.3 | 12153 | 14899 | + | ORF1ab | 4 | find_circ | SRR11550046 |
| SARS-CoV-2_circ_Homo_sapiens_1383 | MN908947.3 | 12288 | 12805 | + | ORF1ab | 4 | circRNA_finder | SRR11550045 |
| SARS-CoV-2_circ_Homo_sapiens_1383 | MN908947.3 | 12288 | 12805 | + | ORF1ab | 4 | find_circ | SRR11550045 |
| SARS-CoV-2_circ_Homo_sapiens_1400 | MN908947.3 | 12343 | 12773 | + | ORF1ab | 4 | circRNA_finder | SRR11550046 |
| SARS-CoV-2_circ_Homo_sapiens_1464 | MN908947.3 | 12974 | 15319 | - | ORF1ab | 4 | CIRI2 | SRR11550045 |
| SARS-CoV-2_circ_Homo_sapiens_1478 | MN908947.3 | 13000 | 13385 | + | ORF1ab | 4 | find_circ | SRR11550045 |
| SARS-CoV-2_circ_Homo_sapiens_1481 | MN908947.3 | 13000 | 16500 | + | ORF1ab | 4 | find_circ | SRR11550046 |
| SARS-CoV-2_circ_Homo_sapiens_1518 | MN908947.3 | 13500 | 14598 | - | ORF1ab | 4 | find_circ | SRR11550045 |
| SARS-CoV-2_circ_Homo_sapiens_1533 | MN908947.3 | 13514 | 17089 | + | ORF1ab | 4 | find_circ | SRR11550045 |
| SARS-CoV-2_circ_Homo_sapiens_1534 | MN908947.3 | 13518 | 14572 | - | ORF1ab | 4 | find_circ | SRR11550046 |
| SARS-CoV-2_circ_Homo_sapiens_1540 | MN908947.3 | 13521 | 15733 | + | ORF1ab | 4 | find_circ | SRR11550046 |
| SARS-CoV-2_circ_Homo_sapiens_1548 | MN908947.3 | 13736 | 14177 | - | ORF1ab | 4 | find_circ | SRR11550045 |
| SARS-CoV-2_circ_Homo_sapiens_1549 | MN908947.3 | 13736 | 14177 | + | ORF1ab | 4 | circRNA_finder | SRR11550045 |
| SARS-CoV-2_circ_Homo_sapiens_1574 | MN908947.3 | 14178 | 14535 | - | ORF1ab | 4 | CIRI2 | SRR11550044 |
| SARS-CoV-2_circ_Homo_sapiens_1574 | MN908947.3 | 14178 | 14535 | - | ORF1ab | 4 | find_circ | SRR11550046 |
| SARS-CoV-2_circ_Homo_sapiens_1577 | MN908947.3 | 14178 | 14572 | - | ORF1ab | 4 | CIRI2 | SRR11550046 |
| SARS-CoV-2_circ_Homo_sapiens_1584 | MN908947.3 | 14178 | 14598 | - | ORF1ab | 4 | find_circ | SRR11550043 |
| SARS-CoV-2_circ_Homo_sapiens_1585 | MN908947.3 | 14178 | 14598 | + | ORF1ab | 4 | circRNA_finder | SRR11550043 |
| SARS-CoV-2_circ_Homo_sapiens_1603 | MN908947.3 | 14184 | 15852 | - | ORF1ab | 4 | CIRI2 | SRR11550046 |
| SARS-CoV-2_circ_Homo_sapiens_1634 | MN908947.3 | 14609 | 15574 | + | ORF1ab | 4 | circRNA_finder | SRR11550043 |
| SARS-CoV-2_circ_Homo_sapiens_1634 | MN908947.3 | 14609 | 15574 | + | ORF1ab | 4 | find_circ | SRR11550043 |
| SARS-CoV-2_circ_Homo_sapiens_165 | MN908947.3 | 698 | 1283 | - | ORF1ab | 4 | find_circ | SRR11550045 |
| SARS-CoV-2_circ_Homo_sapiens_1731 | MN908947.3 | 15744 | 16297 | + | ORF1ab | 4 | circRNA_finder | SRR11550045 |
| SARS-CoV-2_circ_Homo_sapiens_1731 | MN908947.3 | 15744 | 16297 | + | ORF1ab | 4 | find_circ | SRR11550045 |
| SARS-CoV-2_circ_Homo_sapiens_1742 | MN908947.3 | 15853 | 16288 | - | ORF1ab | 4 | find_circ | SRR11550044 |
| SARS-CoV-2_circ_Homo_sapiens_1776 | MN908947.3 | 16397 | 18897 | + | ORF1ab | 4 | CIRI2 | SRR11550045 |
| SARS-CoV-2_circ_Homo_sapiens_1784 | MN908947.3 | 16552 | 16970 | - | ORF1ab | 4 | find_circ | SRR11550045 |
| SARS-CoV-2_circ_Homo_sapiens_1785 | MN908947.3 | 16552 | 16970 | + | ORF1ab | 4 | circRNA_finder | SRR11550045 |
| SARS-CoV-2_circ_Homo_sapiens_1791 | MN908947.3 | 16552 | 28462 | - | ORF6;M;E;ORF3a;S;N;ORF8;ORF7a;ORF7b;ORF1ab | 4 | find_circ | SRR11550046 |
| SARS-CoV-2_circ_Homo_sapiens_1804 | MN908947.3 | 16751 | 17527 | - | ORF1ab | 4 | find_circ | SRR11550046 |
| SARS-CoV-2_circ_Homo_sapiens_1807 | MN908947.3 | 16751 | 18876 | - | ORF1ab | 4 | CIRI2 | SRR11550045 |
| SARS-CoV-2_circ_Homo_sapiens_1812 | MN908947.3 | 16755 | 17426 | + | ORF1ab | 4 | find_circ | SRR11550044 |
| SARS-CoV-2_circ_Homo_sapiens_1812 | MN908947.3 | 16755 | 17426 | + | ORF1ab | 4 | circRNA_finder | SRR11550044 |
| SARS-CoV-2_circ_Homo_sapiens_1828 | MN908947.3 | 17101 | 18024 | + | ORF1ab | 4 | find_circ | SRR11550045 |
| SARS-CoV-2_circ_Homo_sapiens_1832 | MN908947.3 | 17342 | 18016 | + | ORF1ab | 4 | find_circ | SRR11550045 |
| SARS-CoV-2_circ_Homo_sapiens_1844 | MN908947.3 | 17406 | 18815 | + | ORF1ab | 4 | find_circ | SRR11550045 |
| SARS-CoV-2_circ_Homo_sapiens_1854 | MN908947.3 | 17411 | 28146 | - | ORF6;M;E;ORF3a;S;ORF8;ORF7a;ORF7b;ORF1ab | 4 | find_circ | SRR11550046 |
| SARS-CoV-2_circ_Homo_sapiens_1884 | MN908947.3 | 18017 | 18205 | - | ORF1ab | 4 | find_circ | SRR11550045 |
| SARS-CoV-2_circ_Homo_sapiens_1897 | MN908947.3 | 18017 | 18656 | - | ORF1ab | 4 | find_circ | SRR11550045 |
| SARS-CoV-2_circ_Homo_sapiens_19 | MN908947.3 | 8 | 8257 | + | ORF1ab | 4 | CIRI2 | SRR11550046 |
| SARS-CoV-2_circ_Homo_sapiens_1901 | MN908947.3 | 18017 | 18876 | - | ORF1ab | 4 | find_circ | SRR11550045 |
| SARS-CoV-2_circ_Homo_sapiens_1902 | MN908947.3 | 18017 | 18876 | + | ORF1ab | 4 | circRNA_finder | SRR11550045 |
| SARS-CoV-2_circ_Homo_sapiens_1907 | MN908947.3 | 18017 | 19603 | - | ORF1ab | 4 | find_circ | SRR11550045 |
| SARS-CoV-2_circ_Homo_sapiens_1918 | MN908947.3 | 18021 | 18379 | + | ORF1ab | 4 | circRNA_finder | SRR11550045 |
| SARS-CoV-2_circ_Homo_sapiens_1923 | MN908947.3 | 18021 | 18815 | + | ORF1ab | 4 | CIRI2 | SRR11550046 |
| SARS-CoV-2_circ_Homo_sapiens_1934 | MN908947.3 | 18041 | 19074 | + | ORF1ab | 4 | find_circ | SRR11550046 |
| SARS-CoV-2_circ_Homo_sapiens_1949 | MN908947.3 | 18282 | 18744 | + | ORF1ab | 4 | circRNA_finder | SRR11550043 |
| SARS-CoV-2_circ_Homo_sapiens_1949 | MN908947.3 | 18282 | 18744 | + | ORF1ab | 4 | circRNA_finder | SRR11550046 |
| SARS-CoV-2_circ_Homo_sapiens_1949 | MN908947.3 | 18282 | 18744 | + | ORF1ab | 4 | find_circ | SRR11550046 |
| SARS-CoV-2_circ_Homo_sapiens_1949 | MN908947.3 | 18282 | 18744 | + | ORF1ab | 4 | find_circ | SRR11550043 |
| SARS-CoV-2_circ_Homo_sapiens_1968 | MN908947.3 | 18380 | 18850 | + | ORF1ab | 4 | circRNA_finder | SRR11550045 |
| SARS-CoV-2_circ_Homo_sapiens_1968 | MN908947.3 | 18380 | 18850 | + | ORF1ab | 4 | find_circ | SRR11550045 |
| SARS-CoV-2_circ_Homo_sapiens_1972 | MN908947.3 | 18401 | 18663 | - | ORF1ab | 4 | find_circ | SRR11550045 |
| SARS-CoV-2_circ_Homo_sapiens_1976 | MN908947.3 | 18407 | 21607 | + | S;ORF1ab | 4 | CIRI2 | SRR11550043 |
| SARS-CoV-2_circ_Homo_sapiens_1984 | MN908947.3 | 18494 | 19501 | - | ORF1ab | 4 | find_circ | SRR11550044 |
| SARS-CoV-2_circ_Homo_sapiens_1991 | MN908947.3 | 18556 | 19693 | - | ORF1ab | 4 | find_circ | SRR11550045 |
| SARS-CoV-2_circ_Homo_sapiens_1995 | MN908947.3 | 18657 | 18876 | - | ORF1ab | 4 | find_circ | SRR11550046 |
| SARS-CoV-2_circ_Homo_sapiens_1995 | MN908947.3 | 18657 | 18876 | - | ORF1ab | 4 | find_circ | SRR11550045 |
| SARS-CoV-2_circ_Homo_sapiens_1996 | MN908947.3 | 18657 | 18876 | + | ORF1ab | 4 | circRNA_finder | SRR11550046 |
| SARS-CoV-2_circ_Homo_sapiens_2031 | MN908947.3 | 18982 | 19982 | - | ORF1ab | 4 | find_circ | SRR11550045 |
| SARS-CoV-2_circ_Homo_sapiens_204 | MN908947.3 | 897 | 1244 | + | ORF1ab | 4 | find_circ | SRR11550046 |
| SARS-CoV-2_circ_Homo_sapiens_2051 | MN908947.3 | 19603 | 20198 | - | ORF1ab | 4 | find_circ | SRR11550046 |
| SARS-CoV-2_circ_Homo_sapiens_2065 | MN908947.3 | 19611 | 20203 | - | ORF1ab | 4 | find_circ | SRR11550043 |
| SARS-CoV-2_circ_Homo_sapiens_2072 | MN908947.3 | 19764 | 26241 | - | ORF3a;S;ORF1ab | 4 | find_circ | SRR11550043 |
| SARS-CoV-2_circ_Homo_sapiens_2109 | MN908947.3 | 20549 | 21400 | + | ORF1ab | 4 | find_circ | SRR11550043 |
| SARS-CoV-2_circ_Homo_sapiens_2120 | MN908947.3 | 20768 | 21726 | - | S;ORF1ab | 4 | find_circ | SRR11550043 |
| SARS-CoV-2_circ_Homo_sapiens_2147 | MN908947.3 | 21594 | 21723 | + | S | 4 | find_circ | SRR11550046 |
| SARS-CoV-2_circ_Homo_sapiens_2157 | MN908947.3 | 21677 | 27790 | - | ORF6;M;E;ORF3a;S;ORF7a;ORF7b | 4 | find_circ | SRR11550044 |
| SARS-CoV-2_circ_Homo_sapiens_2168 | MN908947.3 | 21722 | 23033 | - | S | 4 | find_circ | SRR11550046 |
| SARS-CoV-2_circ_Homo_sapiens_2194 | MN908947.3 | 21816 | 23372 | - | S | 4 | find_circ | SRR11550045 |
| SARS-CoV-2_circ_Homo_sapiens_2223 | MN908947.3 | 21998 | 26788 | - | ORF3a;S;M;E | 4 | find_circ | SRR11550046 |
| SARS-CoV-2_circ_Homo_sapiens_2244 | MN908947.3 | 22377 | 28895 | - | ORF6;M;E;ORF3a;S;N;ORF8;ORF7a;ORF7b | 4 | find_circ | SRR11550043 |
| SARS-CoV-2_circ_Homo_sapiens_2245 | MN908947.3 | 22377 | 28895 | + | ORF6;M;E;ORF3a;S;N;ORF8;ORF7a;ORF7b | 4 | circRNA_finder | SRR11550043 |
| SARS-CoV-2_circ_Homo_sapiens_2253 | MN908947.3 | 22422 | 23425 | + | S | 4 | circRNA_finder | SRR11550046 |
| SARS-CoV-2_circ_Homo_sapiens_2253 | MN908947.3 | 22422 | 23425 | + | S | 4 | find_circ | SRR11550046 |
| SARS-CoV-2_circ_Homo_sapiens_2275 | MN908947.3 | 22950 | 23305 | - | S | 4 | CIRI2 | SRR11550045 |
| SARS-CoV-2_circ_Homo_sapiens_2277 | MN908947.3 | 22950 | 23392 | - | S | 4 | CIRI2 | SRR11550043 |
| SARS-CoV-2_circ_Homo_sapiens_2279 | MN908947.3 | 22950 | 23446 | - | S | 4 | CIRI2 | SRR11550044 |
| SARS-CoV-2_circ_Homo_sapiens_2279 | MN908947.3 | 22950 | 23446 | - | S | 4 | CIRI2 | SRR11550045 |
| SARS-CoV-2_circ_Homo_sapiens_228 | MN908947.3 | 1009 | 1232 | + | ORF1ab | 4 | find_circ | SRR11550046 |
| SARS-CoV-2_circ_Homo_sapiens_230 | MN908947.3 | 1009 | 1357 | + | ORF1ab | 4 | find_circ | SRR11550043 |
| SARS-CoV-2_circ_Homo_sapiens_2326 | MN908947.3 | 23142 | 23624 | - | S | 4 | CIRI2 | SRR11550046 |
| SARS-CoV-2_circ_Homo_sapiens_2353 | MN908947.3 | 23312 | 25283 | + | S | 4 | find_circ | SRR11550043 |
| SARS-CoV-2_circ_Homo_sapiens_2379 | MN908947.3 | 23568 | 24192 | + | S | 4 | find_circ | SRR11550045 |
| SARS-CoV-2_circ_Homo_sapiens_2384 | MN908947.3 | 23568 | 25243 | + | S | 4 | CIRI2 | SRR11550045 |
| SARS-CoV-2_circ_Homo_sapiens_2394 | MN908947.3 | 23568 | 26783 | + | ORF3a;S;M;E | 4 | CIRI2 | SRR11550046 |
| SARS-CoV-2_circ_Homo_sapiens_2394 | MN908947.3 | 23568 | 26783 | + | ORF3a;S;M;E | 4 | find_circ | SRR11550046 |
| SARS-CoV-2_circ_Homo_sapiens_240 | MN908947.3 | 1009 | 3344 | + | ORF1ab | 4 | find_circ | SRR11550046 |
| SARS-CoV-2_circ_Homo_sapiens_2449 | MN908947.3 | 24021 | 26778 | + | ORF3a;S;M;E | 4 | find_circ | SRR11550045 |
| SARS-CoV-2_circ_Homo_sapiens_2503 | MN908947.3 | 24375 | 24989 | - | S | 4 | find_circ | SRR11550045 |
| SARS-CoV-2_circ_Homo_sapiens_2520 | MN908947.3 | 24463 | 25476 | + | ORF3a;S | 4 | find_circ | SRR11550044 |
| SARS-CoV-2_circ_Homo_sapiens_2562 | MN908947.3 | 24990 | 25516 | + | ORF3a;S | 4 | circRNA_finder | SRR11550044 |
| SARS-CoV-2_circ_Homo_sapiens_2563 | MN908947.3 | 24990 | 25548 | - | ORF3a;S | 4 | find_circ | SRR11550046 |
| SARS-CoV-2_circ_Homo_sapiens_2570 | MN908947.3 | 24990 | 27130 | - | ORF3a;S;M;E | 4 | find_circ | SRR11550044 |
| SARS-CoV-2_circ_Homo_sapiens_2571 | MN908947.3 | 24990 | 27130 | + | ORF3a;S;M;E | 4 | circRNA_finder | SRR11550044 |
| SARS-CoV-2_circ_Homo_sapiens_2574 | MN908947.3 | 24990 | 29707 | - | ORF6;ORF10;M;E;ORF3a;S;N;ORF8;ORF7a;ORF7b | 4 | find_circ | SRR11550043 |
| SARS-CoV-2_circ_Homo_sapiens_2667 | MN908947.3 | 25672 | 26212 | - | ORF3a | 4 | find_circ | SRR11550045 |
| SARS-CoV-2_circ_Homo_sapiens_2673 | MN908947.3 | 25672 | 26304 | - | ORF3a;E | 4 | CIRI2 | SRR11550044 |
| SARS-CoV-2_circ_Homo_sapiens_2676 | MN908947.3 | 25672 | 26343 | - | ORF3a;E | 4 | CIRI2 | SRR11550046 |
| SARS-CoV-2_circ_Homo_sapiens_2683 | MN908947.3 | 25672 | 26541 | - | ORF3a;M;E | 4 | find_circ | SRR11550045 |
| SARS-CoV-2_circ_Homo_sapiens_2686 | MN908947.3 | 25672 | 26570 | + | ORF3a;M;E | 4 | circRNA_finder | SRR11550043 |
| SARS-CoV-2_circ_Homo_sapiens_2692 | MN908947.3 | 25672 | 26780 | - | ORF3a;M;E | 4 | find_circ | SRR11550046 |
| SARS-CoV-2_circ_Homo_sapiens_2692 | MN908947.3 | 25672 | 26780 | - | ORF3a;M;E | 4 | find_circ | SRR11550044 |
| SARS-CoV-2_circ_Homo_sapiens_2693 | MN908947.3 | 25672 | 26780 | + | ORF3a;M;E | 4 | circRNA_finder | SRR11550044 |
| SARS-CoV-2_circ_Homo_sapiens_2697 | MN908947.3 | 25672 | 26868 | - | ORF3a;M;E | 4 | find_circ | SRR11550045 |
| SARS-CoV-2_circ_Homo_sapiens_2708 | MN908947.3 | 25672 | 27130 | - | ORF3a;M;E | 4 | find_circ | SRR11550044 |
| SARS-CoV-2_circ_Homo_sapiens_2728 | MN908947.3 | 25750 | 27015 | - | ORF3a;M;E | 4 | find_circ | SRR11550046 |
| SARS-CoV-2_circ_Homo_sapiens_2737 | MN908947.3 | 25811 | 26570 | - | ORF3a;M;E | 4 | find_circ | SRR11550046 |
| SARS-CoV-2_circ_Homo_sapiens_281 | MN908947.3 | 1520 | 2484 | - | ORF1ab | 4 | find_circ | SRR11550044 |
| SARS-CoV-2_circ_Homo_sapiens_2813 | MN908947.3 | 26874 | 27218 | + | ORF6;M | 4 | circRNA_finder | SRR11550045 |
| SARS-CoV-2_circ_Homo_sapiens_2815 | MN908947.3 | 26874 | 27583 | - | ORF7a;ORF6;M | 4 | find_circ | SRR11550046 |
| SARS-CoV-2_circ_Homo_sapiens_2818 | MN908947.3 | 26874 | 27683 | - | ORF7a;ORF6;M | 4 | find_circ | SRR11550045 |
| SARS-CoV-2_circ_Homo_sapiens_282 | MN908947.3 | 1520 | 2484 | + | ORF1ab | 4 | circRNA_finder | SRR11550044 |
| SARS-CoV-2_circ_Homo_sapiens_2825 | MN908947.3 | 26935 | 29408 | - | ORF6;M;N;ORF8;ORF7a;ORF7b | 4 | find_circ | SRR11550045 |
| SARS-CoV-2_circ_Homo_sapiens_2826 | MN908947.3 | 26935 | 29408 | + | ORF6;M;N;ORF8;ORF7a;ORF7b | 4 | circRNA_finder | SRR11550045 |
| SARS-CoV-2_circ_Homo_sapiens_2831 | MN908947.3 | 27012 | 27558 | - | ORF7a;ORF6;M | 4 | find_circ | SRR11550046 |
| SARS-CoV-2_circ_Homo_sapiens_2871 | MN908947.3 | 27220 | 27678 | - | ORF7a;ORF6 | 4 | find_circ | SRR11550046 |
| SARS-CoV-2_circ_Homo_sapiens_2879 | MN908947.3 | 27226 | 27603 | + | ORF7a;ORF6 | 4 | circRNA_finder | SRR11550044 |
| SARS-CoV-2_circ_Homo_sapiens_2879 | MN908947.3 | 27226 | 27603 | + | ORF7a;ORF6 | 4 | find_circ | SRR11550044 |
| SARS-CoV-2_circ_Homo_sapiens_2937 | MN908947.3 | 27576 | 28895 | - | ORF7a;ORF8;ORF7b;N | 4 | find_circ | SRR11550045 |
| SARS-CoV-2_circ_Homo_sapiens_2939 | MN908947.3 | 27576 | 29066 | - | ORF7a;ORF8;ORF7b;N | 4 | find_circ | SRR11550043 |
| SARS-CoV-2_circ_Homo_sapiens_2942 | MN908947.3 | 27576 | 29787 | - | ORF10;N;ORF8;ORF7a;ORF7b | 4 | find_circ | SRR11550043 |
| SARS-CoV-2_circ_Homo_sapiens_2949 | MN908947.3 | 27626 | 28895 | - | ORF7a;ORF8;ORF7b;N | 4 | find_circ | SRR11550045 |
| SARS-CoV-2_circ_Homo_sapiens_2964 | MN908947.3 | 27685 | 29268 | + | ORF7a;ORF8;ORF7b;N | 4 | circRNA_finder | SRR11550044 |
| SARS-CoV-2_circ_Homo_sapiens_3 | MN908947.3 | 8 | 331 | + | ORF1ab | 4 | find_circ | SRR11550045 |
| SARS-CoV-2_circ_Homo_sapiens_3016 | MN908947.3 | 28321 | 28602 | - | N | 4 | find_circ | SRR11550045 |
| SARS-CoV-2_circ_Homo_sapiens_3027 | MN908947.3 | 28321 | 29066 | - | N | 4 | find_circ | SRR11550044 |
| SARS-CoV-2_circ_Homo_sapiens_3042 | MN908947.3 | 28338 | 29080 | + | N | 4 | find_circ | SRR11550045 |
| SARS-CoV-2_circ_Homo_sapiens_3047 | MN908947.3 | 28404 | 28596 | + | N | 4 | find_circ | SRR11550046 |
| SARS-CoV-2_circ_Homo_sapiens_3047 | MN908947.3 | 28404 | 28596 | + | N | 4 | find_circ | SRR11550045 |
| SARS-CoV-2_circ_Homo_sapiens_3051 | MN908947.3 | 28404 | 28850 | + | N | 4 | circRNA_finder | SRR11550044 |
| SARS-CoV-2_circ_Homo_sapiens_3051 | MN908947.3 | 28404 | 28850 | + | N | 4 | circRNA_finder | SRR11550045 |
| SARS-CoV-2_circ_Homo_sapiens_3051 | MN908947.3 | 28404 | 28850 | + | N | 4 | find_circ | SRR11550043 |
| SARS-CoV-2_circ_Homo_sapiens_3051 | MN908947.3 | 28404 | 28850 | + | N | 4 | find_circ | SRR11550045 |
| SARS-CoV-2_circ_Homo_sapiens_3058 | MN908947.3 | 28404 | 29080 | + | N | 4 | circRNA_finder | SRR11550043 |
| SARS-CoV-2_circ_Homo_sapiens_3058 | MN908947.3 | 28404 | 29080 | + | N | 4 | find_circ | SRR11550043 |
| SARS-CoV-2_circ_Homo_sapiens_3069 | MN908947.3 | 28410 | 28727 | - | N | 4 | find_circ | SRR11550046 |
| SARS-CoV-2_circ_Homo_sapiens_3075 | MN908947.3 | 28410 | 28865 | - | N | 4 | find_circ | SRR11550046 |
| SARS-CoV-2_circ_Homo_sapiens_3083 | MN908947.3 | 28410 | 29012 | + | N | 4 | circRNA_finder | SRR11550045 |
| SARS-CoV-2_circ_Homo_sapiens_3091 | MN908947.3 | 28410 | 29534 | - | N | 4 | find_circ | SRR11550045 |
| SARS-CoV-2_circ_Homo_sapiens_3095 | MN908947.3 | 28420 | 28602 | - | N | 4 | find_circ | SRR11550045 |
| SARS-CoV-2_circ_Homo_sapiens_3101 | MN908947.3 | 28435 | 28727 | - | N | 4 | CIRI2 | SRR11550045 |
| SARS-CoV-2_circ_Homo_sapiens_3111 | MN908947.3 | 28449 | 28895 | - | N | 4 | find_circ | SRR11550046 |
| SARS-CoV-2_circ_Homo_sapiens_3118 | MN908947.3 | 28463 | 28686 | - | N | 4 | CIRI2 | SRR11550043 |
| SARS-CoV-2_circ_Homo_sapiens_3119 | MN908947.3 | 28463 | 28724 | - | N | 4 | CIRI2 | SRR11550045 |
| SARS-CoV-2_circ_Homo_sapiens_312 | MN908947.3 | 1844 | 2421 | + | ORF1ab | 4 | find_circ | SRR11550046 |
| SARS-CoV-2_circ_Homo_sapiens_312 | MN908947.3 | 1844 | 2421 | + | ORF1ab | 4 | circRNA_finder | SRR11550046 |
| SARS-CoV-2_circ_Homo_sapiens_3125 | MN908947.3 | 28463 | 28753 | - | N | 4 | CIRI2 | SRR11550045 |
| SARS-CoV-2_circ_Homo_sapiens_3125 | MN908947.3 | 28463 | 28753 | - | N | 4 | CIRI2 | SRR11550043 |
| SARS-CoV-2_circ_Homo_sapiens_3136 | MN908947.3 | 28463 | 28927 | - | N | 4 | CIRI2 | SRR11550045 |
| SARS-CoV-2_circ_Homo_sapiens_3138 | MN908947.3 | 28463 | 28931 | - | N | 4 | CIRI2 | SRR11550043 |
| SARS-CoV-2_circ_Homo_sapiens_3146 | MN908947.3 | 28463 | 29012 | - | N | 4 | find_circ | SRR11550046 |
| SARS-CoV-2_circ_Homo_sapiens_3164 | MN908947.3 | 28463 | 29607 | - | ORF10;N | 4 | CIRI2 | SRR11550045 |
| SARS-CoV-2_circ_Homo_sapiens_3188 | MN908947.3 | 28607 | 28830 | - | N | 4 | CIRI2 | SRR11550045 |
| SARS-CoV-2_circ_Homo_sapiens_3193 | MN908947.3 | 28607 | 28904 | + | N | 4 | circRNA_finder | SRR11550043 |
| SARS-CoV-2_circ_Homo_sapiens_3210 | MN908947.3 | 28610 | 28942 | - | N | 4 | find_circ | SRR11550044 |
| SARS-CoV-2_circ_Homo_sapiens_3212 | MN908947.3 | 28610 | 29012 | - | N | 4 | find_circ | SRR11550043 |
| SARS-CoV-2_circ_Homo_sapiens_3213 | MN908947.3 | 28610 | 29012 | + | N | 4 | circRNA_finder | SRR11550043 |
| SARS-CoV-2_circ_Homo_sapiens_3218 | MN908947.3 | 28614 | 29008 | + | N | 4 | CIRI2 | SRR11550044 |
| SARS-CoV-2_circ_Homo_sapiens_3232 | MN908947.3 | 28656 | 29276 | + | N | 4 | CIRI2 | SRR11550043 |
| SARS-CoV-2_circ_Homo_sapiens_3232 | MN908947.3 | 28656 | 29276 | + | N | 4 | find_circ | SRR11550043 |
| SARS-CoV-2_circ_Homo_sapiens_3248 | MN908947.3 | 28709 | 28895 | - | N | 4 | find_circ | SRR11550044 |
| SARS-CoV-2_circ_Homo_sapiens_3268 | MN908947.3 | 28771 | 29036 | - | N | 4 | find_circ | SRR11550046 |
| SARS-CoV-2_circ_Homo_sapiens_3269 | MN908947.3 | 28771 | 29066 | - | N | 4 | find_circ | SRR11550044 |
| SARS-CoV-2_circ_Homo_sapiens_3270 | MN908947.3 | 28771 | 29143 | - | N | 4 | find_circ | SRR11550045 |
| SARS-CoV-2_circ_Homo_sapiens_3276 | MN908947.3 | 28790 | 29529 | - | N | 4 | find_circ | SRR11550045 |
| SARS-CoV-2_circ_Homo_sapiens_3300 | MN908947.3 | 29073 | 29572 | + | ORF10;N | 4 | find_circ | SRR11550043 |
| SARS-CoV-2_circ_Homo_sapiens_3301 | MN908947.3 | 29073 | 29650 | + | ORF10;N | 4 | find_circ | SRR11550044 |
| SARS-CoV-2_circ_Homo_sapiens_3309 | MN908947.3 | 29086 | 29329 | - | N | 4 | find_circ | SRR11550044 |
| SARS-CoV-2_circ_Homo_sapiens_3310 | MN908947.3 | 29086 | 29408 | - | N | 4 | find_circ | SRR11550043 |
| SARS-CoV-2_circ_Homo_sapiens_3313 | MN908947.3 | 29086 | 29450 | - | N | 4 | find_circ | SRR11550043 |
| SARS-CoV-2_circ_Homo_sapiens_3321 | MN908947.3 | 29086 | 29665 | + | ORF10;N | 4 | circRNA_finder | SRR11550044 |
| SARS-CoV-2_circ_Homo_sapiens_3321 | MN908947.3 | 29086 | 29665 | + | ORF10;N | 4 | circRNA_finder | SRR11550043 |
| SARS-CoV-2_circ_Homo_sapiens_3324 | MN908947.3 | 29088 | 29529 | - | N | 4 | find_circ | SRR11550045 |
| SARS-CoV-2_circ_Homo_sapiens_3326 | MN908947.3 | 29091 | 29572 | + | ORF10;N | 4 | circRNA_finder | SRR11550044 |
| SARS-CoV-2_circ_Homo_sapiens_3326 | MN908947.3 | 29091 | 29572 | + | ORF10;N | 4 | find_circ | SRR11550044 |
| SARS-CoV-2_circ_Homo_sapiens_3339 | MN908947.3 | 29168 | 29534 | - | N | 4 | find_circ | SRR11550045 |
| SARS-CoV-2_circ_Homo_sapiens_3343 | MN908947.3 | 29172 | 29444 | - | N | 4 | find_circ | SRR11550045 |
| SARS-CoV-2_circ_Homo_sapiens_3343 | MN908947.3 | 29172 | 29444 | - | N | 4 | find_circ | SRR11550046 |
| SARS-CoV-2_circ_Homo_sapiens_3351 | MN908947.3 | 29172 | 29579 | + | ORF10;N | 4 | circRNA_finder | SRR11550046 |
| SARS-CoV-2_circ_Homo_sapiens_3351 | MN908947.3 | 29172 | 29579 | + | ORF10;N | 4 | circRNA_finder | SRR11550043 |
| SARS-CoV-2_circ_Homo_sapiens_3362 | MN908947.3 | 29190 | 29579 | - | ORF10;N | 4 | find_circ | SRR11550046 |
| SARS-CoV-2_circ_Homo_sapiens_3373 | MN908947.3 | 29250 | 29579 | + | ORF10;N | 4 | circRNA_finder | SRR11550046 |
| SARS-CoV-2_circ_Homo_sapiens_3381 | MN908947.3 | 29260 | 29444 | - | N | 4 | find_circ | SRR11550045 |
| SARS-CoV-2_circ_Homo_sapiens_3382 | MN908947.3 | 29260 | 29529 | - | N | 4 | find_circ | SRR11550043 |
| SARS-CoV-2_circ_Homo_sapiens_3382 | MN908947.3 | 29260 | 29529 | - | N | 4 | find_circ | SRR11550045 |
| SARS-CoV-2_circ_Homo_sapiens_3383 | MN908947.3 | 29260 | 29579 | - | ORF10;N | 4 | find_circ | SRR11550045 |
| SARS-CoV-2_circ_Homo_sapiens_3383 | MN908947.3 | 29260 | 29579 | - | ORF10;N | 4 | find_circ | SRR11550043 |
| SARS-CoV-2_circ_Homo_sapiens_3388 | MN908947.3 | 29269 | 29452 | - | N | 4 | CIRI2 | SRR11550046 |
| SARS-CoV-2_circ_Homo_sapiens_3393 | MN908947.3 | 29269 | 29529 | - | N | 4 | find_circ | SRR11550046 |
| SARS-CoV-2_circ_Homo_sapiens_3394 | MN908947.3 | 29269 | 29529 | + | N | 4 | circRNA_finder | SRR11550046 |
| SARS-CoV-2_circ_Homo_sapiens_3397 | MN908947.3 | 29269 | 29562 | - | ORF10;N | 4 | CIRI2 | SRR11550044 |
| SARS-CoV-2_circ_Homo_sapiens_3399 | MN908947.3 | 29269 | 29579 | - | ORF10;N | 4 | find_circ | SRR11550044 |
| SARS-CoV-2_circ_Homo_sapiens_3399 | MN908947.3 | 29269 | 29579 | - | ORF10;N | 4 | find_circ | SRR11550046 |
| SARS-CoV-2_circ_Homo_sapiens_3400 | MN908947.3 | 29269 | 29579 | + | ORF10;N | 4 | circRNA_finder | SRR11550044 |
| SARS-CoV-2_circ_Homo_sapiens_3412 | MN908947.3 | 29316 | 29610 | + | ORF10;N | 4 | circRNA_finder | SRR11550045 |
| SARS-CoV-2_circ_Homo_sapiens_3429 | MN908947.3 | 29410 | 29665 | - | ORF10;N | 4 | find_circ | SRR11550043 |
| SARS-CoV-2_circ_Homo_sapiens_3436 | MN908947.3 | 29671 | 29787 | - | ORF10 | 4 | find_circ | SRR11550043 |
| SARS-CoV-2_circ_Homo_sapiens_347 | MN908947.3 | 2495 | 3353 | + | ORF1ab | 4 | circRNA_finder | SRR11550046 |
| SARS-CoV-2_circ_Homo_sapiens_354 | MN908947.3 | 2559 | 3737 | - | ORF1ab | 4 | find_circ | SRR11550045 |
| SARS-CoV-2_circ_Homo_sapiens_357 | MN908947.3 | 2565 | 3024 | + | ORF1ab | 4 | find_circ | SRR11550045 |
| SARS-CoV-2_circ_Homo_sapiens_357 | MN908947.3 | 2565 | 3024 | + | ORF1ab | 4 | circRNA_finder | SRR11550045 |
| SARS-CoV-2_circ_Homo_sapiens_363 | MN908947.3 | 2571 | 3263 | - | ORF1ab | 4 | find_circ | SRR11550045 |
| SARS-CoV-2_circ_Homo_sapiens_374 | MN908947.3 | 2578 | 3024 | + | ORF1ab | 4 | find_circ | SRR11550046 |
| SARS-CoV-2_circ_Homo_sapiens_377 | MN908947.3 | 2592 | 3029 | + | ORF1ab | 4 | find_circ | SRR11550046 |
| SARS-CoV-2_circ_Homo_sapiens_379 | MN908947.3 | 2592 | 3337 | + | ORF1ab | 4 | find_circ | SRR11550046 |
| SARS-CoV-2_circ_Homo_sapiens_38 | MN908947.3 | 16 | 449 | - | ORF1ab | 4 | find_circ | SRR11550044 |
| SARS-CoV-2_circ_Homo_sapiens_4 | MN908947.3 | 8 | 430 | + | ORF1ab | 4 | find_circ | SRR11550044 |
| SARS-CoV-2_circ_Homo_sapiens_419 | MN908947.3 | 3060 | 3215 | + | ORF1ab | 4 | find_circ | SRR11550046 |
| SARS-CoV-2_circ_Homo_sapiens_458 | MN908947.3 | 3540 | 4806 | + | ORF1ab | 4 | find_circ | SRR11550046 |
| SARS-CoV-2_circ_Homo_sapiens_462 | MN908947.3 | 3552 | 4886 | - | ORF1ab | 4 | find_circ | SRR11550043 |
| SARS-CoV-2_circ_Homo_sapiens_487 | MN908947.3 | 3662 | 7628 | + | ORF1ab | 4 | find_circ | SRR11550046 |
| SARS-CoV-2_circ_Homo_sapiens_514 | MN908947.3 | 4040 | 4448 | - | ORF1ab | 4 | find_circ | SRR11550044 |
| SARS-CoV-2_circ_Homo_sapiens_530 | MN908947.3 | 4284 | 4835 | + | ORF1ab | 4 | CIRI2 | SRR11550045 |
| SARS-CoV-2_circ_Homo_sapiens_574 | MN908947.3 | 4597 | 5564 | - | ORF1ab | 4 | find_circ | SRR11550045 |
| SARS-CoV-2_circ_Homo_sapiens_575 | MN908947.3 | 4597 | 7712 | - | ORF1ab | 4 | find_circ | SRR11550043 |
| SARS-CoV-2_circ_Homo_sapiens_576 | MN908947.3 | 4597 | 7712 | + | ORF1ab | 4 | circRNA_finder | SRR11550043 |
| SARS-CoV-2_circ_Homo_sapiens_585 | MN908947.3 | 4836 | 5331 | + | ORF1ab | 4 | circRNA_finder | SRR11550043 |
| SARS-CoV-2_circ_Homo_sapiens_585 | MN908947.3 | 4836 | 5331 | + | ORF1ab | 4 | find_circ | SRR11550043 |
| SARS-CoV-2_circ_Homo_sapiens_589 | MN908947.3 | 4836 | 5557 | + | ORF1ab | 4 | find_circ | SRR11550045 |
| SARS-CoV-2_circ_Homo_sapiens_649 | MN908947.3 | 5116 | 5816 | - | ORF1ab | 4 | find_circ | SRR11550045 |
| SARS-CoV-2_circ_Homo_sapiens_68 | MN908947.3 | 37 | 26200 | - | ORF3a;S;ORF1ab | 4 | find_circ | SRR11550046 |
| SARS-CoV-2_circ_Homo_sapiens_699 | MN908947.3 | 5527 | 5821 | - | ORF1ab | 4 | find_circ | SRR11550045 |
| SARS-CoV-2_circ_Homo_sapiens_74 | MN908947.3 | 41 | 682 | - | ORF1ab | 4 | find_circ | SRR11550045 |
| SARS-CoV-2_circ_Homo_sapiens_747 | MN908947.3 | 5827 | 6577 | - | ORF1ab | 4 | find_circ | SRR11550045 |
| SARS-CoV-2_circ_Homo_sapiens_748 | MN908947.3 | 5827 | 6605 | - | ORF1ab | 4 | find_circ | SRR11550044 |
| SARS-CoV-2_circ_Homo_sapiens_749 | MN908947.3 | 5827 | 6605 | + | ORF1ab | 4 | circRNA_finder | SRR11550044 |
| SARS-CoV-2_circ_Homo_sapiens_75 | MN908947.3 | 41 | 682 | + | ORF1ab | 4 | circRNA_finder | SRR11550045 |
| SARS-CoV-2_circ_Homo_sapiens_766 | MN908947.3 | 5908 | 6680 | - | ORF1ab | 4 | find_circ | SRR11550046 |
| SARS-CoV-2_circ_Homo_sapiens_766 | MN908947.3 | 5908 | 6680 | - | ORF1ab | 4 | find_circ | SRR11550045 |
| SARS-CoV-2_circ_Homo_sapiens_767 | MN908947.3 | 5908 | 6680 | + | ORF1ab | 4 | circRNA_finder | SRR11550046 |
| SARS-CoV-2_circ_Homo_sapiens_767 | MN908947.3 | 5908 | 6680 | + | ORF1ab | 4 | circRNA_finder | SRR11550045 |
| SARS-CoV-2_circ_Homo_sapiens_770 | MN908947.3 | 5908 | 7028 | - | ORF1ab | 4 | find_circ | SRR11550045 |
| SARS-CoV-2_circ_Homo_sapiens_771 | MN908947.3 | 5908 | 7028 | + | ORF1ab | 4 | circRNA_finder | SRR11550045 |
| SARS-CoV-2_circ_Homo_sapiens_786 | MN908947.3 | 6021 | 6557 | + | ORF1ab | 4 | circRNA_finder | SRR11550046 |
| SARS-CoV-2_circ_Homo_sapiens_82 | MN908947.3 | 41 | 17569 | - | ORF1ab | 4 | find_circ | SRR11550045 |
| SARS-CoV-2_circ_Homo_sapiens_855 | MN908947.3 | 8293 | 8654 | - | ORF1ab | 4 | CIRI2 | SRR11550046 |
| SARS-CoV-2_circ_Homo_sapiens_859 | MN908947.3 | 8293 | 8916 | - | ORF1ab | 4 | find_circ | SRR11550045 |
| SARS-CoV-2_circ_Homo_sapiens_860 | MN908947.3 | 8293 | 8916 | + | ORF1ab | 4 | circRNA_finder | SRR11550045 |
| SARS-CoV-2_circ_Homo_sapiens_880 | MN908947.3 | 8300 | 8921 | - | ORF1ab | 4 | find_circ | SRR11550045 |
| SARS-CoV-2_circ_Homo_sapiens_881 | MN908947.3 | 8300 | 8921 | + | ORF1ab | 4 | circRNA_finder | SRR11550045 |
| SARS-CoV-2_circ_Homo_sapiens_91 | MN908947.3 | 72 | 449 | - | ORF1ab | 4 | find_circ | SRR11550046 |
| SARS-CoV-2_circ_Homo_sapiens_930 | MN908947.3 | 8926 | 9553 | + | ORF1ab | 4 | find_circ | SRR11550045 |
| SARS-CoV-2_circ_Homo_sapiens_937 | MN908947.3 | 8948 | 29534 | - | ORF6;M;E;ORF3a;S;N;ORF8;ORF7a;ORF7b;ORF1ab | 4 | find_circ | SRR11550044 |
| SARS-CoV-2_circ_Homo_sapiens_954 | MN908947.3 | 9170 | 11194 | - | ORF1ab | 4 | CIRI2 | SRR11550046 |
| SARS-CoV-2_circ_Homo_sapiens_958 | MN908947.3 | 9177 | 10097 | + | ORF1ab | 4 | find_circ | SRR11550043 |
| SARS-CoV-2_circ_Homo_sapiens_97 | MN908947.3 | 237 | 442 | + | ORF1ab | 4 | circRNA_finder | SRR11550046 |
| SARS-CoV-2_circ_Homo_sapiens_975 | MN908947.3 | 9639 | 10031 | - | ORF1ab | 4 | find_circ | SRR11550045 |
| SARS-CoV-2_circ_Homo_sapiens_10 | MN908947.3 | 8 | 1360 | + | ORF1ab | 3 | find_circ | SRR11550045 |
| SARS-CoV-2_circ_Homo_sapiens_1006 | MN908947.3 | 9948 | 10487 | + | ORF1ab | 3 | find_circ | SRR11550044 |
| SARS-CoV-2_circ_Homo_sapiens_1006 | MN908947.3 | 9948 | 10487 | + | ORF1ab | 3 | circRNA_finder | SRR11550044 |
| SARS-CoV-2_circ_Homo_sapiens_1029 | MN908947.3 | 10055 | 10495 | + | ORF1ab | 3 | find_circ | SRR11550044 |
| SARS-CoV-2_circ_Homo_sapiens_1030 | MN908947.3 | 10066 | 10353 | + | ORF1ab | 3 | find_circ | SRR11550045 |
| SARS-CoV-2_circ_Homo_sapiens_1030 | MN908947.3 | 10066 | 10353 | + | ORF1ab | 3 | circRNA_finder | SRR11550045 |
| SARS-CoV-2_circ_Homo_sapiens_1033 | MN908947.3 | 10066 | 10663 | + | ORF1ab | 3 | find_circ | SRR11550044 |
| SARS-CoV-2_circ_Homo_sapiens_1073 | MN908947.3 | 10401 | 12105 | + | ORF1ab | 3 | find_circ | SRR11550043 |
| SARS-CoV-2_circ_Homo_sapiens_1075 | MN908947.3 | 10512 | 11482 | + | ORF1ab | 3 | find_circ | SRR11550044 |
| SARS-CoV-2_circ_Homo_sapiens_108 | MN908947.3 | 312 | 1008 | - | ORF1ab | 3 | find_circ | SRR11550045 |
| SARS-CoV-2_circ_Homo_sapiens_1091 | MN908947.3 | 10605 | 10931 | - | ORF1ab | 3 | find_circ | SRR11550045 |
| SARS-CoV-2_circ_Homo_sapiens_1113 | MN908947.3 | 10659 | 11082 | + | ORF1ab | 3 | circRNA_finder | SRR11550045 |
| SARS-CoV-2_circ_Homo_sapiens_1116 | MN908947.3 | 10659 | 11482 | + | ORF1ab | 3 | find_circ | SRR11550043 |
| SARS-CoV-2_circ_Homo_sapiens_1116 | MN908947.3 | 10659 | 11482 | + | ORF1ab | 3 | find_circ | SRR11550045 |
| SARS-CoV-2_circ_Homo_sapiens_112 | MN908947.3 | 312 | 28895 | - | ORF6;M;E;ORF3a;S;N;ORF8;ORF7a;ORF7b;ORF1ab | 3 | find_circ | SRR11550045 |
| SARS-CoV-2_circ_Homo_sapiens_1125 | MN908947.3 | 10659 | 27203 | + | ORF6;M;E;ORF3a;S;ORF1ab | 3 | find_circ | SRR11550045 |
| SARS-CoV-2_circ_Homo_sapiens_1128 | MN908947.3 | 10671 | 10939 | + | ORF1ab | 3 | find_circ | SRR11550046 |
| SARS-CoV-2_circ_Homo_sapiens_1132 | MN908947.3 | 10671 | 11482 | + | ORF1ab | 3 | find_circ | SRR11550043 |
| SARS-CoV-2_circ_Homo_sapiens_1157 | MN908947.3 | 10948 | 24925 | + | S;ORF1ab | 3 | find_circ | SRR11550046 |
| SARS-CoV-2_circ_Homo_sapiens_1157 | MN908947.3 | 10948 | 24925 | + | S;ORF1ab | 3 | circRNA_finder | SRR11550046 |
| SARS-CoV-2_circ_Homo_sapiens_1173 | MN908947.3 | 11284 | 11482 | + | ORF1ab | 3 | find_circ | SRR11550045 |
| SARS-CoV-2_circ_Homo_sapiens_1217 | MN908947.3 | 11515 | 12092 | + | ORF1ab | 3 | circRNA_finder | SRR11550043 |
| SARS-CoV-2_circ_Homo_sapiens_1219 | MN908947.3 | 11638 | 11816 | - | ORF1ab | 3 | find_circ | SRR11550043 |
| SARS-CoV-2_circ_Homo_sapiens_1220 | MN908947.3 | 11638 | 11918 | - | ORF1ab | 3 | find_circ | SRR11550044 |
| SARS-CoV-2_circ_Homo_sapiens_1243 | MN908947.3 | 11645 | 14177 | - | ORF1ab | 3 | CIRI2 | SRR11550043 |
| SARS-CoV-2_circ_Homo_sapiens_1246 | MN908947.3 | 11664 | 11975 | - | ORF1ab | 3 | CIRI2 | SRR11550045 |
| SARS-CoV-2_circ_Homo_sapiens_1248 | MN908947.3 | 11675 | 18813 | - | ORF1ab | 3 | find_circ | SRR11550046 |
| SARS-CoV-2_circ_Homo_sapiens_13 | MN908947.3 | 8 | 3760 | + | ORF1ab | 3 | find_circ | SRR11550043 |
| SARS-CoV-2_circ_Homo_sapiens_132 | MN908947.3 | 527 | 1244 | + | ORF1ab | 3 | find_circ | SRR11550045 |
| SARS-CoV-2_circ_Homo_sapiens_1341 | MN908947.3 | 12142 | 12777 | - | ORF1ab | 3 | find_circ | SRR11550046 |
| SARS-CoV-2_circ_Homo_sapiens_1344 | MN908947.3 | 12153 | 12646 | + | ORF1ab | 3 | find_circ | SRR11550043 |
| SARS-CoV-2_circ_Homo_sapiens_1345 | MN908947.3 | 12153 | 12768 | + | ORF1ab | 3 | circRNA_finder | SRR11550045 |
| SARS-CoV-2_circ_Homo_sapiens_1345 | MN908947.3 | 12153 | 12768 | + | ORF1ab | 3 | find_circ | SRR11550045 |
| SARS-CoV-2_circ_Homo_sapiens_1345 | MN908947.3 | 12153 | 12768 | + | ORF1ab | 3 | circRNA_finder | SRR11550044 |
| SARS-CoV-2_circ_Homo_sapiens_1351 | MN908947.3 | 12153 | 13385 | + | ORF1ab | 3 | find_circ | SRR11550043 |
| SARS-CoV-2_circ_Homo_sapiens_1356 | MN908947.3 | 12153 | 15108 | + | ORF1ab | 3 | find_circ | SRR11550046 |
| SARS-CoV-2_circ_Homo_sapiens_1356 | MN908947.3 | 12153 | 15108 | + | ORF1ab | 3 | find_circ | SRR11550045 |
| SARS-CoV-2_circ_Homo_sapiens_1357 | MN908947.3 | 12153 | 15242 | + | ORF1ab | 3 | find_circ | SRR11550044 |
| SARS-CoV-2_circ_Homo_sapiens_1357 | MN908947.3 | 12153 | 15242 | + | ORF1ab | 3 | circRNA_finder | SRR11550044 |
| SARS-CoV-2_circ_Homo_sapiens_1361 | MN908947.3 | 12164 | 13409 | + | ORF1ab | 3 | find_circ | SRR11550045 |
| SARS-CoV-2_circ_Homo_sapiens_1374 | MN908947.3 | 12261 | 12808 | - | ORF1ab | 3 | find_circ | SRR11550046 |
| SARS-CoV-2_circ_Homo_sapiens_1376 | MN908947.3 | 12261 | 14759 | - | ORF1ab | 3 | find_circ | SRR11550046 |
| SARS-CoV-2_circ_Homo_sapiens_1381 | MN908947.3 | 12288 | 12646 | + | ORF1ab | 3 | find_circ | SRR11550044 |
| SARS-CoV-2_circ_Homo_sapiens_1385 | MN908947.3 | 12288 | 15149 | + | ORF1ab | 3 | find_circ | SRR11550045 |
| SARS-CoV-2_circ_Homo_sapiens_1388 | MN908947.3 | 12295 | 13334 | - | ORF1ab | 3 | find_circ | SRR11550045 |
| SARS-CoV-2_circ_Homo_sapiens_1389 | MN908947.3 | 12295 | 13334 | + | ORF1ab | 3 | circRNA_finder | SRR11550045 |
| SARS-CoV-2_circ_Homo_sapiens_1399 | MN908947.3 | 12343 | 12773 | - | ORF1ab | 3 | find_circ | SRR11550046 |
| SARS-CoV-2_circ_Homo_sapiens_1412 | MN908947.3 | 12624 | 13139 | - | ORF1ab | 3 | find_circ | SRR11550046 |
| SARS-CoV-2_circ_Homo_sapiens_1413 | MN908947.3 | 12624 | 13139 | + | ORF1ab | 3 | circRNA_finder | SRR11550046 |
| SARS-CoV-2_circ_Homo_sapiens_1423 | MN908947.3 | 12774 | 13389 | + | ORF1ab | 3 | find_circ | SRR11550046 |
| SARS-CoV-2_circ_Homo_sapiens_1428 | MN908947.3 | 12831 | 14177 | - | ORF1ab | 3 | CIRI2 | SRR11550046 |
| SARS-CoV-2_circ_Homo_sapiens_1436 | MN908947.3 | 12833 | 13349 | + | ORF1ab | 3 | find_circ | SRR11550045 |
| SARS-CoV-2_circ_Homo_sapiens_1436 | MN908947.3 | 12833 | 13349 | + | ORF1ab | 3 | circRNA_finder | SRR11550045 |
| SARS-CoV-2_circ_Homo_sapiens_1447 | MN908947.3 | 12924 | 14702 | - | ORF1ab | 3 | find_circ | SRR11550045 |
| SARS-CoV-2_circ_Homo_sapiens_1448 | MN908947.3 | 12924 | 14702 | + | ORF1ab | 3 | circRNA_finder | SRR11550045 |
| SARS-CoV-2_circ_Homo_sapiens_1461 | MN908947.3 | 12974 | 14598 | - | ORF1ab | 3 | find_circ | SRR11550045 |
| SARS-CoV-2_circ_Homo_sapiens_1463 | MN908947.3 | 12974 | 14847 | - | ORF1ab | 3 | CIRI2 | SRR11550046 |
| SARS-CoV-2_circ_Homo_sapiens_1471 | MN908947.3 | 12984 | 13409 | + | ORF1ab | 3 | find_circ | SRR11550046 |
| SARS-CoV-2_circ_Homo_sapiens_1482 | MN908947.3 | 13005 | 13389 | + | ORF1ab | 3 | circRNA_finder | SRR11550046 |
| SARS-CoV-2_circ_Homo_sapiens_1482 | MN908947.3 | 13005 | 13389 | + | ORF1ab | 3 | find_circ | SRR11550046 |
| SARS-CoV-2_circ_Homo_sapiens_1498 | MN908947.3 | 13154 | 13458 | + | ORF1ab | 3 | find_circ | SRR11550045 |
| SARS-CoV-2_circ_Homo_sapiens_150 | MN908947.3 | 689 | 1157 | + | ORF1ab | 3 | find_circ | SRR11550045 |
| SARS-CoV-2_circ_Homo_sapiens_1504 | MN908947.3 | 13265 | 14575 | - | ORF1ab | 3 | find_circ | SRR11550045 |
| SARS-CoV-2_circ_Homo_sapiens_1505 | MN908947.3 | 13265 | 14575 | + | ORF1ab | 3 | circRNA_finder | SRR11550045 |
| SARS-CoV-2_circ_Homo_sapiens_1512 | MN908947.3 | 13469 | 18994 | - | ORF1ab | 3 | find_circ | SRR11550043 |
| SARS-CoV-2_circ_Homo_sapiens_1535 | MN908947.3 | 13521 | 13935 | + | ORF1ab | 3 | find_circ | SRR11550046 |
| SARS-CoV-2_circ_Homo_sapiens_1537 | MN908947.3 | 13521 | 14122 | + | ORF1ab | 3 | CIRI2 | SRR11550044 |
| SARS-CoV-2_circ_Homo_sapiens_1550 | MN908947.3 | 13736 | 14283 | - | ORF1ab | 3 | find_circ | SRR11550044 |
| SARS-CoV-2_circ_Homo_sapiens_1555 | MN908947.3 | 13940 | 14539 | + | ORF1ab | 3 | circRNA_finder | SRR11550044 |
| SARS-CoV-2_circ_Homo_sapiens_1555 | MN908947.3 | 13940 | 14539 | + | ORF1ab | 3 | find_circ | SRR11550044 |
| SARS-CoV-2_circ_Homo_sapiens_1564 | MN908947.3 | 13972 | 14644 | - | ORF1ab | 3 | find_circ | SRR11550045 |
| SARS-CoV-2_circ_Homo_sapiens_1569 | MN908947.3 | 13976 | 14539 | + | ORF1ab | 3 | circRNA_finder | SRR11550046 |
| SARS-CoV-2_circ_Homo_sapiens_1574 | MN908947.3 | 14178 | 14535 | - | ORF1ab | 3 | find_circ | SRR11550045 |
| SARS-CoV-2_circ_Homo_sapiens_1575 | MN908947.3 | 14178 | 14535 | + | ORF1ab | 3 | circRNA_finder | SRR11550045 |
| SARS-CoV-2_circ_Homo_sapiens_1575 | MN908947.3 | 14178 | 14535 | + | ORF1ab | 3 | circRNA_finder | SRR11550046 |
| SARS-CoV-2_circ_Homo_sapiens_1577 | MN908947.3 | 14178 | 14572 | - | ORF1ab | 3 | find_circ | SRR11550044 |
| SARS-CoV-2_circ_Homo_sapiens_1578 | MN908947.3 | 14178 | 14572 | + | ORF1ab | 3 | circRNA_finder | SRR11550044 |
| SARS-CoV-2_circ_Homo_sapiens_1579 | MN908947.3 | 14178 | 14575 | - | ORF1ab | 3 | find_circ | SRR11550043 |
| SARS-CoV-2_circ_Homo_sapiens_1580 | MN908947.3 | 14178 | 14575 | + | ORF1ab | 3 | circRNA_finder | SRR11550043 |
| SARS-CoV-2_circ_Homo_sapiens_1584 | MN908947.3 | 14178 | 14598 | - | ORF1ab | 3 | find_circ | SRR11550046 |
| SARS-CoV-2_circ_Homo_sapiens_1609 | MN908947.3 | 14236 | 14572 | - | ORF1ab | 3 | find_circ | SRR11550045 |
| SARS-CoV-2_circ_Homo_sapiens_1611 | MN908947.3 | 14263 | 14535 | - | ORF1ab | 3 | find_circ | SRR11550046 |
| SARS-CoV-2_circ_Homo_sapiens_1612 | MN908947.3 | 14263 | 14535 | + | ORF1ab | 3 | circRNA_finder | SRR11550046 |
| SARS-CoV-2_circ_Homo_sapiens_1616 | MN908947.3 | 14408 | 15196 | + | ORF1ab | 3 | circRNA_finder | SRR11550043 |
| SARS-CoV-2_circ_Homo_sapiens_1631 | MN908947.3 | 14609 | 15198 | + | ORF1ab | 3 | find_circ | SRR11550043 |
| SARS-CoV-2_circ_Homo_sapiens_1632 | MN908947.3 | 14609 | 15201 | + | ORF1ab | 3 | find_circ | SRR11550046 |
| SARS-CoV-2_circ_Homo_sapiens_1632 | MN908947.3 | 14609 | 15201 | + | ORF1ab | 3 | circRNA_finder | SRR11550046 |
| SARS-CoV-2_circ_Homo_sapiens_1636 | MN908947.3 | 14609 | 18658 | + | ORF1ab | 3 | find_circ | SRR11550046 |
| SARS-CoV-2_circ_Homo_sapiens_166 | MN908947.3 | 698 | 1313 | - | ORF1ab | 3 | find_circ | SRR11550045 |
| SARS-CoV-2_circ_Homo_sapiens_1661 | MN908947.3 | 15038 | 15279 | - | ORF1ab | 3 | find_circ | SRR11550046 |
| SARS-CoV-2_circ_Homo_sapiens_1678 | MN908947.3 | 15269 | 16699 | + | ORF1ab | 3 | find_circ | SRR11550045 |
| SARS-CoV-2_circ_Homo_sapiens_1683 | MN908947.3 | 15280 | 16761 | - | ORF1ab | 3 | CIRI2 | SRR11550045 |
| SARS-CoV-2_circ_Homo_sapiens_1688 | MN908947.3 | 15325 | 15852 | - | ORF1ab | 3 | CIRI2 | SRR11550045 |
| SARS-CoV-2_circ_Homo_sapiens_1694 | MN908947.3 | 15333 | 16297 | + | ORF1ab | 3 | find_circ | SRR11550046 |
| SARS-CoV-2_circ_Homo_sapiens_1694 | MN908947.3 | 15333 | 16297 | + | ORF1ab | 3 | circRNA_finder | SRR11550046 |
| SARS-CoV-2_circ_Homo_sapiens_1694 | MN908947.3 | 15333 | 16297 | + | ORF1ab | 3 | find_circ | SRR11550045 |
| SARS-CoV-2_circ_Homo_sapiens_1697 | MN908947.3 | 15333 | 16486 | + | ORF1ab | 3 | find_circ | SRR11550045 |
| SARS-CoV-2_circ_Homo_sapiens_17 | MN908947.3 | 8 | 6712 | + | ORF1ab | 3 | CIRI2 | SRR11550044 |
| SARS-CoV-2_circ_Homo_sapiens_1704 | MN908947.3 | 15402 | 15726 | + | ORF1ab | 3 | circRNA_finder | SRR11550045 |
| SARS-CoV-2_circ_Homo_sapiens_1704 | MN908947.3 | 15402 | 15726 | + | ORF1ab | 3 | find_circ | SRR11550045 |
| SARS-CoV-2_circ_Homo_sapiens_1707 | MN908947.3 | 15402 | 15733 | + | ORF1ab | 3 | find_circ | SRR11550045 |
| SARS-CoV-2_circ_Homo_sapiens_1708 | MN908947.3 | 15402 | 15788 | + | ORF1ab | 3 | circRNA_finder | SRR11550046 |
| SARS-CoV-2_circ_Homo_sapiens_1708 | MN908947.3 | 15402 | 15788 | + | ORF1ab | 3 | find_circ | SRR11550046 |
| SARS-CoV-2_circ_Homo_sapiens_1734 | MN908947.3 | 15747 | 17761 | - | ORF1ab | 3 | find_circ | SRR11550046 |
| SARS-CoV-2_circ_Homo_sapiens_1745 | MN908947.3 | 15853 | 16427 | - | ORF1ab | 3 | find_circ | SRR11550046 |
| SARS-CoV-2_circ_Homo_sapiens_1765 | MN908947.3 | 16333 | 17413 | - | ORF1ab | 3 | find_circ | SRR11550045 |
| SARS-CoV-2_circ_Homo_sapiens_1766 | MN908947.3 | 16352 | 16728 | - | ORF1ab | 3 | find_circ | SRR11550044 |
| SARS-CoV-2_circ_Homo_sapiens_1789 | MN908947.3 | 16552 | 17001 | + | ORF1ab | 3 | circRNA_finder | SRR11550044 |
| SARS-CoV-2_circ_Homo_sapiens_1820 | MN908947.3 | 16944 | 17410 | + | ORF1ab | 3 | circRNA_finder | SRR11550045 |
| SARS-CoV-2_circ_Homo_sapiens_1820 | MN908947.3 | 16944 | 17410 | + | ORF1ab | 3 | find_circ | SRR11550045 |
| SARS-CoV-2_circ_Homo_sapiens_1822 | MN908947.3 | 16972 | 17554 | - | ORF1ab | 3 | find_circ | SRR11550045 |
| SARS-CoV-2_circ_Homo_sapiens_1824 | MN908947.3 | 16996 | 18355 | - | ORF1ab | 3 | CIRI2 | SRR11550045 |
| SARS-CoV-2_circ_Homo_sapiens_1830 | MN908947.3 | 17219 | 18016 | + | ORF1ab | 3 | circRNA_finder | SRR11550045 |
| SARS-CoV-2_circ_Homo_sapiens_1830 | MN908947.3 | 17219 | 18016 | + | ORF1ab | 3 | find_circ | SRR11550045 |
| SARS-CoV-2_circ_Homo_sapiens_1832 | MN908947.3 | 17342 | 18016 | + | ORF1ab | 3 | circRNA_finder | SRR11550046 |
| SARS-CoV-2_circ_Homo_sapiens_1832 | MN908947.3 | 17342 | 18016 | + | ORF1ab | 3 | find_circ | SRR11550046 |
| SARS-CoV-2_circ_Homo_sapiens_1834 | MN908947.3 | 17342 | 18874 | + | ORF1ab | 3 | find_circ | SRR11550043 |
| SARS-CoV-2_circ_Homo_sapiens_1838 | MN908947.3 | 17406 | 17740 | + | ORF1ab | 3 | find_circ | SRR11550045 |
| SARS-CoV-2_circ_Homo_sapiens_1851 | MN908947.3 | 17411 | 18355 | - | ORF1ab | 3 | CIRI2 | SRR11550043 |
| SARS-CoV-2_circ_Homo_sapiens_186 | MN908947.3 | 732 | 2920 | + | ORF1ab | 3 | find_circ | SRR11550043 |
| SARS-CoV-2_circ_Homo_sapiens_1879 | MN908947.3 | 17886 | 18355 | - | ORF1ab | 3 | find_circ | SRR11550045 |
| SARS-CoV-2_circ_Homo_sapiens_1880 | MN908947.3 | 17886 | 18355 | + | ORF1ab | 3 | circRNA_finder | SRR11550045 |
| SARS-CoV-2_circ_Homo_sapiens_1892 | MN908947.3 | 18017 | 18421 | - | ORF1ab | 3 | find_circ | SRR11550046 |
| SARS-CoV-2_circ_Homo_sapiens_1893 | MN908947.3 | 18017 | 18421 | + | ORF1ab | 3 | circRNA_finder | SRR11550046 |
| SARS-CoV-2_circ_Homo_sapiens_1895 | MN908947.3 | 18017 | 18492 | - | ORF1ab | 3 | find_circ | SRR11550046 |
| SARS-CoV-2_circ_Homo_sapiens_1900 | MN908947.3 | 18017 | 18813 | - | ORF1ab | 3 | find_circ | SRR11550044 |
| SARS-CoV-2_circ_Homo_sapiens_1901 | MN908947.3 | 18017 | 18876 | - | ORF1ab | 3 | find_circ | SRR11550044 |
| SARS-CoV-2_circ_Homo_sapiens_1931 | MN908947.3 | 18038 | 18355 | - | ORF1ab | 3 | find_circ | SRR11550043 |
| SARS-CoV-2_circ_Homo_sapiens_1931 | MN908947.3 | 18038 | 18355 | - | ORF1ab | 3 | CIRI2 | SRR11550043 |
| SARS-CoV-2_circ_Homo_sapiens_1947 | MN908947.3 | 18275 | 18874 | + | ORF1ab | 3 | find_circ | SRR11550043 |
| SARS-CoV-2_circ_Homo_sapiens_195 | MN908947.3 | 873 | 1244 | + | ORF1ab | 3 | find_circ | SRR11550045 |
| SARS-CoV-2_circ_Homo_sapiens_1950 | MN908947.3 | 18282 | 18820 | + | ORF1ab | 3 | find_circ | SRR11550046 |
| SARS-CoV-2_circ_Homo_sapiens_1951 | MN908947.3 | 18282 | 23485 | + | S;ORF1ab | 3 | circRNA_finder | SRR11550045 |
| SARS-CoV-2_circ_Homo_sapiens_1951 | MN908947.3 | 18282 | 23485 | + | S;ORF1ab | 3 | find_circ | SRR11550045 |
| SARS-CoV-2_circ_Homo_sapiens_1955 | MN908947.3 | 18356 | 18876 | - | ORF1ab | 3 | find_circ | SRR11550044 |
| SARS-CoV-2_circ_Homo_sapiens_1956 | MN908947.3 | 18356 | 18876 | + | ORF1ab | 3 | circRNA_finder | SRR11550044 |
| SARS-CoV-2_circ_Homo_sapiens_1982 | MN908947.3 | 18494 | 18876 | - | ORF1ab | 3 | find_circ | SRR11550045 |
| SARS-CoV-2_circ_Homo_sapiens_1983 | MN908947.3 | 18494 | 18876 | + | ORF1ab | 3 | circRNA_finder | SRR11550045 |
| SARS-CoV-2_circ_Homo_sapiens_1993 | MN908947.3 | 18602 | 26541 | - | M;E;ORF3a;S;ORF1ab | 3 | find_circ | SRR11550046 |
| SARS-CoV-2_circ_Homo_sapiens_2000 | MN908947.3 | 18657 | 19342 | - | ORF1ab | 3 | find_circ | SRR11550045 |
| SARS-CoV-2_circ_Homo_sapiens_2001 | MN908947.3 | 18657 | 19342 | + | ORF1ab | 3 | circRNA_finder | SRR11550045 |
| SARS-CoV-2_circ_Homo_sapiens_2016 | MN908947.3 | 18873 | 19291 | + | ORF1ab | 3 | find_circ | SRR11550046 |
| SARS-CoV-2_circ_Homo_sapiens_2017 | MN908947.3 | 18881 | 19299 | + | ORF1ab | 3 | find_circ | SRR11550046 |
| SARS-CoV-2_circ_Homo_sapiens_2024 | MN908947.3 | 18913 | 19185 | - | ORF1ab | 3 | find_circ | SRR11550046 |
| SARS-CoV-2_circ_Homo_sapiens_2037 | MN908947.3 | 19033 | 19549 | - | ORF1ab | 3 | find_circ | SRR11550043 |
| SARS-CoV-2_circ_Homo_sapiens_204 | MN908947.3 | 897 | 1244 | + | ORF1ab | 3 | find_circ | SRR11550044 |
| SARS-CoV-2_circ_Homo_sapiens_2050 | MN908947.3 | 19603 | 19982 | - | ORF1ab | 3 | find_circ | SRR11550043 |
| SARS-CoV-2_circ_Homo_sapiens_2050 | MN908947.3 | 19603 | 19982 | - | ORF1ab | 3 | find_circ | SRR11550045 |
| SARS-CoV-2_circ_Homo_sapiens_2051 | MN908947.3 | 19603 | 20198 | - | ORF1ab | 3 | find_circ | SRR11550043 |
| SARS-CoV-2_circ_Homo_sapiens_2053 | MN908947.3 | 19605 | 19982 | - | ORF1ab | 3 | find_circ | SRR11550043 |
| SARS-CoV-2_circ_Homo_sapiens_2055 | MN908947.3 | 19605 | 20098 | - | ORF1ab | 3 | find_circ | SRR11550045 |
| SARS-CoV-2_circ_Homo_sapiens_2059 | MN908947.3 | 19605 | 20198 | + | ORF1ab | 3 | circRNA_finder | SRR11550045 |
| SARS-CoV-2_circ_Homo_sapiens_2063 | MN908947.3 | 19611 | 20015 | - | ORF1ab | 3 | find_circ | SRR11550044 |
| SARS-CoV-2_circ_Homo_sapiens_2065 | MN908947.3 | 19611 | 20203 | - | ORF1ab | 3 | find_circ | SRR11550045 |
| SARS-CoV-2_circ_Homo_sapiens_2066 | MN908947.3 | 19611 | 20203 | + | ORF1ab | 3 | circRNA_finder | SRR11550044 |
| SARS-CoV-2_circ_Homo_sapiens_2069 | MN908947.3 | 19661 | 19982 | - | ORF1ab | 3 | find_circ | SRR11550045 |
| SARS-CoV-2_circ_Homo_sapiens_2075 | MN908947.3 | 20069 | 25659 | + | ORF3a;S;ORF1ab | 3 | CIRI2 | SRR11550044 |
| SARS-CoV-2_circ_Homo_sapiens_208 | MN908947.3 | 907 | 1600 | - | ORF1ab | 3 | find_circ | SRR11550045 |
| SARS-CoV-2_circ_Homo_sapiens_2081 | MN908947.3 | 20270 | 20631 | + | ORF1ab | 3 | find_circ | SRR11550043 |
| SARS-CoV-2_circ_Homo_sapiens_2085 | MN908947.3 | 20336 | 22858 | + | S;ORF1ab | 3 | circRNA_finder | SRR11550044 |
| SARS-CoV-2_circ_Homo_sapiens_2085 | MN908947.3 | 20336 | 22858 | + | S;ORF1ab | 3 | find_circ | SRR11550044 |
| SARS-CoV-2_circ_Homo_sapiens_2086 | MN908947.3 | 20357 | 21665 | + | S;ORF1ab | 3 | find_circ | SRR11550044 |
| SARS-CoV-2_circ_Homo_sapiens_209 | MN908947.3 | 907 | 1600 | + | ORF1ab | 3 | circRNA_finder | SRR11550045 |
| SARS-CoV-2_circ_Homo_sapiens_2096 | MN908947.3 | 20405 | 20955 | - | ORF1ab | 3 | find_circ | SRR11550044 |
| SARS-CoV-2_circ_Homo_sapiens_2097 | MN908947.3 | 20405 | 20955 | + | ORF1ab | 3 | circRNA_finder | SRR11550044 |
| SARS-CoV-2_circ_Homo_sapiens_2115 | MN908947.3 | 20763 | 21653 | - | S;ORF1ab | 3 | find_circ | SRR11550045 |
| SARS-CoV-2_circ_Homo_sapiens_2116 | MN908947.3 | 20763 | 21653 | + | S;ORF1ab | 3 | circRNA_finder | SRR11550045 |
| SARS-CoV-2_circ_Homo_sapiens_2136 | MN908947.3 | 21100 | 21665 | + | S;ORF1ab | 3 | find_circ | SRR11550045 |
| SARS-CoV-2_circ_Homo_sapiens_2145 | MN908947.3 | 21179 | 21540 | - | ORF1ab | 3 | find_circ | SRR11550044 |
| SARS-CoV-2_circ_Homo_sapiens_2151 | MN908947.3 | 21605 | 23485 | + | S | 3 | find_circ | SRR11550045 |
| SARS-CoV-2_circ_Homo_sapiens_2156 | MN908947.3 | 21677 | 22512 | - | S | 3 | find_circ | SRR11550044 |
| SARS-CoV-2_circ_Homo_sapiens_2169 | MN908947.3 | 21722 | 23429 | - | S | 3 | find_circ | SRR11550045 |
| SARS-CoV-2_circ_Homo_sapiens_2174 | MN908947.3 | 21732 | 22387 | - | S | 3 | CIRI2 | SRR11550046 |
| SARS-CoV-2_circ_Homo_sapiens_2206 | MN908947.3 | 21894 | 22360 | + | S | 3 | find_circ | SRR11550045 |
| SARS-CoV-2_circ_Homo_sapiens_2213 | MN908947.3 | 21904 | 22382 | - | S | 3 | find_circ | SRR11550046 |
| SARS-CoV-2_circ_Homo_sapiens_2214 | MN908947.3 | 21904 | 22865 | - | S | 3 | find_circ | SRR11550046 |
| SARS-CoV-2_circ_Homo_sapiens_2216 | MN908947.3 | 21998 | 22387 | - | S | 3 | find_circ | SRR11550045 |
| SARS-CoV-2_circ_Homo_sapiens_2218 | MN908947.3 | 21998 | 22431 | - | S | 3 | find_circ | SRR11550046 |
| SARS-CoV-2_circ_Homo_sapiens_2233 | MN908947.3 | 22287 | 22854 | - | S | 3 | find_circ | SRR11550045 |
| SARS-CoV-2_circ_Homo_sapiens_2251 | MN908947.3 | 22420 | 22865 | - | S | 3 | find_circ | SRR11550045 |
| SARS-CoV-2_circ_Homo_sapiens_2268 | MN908947.3 | 22741 | 23429 | - | S | 3 | find_circ | SRR11550045 |
| SARS-CoV-2_circ_Homo_sapiens_2274 | MN908947.3 | 22933 | 24276 | + | S | 3 | CIRI2 | SRR11550044 |
| SARS-CoV-2_circ_Homo_sapiens_2284 | MN908947.3 | 22950 | 23576 | - | S | 3 | find_circ | SRR11550045 |
| SARS-CoV-2_circ_Homo_sapiens_2289 | MN908947.3 | 22950 | 25299 | - | S | 3 | find_circ | SRR11550044 |
| SARS-CoV-2_circ_Homo_sapiens_2290 | MN908947.3 | 22950 | 25299 | + | S | 3 | circRNA_finder | SRR11550044 |
| SARS-CoV-2_circ_Homo_sapiens_2291 | MN908947.3 | 22950 | 25516 | - | ORF3a;S | 3 | find_circ | SRR11550046 |
| SARS-CoV-2_circ_Homo_sapiens_2292 | MN908947.3 | 22950 | 25516 | + | ORF3a;S | 3 | circRNA_finder | SRR11550046 |
| SARS-CoV-2_circ_Homo_sapiens_2296 | MN908947.3 | 22950 | 26954 | - | ORF3a;S;M;E | 3 | find_circ | SRR11550046 |
| SARS-CoV-2_circ_Homo_sapiens_2297 | MN908947.3 | 22950 | 26954 | + | ORF3a;S;M;E | 3 | circRNA_finder | SRR11550046 |
| SARS-CoV-2_circ_Homo_sapiens_2299 | MN908947.3 | 22962 | 23567 | + | S | 3 | find_circ | SRR11550045 |
| SARS-CoV-2_circ_Homo_sapiens_23 | MN908947.3 | 8 | 21769 | + | S;ORF1ab | 3 | find_circ | SRR11550044 |
| SARS-CoV-2_circ_Homo_sapiens_230 | MN908947.3 | 1009 | 1357 | + | ORF1ab | 3 | find_circ | SRR11550046 |
| SARS-CoV-2_circ_Homo_sapiens_2304 | MN908947.3 | 22998 | 25671 | - | ORF3a;S | 3 | find_circ | SRR11550045 |
| SARS-CoV-2_circ_Homo_sapiens_2305 | MN908947.3 | 22998 | 25671 | + | ORF3a;S | 3 | circRNA_finder | SRR11550045 |
| SARS-CoV-2_circ_Homo_sapiens_231 | MN908947.3 | 1009 | 1360 | + | ORF1ab | 3 | find_circ | SRR11550046 |
| SARS-CoV-2_circ_Homo_sapiens_232 | MN908947.3 | 1009 | 1490 | + | ORF1ab | 3 | find_circ | SRR11550043 |
| SARS-CoV-2_circ_Homo_sapiens_2329 | MN908947.3 | 23142 | 23684 | - | S | 3 | find_circ | SRR11550046 |
| SARS-CoV-2_circ_Homo_sapiens_233 | MN908947.3 | 1009 | 1574 | + | ORF1ab | 3 | find_circ | SRR11550045 |
| SARS-CoV-2_circ_Homo_sapiens_2330 | MN908947.3 | 23142 | 23684 | + | S | 3 | circRNA_finder | SRR11550046 |
| SARS-CoV-2_circ_Homo_sapiens_234 | MN908947.3 | 1009 | 1576 | + | ORF1ab | 3 | find_circ | SRR11550046 |
| SARS-CoV-2_circ_Homo_sapiens_2351 | MN908947.3 | 23307 | 26570 | - | ORF3a;S;M;E | 3 | find_circ | SRR11550046 |
| SARS-CoV-2_circ_Homo_sapiens_2352 | MN908947.3 | 23307 | 26775 | - | ORF3a;S;M;E | 3 | find_circ | SRR11550044 |
| SARS-CoV-2_circ_Homo_sapiens_2374 | MN908947.3 | 23556 | 24457 | - | S | 3 | find_circ | SRR11550043 |
| SARS-CoV-2_circ_Homo_sapiens_2379 | MN908947.3 | 23568 | 24192 | + | S | 3 | circRNA_finder | SRR11550045 |
| SARS-CoV-2_circ_Homo_sapiens_2386 | MN908947.3 | 23568 | 25428 | + | ORF3a;S | 3 | CIRI2 | SRR11550043 |
| SARS-CoV-2_circ_Homo_sapiens_2386 | MN908947.3 | 23568 | 25428 | + | ORF3a;S | 3 | circRNA_finder | SRR11550046 |
| SARS-CoV-2_circ_Homo_sapiens_2387 | MN908947.3 | 23568 | 25444 | + | ORF3a;S | 3 | circRNA_finder | SRR11550046 |
| SARS-CoV-2_circ_Homo_sapiens_2387 | MN908947.3 | 23568 | 25444 | + | ORF3a;S | 3 | find_circ | SRR11550046 |
| SARS-CoV-2_circ_Homo_sapiens_2393 | MN908947.3 | 23568 | 26778 | + | ORF3a;S;M;E | 3 | find_circ | SRR11550043 |
| SARS-CoV-2_circ_Homo_sapiens_2394 | MN908947.3 | 23568 | 26783 | + | ORF3a;S;M;E | 3 | CIRI2 | SRR11550045 |
| SARS-CoV-2_circ_Homo_sapiens_2405 | MN908947.3 | 23581 | 25428 | + | ORF3a;S | 3 | find_circ | SRR11550045 |
| SARS-CoV-2_circ_Homo_sapiens_2405 | MN908947.3 | 23581 | 25428 | + | ORF3a;S | 3 | circRNA_finder | SRR11550045 |
| SARS-CoV-2_circ_Homo_sapiens_2406 | MN908947.3 | 23581 | 25653 | + | ORF3a;S | 3 | circRNA_finder | SRR11550045 |
| SARS-CoV-2_circ_Homo_sapiens_2406 | MN908947.3 | 23581 | 25653 | + | ORF3a;S | 3 | find_circ | SRR11550045 |
| SARS-CoV-2_circ_Homo_sapiens_2413 | MN908947.3 | 23594 | 24192 | + | S | 3 | find_circ | SRR11550046 |
| SARS-CoV-2_circ_Homo_sapiens_2413 | MN908947.3 | 23594 | 24192 | + | S | 3 | find_circ | SRR11550045 |
| SARS-CoV-2_circ_Homo_sapiens_2415 | MN908947.3 | 23594 | 25014 | + | S | 3 | find_circ | SRR11550043 |
| SARS-CoV-2_circ_Homo_sapiens_2419 | MN908947.3 | 23625 | 24006 | + | S | 3 | circRNA_finder | SRR11550046 |
| SARS-CoV-2_circ_Homo_sapiens_2421 | MN908947.3 | 23625 | 24946 | + | S | 3 | find_circ | SRR11550046 |
| SARS-CoV-2_circ_Homo_sapiens_2421 | MN908947.3 | 23625 | 24946 | + | S | 3 | circRNA_finder | SRR11550046 |
| SARS-CoV-2_circ_Homo_sapiens_2440 | MN908947.3 | 23736 | 24284 | + | S | 3 | find_circ | SRR11550045 |
| SARS-CoV-2_circ_Homo_sapiens_2456 | MN908947.3 | 24027 | 24620 | - | S | 3 | find_circ | SRR11550045 |
| SARS-CoV-2_circ_Homo_sapiens_2456 | MN908947.3 | 24027 | 24620 | - | S | 3 | find_circ | SRR11550046 |
| SARS-CoV-2_circ_Homo_sapiens_2457 | MN908947.3 | 24027 | 24620 | + | S | 3 | circRNA_finder | SRR11550046 |
| SARS-CoV-2_circ_Homo_sapiens_2484 | MN908947.3 | 24150 | 26570 | - | ORF3a;S;M;E | 3 | find_circ | SRR11550046 |
| SARS-CoV-2_circ_Homo_sapiens_2485 | MN908947.3 | 24150 | 26570 | + | ORF3a;S;M;E | 3 | circRNA_finder | SRR11550046 |
| SARS-CoV-2_circ_Homo_sapiens_2492 | MN908947.3 | 24198 | 25533 | + | ORF3a;S | 3 | find_circ | SRR11550045 |
| SARS-CoV-2_circ_Homo_sapiens_2499 | MN908947.3 | 24349 | 24763 | + | S | 3 | find_circ | SRR11550045 |
| SARS-CoV-2_circ_Homo_sapiens_2499 | MN908947.3 | 24349 | 24763 | + | S | 3 | circRNA_finder | SRR11550045 |
| SARS-CoV-2_circ_Homo_sapiens_2500 | MN908947.3 | 24349 | 24871 | + | S | 3 | find_circ | SRR11550046 |
| SARS-CoV-2_circ_Homo_sapiens_2500 | MN908947.3 | 24349 | 24871 | + | S | 3 | circRNA_finder | SRR11550046 |
| SARS-CoV-2_circ_Homo_sapiens_2506 | MN908947.3 | 24375 | 25548 | - | ORF3a;S | 3 | CIRI2 | SRR11550046 |
| SARS-CoV-2_circ_Homo_sapiens_2513 | MN908947.3 | 24459 | 24864 | - | S | 3 | find_circ | SRR11550046 |
| SARS-CoV-2_circ_Homo_sapiens_2528 | MN908947.3 | 24580 | 27130 | - | ORF3a;S;M;E | 3 | find_circ | SRR11550045 |
| SARS-CoV-2_circ_Homo_sapiens_2529 | MN908947.3 | 24580 | 27130 | + | ORF3a;S;M;E | 3 | circRNA_finder | SRR11550045 |
| SARS-CoV-2_circ_Homo_sapiens_254 | MN908947.3 | 1058 | 1479 | - | ORF1ab | 3 | find_circ | SRR11550043 |
| SARS-CoV-2_circ_Homo_sapiens_2547 | MN908947.3 | 24922 | 25548 | - | ORF3a;S | 3 | find_circ | SRR11550045 |
| SARS-CoV-2_circ_Homo_sapiens_2556 | MN908947.3 | 24954 | 25653 | + | ORF3a;S | 3 | find_circ | SRR11550045 |
| SARS-CoV-2_circ_Homo_sapiens_2556 | MN908947.3 | 24954 | 25653 | + | ORF3a;S | 3 | circRNA_finder | SRR11550045 |
| SARS-CoV-2_circ_Homo_sapiens_256 | MN908947.3 | 1252 | 2112 | - | ORF1ab | 3 | find_circ | SRR11550045 |
| SARS-CoV-2_circ_Homo_sapiens_2561 | MN908947.3 | 24990 | 25516 | - | ORF3a;S | 3 | find_circ | SRR11550046 |
| SARS-CoV-2_circ_Homo_sapiens_2561 | MN908947.3 | 24990 | 25516 | - | ORF3a;S | 3 | CIRI2 | SRR11550046 |
| SARS-CoV-2_circ_Homo_sapiens_2562 | MN908947.3 | 24990 | 25516 | + | ORF3a;S | 3 | circRNA_finder | SRR11550046 |
| SARS-CoV-2_circ_Homo_sapiens_2563 | MN908947.3 | 24990 | 25548 | - | ORF3a;S | 3 | find_circ | SRR11550045 |
| SARS-CoV-2_circ_Homo_sapiens_2575 | MN908947.3 | 24999 | 25476 | + | ORF3a;S | 3 | find_circ | SRR11550046 |
| SARS-CoV-2_circ_Homo_sapiens_2584 | MN908947.3 | 25020 | 25428 | + | ORF3a;S | 3 | find_circ | SRR11550043 |
| SARS-CoV-2_circ_Homo_sapiens_2584 | MN908947.3 | 25020 | 25428 | + | ORF3a;S | 3 | circRNA_finder | SRR11550045 |
| SARS-CoV-2_circ_Homo_sapiens_2584 | MN908947.3 | 25020 | 25428 | + | ORF3a;S | 3 | circRNA_finder | SRR11550043 |
| SARS-CoV-2_circ_Homo_sapiens_2585 | MN908947.3 | 25020 | 25444 | + | ORF3a;S | 3 | find_circ | SRR11550046 |
| SARS-CoV-2_circ_Homo_sapiens_2592 | MN908947.3 | 25062 | 25444 | + | ORF3a;S | 3 | circRNA_finder | SRR11550046 |
| SARS-CoV-2_circ_Homo_sapiens_2592 | MN908947.3 | 25062 | 25444 | + | ORF3a;S | 3 | find_circ | SRR11550046 |
| SARS-CoV-2_circ_Homo_sapiens_2605 | MN908947.3 | 25391 | 25823 | - | ORF3a | 3 | find_circ | SRR11550043 |
| SARS-CoV-2_circ_Homo_sapiens_2606 | MN908947.3 | 25391 | 25823 | + | ORF3a | 3 | circRNA_finder | SRR11550043 |
| SARS-CoV-2_circ_Homo_sapiens_2611 | MN908947.3 | 25391 | 26780 | - | ORF3a;M;E | 3 | find_circ | SRR11550045 |
| SARS-CoV-2_circ_Homo_sapiens_2613 | MN908947.3 | 25391 | 26876 | - | ORF3a;M;E | 3 | find_circ | SRR11550046 |
| SARS-CoV-2_circ_Homo_sapiens_2616 | MN908947.3 | 25391 | 27130 | - | ORF3a;M;E | 3 | find_circ | SRR11550043 |
| SARS-CoV-2_circ_Homo_sapiens_2617 | MN908947.3 | 25391 | 27130 | + | ORF3a;M;E | 3 | circRNA_finder | SRR11550043 |
| SARS-CoV-2_circ_Homo_sapiens_2621 | MN908947.3 | 25434 | 26775 | - | ORF3a;M;E | 3 | find_circ | SRR11550043 |
| SARS-CoV-2_circ_Homo_sapiens_2625 | MN908947.3 | 25434 | 29450 | - | ORF6;M;E;ORF3a;N;ORF8;ORF7a;ORF7b | 3 | find_circ | SRR11550043 |
| SARS-CoV-2_circ_Homo_sapiens_2649 | MN908947.3 | 25627 | 26780 | - | ORF3a;M;E | 3 | find_circ | SRR11550045 |
| SARS-CoV-2_circ_Homo_sapiens_2650 | MN908947.3 | 25627 | 26780 | + | ORF3a;M;E | 3 | circRNA_finder | SRR11550045 |
| SARS-CoV-2_circ_Homo_sapiens_2656 | MN908947.3 | 25659 | 26788 | - | ORF3a;M;E | 3 | find_circ | SRR11550046 |
| SARS-CoV-2_circ_Homo_sapiens_2658 | MN908947.3 | 25661 | 26283 | + | ORF3a;E | 3 | circRNA_finder | SRR11550045 |
| SARS-CoV-2_circ_Homo_sapiens_2658 | MN908947.3 | 25661 | 26283 | + | ORF3a;E | 3 | CIRI2 | SRR11550045 |
| SARS-CoV-2_circ_Homo_sapiens_2658 | MN908947.3 | 25661 | 26283 | + | ORF3a;E | 3 | find_circ | SRR11550045 |
| SARS-CoV-2_circ_Homo_sapiens_2665 | MN908947.3 | 25672 | 26203 | - | ORF3a | 3 | find_circ | SRR11550045 |
| SARS-CoV-2_circ_Homo_sapiens_2667 | MN908947.3 | 25672 | 26212 | - | ORF3a | 3 | find_circ | SRR11550046 |
| SARS-CoV-2_circ_Homo_sapiens_2667 | MN908947.3 | 25672 | 26212 | - | ORF3a | 3 | find_circ | SRR11550044 |
| SARS-CoV-2_circ_Homo_sapiens_2668 | MN908947.3 | 25672 | 26212 | + | ORF3a | 3 | circRNA_finder | SRR11550046 |
| SARS-CoV-2_circ_Homo_sapiens_2668 | MN908947.3 | 25672 | 26212 | + | ORF3a | 3 | circRNA_finder | SRR11550043 |
| SARS-CoV-2_circ_Homo_sapiens_2684 | MN908947.3 | 25672 | 26541 | + | ORF3a;M;E | 3 | circRNA_finder | SRR11550045 |
| SARS-CoV-2_circ_Homo_sapiens_2685 | MN908947.3 | 25672 | 26570 | - | ORF3a;M;E | 3 | find_circ | SRR11550043 |
| SARS-CoV-2_circ_Homo_sapiens_2685 | MN908947.3 | 25672 | 26570 | - | ORF3a;M;E | 3 | find_circ | SRR11550044 |
| SARS-CoV-2_circ_Homo_sapiens_2686 | MN908947.3 | 25672 | 26570 | + | ORF3a;M;E | 3 | circRNA_finder | SRR11550044 |
| SARS-CoV-2_circ_Homo_sapiens_2686 | MN908947.3 | 25672 | 26570 | + | ORF3a;M;E | 3 | circRNA_finder | SRR11550045 |
| SARS-CoV-2_circ_Homo_sapiens_2690 | MN908947.3 | 25672 | 26775 | - | ORF3a;M;E | 3 | find_circ | SRR11550045 |
| SARS-CoV-2_circ_Homo_sapiens_2691 | MN908947.3 | 25672 | 26775 | + | ORF3a;M;E | 3 | circRNA_finder | SRR11550045 |
| SARS-CoV-2_circ_Homo_sapiens_2692 | MN908947.3 | 25672 | 26780 | - | ORF3a;M;E | 3 | find_circ | SRR11550043 |
| SARS-CoV-2_circ_Homo_sapiens_2692 | MN908947.3 | 25672 | 26780 | - | ORF3a;M;E | 3 | CIRI2 | SRR11550043 |
| SARS-CoV-2_circ_Homo_sapiens_2693 | MN908947.3 | 25672 | 26780 | + | ORF3a;M;E | 3 | circRNA_finder | SRR11550046 |
| SARS-CoV-2_circ_Homo_sapiens_2695 | MN908947.3 | 25672 | 26817 | - | ORF3a;M;E | 3 | find_circ | SRR11550046 |
| SARS-CoV-2_circ_Homo_sapiens_2704 | MN908947.3 | 25672 | 27015 | + | ORF3a;M;E | 3 | circRNA_finder | SRR11550043 |
| SARS-CoV-2_circ_Homo_sapiens_2709 | MN908947.3 | 25672 | 27130 | + | ORF3a;M;E | 3 | circRNA_finder | SRR11550044 |
| SARS-CoV-2_circ_Homo_sapiens_2710 | MN908947.3 | 25672 | 27218 | - | ORF6;ORF3a;M;E | 3 | find_circ | SRR11550045 |
| SARS-CoV-2_circ_Homo_sapiens_2711 | MN908947.3 | 25672 | 27218 | + | ORF6;ORF3a;M;E | 3 | circRNA_finder | SRR11550045 |
| SARS-CoV-2_circ_Homo_sapiens_2717 | MN908947.3 | 25672 | 29214 | - | ORF6;M;E;ORF3a;N;ORF8;ORF7a;ORF7b | 3 | CIRI2 | SRR11550044 |
| SARS-CoV-2_circ_Homo_sapiens_2722 | MN908947.3 | 25727 | 26208 | + | ORF3a | 3 | find_circ | SRR11550045 |
| SARS-CoV-2_circ_Homo_sapiens_2727 | MN908947.3 | 25743 | 26157 | + | ORF3a | 3 | find_circ | SRR11550046 |
| SARS-CoV-2_circ_Homo_sapiens_2729 | MN908947.3 | 25758 | 26157 | + | ORF3a | 3 | find_circ | SRR11550043 |
| SARS-CoV-2_circ_Homo_sapiens_2729 | MN908947.3 | 25758 | 26157 | + | ORF3a | 3 | find_circ | SRR11550045 |
| SARS-CoV-2_circ_Homo_sapiens_2730 | MN908947.3 | 25758 | 26276 | + | ORF3a;E | 3 | find_circ | SRR11550043 |
| SARS-CoV-2_circ_Homo_sapiens_2731 | MN908947.3 | 25770 | 26157 | + | ORF3a | 3 | find_circ | SRR11550046 |
| SARS-CoV-2_circ_Homo_sapiens_274 | MN908947.3 | 1427 | 2821 | - | ORF1ab | 3 | find_circ | SRR11550044 |
| SARS-CoV-2_circ_Homo_sapiens_2741 | MN908947.3 | 25825 | 26212 | - | ORF3a | 3 | find_circ | SRR11550044 |
| SARS-CoV-2_circ_Homo_sapiens_275 | MN908947.3 | 1427 | 2821 | + | ORF1ab | 3 | circRNA_finder | SRR11550044 |
| SARS-CoV-2_circ_Homo_sapiens_2760 | MN908947.3 | 26285 | 26659 | + | M;E | 3 | circRNA_finder | SRR11550044 |
| SARS-CoV-2_circ_Homo_sapiens_2760 | MN908947.3 | 26285 | 26659 | + | M;E | 3 | find_circ | SRR11550044 |
| SARS-CoV-2_circ_Homo_sapiens_2766 | MN908947.3 | 26285 | 27191 | + | M;E | 3 | find_circ | SRR11550045 |
| SARS-CoV-2_circ_Homo_sapiens_2766 | MN908947.3 | 26285 | 27191 | + | M;E | 3 | circRNA_finder | SRR11550045 |
| SARS-CoV-2_circ_Homo_sapiens_2775 | MN908947.3 | 26578 | 26954 | - | M | 3 | find_circ | SRR11550046 |
| SARS-CoV-2_circ_Homo_sapiens_2775 | MN908947.3 | 26578 | 26954 | - | M | 3 | find_circ | SRR11550044 |
| SARS-CoV-2_circ_Homo_sapiens_2775 | MN908947.3 | 26578 | 26954 | - | M | 3 | find_circ | SRR11550045 |
| SARS-CoV-2_circ_Homo_sapiens_2775 | MN908947.3 | 26578 | 26954 | - | M | 3 | find_circ | SRR11550043 |
| SARS-CoV-2_circ_Homo_sapiens_2776 | MN908947.3 | 26578 | 26954 | + | M | 3 | circRNA_finder | SRR11550044 |
| SARS-CoV-2_circ_Homo_sapiens_2776 | MN908947.3 | 26578 | 26954 | + | M | 3 | circRNA_finder | SRR11550045 |
| SARS-CoV-2_circ_Homo_sapiens_2776 | MN908947.3 | 26578 | 26954 | + | M | 3 | circRNA_finder | SRR11550043 |
| SARS-CoV-2_circ_Homo_sapiens_2778 | MN908947.3 | 26578 | 29665 | - | ORF6;ORF10;M;N;ORF8;ORF7a;ORF7b | 3 | find_circ | SRR11550045 |
| SARS-CoV-2_circ_Homo_sapiens_2781 | MN908947.3 | 26673 | 27225 | + | ORF6;M | 3 | circRNA_finder | SRR11550046 |
| SARS-CoV-2_circ_Homo_sapiens_2781 | MN908947.3 | 26673 | 27225 | + | ORF6;M | 3 | find_circ | SRR11550046 |
| SARS-CoV-2_circ_Homo_sapiens_2783 | MN908947.3 | 26705 | 27130 | - | M | 3 | find_circ | SRR11550044 |
| SARS-CoV-2_circ_Homo_sapiens_2797 | MN908947.3 | 26825 | 27270 | + | ORF6;M | 3 | find_circ | SRR11550044 |
| SARS-CoV-2_circ_Homo_sapiens_2798 | MN908947.3 | 26825 | 27905 | + | ORF6;M;ORF8;ORF7a;ORF7b | 3 | find_circ | SRR11550044 |
| SARS-CoV-2_circ_Homo_sapiens_2802 | MN908947.3 | 26827 | 27285 | - | ORF6;M | 3 | find_circ | SRR11550044 |
| SARS-CoV-2_circ_Homo_sapiens_2808 | MN908947.3 | 26840 | 27051 | - | M | 3 | find_circ | SRR11550045 |
| SARS-CoV-2_circ_Homo_sapiens_2809 | MN908947.3 | 26840 | 27051 | + | M | 3 | circRNA_finder | SRR11550045 |
| SARS-CoV-2_circ_Homo_sapiens_281 | MN908947.3 | 1520 | 2484 | - | ORF1ab | 3 | find_circ | SRR11550045 |
| SARS-CoV-2_circ_Homo_sapiens_2836 | MN908947.3 | 27060 | 27583 | - | ORF7a;ORF6;M | 3 | find_circ | SRR11550044 |
| SARS-CoV-2_circ_Homo_sapiens_2846 | MN908947.3 | 27060 | 29329 | - | ORF6;M;N;ORF8;ORF7a;ORF7b | 3 | find_circ | SRR11550046 |
| SARS-CoV-2_circ_Homo_sapiens_2848 | MN908947.3 | 27088 | 27191 | + | M | 3 | find_circ | SRR11550043 |
| SARS-CoV-2_circ_Homo_sapiens_2849 | MN908947.3 | 27093 | 27541 | - | ORF7a;ORF6;M | 3 | find_circ | SRR11550045 |
| SARS-CoV-2_circ_Homo_sapiens_2858 | MN908947.3 | 27144 | 29268 | - | ORF6;M;N;ORF8;ORF7a;ORF7b | 3 | find_circ | SRR11550043 |
| SARS-CoV-2_circ_Homo_sapiens_2859 | MN908947.3 | 27144 | 29268 | + | ORF6;M;N;ORF8;ORF7a;ORF7b | 3 | circRNA_finder | SRR11550043 |
| SARS-CoV-2_circ_Homo_sapiens_2871 | MN908947.3 | 27220 | 27678 | - | ORF7a;ORF6 | 3 | find_circ | SRR11550045 |
| SARS-CoV-2_circ_Homo_sapiens_2871 | MN908947.3 | 27220 | 27678 | - | ORF7a;ORF6 | 3 | find_circ | SRR11550043 |
| SARS-CoV-2_circ_Homo_sapiens_2872 | MN908947.3 | 27220 | 27678 | + | ORF7a;ORF6 | 3 | circRNA_finder | SRR11550046 |
| SARS-CoV-2_circ_Homo_sapiens_2873 | MN908947.3 | 27220 | 27696 | - | ORF7a;ORF6 | 3 | find_circ | SRR11550045 |
| SARS-CoV-2_circ_Homo_sapiens_2884 | MN908947.3 | 27226 | 29262 | + | ORF6;N;ORF8;ORF7a;ORF7b | 3 | CIRI2 | SRR11550043 |
| SARS-CoV-2_circ_Homo_sapiens_2894 | MN908947.3 | 27264 | 27566 | - | ORF7a;ORF6 | 3 | find_circ | SRR11550043 |
| SARS-CoV-2_circ_Homo_sapiens_2895 | MN908947.3 | 27264 | 27566 | + | ORF7a;ORF6 | 3 | circRNA_finder | SRR11550043 |
| SARS-CoV-2_circ_Homo_sapiens_29 | MN908947.3 | 8 | 26827 | + | M;E;ORF3a;S;ORF1ab | 3 | CIRI2 | SRR11550046 |
| SARS-CoV-2_circ_Homo_sapiens_2933 | MN908947.3 | 27576 | 28092 | - | ORF7a;ORF8;ORF7b | 3 | find_circ | SRR11550045 |
| SARS-CoV-2_circ_Homo_sapiens_2935 | MN908947.3 | 27576 | 28646 | - | ORF7a;ORF8;ORF7b;N | 3 | find_circ | SRR11550044 |
| SARS-CoV-2_circ_Homo_sapiens_2941 | MN908947.3 | 27576 | 29529 | - | ORF7a;ORF8;ORF7b;N | 3 | find_circ | SRR11550045 |
| SARS-CoV-2_circ_Homo_sapiens_2956 | MN908947.3 | 27644 | 28895 | - | ORF7a;ORF8;ORF7b;N | 3 | find_circ | SRR11550044 |
| SARS-CoV-2_circ_Homo_sapiens_2957 | MN908947.3 | 27644 | 28895 | + | ORF7a;ORF8;ORF7b;N | 3 | circRNA_finder | SRR11550044 |
| SARS-CoV-2_circ_Homo_sapiens_2961 | MN908947.3 | 27680 | 28092 | - | ORF7a;ORF8;ORF7b | 3 | find_circ | SRR11550043 |
| SARS-CoV-2_circ_Homo_sapiens_2963 | MN908947.3 | 27685 | 29268 | - | ORF7a;ORF8;ORF7b;N | 3 | find_circ | SRR11550044 |
| SARS-CoV-2_circ_Homo_sapiens_2976 | MN908947.3 | 27780 | 29268 | - | ORF8;ORF7b;N | 3 | find_circ | SRR11550044 |
| SARS-CoV-2_circ_Homo_sapiens_2979 | MN908947.3 | 27799 | 28320 | + | ORF8;ORF7b;N | 3 | find_circ | SRR11550043 |
| SARS-CoV-2_circ_Homo_sapiens_2979 | MN908947.3 | 27799 | 28320 | + | ORF8;ORF7b;N | 3 | circRNA_finder | SRR11550046 |
| SARS-CoV-2_circ_Homo_sapiens_2979 | MN908947.3 | 27799 | 28320 | + | ORF8;ORF7b;N | 3 | circRNA_finder | SRR11550043 |
| SARS-CoV-2_circ_Homo_sapiens_2981 | MN908947.3 | 27799 | 28403 | + | ORF8;ORF7b;N | 3 | find_circ | SRR11550043 |
| SARS-CoV-2_circ_Homo_sapiens_2996 | MN908947.3 | 28060 | 29249 | - | ORF8;N | 3 | CIRI2 | SRR11550043 |
| SARS-CoV-2_circ_Homo_sapiens_30 | MN908947.3 | 8 | 28559 | + | ORF6;M;E;ORF3a;S;N;ORF8;ORF7a;ORF7b;ORF1ab | 3 | find_circ | SRR11550044 |
| SARS-CoV-2_circ_Homo_sapiens_3022 | MN908947.3 | 28321 | 28904 | - | N | 3 | find_circ | SRR11550046 |
| SARS-CoV-2_circ_Homo_sapiens_3027 | MN908947.3 | 28321 | 29066 | - | N | 3 | find_circ | SRR11550043 |
| SARS-CoV-2_circ_Homo_sapiens_3028 | MN908947.3 | 28321 | 29268 | - | N | 3 | CIRI2 | SRR11550043 |
| SARS-CoV-2_circ_Homo_sapiens_3039 | MN908947.3 | 28338 | 28850 | + | N | 3 | find_circ | SRR11550046 |
| SARS-CoV-2_circ_Homo_sapiens_3039 | MN908947.3 | 28338 | 28850 | + | N | 3 | circRNA_finder | SRR11550046 |
| SARS-CoV-2_circ_Homo_sapiens_3042 | MN908947.3 | 28338 | 29080 | + | N | 3 | find_circ | SRR11550043 |
| SARS-CoV-2_circ_Homo_sapiens_3048 | MN908947.3 | 28404 | 28669 | + | N | 3 | find_circ | SRR11550043 |
| SARS-CoV-2_circ_Homo_sapiens_3048 | MN908947.3 | 28404 | 28669 | + | N | 3 | CIRI2 | SRR11550043 |
| SARS-CoV-2_circ_Homo_sapiens_3048 | MN908947.3 | 28404 | 28669 | + | N | 3 | CIRI2 | SRR11550046 |
| SARS-CoV-2_circ_Homo_sapiens_305 | MN908947.3 | 1646 | 3098 | + | ORF1ab | 3 | circRNA_finder | SRR11550046 |
| SARS-CoV-2_circ_Homo_sapiens_3051 | MN908947.3 | 28404 | 28850 | + | N | 3 | circRNA_finder | SRR11550046 |
| SARS-CoV-2_circ_Homo_sapiens_3051 | MN908947.3 | 28404 | 28850 | + | N | 3 | find_circ | SRR11550046 |
| SARS-CoV-2_circ_Homo_sapiens_3052 | MN908947.3 | 28404 | 28877 | + | N | 3 | find_circ | SRR11550045 |
| SARS-CoV-2_circ_Homo_sapiens_3052 | MN908947.3 | 28404 | 28877 | + | N | 3 | circRNA_finder | SRR11550045 |
| SARS-CoV-2_circ_Homo_sapiens_3055 | MN908947.3 | 28404 | 28979 | + | N | 3 | find_circ | SRR11550045 |
| SARS-CoV-2_circ_Homo_sapiens_3055 | MN908947.3 | 28404 | 28979 | + | N | 3 | circRNA_finder | SRR11550045 |
| SARS-CoV-2_circ_Homo_sapiens_306 | MN908947.3 | 1671 | 29802 | - | ORF6;ORF10;M;E;ORF3a;S;N;ORF8;ORF7a;ORF7b;ORF1ab | 3 | find_circ | SRR11550044 |
| SARS-CoV-2_circ_Homo_sapiens_3060 | MN908947.3 | 28404 | 29262 | + | N | 3 | find_circ | SRR11550045 |
| SARS-CoV-2_circ_Homo_sapiens_3060 | MN908947.3 | 28404 | 29262 | + | N | 3 | CIRI2 | SRR11550043 |
| SARS-CoV-2_circ_Homo_sapiens_3060 | MN908947.3 | 28404 | 29262 | + | N | 3 | find_circ | SRR11550046 |
| SARS-CoV-2_circ_Homo_sapiens_3062 | MN908947.3 | 28404 | 29572 | + | ORF10;N | 3 | circRNA_finder | SRR11550046 |
| SARS-CoV-2_circ_Homo_sapiens_3064 | MN908947.3 | 28410 | 28602 | - | N | 3 | find_circ | SRR11550046 |
| SARS-CoV-2_circ_Homo_sapiens_3065 | MN908947.3 | 28410 | 28646 | - | N | 3 | find_circ | SRR11550046 |
| SARS-CoV-2_circ_Homo_sapiens_307 | MN908947.3 | 1671 | 29802 | + | ORF6;ORF10;M;E;ORF3a;S;N;ORF8;ORF7a;ORF7b;ORF1ab | 3 | circRNA_finder | SRR11550044 |
| SARS-CoV-2_circ_Homo_sapiens_3073 | MN908947.3 | 28410 | 28785 | - | N | 3 | find_circ | SRR11550044 |
| SARS-CoV-2_circ_Homo_sapiens_3074 | MN908947.3 | 28410 | 28785 | + | N | 3 | circRNA_finder | SRR11550044 |
| SARS-CoV-2_circ_Homo_sapiens_3078 | MN908947.3 | 28410 | 28904 | - | N | 3 | find_circ | SRR11550045 |
| SARS-CoV-2_circ_Homo_sapiens_3079 | MN908947.3 | 28410 | 28904 | + | N | 3 | circRNA_finder | SRR11550045 |
| SARS-CoV-2_circ_Homo_sapiens_308 | MN908947.3 | 1735 | 4154 | - | ORF1ab | 3 | find_circ | SRR11550045 |
| SARS-CoV-2_circ_Homo_sapiens_3092 | MN908947.3 | 28410 | 29534 | + | N | 3 | circRNA_finder | SRR11550045 |
| SARS-CoV-2_circ_Homo_sapiens_3093 | MN908947.3 | 28410 | 29665 | - | ORF10;N | 3 | find_circ | SRR11550043 |
| SARS-CoV-2_circ_Homo_sapiens_3097 | MN908947.3 | 28420 | 28904 | - | N | 3 | find_circ | SRR11550046 |
| SARS-CoV-2_circ_Homo_sapiens_31 | MN908947.3 | 8 | 28596 | + | ORF6;M;E;ORF3a;S;N;ORF8;ORF7a;ORF7b;ORF1ab | 3 | find_circ | SRR11550044 |
| SARS-CoV-2_circ_Homo_sapiens_31 | MN908947.3 | 8 | 28596 | + | ORF6;M;E;ORF3a;S;N;ORF8;ORF7a;ORF7b;ORF1ab | 3 | find_circ | SRR11550046 |
| SARS-CoV-2_circ_Homo_sapiens_3101 | MN908947.3 | 28435 | 28727 | - | N | 3 | find_circ | SRR11550045 |
| SARS-CoV-2_circ_Homo_sapiens_3102 | MN908947.3 | 28435 | 28865 | - | N | 3 | find_circ | SRR11550043 |
| SARS-CoV-2_circ_Homo_sapiens_3102 | MN908947.3 | 28435 | 28865 | - | N | 3 | find_circ | SRR11550045 |
| SARS-CoV-2_circ_Homo_sapiens_3103 | MN908947.3 | 28435 | 28865 | + | N | 3 | circRNA_finder | SRR11550043 |
| SARS-CoV-2_circ_Homo_sapiens_3103 | MN908947.3 | 28435 | 28865 | + | N | 3 | circRNA_finder | SRR11550045 |
| SARS-CoV-2_circ_Homo_sapiens_3111 | MN908947.3 | 28449 | 28895 | - | N | 3 | find_circ | SRR11550045 |
| SARS-CoV-2_circ_Homo_sapiens_3125 | MN908947.3 | 28463 | 28753 | - | N | 3 | find_circ | SRR11550044 |
| SARS-CoV-2_circ_Homo_sapiens_3125 | MN908947.3 | 28463 | 28753 | - | N | 3 | find_circ | SRR11550045 |
| SARS-CoV-2_circ_Homo_sapiens_3126 | MN908947.3 | 28463 | 28753 | + | N | 3 | circRNA_finder | SRR11550044 |
| SARS-CoV-2_circ_Homo_sapiens_3132 | MN908947.3 | 28463 | 28886 | - | N | 3 | find_circ | SRR11550046 |
| SARS-CoV-2_circ_Homo_sapiens_3133 | MN908947.3 | 28463 | 28886 | + | N | 3 | circRNA_finder | SRR11550046 |
| SARS-CoV-2_circ_Homo_sapiens_3138 | MN908947.3 | 28463 | 28931 | - | N | 3 | CIRI2 | SRR11550044 |
| SARS-CoV-2_circ_Homo_sapiens_3142 | MN908947.3 | 28463 | 28960 | - | N | 3 | find_circ | SRR11550044 |
| SARS-CoV-2_circ_Homo_sapiens_3142 | MN908947.3 | 28463 | 28960 | - | N | 3 | find_circ | SRR11550045 |
| SARS-CoV-2_circ_Homo_sapiens_3143 | MN908947.3 | 28463 | 28960 | + | N | 3 | circRNA_finder | SRR11550044 |
| SARS-CoV-2_circ_Homo_sapiens_3146 | MN908947.3 | 28463 | 29012 | - | N | 3 | find_circ | SRR11550045 |
| SARS-CoV-2_circ_Homo_sapiens_3147 | MN908947.3 | 28463 | 29012 | + | N | 3 | circRNA_finder | SRR11550046 |
| SARS-CoV-2_circ_Homo_sapiens_3147 | MN908947.3 | 28463 | 29012 | + | N | 3 | circRNA_finder | SRR11550043 |
| SARS-CoV-2_circ_Homo_sapiens_3150 | MN908947.3 | 28463 | 29066 | - | N | 3 | find_circ | SRR11550043 |
| SARS-CoV-2_circ_Homo_sapiens_3151 | MN908947.3 | 28463 | 29066 | + | N | 3 | circRNA_finder | SRR11550045 |
| SARS-CoV-2_circ_Homo_sapiens_3151 | MN908947.3 | 28463 | 29066 | + | N | 3 | circRNA_finder | SRR11550044 |
| SARS-CoV-2_circ_Homo_sapiens_3182 | MN908947.3 | 28551 | 28895 | - | N | 3 | find_circ | SRR11550044 |
| SARS-CoV-2_circ_Homo_sapiens_3182 | MN908947.3 | 28551 | 28895 | - | N | 3 | find_circ | SRR11550045 |
| SARS-CoV-2_circ_Homo_sapiens_3183 | MN908947.3 | 28551 | 29012 | - | N | 3 | find_circ | SRR11550043 |
| SARS-CoV-2_circ_Homo_sapiens_3184 | MN908947.3 | 28551 | 29066 | - | N | 3 | find_circ | SRR11550043 |
| SARS-CoV-2_circ_Homo_sapiens_3190 | MN908947.3 | 28607 | 28865 | - | N | 3 | find_circ | SRR11550043 |
| SARS-CoV-2_circ_Homo_sapiens_3198 | MN908947.3 | 28607 | 29024 | - | N | 3 | find_circ | SRR11550046 |
| SARS-CoV-2_circ_Homo_sapiens_3199 | MN908947.3 | 28607 | 29024 | + | N | 3 | circRNA_finder | SRR11550046 |
| SARS-CoV-2_circ_Homo_sapiens_3200 | MN908947.3 | 28607 | 29329 | - | N | 3 | find_circ | SRR11550045 |
| SARS-CoV-2_circ_Homo_sapiens_3206 | MN908947.3 | 28610 | 28895 | + | N | 3 | circRNA_finder | SRR11550046 |
| SARS-CoV-2_circ_Homo_sapiens_3207 | MN908947.3 | 28610 | 28904 | - | N | 3 | find_circ | SRR11550045 |
| SARS-CoV-2_circ_Homo_sapiens_3208 | MN908947.3 | 28610 | 28904 | + | N | 3 | circRNA_finder | SRR11550045 |
| SARS-CoV-2_circ_Homo_sapiens_3212 | MN908947.3 | 28610 | 29012 | - | N | 3 | find_circ | SRR11550046 |
| SARS-CoV-2_circ_Homo_sapiens_3213 | MN908947.3 | 28610 | 29012 | + | N | 3 | circRNA_finder | SRR11550046 |
| SARS-CoV-2_circ_Homo_sapiens_3214 | MN908947.3 | 28610 | 29036 | - | N | 3 | CIRI2 | SRR11550046 |
| SARS-CoV-2_circ_Homo_sapiens_3228 | MN908947.3 | 28652 | 29329 | - | N | 3 | find_circ | SRR11550045 |
| SARS-CoV-2_circ_Homo_sapiens_3248 | MN908947.3 | 28709 | 28895 | - | N | 3 | find_circ | SRR11550045 |
| SARS-CoV-2_circ_Homo_sapiens_3250 | MN908947.3 | 28709 | 29021 | - | N | 3 | find_circ | SRR11550046 |
| SARS-CoV-2_circ_Homo_sapiens_3260 | MN908947.3 | 28733 | 29268 | - | N | 3 | find_circ | SRR11550043 |
| SARS-CoV-2_circ_Homo_sapiens_3260 | MN908947.3 | 28733 | 29268 | - | N | 3 | CIRI2 | SRR11550043 |
| SARS-CoV-2_circ_Homo_sapiens_327 | MN908947.3 | 2111 | 2484 | - | ORF1ab | 3 | find_circ | SRR11550046 |
| SARS-CoV-2_circ_Homo_sapiens_3274 | MN908947.3 | 28790 | 29012 | - | N | 3 | find_circ | SRR11550043 |
| SARS-CoV-2_circ_Homo_sapiens_328 | MN908947.3 | 2111 | 2484 | + | ORF1ab | 3 | circRNA_finder | SRR11550046 |
| SARS-CoV-2_circ_Homo_sapiens_3298 | MN908947.3 | 29073 | 29242 | + | N | 3 | find_circ | SRR11550044 |
| SARS-CoV-2_circ_Homo_sapiens_33 | MN908947.3 | 8 | 29259 | + | ORF6;M;E;ORF3a;S;N;ORF8;ORF7a;ORF7b;ORF1ab | 3 | find_circ | SRR11550044 |
| SARS-CoV-2_circ_Homo_sapiens_3300 | MN908947.3 | 29073 | 29572 | + | ORF10;N | 3 | circRNA_finder | SRR11550043 |
| SARS-CoV-2_circ_Homo_sapiens_3322 | MN908947.3 | 29086 | 29787 | - | ORF10;N | 3 | find_circ | SRR11550043 |
| SARS-CoV-2_circ_Homo_sapiens_3323 | MN908947.3 | 29086 | 29787 | + | ORF10;N | 3 | circRNA_finder | SRR11550043 |
| SARS-CoV-2_circ_Homo_sapiens_3349 | MN908947.3 | 29172 | 29562 | - | ORF10;N | 3 | find_circ | SRR11550045 |
| SARS-CoV-2_circ_Homo_sapiens_3350 | MN908947.3 | 29172 | 29579 | - | ORF10;N | 3 | find_circ | SRR11550044 |
| SARS-CoV-2_circ_Homo_sapiens_3354 | MN908947.3 | 29172 | 29631 | - | ORF10;N | 3 | find_circ | SRR11550046 |
| SARS-CoV-2_circ_Homo_sapiens_3358 | MN908947.3 | 29172 | 29707 | - | ORF10;N | 3 | CIRI2 | SRR11550043 |
| SARS-CoV-2_circ_Homo_sapiens_3359 | MN908947.3 | 29172 | 29707 | + | ORF10;N | 3 | circRNA_finder | SRR11550045 |
| SARS-CoV-2_circ_Homo_sapiens_3363 | MN908947.3 | 29190 | 29658 | - | ORF10;N | 3 | find_circ | SRR11550045 |
| SARS-CoV-2_circ_Homo_sapiens_3365 | MN908947.3 | 29203 | 29840 | + | ORF10;N | 3 | find_circ | SRR11550045 |
| SARS-CoV-2_circ_Homo_sapiens_3374 | MN908947.3 | 29250 | 29607 | - | ORF10;N | 3 | CIRI2 | SRR11550046 |
| SARS-CoV-2_circ_Homo_sapiens_3377 | MN908947.3 | 29250 | 29678 | - | ORF10;N | 3 | CIRI2 | SRR11550046 |
| SARS-CoV-2_circ_Homo_sapiens_3381 | MN908947.3 | 29260 | 29444 | - | N | 3 | find_circ | SRR11550044 |
| SARS-CoV-2_circ_Homo_sapiens_3382 | MN908947.3 | 29260 | 29529 | - | N | 3 | find_circ | SRR11550046 |
| SARS-CoV-2_circ_Homo_sapiens_3383 | MN908947.3 | 29260 | 29579 | - | ORF10;N | 3 | find_circ | SRR11550046 |
| SARS-CoV-2_circ_Homo_sapiens_34 | MN908947.3 | 8 | 29572 | + | ORF6;ORF10;M;E;ORF3a;S;N;ORF8;ORF7a;ORF7b;ORF1ab | 3 | find_circ | SRR11550046 |
| SARS-CoV-2_circ_Homo_sapiens_3400 | MN908947.3 | 29269 | 29579 | + | ORF10;N | 3 | circRNA_finder | SRR11550046 |
| SARS-CoV-2_circ_Homo_sapiens_3402 | MN908947.3 | 29269 | 29665 | - | ORF10;N | 3 | find_circ | SRR11550044 |
| SARS-CoV-2_circ_Homo_sapiens_3414 | MN908947.3 | 29322 | 29446 | + | N | 3 | find_circ | SRR11550044 |
| SARS-CoV-2_circ_Homo_sapiens_3421 | MN908947.3 | 29348 | 29534 | - | N | 3 | find_circ | SRR11550045 |
| SARS-CoV-2_circ_Homo_sapiens_3423 | MN908947.3 | 29348 | 29665 | - | ORF10;N | 3 | find_circ | SRR11550046 |
| SARS-CoV-2_circ_Homo_sapiens_3423 | MN908947.3 | 29348 | 29665 | - | ORF10;N | 3 | find_circ | SRR11550043 |
| SARS-CoV-2_circ_Homo_sapiens_3423 | MN908947.3 | 29348 | 29665 | - | ORF10;N | 3 | find_circ | SRR11550045 |
| SARS-CoV-2_circ_Homo_sapiens_3425 | MN908947.3 | 29348 | 29787 | - | ORF10;N | 3 | find_circ | SRR11550045 |
| SARS-CoV-2_circ_Homo_sapiens_3431 | MN908947.3 | 29452 | 29665 | - | ORF10;N | 3 | find_circ | SRR11550046 |
| SARS-CoV-2_circ_Homo_sapiens_355 | MN908947.3 | 2559 | 3737 | + | ORF1ab | 3 | circRNA_finder | SRR11550045 |
| SARS-CoV-2_circ_Homo_sapiens_36 | MN908947.3 | 8 | 29807 | + | ORF6;ORF10;M;E;ORF3a;S;N;ORF8;ORF7a;ORF7b;ORF1ab | 3 | find_circ | SRR11550045 |
| SARS-CoV-2_circ_Homo_sapiens_364 | MN908947.3 | 2571 | 3263 | + | ORF1ab | 3 | circRNA_finder | SRR11550045 |
| SARS-CoV-2_circ_Homo_sapiens_365 | MN908947.3 | 2571 | 3266 | - | ORF1ab | 3 | find_circ | SRR11550046 |
| SARS-CoV-2_circ_Homo_sapiens_366 | MN908947.3 | 2571 | 3266 | + | ORF1ab | 3 | circRNA_finder | SRR11550046 |
| SARS-CoV-2_circ_Homo_sapiens_368 | MN908947.3 | 2571 | 3539 | - | ORF1ab | 3 | find_circ | SRR11550045 |
| SARS-CoV-2_circ_Homo_sapiens_37 | MN908947.3 | 16 | 320 | - | ORF1ab | 3 | find_circ | SRR11550046 |
| SARS-CoV-2_circ_Homo_sapiens_374 | MN908947.3 | 2578 | 3024 | + | ORF1ab | 3 | circRNA_finder | SRR11550046 |
| SARS-CoV-2_circ_Homo_sapiens_376 | MN908947.3 | 2578 | 29687 | + | ORF6;ORF10;M;E;ORF3a;S;N;ORF8;ORF7a;ORF7b;ORF1ab | 3 | circRNA_finder | SRR11550046 |
| SARS-CoV-2_circ_Homo_sapiens_376 | MN908947.3 | 2578 | 29687 | + | ORF6;ORF10;M;E;ORF3a;S;N;ORF8;ORF7a;ORF7b;ORF1ab | 3 | find_circ | SRR11550046 |
| SARS-CoV-2_circ_Homo_sapiens_380 | MN908947.3 | 2592 | 3353 | + | ORF1ab | 3 | find_circ | SRR11550046 |
| SARS-CoV-2_circ_Homo_sapiens_382 | MN908947.3 | 2676 | 3266 | - | ORF1ab | 3 | CIRI2 | SRR11550046 |
| SARS-CoV-2_circ_Homo_sapiens_389 | MN908947.3 | 2732 | 3201 | + | ORF1ab | 3 | circRNA_finder | SRR11550046 |
| SARS-CoV-2_circ_Homo_sapiens_392 | MN908947.3 | 2774 | 2941 | - | ORF1ab | 3 | find_circ | SRR11550044 |
| SARS-CoV-2_circ_Homo_sapiens_401 | MN908947.3 | 2809 | 3353 | + | ORF1ab | 3 | find_circ | SRR11550045 |
| SARS-CoV-2_circ_Homo_sapiens_402 | MN908947.3 | 2823 | 3329 | - | ORF1ab | 3 | find_circ | SRR11550045 |
| SARS-CoV-2_circ_Homo_sapiens_403 | MN908947.3 | 2823 | 3329 | + | ORF1ab | 3 | circRNA_finder | SRR11550045 |
| SARS-CoV-2_circ_Homo_sapiens_419 | MN908947.3 | 3060 | 3215 | + | ORF1ab | 3 | find_circ | SRR11550045 |
| SARS-CoV-2_circ_Homo_sapiens_42 | MN908947.3 | 16 | 2444 | - | ORF1ab | 3 | find_circ | SRR11550043 |
| SARS-CoV-2_circ_Homo_sapiens_420 | MN908947.3 | 3060 | 3337 | + | ORF1ab | 3 | find_circ | SRR11550046 |
| SARS-CoV-2_circ_Homo_sapiens_434 | MN908947.3 | 3177 | 5588 | - | ORF1ab | 3 | find_circ | SRR11550043 |
| SARS-CoV-2_circ_Homo_sapiens_435 | MN908947.3 | 3177 | 5588 | + | ORF1ab | 3 | circRNA_finder | SRR11550043 |
| SARS-CoV-2_circ_Homo_sapiens_437 | MN908947.3 | 3207 | 28370 | + | ORF6;M;E;ORF3a;S;N;ORF8;ORF7a;ORF7b;ORF1ab | 3 | find_circ | SRR11550043 |
| SARS-CoV-2_circ_Homo_sapiens_45 | MN908947.3 | 16 | 6593 | - | ORF1ab | 3 | find_circ | SRR11550046 |
| SARS-CoV-2_circ_Homo_sapiens_456 | MN908947.3 | 3540 | 4487 | + | ORF1ab | 3 | find_circ | SRR11550045 |
| SARS-CoV-2_circ_Homo_sapiens_460 | MN908947.3 | 3552 | 4595 | - | ORF1ab | 3 | find_circ | SRR11550045 |
| SARS-CoV-2_circ_Homo_sapiens_462 | MN908947.3 | 3552 | 4886 | - | ORF1ab | 3 | find_circ | SRR11550044 |
| SARS-CoV-2_circ_Homo_sapiens_466 | MN908947.3 | 3552 | 11480 | - | ORF1ab | 3 | find_circ | SRR11550043 |
| SARS-CoV-2_circ_Homo_sapiens_47 | MN908947.3 | 16 | 6761 | - | ORF1ab | 3 | find_circ | SRR11550043 |
| SARS-CoV-2_circ_Homo_sapiens_470 | MN908947.3 | 3555 | 4466 | - | ORF1ab | 3 | find_circ | SRR11550045 |
| SARS-CoV-2_circ_Homo_sapiens_471 | MN908947.3 | 3555 | 4466 | + | ORF1ab | 3 | circRNA_finder | SRR11550045 |
| SARS-CoV-2_circ_Homo_sapiens_472 | MN908947.3 | 3555 | 4585 | - | ORF1ab | 3 | find_circ | SRR11550044 |
| SARS-CoV-2_circ_Homo_sapiens_493 | MN908947.3 | 3737 | 5548 | - | ORF1ab | 3 | find_circ | SRR11550046 |
| SARS-CoV-2_circ_Homo_sapiens_496 | MN908947.3 | 3744 | 4533 | - | ORF1ab | 3 | find_circ | SRR11550046 |
| SARS-CoV-2_circ_Homo_sapiens_496 | MN908947.3 | 3744 | 4533 | - | ORF1ab | 3 | find_circ | SRR11550045 |
| SARS-CoV-2_circ_Homo_sapiens_50 | MN908947.3 | 16 | 8326 | - | ORF1ab | 3 | find_circ | SRR11550044 |
| SARS-CoV-2_circ_Homo_sapiens_501 | MN908947.3 | 3778 | 5387 | - | ORF1ab | 3 | find_circ | SRR11550044 |
| SARS-CoV-2_circ_Homo_sapiens_506 | MN908947.3 | 3832 | 4145 | + | ORF1ab | 3 | find_circ | SRR11550045 |
| SARS-CoV-2_circ_Homo_sapiens_513 | MN908947.3 | 3985 | 5821 | - | ORF1ab | 3 | find_circ | SRR11550046 |
| SARS-CoV-2_circ_Homo_sapiens_519 | MN908947.3 | 4156 | 4344 | - | ORF1ab | 3 | find_circ | SRR11550046 |
| SARS-CoV-2_circ_Homo_sapiens_520 | MN908947.3 | 4156 | 4466 | - | ORF1ab | 3 | find_circ | SRR11550043 |
| SARS-CoV-2_circ_Homo_sapiens_524 | MN908947.3 | 4170 | 5347 | - | ORF1ab | 3 | find_circ | SRR11550043 |
| SARS-CoV-2_circ_Homo_sapiens_525 | MN908947.3 | 4170 | 5347 | + | ORF1ab | 3 | circRNA_finder | SRR11550043 |
| SARS-CoV-2_circ_Homo_sapiens_526 | MN908947.3 | 4212 | 4487 | + | ORF1ab | 3 | find_circ | SRR11550045 |
| SARS-CoV-2_circ_Homo_sapiens_530 | MN908947.3 | 4284 | 4835 | + | ORF1ab | 3 | find_circ | SRR11550045 |
| SARS-CoV-2_circ_Homo_sapiens_533 | MN908947.3 | 4286 | 7136 | + | ORF1ab | 3 | circRNA_finder | SRR11550046 |
| SARS-CoV-2_circ_Homo_sapiens_533 | MN908947.3 | 4286 | 7136 | + | ORF1ab | 3 | find_circ | SRR11550046 |
| SARS-CoV-2_circ_Homo_sapiens_534 | MN908947.3 | 4295 | 4835 | + | ORF1ab | 3 | find_circ | SRR11550045 |
| SARS-CoV-2_circ_Homo_sapiens_544 | MN908947.3 | 4535 | 4814 | - | ORF1ab | 3 | find_circ | SRR11550046 |
| SARS-CoV-2_circ_Homo_sapiens_56 | MN908947.3 | 16 | 28602 | - | ORF6;M;E;ORF3a;S;N;ORF8;ORF7a;ORF7b;ORF1ab | 3 | find_circ | SRR11550044 |
| SARS-CoV-2_circ_Homo_sapiens_562 | MN908947.3 | 4560 | 9320 | + | ORF1ab | 3 | find_circ | SRR11550046 |
| SARS-CoV-2_circ_Homo_sapiens_567 | MN908947.3 | 4578 | 5821 | - | ORF1ab | 3 | find_circ | SRR11550045 |
| SARS-CoV-2_circ_Homo_sapiens_568 | MN908947.3 | 4578 | 5906 | - | ORF1ab | 3 | find_circ | SRR11550046 |
| SARS-CoV-2_circ_Homo_sapiens_573 | MN908947.3 | 4597 | 5090 | - | ORF1ab | 3 | find_circ | SRR11550045 |
| SARS-CoV-2_circ_Homo_sapiens_574 | MN908947.3 | 4597 | 5564 | - | ORF1ab | 3 | find_circ | SRR11550046 |
| SARS-CoV-2_circ_Homo_sapiens_58 | MN908947.3 | 16 | 29329 | - | ORF6;M;E;ORF3a;S;N;ORF8;ORF7a;ORF7b;ORF1ab | 3 | find_circ | SRR11550043 |
| SARS-CoV-2_circ_Homo_sapiens_58 | MN908947.3 | 16 | 29329 | - | ORF6;M;E;ORF3a;S;N;ORF8;ORF7a;ORF7b;ORF1ab | 3 | find_circ | SRR11550044 |
| SARS-CoV-2_circ_Homo_sapiens_588 | MN908947.3 | 4836 | 5555 | + | ORF1ab | 3 | find_circ | SRR11550046 |
| SARS-CoV-2_circ_Homo_sapiens_589 | MN908947.3 | 4836 | 5557 | + | ORF1ab | 3 | CIRI2 | SRR11550046 |
| SARS-CoV-2_circ_Homo_sapiens_6 | MN908947.3 | 8 | 514 | + | ORF1ab | 3 | find_circ | SRR11550044 |
| SARS-CoV-2_circ_Homo_sapiens_621 | MN908947.3 | 4921 | 5525 | - | ORF1ab | 3 | find_circ | SRR11550045 |
| SARS-CoV-2_circ_Homo_sapiens_631 | MN908947.3 | 4937 | 5517 | + | ORF1ab | 3 | find_circ | SRR11550045 |
| SARS-CoV-2_circ_Homo_sapiens_638 | MN908947.3 | 4973 | 5396 | + | ORF1ab | 3 | find_circ | SRR11550045 |
| SARS-CoV-2_circ_Homo_sapiens_646 | MN908947.3 | 5116 | 5525 | - | ORF1ab | 3 | find_circ | SRR11550045 |
| SARS-CoV-2_circ_Homo_sapiens_647 | MN908947.3 | 5116 | 5525 | + | ORF1ab | 3 | circRNA_finder | SRR11550045 |
| SARS-CoV-2_circ_Homo_sapiens_648 | MN908947.3 | 5116 | 5783 | - | ORF1ab | 3 | find_circ | SRR11550045 |
| SARS-CoV-2_circ_Homo_sapiens_659 | MN908947.3 | 5130 | 5588 | - | ORF1ab | 3 | find_circ | SRR11550045 |
| SARS-CoV-2_circ_Homo_sapiens_679 | MN908947.3 | 5231 | 5630 | + | ORF1ab | 3 | find_circ | SRR11550043 |
| SARS-CoV-2_circ_Homo_sapiens_683 | MN908947.3 | 5366 | 5729 | - | ORF1ab | 3 | find_circ | SRR11550045 |
| SARS-CoV-2_circ_Homo_sapiens_684 | MN908947.3 | 5366 | 5821 | - | ORF1ab | 3 | find_circ | SRR11550045 |
| SARS-CoV-2_circ_Homo_sapiens_684 | MN908947.3 | 5366 | 5821 | - | ORF1ab | 3 | find_circ | SRR11550046 |
| SARS-CoV-2_circ_Homo_sapiens_690 | MN908947.3 | 5368 | 16282 | + | ORF1ab | 3 | find_circ | SRR11550045 |
| SARS-CoV-2_circ_Homo_sapiens_696 | MN908947.3 | 5478 | 5696 | - | ORF1ab | 3 | find_circ | SRR11550046 |
| SARS-CoV-2_circ_Homo_sapiens_697 | MN908947.3 | 5478 | 5696 | + | ORF1ab | 3 | circRNA_finder | SRR11550046 |
| SARS-CoV-2_circ_Homo_sapiens_701 | MN908947.3 | 5527 | 5978 | - | ORF1ab | 3 | find_circ | SRR11550045 |
| SARS-CoV-2_circ_Homo_sapiens_71 | MN908947.3 | 41 | 400 | - | ORF1ab | 3 | find_circ | SRR11550046 |
| SARS-CoV-2_circ_Homo_sapiens_730 | MN908947.3 | 5811 | 6055 | + | ORF1ab | 3 | find_circ | SRR11550045 |
| SARS-CoV-2_circ_Homo_sapiens_755 | MN908947.3 | 5827 | 25707 | - | ORF3a;S;ORF1ab | 3 | find_circ | SRR11550044 |
| SARS-CoV-2_circ_Homo_sapiens_756 | MN908947.3 | 5827 | 25707 | + | ORF3a;S;ORF1ab | 3 | circRNA_finder | SRR11550044 |
| SARS-CoV-2_circ_Homo_sapiens_774 | MN908947.3 | 5908 | 8292 | - | ORF1ab | 3 | find_circ | SRR11550045 |
| SARS-CoV-2_circ_Homo_sapiens_779 | MN908947.3 | 5989 | 6379 | - | ORF1ab | 3 | find_circ | SRR11550046 |
| SARS-CoV-2_circ_Homo_sapiens_785 | MN908947.3 | 6021 | 6557 | - | ORF1ab | 3 | find_circ | SRR11550046 |
| SARS-CoV-2_circ_Homo_sapiens_788 | MN908947.3 | 6053 | 6512 | + | ORF1ab | 3 | circRNA_finder | SRR11550045 |
| SARS-CoV-2_circ_Homo_sapiens_788 | MN908947.3 | 6053 | 6512 | + | ORF1ab | 3 | find_circ | SRR11550045 |
| SARS-CoV-2_circ_Homo_sapiens_797 | MN908947.3 | 6318 | 6647 | - | ORF1ab | 3 | find_circ | SRR11550043 |
| SARS-CoV-2_circ_Homo_sapiens_8 | MN908947.3 | 8 | 668 | + | ORF1ab | 3 | find_circ | SRR11550046 |
| SARS-CoV-2_circ_Homo_sapiens_814 | MN908947.3 | 6681 | 8926 | + | ORF1ab | 3 | find_circ | SRR11550046 |
| SARS-CoV-2_circ_Homo_sapiens_821 | MN908947.3 | 6735 | 12906 | + | ORF1ab | 3 | circRNA_finder | SRR11550044 |
| SARS-CoV-2_circ_Homo_sapiens_821 | MN908947.3 | 6735 | 12906 | + | ORF1ab | 3 | find_circ | SRR11550044 |
| SARS-CoV-2_circ_Homo_sapiens_826 | MN908947.3 | 7125 | 10009 | - | ORF1ab | 3 | find_circ | SRR11550046 |
| SARS-CoV-2_circ_Homo_sapiens_836 | MN908947.3 | 7778 | 8916 | - | ORF1ab | 3 | find_circ | SRR11550046 |
| SARS-CoV-2_circ_Homo_sapiens_837 | MN908947.3 | 7778 | 8916 | + | ORF1ab | 3 | circRNA_finder | SRR11550046 |
| SARS-CoV-2_circ_Homo_sapiens_841 | MN908947.3 | 7946 | 8921 | - | ORF1ab | 3 | CIRI2 | SRR11550046 |
| SARS-CoV-2_circ_Homo_sapiens_847 | MN908947.3 | 8048 | 29066 | - | ORF6;M;E;ORF3a;S;N;ORF8;ORF7a;ORF7b;ORF1ab | 3 | find_circ | SRR11550046 |
| SARS-CoV-2_circ_Homo_sapiens_852 | MN908947.3 | 8263 | 8873 | - | ORF1ab | 3 | find_circ | SRR11550046 |
| SARS-CoV-2_circ_Homo_sapiens_855 | MN908947.3 | 8293 | 8654 | - | ORF1ab | 3 | find_circ | SRR11550046 |
| SARS-CoV-2_circ_Homo_sapiens_857 | MN908947.3 | 8293 | 8696 | - | ORF1ab | 3 | find_circ | SRR11550045 |
| SARS-CoV-2_circ_Homo_sapiens_861 | MN908947.3 | 8293 | 8921 | - | ORF1ab | 3 | CIRI2 | SRR11550045 |
| SARS-CoV-2_circ_Homo_sapiens_873 | MN908947.3 | 8293 | 11918 | - | ORF1ab | 3 | CIRI2 | SRR11550044 |
| SARS-CoV-2_circ_Homo_sapiens_887 | MN908947.3 | 8327 | 8921 | - | ORF1ab | 3 | CIRI2 | SRR11550045 |
| SARS-CoV-2_circ_Homo_sapiens_892 | MN908947.3 | 8481 | 8904 | - | ORF1ab | 3 | CIRI2 | SRR11550043 |
| SARS-CoV-2_circ_Homo_sapiens_907 | MN908947.3 | 8773 | 10038 | - | ORF1ab | 3 | find_circ | SRR11550044 |
| SARS-CoV-2_circ_Homo_sapiens_942 | MN908947.3 | 9087 | 9176 | + | ORF1ab | 3 | find_circ | SRR11550046 |
| SARS-CoV-2_circ_Homo_sapiens_97 | MN908947.3 | 237 | 442 | + | ORF1ab | 3 | find_circ | SRR11550045 |
| SARS-CoV-2_circ_Homo_sapiens_97 | MN908947.3 | 237 | 442 | + | ORF1ab | 3 | circRNA_finder | SRR11550045 |
| SARS-CoV-2_circ_Homo_sapiens_971 | MN908947.3 | 9636 | 10426 | + | ORF1ab | 3 | find_circ | SRR11550046 |
| SARS-CoV-2_circ_Homo_sapiens_975 | MN908947.3 | 9639 | 10031 | - | ORF1ab | 3 | find_circ | SRR11550046 |
| SARS-CoV-2_circ_Homo_sapiens_975 | MN908947.3 | 9639 | 10031 | - | ORF1ab | 3 | find_circ | SRR11550043 |
| SARS-CoV-2_circ_Homo_sapiens_1 | MN908947.3 | 8 | 236 | + | . | 2 | CIRI2 | SRR11550045 |
| SARS-CoV-2_circ_Homo_sapiens_100 | MN908947.3 | 237 | 644 | + | ORF1ab | 2 | find_circ | SRR11550045 |
| SARS-CoV-2_circ_Homo_sapiens_100 | MN908947.3 | 237 | 644 | + | ORF1ab | 2 | find_circ | SRR11550043 |
| SARS-CoV-2_circ_Homo_sapiens_100 | MN908947.3 | 237 | 644 | + | ORF1ab | 2 | circRNA_finder | SRR11550045 |
| SARS-CoV-2_circ_Homo_sapiens_100 | MN908947.3 | 237 | 644 | + | ORF1ab | 2 | circRNA_finder | SRR11550043 |
| SARS-CoV-2_circ_Homo_sapiens_1000 | MN908947.3 | 9944 | 10649 | - | ORF1ab | 2 | find_circ | SRR11550046 |
| SARS-CoV-2_circ_Homo_sapiens_1011 | MN908947.3 | 9951 | 10663 | + | ORF1ab | 2 | circRNA_finder | SRR11550045 |
| SARS-CoV-2_circ_Homo_sapiens_1011 | MN908947.3 | 9951 | 10663 | + | ORF1ab | 2 | find_circ | SRR11550045 |
| SARS-CoV-2_circ_Homo_sapiens_1014 | MN908947.3 | 10016 | 10457 | - | ORF1ab | 2 | find_circ | SRR11550045 |
| SARS-CoV-2_circ_Homo_sapiens_1015 | MN908947.3 | 10016 | 10518 | - | ORF1ab | 2 | find_circ | SRR11550046 |
| SARS-CoV-2_circ_Homo_sapiens_1016 | MN908947.3 | 10030 | 10524 | - | ORF1ab | 2 | CIRI2 | SRR11550046 |
| SARS-CoV-2_circ_Homo_sapiens_1018 | MN908947.3 | 10030 | 10526 | - | ORF1ab | 2 | find_circ | SRR11550046 |
| SARS-CoV-2_circ_Homo_sapiens_1019 | MN908947.3 | 10030 | 10526 | + | ORF1ab | 2 | circRNA_finder | SRR11550046 |
| SARS-CoV-2_circ_Homo_sapiens_1020 | MN908947.3 | 10030 | 11192 | - | ORF1ab | 2 | find_circ | SRR11550045 |
| SARS-CoV-2_circ_Homo_sapiens_1021 | MN908947.3 | 10030 | 11192 | + | ORF1ab | 2 | circRNA_finder | SRR11550045 |
| SARS-CoV-2_circ_Homo_sapiens_1022 | MN908947.3 | 10044 | 10307 | + | ORF1ab | 2 | circRNA_finder | SRR11550043 |
| SARS-CoV-2_circ_Homo_sapiens_1022 | MN908947.3 | 10044 | 10307 | + | ORF1ab | 2 | find_circ | SRR11550043 |
| SARS-CoV-2_circ_Homo_sapiens_1025 | MN908947.3 | 10044 | 10493 | + | ORF1ab | 2 | find_circ | SRR11550044 |
| SARS-CoV-2_circ_Homo_sapiens_1026 | MN908947.3 | 10044 | 10499 | + | ORF1ab | 2 | find_circ | SRR11550045 |
| SARS-CoV-2_circ_Homo_sapiens_1026 | MN908947.3 | 10044 | 10499 | + | ORF1ab | 2 | find_circ | SRR11550043 |
| SARS-CoV-2_circ_Homo_sapiens_1027 | MN908947.3 | 10044 | 11178 | + | ORF1ab | 2 | circRNA_finder | SRR11550043 |
| SARS-CoV-2_circ_Homo_sapiens_1027 | MN908947.3 | 10044 | 11178 | + | ORF1ab | 2 | find_circ | SRR11550043 |
| SARS-CoV-2_circ_Homo_sapiens_1029 | MN908947.3 | 10055 | 10495 | + | ORF1ab | 2 | find_circ | SRR11550045 |
| SARS-CoV-2_circ_Homo_sapiens_1029 | MN908947.3 | 10055 | 10495 | + | ORF1ab | 2 | find_circ | SRR11550043 |
| SARS-CoV-2_circ_Homo_sapiens_1031 | MN908947.3 | 10066 | 10499 | + | ORF1ab | 2 | circRNA_finder | SRR11550045 |
| SARS-CoV-2_circ_Homo_sapiens_1031 | MN908947.3 | 10066 | 10499 | + | ORF1ab | 2 | find_circ | SRR11550045 |
| SARS-CoV-2_circ_Homo_sapiens_1031 | MN908947.3 | 10066 | 10499 | + | ORF1ab | 2 | CIRI2 | SRR11550045 |
| SARS-CoV-2_circ_Homo_sapiens_1032 | MN908947.3 | 10066 | 10532 | + | ORF1ab | 2 | find_circ | SRR11550046 |
| SARS-CoV-2_circ_Homo_sapiens_1033 | MN908947.3 | 10066 | 10663 | + | ORF1ab | 2 | find_circ | SRR11550046 |
| SARS-CoV-2_circ_Homo_sapiens_1033 | MN908947.3 | 10066 | 10663 | + | ORF1ab | 2 | find_circ | SRR11550043 |
| SARS-CoV-2_circ_Homo_sapiens_1033 | MN908947.3 | 10066 | 10663 | + | ORF1ab | 2 | circRNA_finder | SRR11550046 |
| SARS-CoV-2_circ_Homo_sapiens_1042 | MN908947.3 | 10157 | 10593 | - | ORF1ab | 2 | find_circ | SRR11550045 |
| SARS-CoV-2_circ_Homo_sapiens_1048 | MN908947.3 | 10161 | 10499 | + | ORF1ab | 2 | circRNA_finder | SRR11550046 |
| SARS-CoV-2_circ_Homo_sapiens_1048 | MN908947.3 | 10161 | 10499 | + | ORF1ab | 2 | find_circ | SRR11550046 |
| SARS-CoV-2_circ_Homo_sapiens_1049 | MN908947.3 | 10161 | 10522 | + | ORF1ab | 2 | circRNA_finder | SRR11550046 |
| SARS-CoV-2_circ_Homo_sapiens_1049 | MN908947.3 | 10161 | 10522 | + | ORF1ab | 2 | find_circ | SRR11550046 |
| SARS-CoV-2_circ_Homo_sapiens_1056 | MN908947.3 | 10166 | 10457 | - | ORF1ab | 2 | find_circ | SRR11550046 |
| SARS-CoV-2_circ_Homo_sapiens_1057 | MN908947.3 | 10176 | 10649 | - | ORF1ab | 2 | find_circ | SRR11550045 |
| SARS-CoV-2_circ_Homo_sapiens_1058 | MN908947.3 | 10176 | 10649 | + | ORF1ab | 2 | circRNA_finder | SRR11550045 |
| SARS-CoV-2_circ_Homo_sapiens_106 | MN908947.3 | 272 | 509 | + | ORF1ab | 2 | find_circ | SRR11550044 |
| SARS-CoV-2_circ_Homo_sapiens_1066 | MN908947.3 | 10350 | 10559 | - | ORF1ab | 2 | find_circ | SRR11550045 |
| SARS-CoV-2_circ_Homo_sapiens_1067 | MN908947.3 | 10350 | 10559 | + | ORF1ab | 2 | circRNA_finder | SRR11550045 |
| SARS-CoV-2_circ_Homo_sapiens_107 | MN908947.3 | 312 | 682 | - | ORF1ab | 2 | find_circ | SRR11550043 |
| SARS-CoV-2_circ_Homo_sapiens_107 | MN908947.3 | 312 | 682 | - | ORF1ab | 2 | find_circ | SRR11550044 |
| SARS-CoV-2_circ_Homo_sapiens_1071 | MN908947.3 | 10395 | 11291 | + | ORF1ab | 2 | find_circ | SRR11550044 |
| SARS-CoV-2_circ_Homo_sapiens_1075 | MN908947.3 | 10512 | 11482 | + | ORF1ab | 2 | circRNA_finder | SRR11550044 |
| SARS-CoV-2_circ_Homo_sapiens_1076 | MN908947.3 | 10512 | 23660 | + | S;ORF1ab | 2 | find_circ | SRR11550045 |
| SARS-CoV-2_circ_Homo_sapiens_1076 | MN908947.3 | 10512 | 23660 | + | S;ORF1ab | 2 | circRNA_finder | SRR11550045 |
| SARS-CoV-2_circ_Homo_sapiens_1078 | MN908947.3 | 10583 | 11073 | - | ORF1ab | 2 | find_circ | SRR11550046 |
| SARS-CoV-2_circ_Homo_sapiens_1078 | MN908947.3 | 10583 | 11073 | - | ORF1ab | 2 | find_circ | SRR11550045 |
| SARS-CoV-2_circ_Homo_sapiens_108 | MN908947.3 | 312 | 1008 | - | ORF1ab | 2 | find_circ | SRR11550043 |
| SARS-CoV-2_circ_Homo_sapiens_1088 | MN908947.3 | 10595 | 17351 | - | ORF1ab | 2 | find_circ | SRR11550043 |
| SARS-CoV-2_circ_Homo_sapiens_1089 | MN908947.3 | 10595 | 17351 | + | ORF1ab | 2 | circRNA_finder | SRR11550043 |
| SARS-CoV-2_circ_Homo_sapiens_109 | MN908947.3 | 312 | 3329 | - | ORF1ab | 2 | find_circ | SRR11550044 |
| SARS-CoV-2_circ_Homo_sapiens_1090 | MN908947.3 | 10595 | 27535 | - | ORF6;M;E;ORF3a;S;ORF7a;ORF1ab | 2 | find_circ | SRR11550045 |
| SARS-CoV-2_circ_Homo_sapiens_1092 | MN908947.3 | 10605 | 10931 | + | ORF1ab | 2 | circRNA_finder | SRR11550045 |
| SARS-CoV-2_circ_Homo_sapiens_1093 | MN908947.3 | 10605 | 11035 | - | ORF1ab | 2 | find_circ | SRR11550044 |
| SARS-CoV-2_circ_Homo_sapiens_1094 | MN908947.3 | 10605 | 11035 | + | ORF1ab | 2 | circRNA_finder | SRR11550044 |
| SARS-CoV-2_circ_Homo_sapiens_1095 | MN908947.3 | 10605 | 11066 | - | ORF1ab | 2 | CIRI2 | SRR11550045 |
| SARS-CoV-2_circ_Homo_sapiens_1097 | MN908947.3 | 10605 | 11170 | - | ORF1ab | 2 | find_circ | SRR11550046 |
| SARS-CoV-2_circ_Homo_sapiens_1098 | MN908947.3 | 10605 | 11192 | + | ORF1ab | 2 | circRNA_finder | SRR11550046 |
| SARS-CoV-2_circ_Homo_sapiens_11 | MN908947.3 | 8 | 1835 | + | ORF1ab | 2 | find_circ | SRR11550044 |
| SARS-CoV-2_circ_Homo_sapiens_1100 | MN908947.3 | 10605 | 13139 | - | ORF1ab | 2 | find_circ | SRR11550044 |
| SARS-CoV-2_circ_Homo_sapiens_111 | MN908947.3 | 312 | 27015 | - | M;E;ORF3a;S;ORF1ab | 2 | find_circ | SRR11550044 |
| SARS-CoV-2_circ_Homo_sapiens_1111 | MN908947.3 | 10657 | 10931 | - | ORF1ab | 2 | find_circ | SRR11550045 |
| SARS-CoV-2_circ_Homo_sapiens_1112 | MN908947.3 | 10657 | 11073 | - | ORF1ab | 2 | find_circ | SRR11550044 |
| SARS-CoV-2_circ_Homo_sapiens_1113 | MN908947.3 | 10659 | 11082 | + | ORF1ab | 2 | find_circ | SRR11550045 |
| SARS-CoV-2_circ_Homo_sapiens_1116 | MN908947.3 | 10659 | 11482 | + | ORF1ab | 2 | find_circ | SRR11550046 |
| SARS-CoV-2_circ_Homo_sapiens_1118 | MN908947.3 | 10659 | 12260 | + | ORF1ab | 2 | find_circ | SRR11550045 |
| SARS-CoV-2_circ_Homo_sapiens_1118 | MN908947.3 | 10659 | 12260 | + | ORF1ab | 2 | circRNA_finder | SRR11550046 |
| SARS-CoV-2_circ_Homo_sapiens_1118 | MN908947.3 | 10659 | 12260 | + | ORF1ab | 2 | find_circ | SRR11550046 |
| SARS-CoV-2_circ_Homo_sapiens_1121 | MN908947.3 | 10659 | 18016 | + | ORF1ab | 2 | circRNA_finder | SRR11550045 |
| SARS-CoV-2_circ_Homo_sapiens_1121 | MN908947.3 | 10659 | 18016 | + | ORF1ab | 2 | find_circ | SRR11550045 |
| SARS-CoV-2_circ_Homo_sapiens_1126 | MN908947.3 | 10659 | 28979 | + | ORF6;M;E;ORF3a;S;N;ORF8;ORF7a;ORF7b;ORF1ab | 2 | circRNA_finder | SRR11550045 |
| SARS-CoV-2_circ_Homo_sapiens_1126 | MN908947.3 | 10659 | 28979 | + | ORF6;M;E;ORF3a;S;N;ORF8;ORF7a;ORF7b;ORF1ab | 2 | find_circ | SRR11550045 |
| SARS-CoV-2_circ_Homo_sapiens_1127 | MN908947.3 | 10659 | 29080 | + | ORF6;M;E;ORF3a;S;N;ORF8;ORF7a;ORF7b;ORF1ab | 2 | find_circ | SRR11550046 |
| SARS-CoV-2_circ_Homo_sapiens_1127 | MN908947.3 | 10659 | 29080 | + | ORF6;M;E;ORF3a;S;N;ORF8;ORF7a;ORF7b;ORF1ab | 2 | circRNA_finder | SRR11550046 |
| SARS-CoV-2_circ_Homo_sapiens_113 | MN908947.3 | 316 | 514 | + | ORF1ab | 2 | find_circ | SRR11550046 |
| SARS-CoV-2_circ_Homo_sapiens_1130 | MN908947.3 | 10671 | 11082 | + | ORF1ab | 2 | find_circ | SRR11550046 |
| SARS-CoV-2_circ_Homo_sapiens_1130 | MN908947.3 | 10671 | 11082 | + | ORF1ab | 2 | find_circ | SRR11550045 |
| SARS-CoV-2_circ_Homo_sapiens_1132 | MN908947.3 | 10671 | 11482 | + | ORF1ab | 2 | circRNA_finder | SRR11550043 |
| SARS-CoV-2_circ_Homo_sapiens_1134 | MN908947.3 | 10671 | 12105 | + | ORF1ab | 2 | find_circ | SRR11550045 |
| SARS-CoV-2_circ_Homo_sapiens_1134 | MN908947.3 | 10671 | 12105 | + | ORF1ab | 2 | circRNA_finder | SRR11550045 |
| SARS-CoV-2_circ_Homo_sapiens_1143 | MN908947.3 | 10803 | 18166 | - | ORF1ab | 2 | find_circ | SRR11550045 |
| SARS-CoV-2_circ_Homo_sapiens_1144 | MN908947.3 | 10803 | 18166 | + | ORF1ab | 2 | circRNA_finder | SRR11550045 |
| SARS-CoV-2_circ_Homo_sapiens_1147 | MN908947.3 | 10932 | 11192 | - | ORF1ab | 2 | find_circ | SRR11550046 |
| SARS-CoV-2_circ_Homo_sapiens_1148 | MN908947.3 | 10932 | 11192 | + | ORF1ab | 2 | circRNA_finder | SRR11550046 |
| SARS-CoV-2_circ_Homo_sapiens_1159 | MN908947.3 | 11103 | 11480 | - | ORF1ab | 2 | find_circ | SRR11550046 |
| SARS-CoV-2_circ_Homo_sapiens_116 | MN908947.3 | 316 | 1598 | + | ORF1ab | 2 | find_circ | SRR11550046 |
| SARS-CoV-2_circ_Homo_sapiens_1163 | MN908947.3 | 11190 | 11816 | - | ORF1ab | 2 | find_circ | SRR11550046 |
| SARS-CoV-2_circ_Homo_sapiens_1165 | MN908947.3 | 11190 | 12035 | - | ORF1ab | 2 | find_circ | SRR11550043 |
| SARS-CoV-2_circ_Homo_sapiens_1166 | MN908947.3 | 11190 | 12035 | + | ORF1ab | 2 | circRNA_finder | SRR11550043 |
| SARS-CoV-2_circ_Homo_sapiens_1172 | MN908947.3 | 11208 | 11506 | + | ORF1ab | 2 | circRNA_finder | SRR11550046 |
| SARS-CoV-2_circ_Homo_sapiens_1172 | MN908947.3 | 11208 | 11506 | + | ORF1ab | 2 | find_circ | SRR11550046 |
| SARS-CoV-2_circ_Homo_sapiens_1175 | MN908947.3 | 11284 | 28027 | + | ORF6;M;E;ORF3a;S;ORF8;ORF7a;ORF7b;ORF1ab | 2 | find_circ | SRR11550046 |
| SARS-CoV-2_circ_Homo_sapiens_1177 | MN908947.3 | 11300 | 12056 | + | ORF1ab | 2 | find_circ | SRR11550045 |
| SARS-CoV-2_circ_Homo_sapiens_1177 | MN908947.3 | 11300 | 12056 | + | ORF1ab | 2 | find_circ | SRR11550046 |
| SARS-CoV-2_circ_Homo_sapiens_1179 | MN908947.3 | 11307 | 11850 | + | ORF1ab | 2 | circRNA_finder | SRR11550044 |
| SARS-CoV-2_circ_Homo_sapiens_1187 | MN908947.3 | 11488 | 11959 | - | ORF1ab | 2 | find_circ | SRR11550045 |
| SARS-CoV-2_circ_Homo_sapiens_1188 | MN908947.3 | 11488 | 11966 | - | ORF1ab | 2 | find_circ | SRR11550043 |
| SARS-CoV-2_circ_Homo_sapiens_1189 | MN908947.3 | 11488 | 11966 | + | ORF1ab | 2 | circRNA_finder | SRR11550043 |
| SARS-CoV-2_circ_Homo_sapiens_1194 | MN908947.3 | 11488 | 12137 | - | ORF1ab | 2 | find_circ | SRR11550044 |
| SARS-CoV-2_circ_Homo_sapiens_1198 | MN908947.3 | 11488 | 13729 | - | ORF1ab | 2 | find_circ | SRR11550046 |
| SARS-CoV-2_circ_Homo_sapiens_1199 | MN908947.3 | 11488 | 13729 | + | ORF1ab | 2 | circRNA_finder | SRR11550046 |
| SARS-CoV-2_circ_Homo_sapiens_1200 | MN908947.3 | 11488 | 14177 | - | ORF1ab | 2 | find_circ | SRR11550045 |
| SARS-CoV-2_circ_Homo_sapiens_1201 | MN908947.3 | 11488 | 14177 | + | ORF1ab | 2 | circRNA_finder | SRR11550045 |
| SARS-CoV-2_circ_Homo_sapiens_1203 | MN908947.3 | 11488 | 15852 | - | ORF1ab | 2 | CIRI2 | SRR11550046 |
| SARS-CoV-2_circ_Homo_sapiens_1205 | MN908947.3 | 11488 | 17134 | - | ORF1ab | 2 | find_circ | SRR11550046 |
| SARS-CoV-2_circ_Homo_sapiens_1206 | MN908947.3 | 11488 | 17134 | + | ORF1ab | 2 | circRNA_finder | SRR11550046 |
| SARS-CoV-2_circ_Homo_sapiens_1207 | MN908947.3 | 11488 | 18355 | - | ORF1ab | 2 | find_circ | SRR11550043 |
| SARS-CoV-2_circ_Homo_sapiens_1208 | MN908947.3 | 11502 | 13145 | + | ORF1ab | 2 | find_circ | SRR11550045 |
| SARS-CoV-2_circ_Homo_sapiens_1208 | MN908947.3 | 11502 | 13145 | + | ORF1ab | 2 | circRNA_finder | SRR11550045 |
| SARS-CoV-2_circ_Homo_sapiens_1211 | MN908947.3 | 11502 | 26234 | + | ORF3a;S;ORF1ab | 2 | find_circ | SRR11550046 |
| SARS-CoV-2_circ_Homo_sapiens_122 | MN908947.3 | 399 | 1916 | - | ORF1ab | 2 | find_circ | SRR11550044 |
| SARS-CoV-2_circ_Homo_sapiens_1222 | MN908947.3 | 11638 | 12011 | - | ORF1ab | 2 | find_circ | SRR11550045 |
| SARS-CoV-2_circ_Homo_sapiens_1223 | MN908947.3 | 11638 | 12011 | + | ORF1ab | 2 | circRNA_finder | SRR11550045 |
| SARS-CoV-2_circ_Homo_sapiens_1224 | MN908947.3 | 11638 | 12084 | - | ORF1ab | 2 | find_circ | SRR11550043 |
| SARS-CoV-2_circ_Homo_sapiens_1224 | MN908947.3 | 11638 | 12084 | - | ORF1ab | 2 | find_circ | SRR11550045 |
| SARS-CoV-2_circ_Homo_sapiens_123 | MN908947.3 | 399 | 1916 | + | ORF1ab | 2 | circRNA_finder | SRR11550044 |
| SARS-CoV-2_circ_Homo_sapiens_1236 | MN908947.3 | 11645 | 12137 | - | ORF1ab | 2 | find_circ | SRR11550045 |
| SARS-CoV-2_circ_Homo_sapiens_1237 | MN908947.3 | 11645 | 12137 | + | ORF1ab | 2 | circRNA_finder | SRR11550046 |
| SARS-CoV-2_circ_Homo_sapiens_1239 | MN908947.3 | 11645 | 12773 | - | ORF1ab | 2 | find_circ | SRR11550046 |
| SARS-CoV-2_circ_Homo_sapiens_124 | MN908947.3 | 399 | 5141 | - | ORF1ab | 2 | find_circ | SRR11550046 |
| SARS-CoV-2_circ_Homo_sapiens_1240 | MN908947.3 | 11645 | 12951 | - | ORF1ab | 2 | find_circ | SRR11550043 |
| SARS-CoV-2_circ_Homo_sapiens_1241 | MN908947.3 | 11645 | 13494 | - | ORF1ab | 2 | find_circ | SRR11550044 |
| SARS-CoV-2_circ_Homo_sapiens_1242 | MN908947.3 | 11645 | 13494 | + | ORF1ab | 2 | circRNA_finder | SRR11550044 |
| SARS-CoV-2_circ_Homo_sapiens_1245 | MN908947.3 | 11645 | 14572 | - | ORF1ab | 2 | find_circ | SRR11550046 |
| SARS-CoV-2_circ_Homo_sapiens_1250 | MN908947.3 | 11705 | 11930 | - | ORF1ab | 2 | find_circ | SRR11550044 |
| SARS-CoV-2_circ_Homo_sapiens_1254 | MN908947.3 | 11705 | 12035 | - | ORF1ab | 2 | find_circ | SRR11550046 |
| SARS-CoV-2_circ_Homo_sapiens_1255 | MN908947.3 | 11705 | 12084 | - | ORF1ab | 2 | find_circ | SRR11550044 |
| SARS-CoV-2_circ_Homo_sapiens_1256 | MN908947.3 | 11705 | 12131 | - | ORF1ab | 2 | find_circ | SRR11550043 |
| SARS-CoV-2_circ_Homo_sapiens_1258 | MN908947.3 | 11705 | 12152 | - | ORF1ab | 2 | find_circ | SRR11550044 |
| SARS-CoV-2_circ_Homo_sapiens_127 | MN908947.3 | 456 | 1472 | - | ORF1ab | 2 | find_circ | SRR11550043 |
| SARS-CoV-2_circ_Homo_sapiens_1271 | MN908947.3 | 11709 | 12037 | + | ORF1ab | 2 | find_circ | SRR11550046 |
| SARS-CoV-2_circ_Homo_sapiens_1272 | MN908947.3 | 11709 | 12110 | + | ORF1ab | 2 | find_circ | SRR11550044 |
| SARS-CoV-2_circ_Homo_sapiens_1273 | MN908947.3 | 11709 | 12599 | + | ORF1ab | 2 | circRNA_finder | SRR11550045 |
| SARS-CoV-2_circ_Homo_sapiens_1273 | MN908947.3 | 11709 | 12599 | + | ORF1ab | 2 | find_circ | SRR11550045 |
| SARS-CoV-2_circ_Homo_sapiens_1274 | MN908947.3 | 11709 | 13389 | + | ORF1ab | 2 | circRNA_finder | SRR11550045 |
| SARS-CoV-2_circ_Homo_sapiens_1274 | MN908947.3 | 11709 | 13389 | + | ORF1ab | 2 | find_circ | SRR11550045 |
| SARS-CoV-2_circ_Homo_sapiens_1277 | MN908947.3 | 11709 | 17095 | + | ORF1ab | 2 | CIRI2 | SRR11550043 |
| SARS-CoV-2_circ_Homo_sapiens_1278 | MN908947.3 | 11817 | 12084 | - | ORF1ab | 2 | CIRI2 | SRR11550044 |
| SARS-CoV-2_circ_Homo_sapiens_128 | MN908947.3 | 456 | 1472 | + | ORF1ab | 2 | circRNA_finder | SRR11550043 |
| SARS-CoV-2_circ_Homo_sapiens_1280 | MN908947.3 | 11817 | 12115 | - | ORF1ab | 2 | CIRI2 | SRR11550046 |
| SARS-CoV-2_circ_Homo_sapiens_1288 | MN908947.3 | 11877 | 24276 | + | S;ORF1ab | 2 | CIRI2 | SRR11550044 |
| SARS-CoV-2_circ_Homo_sapiens_1289 | MN908947.3 | 11889 | 12768 | + | ORF1ab | 2 | circRNA_finder | SRR11550045 |
| SARS-CoV-2_circ_Homo_sapiens_1289 | MN908947.3 | 11889 | 12768 | + | ORF1ab | 2 | find_circ | SRR11550045 |
| SARS-CoV-2_circ_Homo_sapiens_129 | MN908947.3 | 527 | 687 | + | ORF1ab | 2 | find_circ | SRR11550045 |
| SARS-CoV-2_circ_Homo_sapiens_1290 | MN908947.3 | 11889 | 13084 | + | ORF1ab | 2 | find_circ | SRR11550043 |
| SARS-CoV-2_circ_Homo_sapiens_1291 | MN908947.3 | 11949 | 13445 | - | ORF1ab | 2 | find_circ | SRR11550045 |
| SARS-CoV-2_circ_Homo_sapiens_1292 | MN908947.3 | 11967 | 13256 | + | ORF1ab | 2 | find_circ | SRR11550046 |
| SARS-CoV-2_circ_Homo_sapiens_1293 | MN908947.3 | 11967 | 13458 | + | ORF1ab | 2 | find_circ | SRR11550045 |
| SARS-CoV-2_circ_Homo_sapiens_1294 | MN908947.3 | 11967 | 16776 | + | ORF1ab | 2 | find_circ | SRR11550045 |
| SARS-CoV-2_circ_Homo_sapiens_1294 | MN908947.3 | 11967 | 16776 | + | ORF1ab | 2 | circRNA_finder | SRR11550045 |
| SARS-CoV-2_circ_Homo_sapiens_1298 | MN908947.3 | 12044 | 15049 | - | ORF1ab | 2 | find_circ | SRR11550046 |
| SARS-CoV-2_circ_Homo_sapiens_1299 | MN908947.3 | 12044 | 15049 | + | ORF1ab | 2 | circRNA_finder | SRR11550046 |
| SARS-CoV-2_circ_Homo_sapiens_1301 | MN908947.3 | 12050 | 12951 | - | ORF1ab | 2 | find_circ | SRR11550045 |
| SARS-CoV-2_circ_Homo_sapiens_1303 | MN908947.3 | 12063 | 12646 | + | ORF1ab | 2 | find_circ | SRR11550045 |
| SARS-CoV-2_circ_Homo_sapiens_1304 | MN908947.3 | 12063 | 16699 | + | ORF1ab | 2 | find_circ | SRR11550044 |
| SARS-CoV-2_circ_Homo_sapiens_1305 | MN908947.3 | 12063 | 18676 | + | ORF1ab | 2 | find_circ | SRR11550044 |
| SARS-CoV-2_circ_Homo_sapiens_1312 | MN908947.3 | 12085 | 14876 | - | ORF1ab | 2 | CIRI2 | SRR11550045 |
| SARS-CoV-2_circ_Homo_sapiens_1316 | MN908947.3 | 12085 | 17092 | - | ORF1ab | 2 | find_circ | SRR11550046 |
| SARS-CoV-2_circ_Homo_sapiens_132 | MN908947.3 | 527 | 1244 | + | ORF1ab | 2 | find_circ | SRR11550046 |
| SARS-CoV-2_circ_Homo_sapiens_1322 | MN908947.3 | 12093 | 13385 | + | ORF1ab | 2 | find_circ | SRR11550045 |
| SARS-CoV-2_circ_Homo_sapiens_1329 | MN908947.3 | 12132 | 12599 | + | ORF1ab | 2 | find_circ | SRR11550046 |
| SARS-CoV-2_circ_Homo_sapiens_1329 | MN908947.3 | 12132 | 12599 | + | ORF1ab | 2 | find_circ | SRR11550045 |
| SARS-CoV-2_circ_Homo_sapiens_1330 | MN908947.3 | 12132 | 12768 | + | ORF1ab | 2 | circRNA_finder | SRR11550045 |
| SARS-CoV-2_circ_Homo_sapiens_1331 | MN908947.3 | 12132 | 12801 | + | ORF1ab | 2 | circRNA_finder | SRR11550043 |
| SARS-CoV-2_circ_Homo_sapiens_1331 | MN908947.3 | 12132 | 12801 | + | ORF1ab | 2 | find_circ | SRR11550043 |
| SARS-CoV-2_circ_Homo_sapiens_1332 | MN908947.3 | 12132 | 13012 | + | ORF1ab | 2 | find_circ | SRR11550045 |
| SARS-CoV-2_circ_Homo_sapiens_1335 | MN908947.3 | 12132 | 13492 | + | ORF1ab | 2 | find_circ | SRR11550045 |
| SARS-CoV-2_circ_Homo_sapiens_1336 | MN908947.3 | 12132 | 13925 | + | ORF1ab | 2 | find_circ | SRR11550045 |
| SARS-CoV-2_circ_Homo_sapiens_1343 | MN908947.3 | 12153 | 12599 | + | ORF1ab | 2 | find_circ | SRR11550045 |
| SARS-CoV-2_circ_Homo_sapiens_1346 | MN908947.3 | 12153 | 12801 | + | ORF1ab | 2 | find_circ | SRR11550046 |
| SARS-CoV-2_circ_Homo_sapiens_1346 | MN908947.3 | 12153 | 12801 | + | ORF1ab | 2 | circRNA_finder | SRR11550046 |
| SARS-CoV-2_circ_Homo_sapiens_1347 | MN908947.3 | 12153 | 12816 | + | ORF1ab | 2 | find_circ | SRR11550046 |
| SARS-CoV-2_circ_Homo_sapiens_1349 | MN908947.3 | 12153 | 12937 | + | ORF1ab | 2 | find_circ | SRR11550043 |
| SARS-CoV-2_circ_Homo_sapiens_1350 | MN908947.3 | 12153 | 13028 | + | ORF1ab | 2 | find_circ | SRR11550045 |
| SARS-CoV-2_circ_Homo_sapiens_1352 | MN908947.3 | 12153 | 13458 | + | ORF1ab | 2 | circRNA_finder | SRR11550045 |
| SARS-CoV-2_circ_Homo_sapiens_1352 | MN908947.3 | 12153 | 13458 | + | ORF1ab | 2 | find_circ | SRR11550045 |
| SARS-CoV-2_circ_Homo_sapiens_1353 | MN908947.3 | 12153 | 13479 | + | ORF1ab | 2 | find_circ | SRR11550043 |
| SARS-CoV-2_circ_Homo_sapiens_1357 | MN908947.3 | 12153 | 15242 | + | ORF1ab | 2 | find_circ | SRR11550046 |
| SARS-CoV-2_circ_Homo_sapiens_1358 | MN908947.3 | 12153 | 16500 | + | ORF1ab | 2 | find_circ | SRR11550045 |
| SARS-CoV-2_circ_Homo_sapiens_1358 | MN908947.3 | 12153 | 16500 | + | ORF1ab | 2 | circRNA_finder | SRR11550045 |
| SARS-CoV-2_circ_Homo_sapiens_1359 | MN908947.3 | 12153 | 23567 | + | S;ORF1ab | 2 | find_circ | SRR11550045 |
| SARS-CoV-2_circ_Homo_sapiens_136 | MN908947.3 | 527 | 1576 | + | ORF1ab | 2 | find_circ | SRR11550046 |
| SARS-CoV-2_circ_Homo_sapiens_1360 | MN908947.3 | 12164 | 12910 | + | ORF1ab | 2 | find_circ | SRR11550043 |
| SARS-CoV-2_circ_Homo_sapiens_1362 | MN908947.3 | 12189 | 12805 | + | ORF1ab | 2 | find_circ | SRR11550046 |
| SARS-CoV-2_circ_Homo_sapiens_1363 | MN908947.3 | 12189 | 13458 | + | ORF1ab | 2 | find_circ | SRR11550043 |
| SARS-CoV-2_circ_Homo_sapiens_1366 | MN908947.3 | 12203 | 13483 | + | ORF1ab | 2 | find_circ | SRR11550046 |
| SARS-CoV-2_circ_Homo_sapiens_1371 | MN908947.3 | 12209 | 17657 | + | ORF1ab | 2 | find_circ | SRR11550045 |
| SARS-CoV-2_circ_Homo_sapiens_1372 | MN908947.3 | 12261 | 12595 | - | ORF1ab | 2 | find_circ | SRR11550046 |
| SARS-CoV-2_circ_Homo_sapiens_1373 | MN908947.3 | 12261 | 12623 | - | ORF1ab | 2 | find_circ | SRR11550045 |
| SARS-CoV-2_circ_Homo_sapiens_1378 | MN908947.3 | 12266 | 12797 | + | ORF1ab | 2 | find_circ | SRR11550044 |
| SARS-CoV-2_circ_Homo_sapiens_1380 | MN908947.3 | 12275 | 12929 | + | ORF1ab | 2 | CIRI2 | SRR11550046 |
| SARS-CoV-2_circ_Homo_sapiens_1381 | MN908947.3 | 12288 | 12646 | + | ORF1ab | 2 | circRNA_finder | SRR11550044 |
| SARS-CoV-2_circ_Homo_sapiens_1384 | MN908947.3 | 12288 | 13458 | + | ORF1ab | 2 | find_circ | SRR11550044 |
| SARS-CoV-2_circ_Homo_sapiens_139 | MN908947.3 | 527 | 3337 | + | ORF1ab | 2 | find_circ | SRR11550045 |
| SARS-CoV-2_circ_Homo_sapiens_139 | MN908947.3 | 527 | 3337 | + | ORF1ab | 2 | find_circ | SRR11550043 |
| SARS-CoV-2_circ_Homo_sapiens_139 | MN908947.3 | 527 | 3337 | + | ORF1ab | 2 | circRNA_finder | SRR11550045 |
| SARS-CoV-2_circ_Homo_sapiens_139 | MN908947.3 | 527 | 3337 | + | ORF1ab | 2 | find_circ | SRR11550046 |
| SARS-CoV-2_circ_Homo_sapiens_139 | MN908947.3 | 527 | 3337 | + | ORF1ab | 2 | circRNA_finder | SRR11550043 |
| SARS-CoV-2_circ_Homo_sapiens_139 | MN908947.3 | 527 | 3337 | + | ORF1ab | 2 | circRNA_finder | SRR11550046 |
| SARS-CoV-2_circ_Homo_sapiens_1391 | MN908947.3 | 12309 | 12777 | - | ORF1ab | 2 | CIRI2 | SRR11550046 |
| SARS-CoV-2_circ_Homo_sapiens_1393 | MN908947.3 | 12309 | 12951 | - | ORF1ab | 2 | find_circ | SRR11550044 |
| SARS-CoV-2_circ_Homo_sapiens_1394 | MN908947.3 | 12309 | 14572 | - | ORF1ab | 2 | find_circ | SRR11550046 |
| SARS-CoV-2_circ_Homo_sapiens_14 | MN908947.3 | 8 | 5902 | + | ORF1ab | 2 | CIRI2 | SRR11550046 |
| SARS-CoV-2_circ_Homo_sapiens_1401 | MN908947.3 | 12370 | 16728 | - | ORF1ab | 2 | find_circ | SRR11550043 |
| SARS-CoV-2_circ_Homo_sapiens_1402 | MN908947.3 | 12426 | 13385 | + | ORF1ab | 2 | find_circ | SRR11550043 |
| SARS-CoV-2_circ_Homo_sapiens_1403 | MN908947.3 | 12426 | 16500 | + | ORF1ab | 2 | find_circ | SRR11550046 |
| SARS-CoV-2_circ_Homo_sapiens_1404 | MN908947.3 | 12494 | 13357 | - | ORF1ab | 2 | find_circ | SRR11550043 |
| SARS-CoV-2_circ_Homo_sapiens_1407 | MN908947.3 | 12502 | 15395 | - | ORF1ab | 2 | find_circ | SRR11550044 |
| SARS-CoV-2_circ_Homo_sapiens_1408 | MN908947.3 | 12502 | 15395 | + | ORF1ab | 2 | circRNA_finder | SRR11550044 |
| SARS-CoV-2_circ_Homo_sapiens_1419 | MN908947.3 | 12695 | 13304 | + | ORF1ab | 2 | find_circ | SRR11550043 |
| SARS-CoV-2_circ_Homo_sapiens_1423 | MN908947.3 | 12774 | 13389 | + | ORF1ab | 2 | find_circ | SRR11550045 |
| SARS-CoV-2_circ_Homo_sapiens_1423 | MN908947.3 | 12774 | 13389 | + | ORF1ab | 2 | circRNA_finder | SRR11550046 |
| SARS-CoV-2_circ_Homo_sapiens_1424 | MN908947.3 | 12774 | 13412 | + | ORF1ab | 2 | find_circ | SRR11550045 |
| SARS-CoV-2_circ_Homo_sapiens_143 | MN908947.3 | 527 | 6921 | + | ORF1ab | 2 | find_circ | SRR11550045 |
| SARS-CoV-2_circ_Homo_sapiens_1430 | MN908947.3 | 12831 | 14575 | - | ORF1ab | 2 | find_circ | SRR11550046 |
| SARS-CoV-2_circ_Homo_sapiens_1431 | MN908947.3 | 12831 | 14575 | + | ORF1ab | 2 | circRNA_finder | SRR11550046 |
| SARS-CoV-2_circ_Homo_sapiens_1432 | MN908947.3 | 12831 | 16603 | - | ORF1ab | 2 | find_circ | SRR11550046 |
| SARS-CoV-2_circ_Homo_sapiens_1435 | MN908947.3 | 12833 | 13304 | + | ORF1ab | 2 | find_circ | SRR11550045 |
| SARS-CoV-2_circ_Homo_sapiens_144 | MN908947.3 | 527 | 11145 | + | ORF1ab | 2 | circRNA_finder | SRR11550045 |
| SARS-CoV-2_circ_Homo_sapiens_144 | MN908947.3 | 527 | 11145 | + | ORF1ab | 2 | find_circ | SRR11550045 |
| SARS-CoV-2_circ_Homo_sapiens_1445 | MN908947.3 | 12924 | 13357 | - | ORF1ab | 2 | find_circ | SRR11550044 |
| SARS-CoV-2_circ_Homo_sapiens_1446 | MN908947.3 | 12924 | 13357 | + | ORF1ab | 2 | circRNA_finder | SRR11550044 |
| SARS-CoV-2_circ_Homo_sapiens_1450 | MN908947.3 | 12953 | 13494 | - | ORF1ab | 2 | find_circ | SRR11550045 |
| SARS-CoV-2_circ_Homo_sapiens_1451 | MN908947.3 | 12953 | 13494 | + | ORF1ab | 2 | circRNA_finder | SRR11550045 |
| SARS-CoV-2_circ_Homo_sapiens_1453 | MN908947.3 | 12974 | 13334 | - | ORF1ab | 2 | CIRI2 | SRR11550045 |
| SARS-CoV-2_circ_Homo_sapiens_1456 | MN908947.3 | 12974 | 13823 | - | ORF1ab | 2 | CIRI2 | SRR11550046 |
| SARS-CoV-2_circ_Homo_sapiens_1467 | MN908947.3 | 12982 | 13389 | + | ORF1ab | 2 | find_circ | SRR11550046 |
| SARS-CoV-2_circ_Homo_sapiens_1467 | MN908947.3 | 12982 | 13389 | + | ORF1ab | 2 | circRNA_finder | SRR11550046 |
| SARS-CoV-2_circ_Homo_sapiens_147 | MN908947.3 | 534 | 1273 | + | ORF1ab | 2 | find_circ | SRR11550043 |
| SARS-CoV-2_circ_Homo_sapiens_147 | MN908947.3 | 534 | 1273 | + | ORF1ab | 2 | circRNA_finder | SRR11550043 |
| SARS-CoV-2_circ_Homo_sapiens_1470 | MN908947.3 | 12984 | 13389 | + | ORF1ab | 2 | find_circ | SRR11550044 |
| SARS-CoV-2_circ_Homo_sapiens_1470 | MN908947.3 | 12984 | 13389 | + | ORF1ab | 2 | circRNA_finder | SRR11550044 |
| SARS-CoV-2_circ_Homo_sapiens_1472 | MN908947.3 | 12984 | 13412 | + | ORF1ab | 2 | find_circ | SRR11550045 |
| SARS-CoV-2_circ_Homo_sapiens_1472 | MN908947.3 | 12984 | 13412 | + | ORF1ab | 2 | circRNA_finder | SRR11550045 |
| SARS-CoV-2_circ_Homo_sapiens_1473 | MN908947.3 | 12984 | 15201 | + | ORF1ab | 2 | CIRI2 | SRR11550043 |
| SARS-CoV-2_circ_Homo_sapiens_1475 | MN908947.3 | 12984 | 22988 | + | S;ORF1ab | 2 | circRNA_finder | SRR11550046 |
| SARS-CoV-2_circ_Homo_sapiens_1475 | MN908947.3 | 12984 | 22988 | + | S;ORF1ab | 2 | find_circ | SRR11550046 |
| SARS-CoV-2_circ_Homo_sapiens_1477 | MN908947.3 | 13000 | 13308 | + | ORF1ab | 2 | find_circ | SRR11550046 |
| SARS-CoV-2_circ_Homo_sapiens_1482 | MN908947.3 | 13005 | 13389 | + | ORF1ab | 2 | find_circ | SRR11550045 |
| SARS-CoV-2_circ_Homo_sapiens_1482 | MN908947.3 | 13005 | 13389 | + | ORF1ab | 2 | circRNA_finder | SRR11550045 |
| SARS-CoV-2_circ_Homo_sapiens_1489 | MN908947.3 | 13023 | 18247 | - | ORF1ab | 2 | find_circ | SRR11550046 |
| SARS-CoV-2_circ_Homo_sapiens_1497 | MN908947.3 | 13141 | 14535 | - | ORF1ab | 2 | find_circ | SRR11550045 |
| SARS-CoV-2_circ_Homo_sapiens_1498 | MN908947.3 | 13154 | 13458 | + | ORF1ab | 2 | find_circ | SRR11550046 |
| SARS-CoV-2_circ_Homo_sapiens_1499 | MN908947.3 | 13154 | 14853 | + | ORF1ab | 2 | find_circ | SRR11550046 |
| SARS-CoV-2_circ_Homo_sapiens_1499 | MN908947.3 | 13154 | 14853 | + | ORF1ab | 2 | circRNA_finder | SRR11550046 |
| SARS-CoV-2_circ_Homo_sapiens_15 | MN908947.3 | 8 | 6051 | + | ORF1ab | 2 | find_circ | SRR11550046 |
| SARS-CoV-2_circ_Homo_sapiens_1500 | MN908947.3 | 13154 | 14856 | + | ORF1ab | 2 | find_circ | SRR11550046 |
| SARS-CoV-2_circ_Homo_sapiens_1501 | MN908947.3 | 13154 | 17346 | + | ORF1ab | 2 | find_circ | SRR11550046 |
| SARS-CoV-2_circ_Homo_sapiens_1503 | MN908947.3 | 13154 | 19074 | + | ORF1ab | 2 | find_circ | SRR11550046 |
| SARS-CoV-2_circ_Homo_sapiens_1514 | MN908947.3 | 13500 | 13828 | - | ORF1ab | 2 | find_circ | SRR11550045 |
| SARS-CoV-2_circ_Homo_sapiens_1516 | MN908947.3 | 13500 | 14575 | - | ORF1ab | 2 | find_circ | SRR11550046 |
| SARS-CoV-2_circ_Homo_sapiens_1517 | MN908947.3 | 13500 | 14575 | + | ORF1ab | 2 | circRNA_finder | SRR11550046 |
| SARS-CoV-2_circ_Homo_sapiens_1521 | MN908947.3 | 13500 | 14767 | - | ORF1ab | 2 | find_circ | SRR11550045 |
| SARS-CoV-2_circ_Homo_sapiens_153 | MN908947.3 | 689 | 1508 | + | ORF1ab | 2 | find_circ | SRR11550043 |
| SARS-CoV-2_circ_Homo_sapiens_1530 | MN908947.3 | 13502 | 15250 | - | ORF1ab | 2 | find_circ | SRR11550046 |
| SARS-CoV-2_circ_Homo_sapiens_1530 | MN908947.3 | 13502 | 15250 | - | ORF1ab | 2 | find_circ | SRR11550045 |
| SARS-CoV-2_circ_Homo_sapiens_1531 | MN908947.3 | 13502 | 17009 | - | ORF1ab | 2 | find_circ | SRR11550045 |
| SARS-CoV-2_circ_Homo_sapiens_1532 | MN908947.3 | 13512 | 14635 | - | ORF1ab | 2 | find_circ | SRR11550045 |
| SARS-CoV-2_circ_Homo_sapiens_1535 | MN908947.3 | 13521 | 13935 | + | ORF1ab | 2 | find_circ | SRR11550045 |
| SARS-CoV-2_circ_Homo_sapiens_1536 | MN908947.3 | 13521 | 13997 | + | ORF1ab | 2 | find_circ | SRR11550045 |
| SARS-CoV-2_circ_Homo_sapiens_1542 | MN908947.3 | 13521 | 16699 | + | ORF1ab | 2 | find_circ | SRR11550045 |
| SARS-CoV-2_circ_Homo_sapiens_1543 | MN908947.3 | 13521 | 16942 | + | ORF1ab | 2 | find_circ | SRR11550046 |
| SARS-CoV-2_circ_Homo_sapiens_1545 | MN908947.3 | 13521 | 24763 | + | S;ORF1ab | 2 | find_circ | SRR11550045 |
| SARS-CoV-2_circ_Homo_sapiens_1547 | MN908947.3 | 13724 | 14494 | + | ORF1ab | 2 | circRNA_finder | SRR11550044 |
| SARS-CoV-2_circ_Homo_sapiens_1547 | MN908947.3 | 13724 | 14494 | + | ORF1ab | 2 | find_circ | SRR11550044 |
| SARS-CoV-2_circ_Homo_sapiens_155 | MN908947.3 | 689 | 3353 | + | ORF1ab | 2 | find_circ | SRR11550045 |
| SARS-CoV-2_circ_Homo_sapiens_1552 | MN908947.3 | 13836 | 14240 | + | ORF1ab | 2 | circRNA_finder | SRR11550045 |
| SARS-CoV-2_circ_Homo_sapiens_1552 | MN908947.3 | 13836 | 14240 | + | ORF1ab | 2 | find_circ | SRR11550045 |
| SARS-CoV-2_circ_Homo_sapiens_1559 | MN908947.3 | 13972 | 14572 | - | ORF1ab | 2 | find_circ | SRR11550045 |
| SARS-CoV-2_circ_Homo_sapiens_156 | MN908947.3 | 689 | 3559 | + | ORF1ab | 2 | find_circ | SRR11550043 |
| SARS-CoV-2_circ_Homo_sapiens_1561 | MN908947.3 | 13972 | 14575 | - | ORF1ab | 2 | find_circ | SRR11550046 |
| SARS-CoV-2_circ_Homo_sapiens_1562 | MN908947.3 | 13972 | 14575 | + | ORF1ab | 2 | circRNA_finder | SRR11550046 |
| SARS-CoV-2_circ_Homo_sapiens_1570 | MN908947.3 | 13976 | 14809 | + | ORF1ab | 2 | find_circ | SRR11550043 |
| SARS-CoV-2_circ_Homo_sapiens_1574 | MN908947.3 | 14178 | 14535 | - | ORF1ab | 2 | find_circ | SRR11550044 |
| SARS-CoV-2_circ_Homo_sapiens_1577 | MN908947.3 | 14178 | 14572 | - | ORF1ab | 2 | find_circ | SRR11550046 |
| SARS-CoV-2_circ_Homo_sapiens_1578 | MN908947.3 | 14178 | 14572 | + | ORF1ab | 2 | circRNA_finder | SRR11550046 |
| SARS-CoV-2_circ_Homo_sapiens_1579 | MN908947.3 | 14178 | 14575 | - | ORF1ab | 2 | find_circ | SRR11550044 |
| SARS-CoV-2_circ_Homo_sapiens_1580 | MN908947.3 | 14178 | 14575 | + | ORF1ab | 2 | circRNA_finder | SRR11550044 |
| SARS-CoV-2_circ_Homo_sapiens_1582 | MN908947.3 | 14178 | 14590 | - | ORF1ab | 2 | find_circ | SRR11550046 |
| SARS-CoV-2_circ_Homo_sapiens_1585 | MN908947.3 | 14178 | 14598 | + | ORF1ab | 2 | circRNA_finder | SRR11550046 |
| SARS-CoV-2_circ_Homo_sapiens_1589 | MN908947.3 | 14178 | 14847 | - | ORF1ab | 2 | find_circ | SRR11550046 |
| SARS-CoV-2_circ_Homo_sapiens_159 | MN908947.3 | 689 | 18582 | + | ORF1ab | 2 | find_circ | SRR11550043 |
| SARS-CoV-2_circ_Homo_sapiens_1592 | MN908947.3 | 14178 | 16422 | - | ORF1ab | 2 | find_circ | SRR11550045 |
| SARS-CoV-2_circ_Homo_sapiens_1594 | MN908947.3 | 14178 | 17184 | + | ORF1ab | 2 | circRNA_finder | SRR11550045 |
| SARS-CoV-2_circ_Homo_sapiens_1595 | MN908947.3 | 14178 | 20404 | - | ORF1ab | 2 | CIRI2 | SRR11550043 |
| SARS-CoV-2_circ_Homo_sapiens_1597 | MN908947.3 | 14178 | 27130 | - | M;E;ORF3a;S;ORF1ab | 2 | find_circ | SRR11550045 |
| SARS-CoV-2_circ_Homo_sapiens_1598 | MN908947.3 | 14178 | 27130 | + | M;E;ORF3a;S;ORF1ab | 2 | circRNA_finder | SRR11550045 |
| SARS-CoV-2_circ_Homo_sapiens_16 | MN908947.3 | 8 | 6562 | + | ORF1ab | 2 | find_circ | SRR11550045 |
| SARS-CoV-2_circ_Homo_sapiens_160 | MN908947.3 | 689 | 24139 | + | S;ORF1ab | 2 | find_circ | SRR11550046 |
| SARS-CoV-2_circ_Homo_sapiens_1605 | MN908947.3 | 14196 | 14563 | - | ORF1ab | 2 | find_circ | SRR11550046 |
| SARS-CoV-2_circ_Homo_sapiens_1607 | MN908947.3 | 14207 | 14598 | - | ORF1ab | 2 | find_circ | SRR11550046 |
| SARS-CoV-2_circ_Homo_sapiens_1608 | MN908947.3 | 14207 | 26570 | - | M;E;ORF3a;S;ORF1ab | 2 | find_circ | SRR11550043 |
| SARS-CoV-2_circ_Homo_sapiens_1610 | MN908947.3 | 14236 | 14575 | - | ORF1ab | 2 | find_circ | SRR11550045 |
| SARS-CoV-2_circ_Homo_sapiens_1614 | MN908947.3 | 14408 | 14804 | - | ORF1ab | 2 | find_circ | SRR11550045 |
| SARS-CoV-2_circ_Homo_sapiens_1614 | MN908947.3 | 14408 | 14804 | - | ORF1ab | 2 | CIRI2 | SRR11550045 |
| SARS-CoV-2_circ_Homo_sapiens_162 | MN908947.3 | 698 | 998 | - | ORF1ab | 2 | find_circ | SRR11550046 |
| SARS-CoV-2_circ_Homo_sapiens_1620 | MN908947.3 | 14408 | 17765 | - | ORF1ab | 2 | find_circ | SRR11550045 |
| SARS-CoV-2_circ_Homo_sapiens_1622 | MN908947.3 | 14412 | 17009 | + | ORF1ab | 2 | circRNA_finder | SRR11550044 |
| SARS-CoV-2_circ_Homo_sapiens_1625 | MN908947.3 | 14423 | 27130 | - | M;E;ORF3a;S;ORF1ab | 2 | find_circ | SRR11550045 |
| SARS-CoV-2_circ_Homo_sapiens_1626 | MN908947.3 | 14465 | 15593 | + | ORF1ab | 2 | circRNA_finder | SRR11550044 |
| SARS-CoV-2_circ_Homo_sapiens_1626 | MN908947.3 | 14465 | 15593 | + | ORF1ab | 2 | find_circ | SRR11550044 |
| SARS-CoV-2_circ_Homo_sapiens_1627 | MN908947.3 | 14465 | 17533 | + | ORF1ab | 2 | find_circ | SRR11550044 |
| SARS-CoV-2_circ_Homo_sapiens_1627 | MN908947.3 | 14465 | 17533 | + | ORF1ab | 2 | circRNA_finder | SRR11550044 |
| SARS-CoV-2_circ_Homo_sapiens_163 | MN908947.3 | 698 | 1213 | - | ORF1ab | 2 | find_circ | SRR11550045 |
| SARS-CoV-2_circ_Homo_sapiens_1631 | MN908947.3 | 14609 | 15198 | + | ORF1ab | 2 | circRNA_finder | SRR11550043 |
| SARS-CoV-2_circ_Homo_sapiens_1631 | MN908947.3 | 14609 | 15198 | + | ORF1ab | 2 | circRNA_finder | SRR11550046 |
| SARS-CoV-2_circ_Homo_sapiens_1631 | MN908947.3 | 14609 | 15198 | + | ORF1ab | 2 | circRNA_finder | SRR11550044 |
| SARS-CoV-2_circ_Homo_sapiens_1631 | MN908947.3 | 14609 | 15198 | + | ORF1ab | 2 | find_circ | SRR11550046 |
| SARS-CoV-2_circ_Homo_sapiens_1631 | MN908947.3 | 14609 | 15198 | + | ORF1ab | 2 | find_circ | SRR11550044 |
| SARS-CoV-2_circ_Homo_sapiens_1632 | MN908947.3 | 14609 | 15201 | + | ORF1ab | 2 | circRNA_finder | SRR11550045 |
| SARS-CoV-2_circ_Homo_sapiens_1632 | MN908947.3 | 14609 | 15201 | + | ORF1ab | 2 | find_circ | SRR11550043 |
| SARS-CoV-2_circ_Homo_sapiens_1632 | MN908947.3 | 14609 | 15201 | + | ORF1ab | 2 | circRNA_finder | SRR11550043 |
| SARS-CoV-2_circ_Homo_sapiens_1632 | MN908947.3 | 14609 | 15201 | + | ORF1ab | 2 | find_circ | SRR11550045 |
| SARS-CoV-2_circ_Homo_sapiens_1635 | MN908947.3 | 14609 | 18016 | + | ORF1ab | 2 | circRNA_finder | SRR11550045 |
| SARS-CoV-2_circ_Homo_sapiens_1635 | MN908947.3 | 14609 | 18016 | + | ORF1ab | 2 | find_circ | SRR11550045 |
| SARS-CoV-2_circ_Homo_sapiens_1637 | MN908947.3 | 14619 | 16948 | - | ORF1ab | 2 | find_circ | SRR11550043 |
| SARS-CoV-2_circ_Homo_sapiens_164 | MN908947.3 | 698 | 1220 | - | ORF1ab | 2 | find_circ | SRR11550045 |
| SARS-CoV-2_circ_Homo_sapiens_1642 | MN908947.3 | 14689 | 15298 | - | ORF1ab | 2 | find_circ | SRR11550044 |
| SARS-CoV-2_circ_Homo_sapiens_1643 | MN908947.3 | 14689 | 15298 | + | ORF1ab | 2 | circRNA_finder | SRR11550044 |
| SARS-CoV-2_circ_Homo_sapiens_1645 | MN908947.3 | 14793 | 16507 | + | ORF1ab | 2 | find_circ | SRR11550046 |
| SARS-CoV-2_circ_Homo_sapiens_165 | MN908947.3 | 698 | 1283 | - | ORF1ab | 2 | find_circ | SRR11550046 |
| SARS-CoV-2_circ_Homo_sapiens_1652 | MN908947.3 | 14914 | 16427 | - | ORF1ab | 2 | find_circ | SRR11550046 |
| SARS-CoV-2_circ_Homo_sapiens_1660 | MN908947.3 | 15020 | 26203 | - | ORF3a;S;ORF1ab | 2 | find_circ | SRR11550045 |
| SARS-CoV-2_circ_Homo_sapiens_1663 | MN908947.3 | 15038 | 28092 | - | ORF6;M;E;ORF3a;S;ORF8;ORF7a;ORF7b;ORF1ab | 2 | find_circ | SRR11550044 |
| SARS-CoV-2_circ_Homo_sapiens_1672 | MN908947.3 | 15216 | 17533 | + | ORF1ab | 2 | circRNA_finder | SRR11550046 |
| SARS-CoV-2_circ_Homo_sapiens_1672 | MN908947.3 | 15216 | 17533 | + | ORF1ab | 2 | find_circ | SRR11550046 |
| SARS-CoV-2_circ_Homo_sapiens_1673 | MN908947.3 | 15216 | 18073 | + | ORF1ab | 2 | find_circ | SRR11550045 |
| SARS-CoV-2_circ_Homo_sapiens_1677 | MN908947.3 | 15269 | 15918 | + | ORF1ab | 2 | circRNA_finder | SRR11550043 |
| SARS-CoV-2_circ_Homo_sapiens_1677 | MN908947.3 | 15269 | 15918 | + | ORF1ab | 2 | find_circ | SRR11550043 |
| SARS-CoV-2_circ_Homo_sapiens_1681 | MN908947.3 | 15280 | 16307 | - | ORF1ab | 2 | find_circ | SRR11550043 |
| SARS-CoV-2_circ_Homo_sapiens_1682 | MN908947.3 | 15280 | 16307 | + | ORF1ab | 2 | circRNA_finder | SRR11550043 |
| SARS-CoV-2_circ_Homo_sapiens_1686 | MN908947.3 | 15280 | 18813 | - | ORF1ab | 2 | find_circ | SRR11550046 |
| SARS-CoV-2_circ_Homo_sapiens_1687 | MN908947.3 | 15280 | 18813 | + | ORF1ab | 2 | circRNA_finder | SRR11550046 |
| SARS-CoV-2_circ_Homo_sapiens_1688 | MN908947.3 | 15325 | 15852 | - | ORF1ab | 2 | find_circ | SRR11550045 |
| SARS-CoV-2_circ_Homo_sapiens_1689 | MN908947.3 | 15325 | 15852 | + | ORF1ab | 2 | circRNA_finder | SRR11550045 |
| SARS-CoV-2_circ_Homo_sapiens_169 | MN908947.3 | 698 | 1472 | - | ORF1ab | 2 | find_circ | SRR11550046 |
| SARS-CoV-2_circ_Homo_sapiens_1695 | MN908947.3 | 15333 | 16312 | + | ORF1ab | 2 | find_circ | SRR11550044 |
| SARS-CoV-2_circ_Homo_sapiens_1696 | MN908947.3 | 15333 | 16474 | + | ORF1ab | 2 | find_circ | SRR11550045 |
| SARS-CoV-2_circ_Homo_sapiens_17 | MN908947.3 | 8 | 6712 | + | ORF1ab | 2 | find_circ | SRR11550044 |
| SARS-CoV-2_circ_Homo_sapiens_1702 | MN908947.3 | 15347 | 15745 | - | ORF1ab | 2 | find_circ | SRR11550044 |
| SARS-CoV-2_circ_Homo_sapiens_1704 | MN908947.3 | 15402 | 15726 | + | ORF1ab | 2 | circRNA_finder | SRR11550046 |
| SARS-CoV-2_circ_Homo_sapiens_1704 | MN908947.3 | 15402 | 15726 | + | ORF1ab | 2 | find_circ | SRR11550046 |
| SARS-CoV-2_circ_Homo_sapiens_1704 | MN908947.3 | 15402 | 15726 | + | ORF1ab | 2 | find_circ | SRR11550044 |
| SARS-CoV-2_circ_Homo_sapiens_1704 | MN908947.3 | 15402 | 15726 | + | ORF1ab | 2 | circRNA_finder | SRR11550044 |
| SARS-CoV-2_circ_Homo_sapiens_1705 | MN908947.3 | 15402 | 15729 | + | ORF1ab | 2 | find_circ | SRR11550045 |
| SARS-CoV-2_circ_Homo_sapiens_1707 | MN908947.3 | 15402 | 15733 | + | ORF1ab | 2 | circRNA_finder | SRR11550046 |
| SARS-CoV-2_circ_Homo_sapiens_1707 | MN908947.3 | 15402 | 15733 | + | ORF1ab | 2 | find_circ | SRR11550046 |
| SARS-CoV-2_circ_Homo_sapiens_171 | MN908947.3 | 698 | 1479 | - | ORF1ab | 2 | find_circ | SRR11550046 |
| SARS-CoV-2_circ_Homo_sapiens_1710 | MN908947.3 | 15402 | 16297 | + | ORF1ab | 2 | find_circ | SRR11550043 |
| SARS-CoV-2_circ_Homo_sapiens_1710 | MN908947.3 | 15402 | 16297 | + | ORF1ab | 2 | circRNA_finder | SRR11550043 |
| SARS-CoV-2_circ_Homo_sapiens_1713 | MN908947.3 | 15407 | 15574 | + | ORF1ab | 2 | find_circ | SRR11550043 |
| SARS-CoV-2_circ_Homo_sapiens_1714 | MN908947.3 | 15407 | 15788 | + | ORF1ab | 2 | circRNA_finder | SRR11550045 |
| SARS-CoV-2_circ_Homo_sapiens_1714 | MN908947.3 | 15407 | 15788 | + | ORF1ab | 2 | find_circ | SRR11550045 |
| SARS-CoV-2_circ_Homo_sapiens_1717 | MN908947.3 | 15407 | 16297 | + | ORF1ab | 2 | find_circ | SRR11550046 |
| SARS-CoV-2_circ_Homo_sapiens_1717 | MN908947.3 | 15407 | 16297 | + | ORF1ab | 2 | circRNA_finder | SRR11550046 |
| SARS-CoV-2_circ_Homo_sapiens_1718 | MN908947.3 | 15407 | 16315 | + | ORF1ab | 2 | find_circ | SRR11550046 |
| SARS-CoV-2_circ_Homo_sapiens_1718 | MN908947.3 | 15407 | 16315 | + | ORF1ab | 2 | circRNA_finder | SRR11550046 |
| SARS-CoV-2_circ_Homo_sapiens_172 | MN908947.3 | 698 | 1527 | - | ORF1ab | 2 | find_circ | SRR11550045 |
| SARS-CoV-2_circ_Homo_sapiens_1723 | MN908947.3 | 15560 | 16422 | - | ORF1ab | 2 | find_circ | SRR11550045 |
| SARS-CoV-2_circ_Homo_sapiens_1724 | MN908947.3 | 15560 | 16422 | + | ORF1ab | 2 | circRNA_finder | SRR11550045 |
| SARS-CoV-2_circ_Homo_sapiens_173 | MN908947.3 | 698 | 1600 | - | ORF1ab | 2 | find_circ | SRR11550046 |
| SARS-CoV-2_circ_Homo_sapiens_1732 | MN908947.3 | 15744 | 16312 | + | ORF1ab | 2 | find_circ | SRR11550044 |
| SARS-CoV-2_circ_Homo_sapiens_1732 | MN908947.3 | 15744 | 16312 | + | ORF1ab | 2 | circRNA_finder | SRR11550044 |
| SARS-CoV-2_circ_Homo_sapiens_1737 | MN908947.3 | 15853 | 16158 | - | ORF1ab | 2 | find_circ | SRR11550046 |
| SARS-CoV-2_circ_Homo_sapiens_1738 | MN908947.3 | 15853 | 16158 | + | ORF1ab | 2 | circRNA_finder | SRR11550046 |
| SARS-CoV-2_circ_Homo_sapiens_1739 | MN908947.3 | 15853 | 16162 | - | ORF1ab | 2 | find_circ | SRR11550045 |
| SARS-CoV-2_circ_Homo_sapiens_174 | MN908947.3 | 698 | 2535 | - | ORF1ab | 2 | find_circ | SRR11550044 |
| SARS-CoV-2_circ_Homo_sapiens_1740 | MN908947.3 | 15853 | 16162 | + | ORF1ab | 2 | circRNA_finder | SRR11550045 |
| SARS-CoV-2_circ_Homo_sapiens_1741 | MN908947.3 | 15853 | 16207 | - | ORF1ab | 2 | find_circ | SRR11550044 |
| SARS-CoV-2_circ_Homo_sapiens_1742 | MN908947.3 | 15853 | 16288 | - | ORF1ab | 2 | find_circ | SRR11550043 |
| SARS-CoV-2_circ_Homo_sapiens_1743 | MN908947.3 | 15853 | 16288 | + | ORF1ab | 2 | circRNA_finder | SRR11550043 |
| SARS-CoV-2_circ_Homo_sapiens_1748 | MN908947.3 | 15925 | 16270 | - | ORF1ab | 2 | find_circ | SRR11550045 |
| SARS-CoV-2_circ_Homo_sapiens_175 | MN908947.3 | 698 | 14407 | + | ORF1ab | 2 | circRNA_finder | SRR11550045 |
| SARS-CoV-2_circ_Homo_sapiens_1754 | MN908947.3 | 16028 | 16786 | + | ORF1ab | 2 | find_circ | SRR11550043 |
| SARS-CoV-2_circ_Homo_sapiens_1757 | MN908947.3 | 16028 | 18897 | + | ORF1ab | 2 | find_circ | SRR11550044 |
| SARS-CoV-2_circ_Homo_sapiens_1759 | MN908947.3 | 16093 | 26241 | - | ORF3a;S;ORF1ab | 2 | find_circ | SRR11550045 |
| SARS-CoV-2_circ_Homo_sapiens_1767 | MN908947.3 | 16361 | 16911 | + | ORF1ab | 2 | find_circ | SRR11550046 |
| SARS-CoV-2_circ_Homo_sapiens_1767 | MN908947.3 | 16361 | 16911 | + | ORF1ab | 2 | circRNA_finder | SRR11550046 |
| SARS-CoV-2_circ_Homo_sapiens_1772 | MN908947.3 | 16397 | 16974 | + | ORF1ab | 2 | CIRI2 | SRR11550046 |
| SARS-CoV-2_circ_Homo_sapiens_1775 | MN908947.3 | 16397 | 18016 | + | ORF1ab | 2 | circRNA_finder | SRR11550043 |
| SARS-CoV-2_circ_Homo_sapiens_1775 | MN908947.3 | 16397 | 18016 | + | ORF1ab | 2 | find_circ | SRR11550043 |
| SARS-CoV-2_circ_Homo_sapiens_1775 | MN908947.3 | 16397 | 18016 | + | ORF1ab | 2 | find_circ | SRR11550046 |
| SARS-CoV-2_circ_Homo_sapiens_1775 | MN908947.3 | 16397 | 18016 | + | ORF1ab | 2 | circRNA_finder | SRR11550046 |
| SARS-CoV-2_circ_Homo_sapiens_1776 | MN908947.3 | 16397 | 18897 | + | ORF1ab | 2 | find_circ | SRR11550045 |
| SARS-CoV-2_circ_Homo_sapiens_1776 | MN908947.3 | 16397 | 18897 | + | ORF1ab | 2 | circRNA_finder | SRR11550045 |
| SARS-CoV-2_circ_Homo_sapiens_1780 | MN908947.3 | 16436 | 16974 | + | ORF1ab | 2 | find_circ | SRR11550043 |
| SARS-CoV-2_circ_Homo_sapiens_1780 | MN908947.3 | 16436 | 16974 | + | ORF1ab | 2 | circRNA_finder | SRR11550043 |
| SARS-CoV-2_circ_Homo_sapiens_1782 | MN908947.3 | 16536 | 18016 | + | ORF1ab | 2 | find_circ | SRR11550046 |
| SARS-CoV-2_circ_Homo_sapiens_1783 | MN908947.3 | 16552 | 16750 | - | ORF1ab | 2 | find_circ | SRR11550046 |
| SARS-CoV-2_circ_Homo_sapiens_1783 | MN908947.3 | 16552 | 16750 | - | ORF1ab | 2 | find_circ | SRR11550045 |
| SARS-CoV-2_circ_Homo_sapiens_1786 | MN908947.3 | 16552 | 16988 | - | ORF1ab | 2 | find_circ | SRR11550045 |
| SARS-CoV-2_circ_Homo_sapiens_1787 | MN908947.3 | 16552 | 16988 | + | ORF1ab | 2 | circRNA_finder | SRR11550045 |
| SARS-CoV-2_circ_Homo_sapiens_1788 | MN908947.3 | 16552 | 17001 | - | ORF1ab | 2 | find_circ | SRR11550044 |
| SARS-CoV-2_circ_Homo_sapiens_1792 | MN908947.3 | 16604 | 18016 | + | ORF1ab | 2 | circRNA_finder | SRR11550045 |
| SARS-CoV-2_circ_Homo_sapiens_1794 | MN908947.3 | 16611 | 24626 | - | S;ORF1ab | 2 | find_circ | SRR11550046 |
| SARS-CoV-2_circ_Homo_sapiens_1797 | MN908947.3 | 16668 | 18656 | - | ORF1ab | 2 | find_circ | SRR11550043 |
| SARS-CoV-2_circ_Homo_sapiens_1800 | MN908947.3 | 16717 | 17569 | - | ORF1ab | 2 | find_circ | SRR11550043 |
| SARS-CoV-2_circ_Homo_sapiens_1801 | MN908947.3 | 16717 | 17569 | + | ORF1ab | 2 | circRNA_finder | SRR11550043 |
| SARS-CoV-2_circ_Homo_sapiens_1807 | MN908947.3 | 16751 | 18876 | - | ORF1ab | 2 | CIRI2 | SRR11550043 |
| SARS-CoV-2_circ_Homo_sapiens_1816 | MN908947.3 | 16800 | 20203 | - | ORF1ab | 2 | find_circ | SRR11550046 |
| SARS-CoV-2_circ_Homo_sapiens_1819 | MN908947.3 | 16944 | 17346 | + | ORF1ab | 2 | find_circ | SRR11550046 |
| SARS-CoV-2_circ_Homo_sapiens_1821 | MN908947.3 | 16949 | 29036 | - | ORF6;M;E;ORF3a;S;N;ORF8;ORF7a;ORF7b;ORF1ab | 2 | find_circ | SRR11550045 |
| SARS-CoV-2_circ_Homo_sapiens_1831 | MN908947.3 | 17232 | 23561 | + | S;ORF1ab | 2 | CIRI2 | SRR11550043 |
| SARS-CoV-2_circ_Homo_sapiens_1832 | MN908947.3 | 17342 | 18016 | + | ORF1ab | 2 | circRNA_finder | SRR11550045 |
| SARS-CoV-2_circ_Homo_sapiens_1832 | MN908947.3 | 17342 | 18016 | + | ORF1ab | 2 | find_circ | SRR11550043 |
| SARS-CoV-2_circ_Homo_sapiens_1832 | MN908947.3 | 17342 | 18016 | + | ORF1ab | 2 | circRNA_finder | SRR11550043 |
| SARS-CoV-2_circ_Homo_sapiens_1833 | MN908947.3 | 17342 | 18815 | + | ORF1ab | 2 | find_circ | SRR11550046 |
| SARS-CoV-2_circ_Homo_sapiens_1833 | MN908947.3 | 17342 | 18815 | + | ORF1ab | 2 | circRNA_finder | SRR11550046 |
| SARS-CoV-2_circ_Homo_sapiens_1835 | MN908947.3 | 17342 | 18897 | + | ORF1ab | 2 | find_circ | SRR11550046 |
| SARS-CoV-2_circ_Homo_sapiens_1841 | MN908947.3 | 17406 | 18309 | + | ORF1ab | 2 | find_circ | SRR11550046 |
| SARS-CoV-2_circ_Homo_sapiens_1844 | MN908947.3 | 17406 | 18815 | + | ORF1ab | 2 | find_circ | SRR11550046 |
| SARS-CoV-2_circ_Homo_sapiens_1851 | MN908947.3 | 17411 | 18355 | - | ORF1ab | 2 | find_circ | SRR11550045 |
| SARS-CoV-2_circ_Homo_sapiens_1856 | MN908947.3 | 17431 | 18247 | - | ORF1ab | 2 | find_circ | SRR11550046 |
| SARS-CoV-2_circ_Homo_sapiens_1861 | MN908947.3 | 17466 | 20940 | - | ORF1ab | 2 | find_circ | SRR11550045 |
| SARS-CoV-2_circ_Homo_sapiens_1862 | MN908947.3 | 17466 | 20940 | + | ORF1ab | 2 | circRNA_finder | SRR11550045 |
| SARS-CoV-2_circ_Homo_sapiens_1863 | MN908947.3 | 17466 | 29066 | - | ORF6;M;E;ORF3a;S;N;ORF8;ORF7a;ORF7b;ORF1ab | 2 | find_circ | SRR11550045 |
| SARS-CoV-2_circ_Homo_sapiens_1865 | MN908947.3 | 17617 | 18234 | + | ORF1ab | 2 | find_circ | SRR11550044 |
| SARS-CoV-2_circ_Homo_sapiens_1878 | MN908947.3 | 17883 | 18355 | - | ORF1ab | 2 | find_circ | SRR11550045 |
| SARS-CoV-2_circ_Homo_sapiens_188 | MN908947.3 | 752 | 1600 | - | ORF1ab | 2 | find_circ | SRR11550046 |
| SARS-CoV-2_circ_Homo_sapiens_1882 | MN908947.3 | 17916 | 18897 | + | ORF1ab | 2 | circRNA_finder | SRR11550045 |
| SARS-CoV-2_circ_Homo_sapiens_1883 | MN908947.3 | 18017 | 18130 | - | ORF1ab | 2 | find_circ | SRR11550043 |
| SARS-CoV-2_circ_Homo_sapiens_1884 | MN908947.3 | 18017 | 18205 | - | ORF1ab | 2 | find_circ | SRR11550046 |
| SARS-CoV-2_circ_Homo_sapiens_1886 | MN908947.3 | 18017 | 18274 | - | ORF1ab | 2 | find_circ | SRR11550045 |
| SARS-CoV-2_circ_Homo_sapiens_1887 | MN908947.3 | 18017 | 18274 | + | ORF1ab | 2 | circRNA_finder | SRR11550045 |
| SARS-CoV-2_circ_Homo_sapiens_1891 | MN908947.3 | 18017 | 18394 | - | ORF1ab | 2 | find_circ | SRR11550043 |
| SARS-CoV-2_circ_Homo_sapiens_1895 | MN908947.3 | 18017 | 18492 | - | ORF1ab | 2 | find_circ | SRR11550045 |
| SARS-CoV-2_circ_Homo_sapiens_1896 | MN908947.3 | 18017 | 18511 | - | ORF1ab | 2 | find_circ | SRR11550046 |
| SARS-CoV-2_circ_Homo_sapiens_1902 | MN908947.3 | 18017 | 18876 | + | ORF1ab | 2 | circRNA_finder | SRR11550046 |
| SARS-CoV-2_circ_Homo_sapiens_1902 | MN908947.3 | 18017 | 18876 | + | ORF1ab | 2 | circRNA_finder | SRR11550044 |
| SARS-CoV-2_circ_Homo_sapiens_1904 | MN908947.3 | 18017 | 19088 | - | ORF1ab | 2 | find_circ | SRR11550045 |
| SARS-CoV-2_circ_Homo_sapiens_1905 | MN908947.3 | 18017 | 19088 | + | ORF1ab | 2 | circRNA_finder | SRR11550045 |
| SARS-CoV-2_circ_Homo_sapiens_1906 | MN908947.3 | 18017 | 19549 | - | ORF1ab | 2 | find_circ | SRR11550045 |
| SARS-CoV-2_circ_Homo_sapiens_1906 | MN908947.3 | 18017 | 19549 | - | ORF1ab | 2 | find_circ | SRR11550044 |
| SARS-CoV-2_circ_Homo_sapiens_191 | MN908947.3 | 800 | 1414 | - | ORF1ab | 2 | find_circ | SRR11550045 |
| SARS-CoV-2_circ_Homo_sapiens_1917 | MN908947.3 | 18021 | 18309 | + | ORF1ab | 2 | CIRI2 | SRR11550045 |
| SARS-CoV-2_circ_Homo_sapiens_1921 | MN908947.3 | 18021 | 18497 | + | ORF1ab | 2 | find_circ | SRR11550045 |
| SARS-CoV-2_circ_Homo_sapiens_1923 | MN908947.3 | 18021 | 18815 | + | ORF1ab | 2 | find_circ | SRR11550046 |
| SARS-CoV-2_circ_Homo_sapiens_1924 | MN908947.3 | 18021 | 18897 | + | ORF1ab | 2 | find_circ | SRR11550043 |
| SARS-CoV-2_circ_Homo_sapiens_1924 | MN908947.3 | 18021 | 18897 | + | ORF1ab | 2 | circRNA_finder | SRR11550043 |
| SARS-CoV-2_circ_Homo_sapiens_1925 | MN908947.3 | 18021 | 19036 | + | ORF1ab | 2 | find_circ | SRR11550045 |
| SARS-CoV-2_circ_Homo_sapiens_1925 | MN908947.3 | 18021 | 19036 | + | ORF1ab | 2 | circRNA_finder | SRR11550045 |
| SARS-CoV-2_circ_Homo_sapiens_1926 | MN908947.3 | 18021 | 19055 | + | ORF1ab | 2 | find_circ | SRR11550045 |
| SARS-CoV-2_circ_Homo_sapiens_1929 | MN908947.3 | 18021 | 19966 | + | ORF1ab | 2 | find_circ | SRR11550043 |
| SARS-CoV-2_circ_Homo_sapiens_1929 | MN908947.3 | 18021 | 19966 | + | ORF1ab | 2 | circRNA_finder | SRR11550043 |
| SARS-CoV-2_circ_Homo_sapiens_193 | MN908947.3 | 833 | 4585 | - | ORF1ab | 2 | find_circ | SRR11550044 |
| SARS-CoV-2_circ_Homo_sapiens_1932 | MN908947.3 | 18038 | 18355 | + | ORF1ab | 2 | circRNA_finder | SRR11550043 |
| SARS-CoV-2_circ_Homo_sapiens_1935 | MN908947.3 | 18068 | 18582 | + | ORF1ab | 2 | find_circ | SRR11550043 |
| SARS-CoV-2_circ_Homo_sapiens_1938 | MN908947.3 | 18149 | 18906 | + | ORF1ab | 2 | circRNA_finder | SRR11550045 |
| SARS-CoV-2_circ_Homo_sapiens_1938 | MN908947.3 | 18149 | 18906 | + | ORF1ab | 2 | find_circ | SRR11550045 |
| SARS-CoV-2_circ_Homo_sapiens_194 | MN908947.3 | 863 | 1244 | + | ORF1ab | 2 | find_circ | SRR11550045 |
| SARS-CoV-2_circ_Homo_sapiens_1943 | MN908947.3 | 18253 | 18813 | - | ORF1ab | 2 | find_circ | SRR11550046 |
| SARS-CoV-2_circ_Homo_sapiens_1946 | MN908947.3 | 18275 | 18661 | + | ORF1ab | 2 | find_circ | SRR11550043 |
| SARS-CoV-2_circ_Homo_sapiens_1949 | MN908947.3 | 18282 | 18744 | + | ORF1ab | 2 | find_circ | SRR11550045 |
| SARS-CoV-2_circ_Homo_sapiens_1950 | MN908947.3 | 18282 | 18820 | + | ORF1ab | 2 | find_circ | SRR11550045 |
| SARS-CoV-2_circ_Homo_sapiens_196 | MN908947.3 | 873 | 2423 | + | ORF1ab | 2 | find_circ | SRR11550045 |
| SARS-CoV-2_circ_Homo_sapiens_1965 | MN908947.3 | 18356 | 26954 | - | M;E;ORF3a;S;ORF1ab | 2 | find_circ | SRR11550045 |
| SARS-CoV-2_circ_Homo_sapiens_1966 | MN908947.3 | 18368 | 18874 | + | ORF1ab | 2 | find_circ | SRR11550045 |
| SARS-CoV-2_circ_Homo_sapiens_1975 | MN908947.3 | 18407 | 18897 | + | ORF1ab | 2 | find_circ | SRR11550043 |
| SARS-CoV-2_circ_Homo_sapiens_1975 | MN908947.3 | 18407 | 18897 | + | ORF1ab | 2 | circRNA_finder | SRR11550043 |
| SARS-CoV-2_circ_Homo_sapiens_1976 | MN908947.3 | 18407 | 21607 | + | S;ORF1ab | 2 | find_circ | SRR11550043 |
| SARS-CoV-2_circ_Homo_sapiens_1977 | MN908947.3 | 18422 | 18876 | - | ORF1ab | 2 | CIRI2 | SRR11550045 |
| SARS-CoV-2_circ_Homo_sapiens_1981 | MN908947.3 | 18458 | 24620 | - | S;ORF1ab | 2 | find_circ | SRR11550046 |
| SARS-CoV-2_circ_Homo_sapiens_199 | MN908947.3 | 875 | 2134 | - | ORF1ab | 2 | find_circ | SRR11550044 |
| SARS-CoV-2_circ_Homo_sapiens_1995 | MN908947.3 | 18657 | 18876 | - | ORF1ab | 2 | find_circ | SRR11550044 |
| SARS-CoV-2_circ_Homo_sapiens_1996 | MN908947.3 | 18657 | 18876 | + | ORF1ab | 2 | circRNA_finder | SRR11550045 |
| SARS-CoV-2_circ_Homo_sapiens_1996 | MN908947.3 | 18657 | 18876 | + | ORF1ab | 2 | circRNA_finder | SRR11550044 |
| SARS-CoV-2_circ_Homo_sapiens_2 | MN908947.3 | 8 | 314 | + | ORF1ab | 2 | CIRI2 | SRR11550044 |
| SARS-CoV-2_circ_Homo_sapiens_20 | MN908947.3 | 8 | 8487 | + | ORF1ab | 2 | find_circ | SRR11550044 |
| SARS-CoV-2_circ_Homo_sapiens_2003 | MN908947.3 | 18657 | 20103 | - | ORF1ab | 2 | find_circ | SRR11550045 |
| SARS-CoV-2_circ_Homo_sapiens_2004 | MN908947.3 | 18657 | 20103 | + | ORF1ab | 2 | circRNA_finder | SRR11550045 |
| SARS-CoV-2_circ_Homo_sapiens_2005 | MN908947.3 | 18657 | 20697 | - | ORF1ab | 2 | find_circ | SRR11550045 |
| SARS-CoV-2_circ_Homo_sapiens_2006 | MN908947.3 | 18657 | 20697 | + | ORF1ab | 2 | circRNA_finder | SRR11550045 |
| SARS-CoV-2_circ_Homo_sapiens_2007 | MN908947.3 | 18657 | 21109 | - | ORF1ab | 2 | find_circ | SRR11550045 |
| SARS-CoV-2_circ_Homo_sapiens_2008 | MN908947.3 | 18657 | 21109 | + | ORF1ab | 2 | circRNA_finder | SRR11550045 |
| SARS-CoV-2_circ_Homo_sapiens_2011 | MN908947.3 | 18657 | 22739 | - | S;ORF1ab | 2 | find_circ | SRR11550045 |
| SARS-CoV-2_circ_Homo_sapiens_2019 | MN908947.3 | 18881 | 20809 | + | ORF1ab | 2 | find_circ | SRR11550044 |
| SARS-CoV-2_circ_Homo_sapiens_2019 | MN908947.3 | 18881 | 20809 | + | ORF1ab | 2 | circRNA_finder | SRR11550044 |
| SARS-CoV-2_circ_Homo_sapiens_202 | MN908947.3 | 885 | 1403 | - | ORF1ab | 2 | find_circ | SRR11550046 |
| SARS-CoV-2_circ_Homo_sapiens_2020 | MN908947.3 | 18904 | 19085 | + | ORF1ab | 2 | find_circ | SRR11550045 |
| SARS-CoV-2_circ_Homo_sapiens_2021 | MN908947.3 | 18904 | 19644 | + | ORF1ab | 2 | circRNA_finder | SRR11550043 |
| SARS-CoV-2_circ_Homo_sapiens_2021 | MN908947.3 | 18904 | 19644 | + | ORF1ab | 2 | find_circ | SRR11550043 |
| SARS-CoV-2_circ_Homo_sapiens_2025 | MN908947.3 | 18918 | 21887 | - | S;ORF1ab | 2 | find_circ | SRR11550046 |
| SARS-CoV-2_circ_Homo_sapiens_2029 | MN908947.3 | 18982 | 19549 | - | ORF1ab | 2 | find_circ | SRR11550044 |
| SARS-CoV-2_circ_Homo_sapiens_203 | MN908947.3 | 885 | 1403 | + | ORF1ab | 2 | circRNA_finder | SRR11550046 |
| SARS-CoV-2_circ_Homo_sapiens_2030 | MN908947.3 | 18982 | 19549 | + | ORF1ab | 2 | circRNA_finder | SRR11550044 |
| SARS-CoV-2_circ_Homo_sapiens_2032 | MN908947.3 | 18982 | 23102 | - | S;ORF1ab | 2 | find_circ | SRR11550046 |
| SARS-CoV-2_circ_Homo_sapiens_2036 | MN908947.3 | 19010 | 20068 | + | ORF1ab | 2 | find_circ | SRR11550043 |
| SARS-CoV-2_circ_Homo_sapiens_2038 | MN908947.3 | 19033 | 27051 | - | M;E;ORF3a;S;ORF1ab | 2 | find_circ | SRR11550044 |
| SARS-CoV-2_circ_Homo_sapiens_2039 | MN908947.3 | 19033 | 27051 | + | M;E;ORF3a;S;ORF1ab | 2 | circRNA_finder | SRR11550044 |
| SARS-CoV-2_circ_Homo_sapiens_2040 | MN908947.3 | 19033 | 29787 | - | ORF6;ORF10;M;E;ORF3a;S;N;ORF8;ORF7a;ORF7b;ORF1ab | 2 | find_circ | SRR11550046 |
| SARS-CoV-2_circ_Homo_sapiens_2042 | MN908947.3 | 19079 | 19299 | + | ORF1ab | 2 | find_circ | SRR11550046 |
| SARS-CoV-2_circ_Homo_sapiens_2042 | MN908947.3 | 19079 | 19299 | + | ORF1ab | 2 | circRNA_finder | SRR11550046 |
| SARS-CoV-2_circ_Homo_sapiens_2044 | MN908947.3 | 19157 | 20198 | - | ORF1ab | 2 | find_circ | SRR11550045 |
| SARS-CoV-2_circ_Homo_sapiens_2048 | MN908947.3 | 19587 | 19954 | - | ORF1ab | 2 | find_circ | SRR11550044 |
| SARS-CoV-2_circ_Homo_sapiens_2049 | MN908947.3 | 19587 | 19954 | + | ORF1ab | 2 | circRNA_finder | SRR11550044 |
| SARS-CoV-2_circ_Homo_sapiens_2051 | MN908947.3 | 19603 | 20198 | - | ORF1ab | 2 | find_circ | SRR11550044 |
| SARS-CoV-2_circ_Homo_sapiens_2051 | MN908947.3 | 19603 | 20198 | - | ORF1ab | 2 | find_circ | SRR11550045 |
| SARS-CoV-2_circ_Homo_sapiens_2053 | MN908947.3 | 19605 | 19982 | - | ORF1ab | 2 | find_circ | SRR11550044 |
| SARS-CoV-2_circ_Homo_sapiens_2054 | MN908947.3 | 19605 | 19982 | + | ORF1ab | 2 | circRNA_finder | SRR11550043 |
| SARS-CoV-2_circ_Homo_sapiens_2057 | MN908947.3 | 19605 | 20103 | + | ORF1ab | 2 | circRNA_finder | SRR11550045 |
| SARS-CoV-2_circ_Homo_sapiens_206 | MN908947.3 | 907 | 1283 | - | ORF1ab | 2 | find_circ | SRR11550045 |
| SARS-CoV-2_circ_Homo_sapiens_2062 | MN908947.3 | 19605 | 21647 | - | S;ORF1ab | 2 | find_circ | SRR11550045 |
| SARS-CoV-2_circ_Homo_sapiens_2064 | MN908947.3 | 19611 | 20103 | - | ORF1ab | 2 | find_circ | SRR11550046 |
| SARS-CoV-2_circ_Homo_sapiens_2066 | MN908947.3 | 19611 | 20203 | + | ORF1ab | 2 | circRNA_finder | SRR11550043 |
| SARS-CoV-2_circ_Homo_sapiens_2066 | MN908947.3 | 19611 | 20203 | + | ORF1ab | 2 | circRNA_finder | SRR11550045 |
| SARS-CoV-2_circ_Homo_sapiens_2067 | MN908947.3 | 19614 | 20005 | + | ORF1ab | 2 | circRNA_finder | SRR11550045 |
| SARS-CoV-2_circ_Homo_sapiens_2067 | MN908947.3 | 19614 | 20005 | + | ORF1ab | 2 | find_circ | SRR11550045 |
| SARS-CoV-2_circ_Homo_sapiens_2068 | MN908947.3 | 19623 | 20061 | + | ORF1ab | 2 | find_circ | SRR11550044 |
| SARS-CoV-2_circ_Homo_sapiens_207 | MN908947.3 | 907 | 1283 | + | ORF1ab | 2 | circRNA_finder | SRR11550045 |
| SARS-CoV-2_circ_Homo_sapiens_2079 | MN908947.3 | 20222 | 22445 | - | S;ORF1ab | 2 | find_circ | SRR11550045 |
| SARS-CoV-2_circ_Homo_sapiens_2083 | MN908947.3 | 20336 | 20907 | + | ORF1ab | 2 | find_circ | SRR11550044 |
| SARS-CoV-2_circ_Homo_sapiens_2083 | MN908947.3 | 20336 | 20907 | + | ORF1ab | 2 | circRNA_finder | SRR11550044 |
| SARS-CoV-2_circ_Homo_sapiens_2084 | MN908947.3 | 20336 | 21700 | + | S;ORF1ab | 2 | find_circ | SRR11550045 |
| SARS-CoV-2_circ_Homo_sapiens_2084 | MN908947.3 | 20336 | 21700 | + | S;ORF1ab | 2 | circRNA_finder | SRR11550045 |
| SARS-CoV-2_circ_Homo_sapiens_2084 | MN908947.3 | 20336 | 21700 | + | S;ORF1ab | 2 | find_circ | SRR11550043 |
| SARS-CoV-2_circ_Homo_sapiens_2087 | MN908947.3 | 20357 | 21986 | + | S;ORF1ab | 2 | find_circ | SRR11550044 |
| SARS-CoV-2_circ_Homo_sapiens_2088 | MN908947.3 | 20366 | 20697 | - | ORF1ab | 2 | find_circ | SRR11550046 |
| SARS-CoV-2_circ_Homo_sapiens_2093 | MN908947.3 | 20384 | 23567 | + | S;ORF1ab | 2 | find_circ | SRR11550045 |
| SARS-CoV-2_circ_Homo_sapiens_2093 | MN908947.3 | 20384 | 23567 | + | S;ORF1ab | 2 | circRNA_finder | SRR11550045 |
| SARS-CoV-2_circ_Homo_sapiens_2099 | MN908947.3 | 20405 | 21181 | - | ORF1ab | 2 | CIRI2 | SRR11550043 |
| SARS-CoV-2_circ_Homo_sapiens_2101 | MN908947.3 | 20405 | 22739 | - | S;ORF1ab | 2 | find_circ | SRR11550046 |
| SARS-CoV-2_circ_Homo_sapiens_2102 | MN908947.3 | 20405 | 22739 | + | S;ORF1ab | 2 | circRNA_finder | SRR11550046 |
| SARS-CoV-2_circ_Homo_sapiens_2105 | MN908947.3 | 20420 | 21685 | + | S;ORF1ab | 2 | find_circ | SRR11550045 |
| SARS-CoV-2_circ_Homo_sapiens_211 | MN908947.3 | 907 | 1669 | - | ORF1ab | 2 | find_circ | SRR11550043 |
| SARS-CoV-2_circ_Homo_sapiens_2113 | MN908947.3 | 20763 | 21181 | - | ORF1ab | 2 | find_circ | SRR11550045 |
| SARS-CoV-2_circ_Homo_sapiens_2114 | MN908947.3 | 20763 | 21181 | + | ORF1ab | 2 | circRNA_finder | SRR11550045 |
| SARS-CoV-2_circ_Homo_sapiens_2117 | MN908947.3 | 20763 | 21720 | - | S;ORF1ab | 2 | find_circ | SRR11550045 |
| SARS-CoV-2_circ_Homo_sapiens_2118 | MN908947.3 | 20763 | 21720 | + | S;ORF1ab | 2 | circRNA_finder | SRR11550045 |
| SARS-CoV-2_circ_Homo_sapiens_212 | MN908947.3 | 907 | 1669 | + | ORF1ab | 2 | circRNA_finder | SRR11550043 |
| SARS-CoV-2_circ_Homo_sapiens_2121 | MN908947.3 | 20768 | 21726 | + | S;ORF1ab | 2 | circRNA_finder | SRR11550043 |
| SARS-CoV-2_circ_Homo_sapiens_2122 | MN908947.3 | 20864 | 21653 | - | S;ORF1ab | 2 | find_circ | SRR11550043 |
| SARS-CoV-2_circ_Homo_sapiens_2123 | MN908947.3 | 20864 | 21653 | + | S;ORF1ab | 2 | circRNA_finder | SRR11550043 |
| SARS-CoV-2_circ_Homo_sapiens_2125 | MN908947.3 | 20888 | 23066 | + | S;ORF1ab | 2 | find_circ | SRR11550046 |
| SARS-CoV-2_circ_Homo_sapiens_2126 | MN908947.3 | 20891 | 23660 | + | S;ORF1ab | 2 | CIRI2 | SRR11550043 |
| SARS-CoV-2_circ_Homo_sapiens_2129 | MN908947.3 | 20965 | 21647 | - | S;ORF1ab | 2 | find_circ | SRR11550046 |
| SARS-CoV-2_circ_Homo_sapiens_2130 | MN908947.3 | 20965 | 21720 | - | S;ORF1ab | 2 | find_circ | SRR11550045 |
| SARS-CoV-2_circ_Homo_sapiens_2133 | MN908947.3 | 21058 | 21720 | - | S;ORF1ab | 2 | find_circ | SRR11550046 |
| SARS-CoV-2_circ_Homo_sapiens_2137 | MN908947.3 | 21111 | 21540 | - | ORF1ab | 2 | find_circ | SRR11550046 |
| SARS-CoV-2_circ_Homo_sapiens_2138 | MN908947.3 | 21111 | 21540 | + | ORF1ab | 2 | circRNA_finder | SRR11550046 |
| SARS-CoV-2_circ_Homo_sapiens_2139 | MN908947.3 | 21111 | 21638 | - | S;ORF1ab | 2 | find_circ | SRR11550044 |
| SARS-CoV-2_circ_Homo_sapiens_2143 | MN908947.3 | 21174 | 21574 | - | S;ORF1ab | 2 | find_circ | SRR11550045 |
| SARS-CoV-2_circ_Homo_sapiens_2153 | MN908947.3 | 21636 | 23409 | - | S | 2 | find_circ | SRR11550045 |
| SARS-CoV-2_circ_Homo_sapiens_2154 | MN908947.3 | 21636 | 23409 | + | S | 2 | circRNA_finder | SRR11550045 |
| SARS-CoV-2_circ_Homo_sapiens_2155 | MN908947.3 | 21647 | 25389 | - | S | 2 | find_circ | SRR11550045 |
| SARS-CoV-2_circ_Homo_sapiens_2159 | MN908947.3 | 21687 | 22483 | + | S | 2 | find_circ | SRR11550044 |
| SARS-CoV-2_circ_Homo_sapiens_216 | MN908947.3 | 926 | 3737 | - | ORF1ab | 2 | find_circ | SRR11550046 |
| SARS-CoV-2_circ_Homo_sapiens_2162 | MN908947.3 | 21687 | 26283 | + | ORF3a;S;E | 2 | find_circ | SRR11550044 |
| SARS-CoV-2_circ_Homo_sapiens_2162 | MN908947.3 | 21687 | 26283 | + | ORF3a;S;E | 2 | circRNA_finder | SRR11550044 |
| SARS-CoV-2_circ_Homo_sapiens_2165 | MN908947.3 | 21708 | 29534 | - | ORF6;M;E;ORF3a;S;N;ORF8;ORF7a;ORF7b | 2 | find_circ | SRR11550046 |
| SARS-CoV-2_circ_Homo_sapiens_2166 | MN908947.3 | 21708 | 29534 | + | ORF6;M;E;ORF3a;S;N;ORF8;ORF7a;ORF7b | 2 | circRNA_finder | SRR11550046 |
| SARS-CoV-2_circ_Homo_sapiens_2170 | MN908947.3 | 21722 | 23446 | - | S | 2 | find_circ | SRR11550044 |
| SARS-CoV-2_circ_Homo_sapiens_2171 | MN908947.3 | 21732 | 22126 | - | S | 2 | find_circ | SRR11550046 |
| SARS-CoV-2_circ_Homo_sapiens_2172 | MN908947.3 | 21732 | 22126 | + | S | 2 | circRNA_finder | SRR11550046 |
| SARS-CoV-2_circ_Homo_sapiens_2174 | MN908947.3 | 21732 | 22387 | - | S | 2 | find_circ | SRR11550046 |
| SARS-CoV-2_circ_Homo_sapiens_2175 | MN908947.3 | 21732 | 22387 | + | S | 2 | circRNA_finder | SRR11550046 |
| SARS-CoV-2_circ_Homo_sapiens_2177 | MN908947.3 | 21732 | 22438 | - | S | 2 | CIRI2 | SRR11550046 |
| SARS-CoV-2_circ_Homo_sapiens_218 | MN908947.3 | 926 | 5821 | - | ORF1ab | 2 | find_circ | SRR11550044 |
| SARS-CoV-2_circ_Homo_sapiens_2180 | MN908947.3 | 21732 | 22447 | - | S | 2 | find_circ | SRR11550045 |
| SARS-CoV-2_circ_Homo_sapiens_2183 | MN908947.3 | 21768 | 22387 | - | S | 2 | find_circ | SRR11550045 |
| SARS-CoV-2_circ_Homo_sapiens_2184 | MN908947.3 | 21768 | 22424 | - | S | 2 | find_circ | SRR11550045 |
| SARS-CoV-2_circ_Homo_sapiens_2185 | MN908947.3 | 21768 | 22424 | + | S | 2 | circRNA_finder | SRR11550045 |
| SARS-CoV-2_circ_Homo_sapiens_2186 | MN908947.3 | 21768 | 22447 | - | S | 2 | find_circ | SRR11550046 |
| SARS-CoV-2_circ_Homo_sapiens_2189 | MN908947.3 | 21768 | 23028 | - | S | 2 | find_circ | SRR11550044 |
| SARS-CoV-2_circ_Homo_sapiens_219 | MN908947.3 | 926 | 5821 | + | ORF1ab | 2 | circRNA_finder | SRR11550044 |
| SARS-CoV-2_circ_Homo_sapiens_2195 | MN908947.3 | 21889 | 22289 | - | S | 2 | find_circ | SRR11550043 |
| SARS-CoV-2_circ_Homo_sapiens_2196 | MN908947.3 | 21889 | 22325 | - | S | 2 | find_circ | SRR11550043 |
| SARS-CoV-2_circ_Homo_sapiens_2198 | MN908947.3 | 21889 | 22382 | - | S | 2 | find_circ | SRR11550046 |
| SARS-CoV-2_circ_Homo_sapiens_2199 | MN908947.3 | 21889 | 22382 | + | S | 2 | circRNA_finder | SRR11550046 |
| SARS-CoV-2_circ_Homo_sapiens_22 | MN908947.3 | 8 | 20921 | + | ORF1ab | 2 | find_circ | SRR11550046 |
| SARS-CoV-2_circ_Homo_sapiens_2200 | MN908947.3 | 21889 | 22438 | - | S | 2 | find_circ | SRR11550045 |
| SARS-CoV-2_circ_Homo_sapiens_2202 | MN908947.3 | 21889 | 23333 | - | S | 2 | find_circ | SRR11550043 |
| SARS-CoV-2_circ_Homo_sapiens_2204 | MN908947.3 | 21889 | 25702 | - | ORF3a;S | 2 | find_circ | SRR11550046 |
| SARS-CoV-2_circ_Homo_sapiens_2205 | MN908947.3 | 21889 | 25702 | + | ORF3a;S | 2 | circRNA_finder | SRR11550046 |
| SARS-CoV-2_circ_Homo_sapiens_2207 | MN908947.3 | 21894 | 23066 | + | S | 2 | find_circ | SRR11550046 |
| SARS-CoV-2_circ_Homo_sapiens_2209 | MN908947.3 | 21894 | 23723 | + | S | 2 | find_circ | SRR11550045 |
| SARS-CoV-2_circ_Homo_sapiens_221 | MN908947.3 | 926 | 29375 | - | ORF6;M;E;ORF3a;S;N;ORF8;ORF7a;ORF7b;ORF1ab | 2 | find_circ | SRR11550046 |
| SARS-CoV-2_circ_Homo_sapiens_2210 | MN908947.3 | 21894 | 25533 | + | ORF3a;S | 2 | find_circ | SRR11550045 |
| SARS-CoV-2_circ_Homo_sapiens_2219 | MN908947.3 | 21998 | 22865 | - | S | 2 | find_circ | SRR11550044 |
| SARS-CoV-2_circ_Homo_sapiens_2219 | MN908947.3 | 21998 | 22865 | - | S | 2 | find_circ | SRR11550043 |
| SARS-CoV-2_circ_Homo_sapiens_2221 | MN908947.3 | 21998 | 24462 | - | S | 2 | find_circ | SRR11550046 |
| SARS-CoV-2_circ_Homo_sapiens_2224 | MN908947.3 | 22001 | 22447 | - | S | 2 | find_circ | SRR11550043 |
| SARS-CoV-2_circ_Homo_sapiens_2226 | MN908947.3 | 22001 | 22915 | - | S | 2 | find_circ | SRR11550046 |
| SARS-CoV-2_circ_Homo_sapiens_2227 | MN908947.3 | 22001 | 24457 | - | S | 2 | find_circ | SRR11550044 |
| SARS-CoV-2_circ_Homo_sapiens_223 | MN908947.3 | 935 | 29268 | - | ORF6;M;E;ORF3a;S;N;ORF8;ORF7a;ORF7b;ORF1ab | 2 | find_circ | SRR11550045 |
| SARS-CoV-2_circ_Homo_sapiens_2233 | MN908947.3 | 22287 | 22854 | - | S | 2 | find_circ | SRR11550043 |
| SARS-CoV-2_circ_Homo_sapiens_2234 | MN908947.3 | 22287 | 26567 | - | ORF3a;S;M;E | 2 | find_circ | SRR11550045 |
| SARS-CoV-2_circ_Homo_sapiens_2235 | MN908947.3 | 22296 | 23060 | - | S | 2 | find_circ | SRR11550046 |
| SARS-CoV-2_circ_Homo_sapiens_2236 | MN908947.3 | 22296 | 23060 | + | S | 2 | circRNA_finder | SRR11550046 |
| SARS-CoV-2_circ_Homo_sapiens_224 | MN908947.3 | 935 | 29268 | + | ORF6;M;E;ORF3a;S;N;ORF8;ORF7a;ORF7b;ORF1ab | 2 | circRNA_finder | SRR11550045 |
| SARS-CoV-2_circ_Homo_sapiens_2249 | MN908947.3 | 22414 | 24025 | - | S | 2 | find_circ | SRR11550044 |
| SARS-CoV-2_circ_Homo_sapiens_225 | MN908947.3 | 966 | 1403 | - | ORF1ab | 2 | find_circ | SRR11550046 |
| SARS-CoV-2_circ_Homo_sapiens_2250 | MN908947.3 | 22414 | 24025 | + | S | 2 | circRNA_finder | SRR11550044 |
| SARS-CoV-2_circ_Homo_sapiens_2254 | MN908947.3 | 22431 | 22858 | + | S | 2 | find_circ | SRR11550043 |
| SARS-CoV-2_circ_Homo_sapiens_2255 | MN908947.3 | 22431 | 23561 | + | S | 2 | circRNA_finder | SRR11550045 |
| SARS-CoV-2_circ_Homo_sapiens_2255 | MN908947.3 | 22431 | 23561 | + | S | 2 | find_circ | SRR11550045 |
| SARS-CoV-2_circ_Homo_sapiens_2258 | MN908947.3 | 22483 | 23276 | - | S | 2 | find_circ | SRR11550046 |
| SARS-CoV-2_circ_Homo_sapiens_2259 | MN908947.3 | 22488 | 23561 | + | S | 2 | CIRI2 | SRR11550046 |
| SARS-CoV-2_circ_Homo_sapiens_226 | MN908947.3 | 996 | 1472 | - | ORF1ab | 2 | find_circ | SRR11550046 |
| SARS-CoV-2_circ_Homo_sapiens_2262 | MN908947.3 | 22584 | 23015 | + | S | 2 | find_circ | SRR11550045 |
| SARS-CoV-2_circ_Homo_sapiens_2262 | MN908947.3 | 22584 | 23015 | + | S | 2 | circRNA_finder | SRR11550045 |
| SARS-CoV-2_circ_Homo_sapiens_2263 | MN908947.3 | 22592 | 22865 | - | S | 2 | find_circ | SRR11550043 |
| SARS-CoV-2_circ_Homo_sapiens_2269 | MN908947.3 | 22852 | 23691 | - | S | 2 | find_circ | SRR11550044 |
| SARS-CoV-2_circ_Homo_sapiens_227 | MN908947.3 | 1009 | 1230 | + | ORF1ab | 2 | find_circ | SRR11550045 |
| SARS-CoV-2_circ_Homo_sapiens_2270 | MN908947.3 | 22866 | 23339 | + | S | 2 | find_circ | SRR11550045 |
| SARS-CoV-2_circ_Homo_sapiens_2270 | MN908947.3 | 22866 | 23339 | + | S | 2 | circRNA_finder | SRR11550045 |
| SARS-CoV-2_circ_Homo_sapiens_2271 | MN908947.3 | 22866 | 23425 | + | S | 2 | find_circ | SRR11550045 |
| SARS-CoV-2_circ_Homo_sapiens_2271 | MN908947.3 | 22866 | 23425 | + | S | 2 | circRNA_finder | SRR11550045 |
| SARS-CoV-2_circ_Homo_sapiens_2272 | MN908947.3 | 22866 | 25653 | + | ORF3a;S | 2 | find_circ | SRR11550043 |
| SARS-CoV-2_circ_Homo_sapiens_2275 | MN908947.3 | 22950 | 23305 | - | S | 2 | CIRI2 | SRR11550043 |
| SARS-CoV-2_circ_Homo_sapiens_2275 | MN908947.3 | 22950 | 23305 | - | S | 2 | find_circ | SRR11550045 |
| SARS-CoV-2_circ_Homo_sapiens_2275 | MN908947.3 | 22950 | 23305 | - | S | 2 | find_circ | SRR11550043 |
| SARS-CoV-2_circ_Homo_sapiens_2279 | MN908947.3 | 22950 | 23446 | - | S | 2 | find_circ | SRR11550045 |
| SARS-CoV-2_circ_Homo_sapiens_228 | MN908947.3 | 1009 | 1232 | + | ORF1ab | 2 | find_circ | SRR11550045 |
| SARS-CoV-2_circ_Homo_sapiens_228 | MN908947.3 | 1009 | 1232 | + | ORF1ab | 2 | find_circ | SRR11550043 |
| SARS-CoV-2_circ_Homo_sapiens_2280 | MN908947.3 | 22950 | 23446 | + | S | 2 | circRNA_finder | SRR11550045 |
| SARS-CoV-2_circ_Homo_sapiens_2281 | MN908947.3 | 22950 | 23456 | - | S | 2 | find_circ | SRR11550043 |
| SARS-CoV-2_circ_Homo_sapiens_2282 | MN908947.3 | 22950 | 23456 | + | S | 2 | circRNA_finder | SRR11550043 |
| SARS-CoV-2_circ_Homo_sapiens_2283 | MN908947.3 | 22950 | 23519 | + | S | 2 | circRNA_finder | SRR11550046 |
| SARS-CoV-2_circ_Homo_sapiens_2285 | MN908947.3 | 22950 | 23576 | + | S | 2 | circRNA_finder | SRR11550045 |
| SARS-CoV-2_circ_Homo_sapiens_2286 | MN908947.3 | 22950 | 23693 | - | S | 2 | find_circ | SRR11550045 |
| SARS-CoV-2_circ_Homo_sapiens_2287 | MN908947.3 | 22950 | 23693 | + | S | 2 | circRNA_finder | SRR11550045 |
| SARS-CoV-2_circ_Homo_sapiens_2291 | MN908947.3 | 22950 | 25516 | - | ORF3a;S | 2 | find_circ | SRR11550043 |
| SARS-CoV-2_circ_Homo_sapiens_2291 | MN908947.3 | 22950 | 25516 | - | ORF3a;S | 2 | find_circ | SRR11550044 |
| SARS-CoV-2_circ_Homo_sapiens_2292 | MN908947.3 | 22950 | 25516 | + | ORF3a;S | 2 | circRNA_finder | SRR11550044 |
| SARS-CoV-2_circ_Homo_sapiens_2293 | MN908947.3 | 22950 | 26241 | - | ORF3a;S | 2 | find_circ | SRR11550044 |
| SARS-CoV-2_circ_Homo_sapiens_2299 | MN908947.3 | 22962 | 23567 | + | S | 2 | find_circ | SRR11550046 |
| SARS-CoV-2_circ_Homo_sapiens_2299 | MN908947.3 | 22962 | 23567 | + | S | 2 | circRNA_finder | SRR11550045 |
| SARS-CoV-2_circ_Homo_sapiens_230 | MN908947.3 | 1009 | 1357 | + | ORF1ab | 2 | find_circ | SRR11550045 |
| SARS-CoV-2_circ_Homo_sapiens_2314 | MN908947.3 | 23062 | 24350 | - | S | 2 | find_circ | SRR11550043 |
| SARS-CoV-2_circ_Homo_sapiens_2316 | MN908947.3 | 23097 | 23660 | + | S | 2 | CIRI2 | SRR11550045 |
| SARS-CoV-2_circ_Homo_sapiens_2317 | MN908947.3 | 23100 | 23678 | - | S | 2 | find_circ | SRR11550045 |
| SARS-CoV-2_circ_Homo_sapiens_2322 | MN908947.3 | 23142 | 23519 | - | S | 2 | find_circ | SRR11550045 |
| SARS-CoV-2_circ_Homo_sapiens_2323 | MN908947.3 | 23142 | 23519 | + | S | 2 | circRNA_finder | SRR11550045 |
| SARS-CoV-2_circ_Homo_sapiens_2324 | MN908947.3 | 23142 | 23594 | - | S | 2 | CIRI2 | SRR11550044 |
| SARS-CoV-2_circ_Homo_sapiens_2326 | MN908947.3 | 23142 | 23624 | - | S | 2 | CIRI2 | SRR11550045 |
| SARS-CoV-2_circ_Homo_sapiens_233 | MN908947.3 | 1009 | 1574 | + | ORF1ab | 2 | find_circ | SRR11550046 |
| SARS-CoV-2_circ_Homo_sapiens_2331 | MN908947.3 | 23142 | 25548 | - | ORF3a;S | 2 | CIRI2 | SRR11550046 |
| SARS-CoV-2_circ_Homo_sapiens_2335 | MN908947.3 | 23142 | 25625 | - | ORF3a;S | 2 | find_circ | SRR11550046 |
| SARS-CoV-2_circ_Homo_sapiens_2336 | MN908947.3 | 23142 | 25625 | + | ORF3a;S | 2 | circRNA_finder | SRR11550046 |
| SARS-CoV-2_circ_Homo_sapiens_2338 | MN908947.3 | 23171 | 23333 | - | S | 2 | find_circ | SRR11550045 |
| SARS-CoV-2_circ_Homo_sapiens_2339 | MN908947.3 | 23189 | 26825 | - | ORF3a;S;M;E | 2 | find_circ | SRR11550043 |
| SARS-CoV-2_circ_Homo_sapiens_2340 | MN908947.3 | 23203 | 23600 | - | S | 2 | find_circ | SRR11550043 |
| SARS-CoV-2_circ_Homo_sapiens_2341 | MN908947.3 | 23203 | 26788 | - | ORF3a;S;M;E | 2 | find_circ | SRR11550046 |
| SARS-CoV-2_circ_Homo_sapiens_2345 | MN908947.3 | 23267 | 23519 | - | S | 2 | find_circ | SRR11550045 |
| SARS-CoV-2_circ_Homo_sapiens_235 | MN908947.3 | 1009 | 1579 | + | ORF1ab | 2 | find_circ | SRR11550045 |
| SARS-CoV-2_circ_Homo_sapiens_2355 | MN908947.3 | 23326 | 25664 | - | ORF3a;S | 2 | find_circ | SRR11550043 |
| SARS-CoV-2_circ_Homo_sapiens_2356 | MN908947.3 | 23328 | 25545 | - | ORF3a;S | 2 | find_circ | SRR11550045 |
| SARS-CoV-2_circ_Homo_sapiens_2358 | MN908947.3 | 23381 | 25707 | - | ORF3a;S | 2 | find_circ | SRR11550044 |
| SARS-CoV-2_circ_Homo_sapiens_2359 | MN908947.3 | 23384 | 24192 | + | S | 2 | circRNA_finder | SRR11550045 |
| SARS-CoV-2_circ_Homo_sapiens_2359 | MN908947.3 | 23384 | 24192 | + | S | 2 | find_circ | SRR11550045 |
| SARS-CoV-2_circ_Homo_sapiens_2360 | MN908947.3 | 23411 | 24462 | - | S | 2 | find_circ | SRR11550044 |
| SARS-CoV-2_circ_Homo_sapiens_2362 | MN908947.3 | 23418 | 24276 | + | S | 2 | circRNA_finder | SRR11550044 |
| SARS-CoV-2_circ_Homo_sapiens_2362 | MN908947.3 | 23418 | 24276 | + | S | 2 | find_circ | SRR11550044 |
| SARS-CoV-2_circ_Homo_sapiens_2364 | MN908947.3 | 23478 | 25087 | + | S | 2 | find_circ | SRR11550046 |
| SARS-CoV-2_circ_Homo_sapiens_2366 | MN908947.3 | 23478 | 26827 | + | ORF3a;S;M;E | 2 | CIRI2 | SRR11550045 |
| SARS-CoV-2_circ_Homo_sapiens_2367 | MN908947.3 | 23526 | 25432 | - | ORF3a;S | 2 | find_circ | SRR11550044 |
| SARS-CoV-2_circ_Homo_sapiens_237 | MN908947.3 | 1009 | 2779 | + | ORF1ab | 2 | find_circ | SRR11550043 |
| SARS-CoV-2_circ_Homo_sapiens_237 | MN908947.3 | 1009 | 2779 | + | ORF1ab | 2 | find_circ | SRR11550045 |
| SARS-CoV-2_circ_Homo_sapiens_2372 | MN908947.3 | 23556 | 24377 | - | S | 2 | find_circ | SRR11550045 |
| SARS-CoV-2_circ_Homo_sapiens_2372 | MN908947.3 | 23556 | 24377 | - | S | 2 | find_circ | SRR11550043 |
| SARS-CoV-2_circ_Homo_sapiens_2373 | MN908947.3 | 23556 | 24377 | + | S | 2 | circRNA_finder | SRR11550045 |
| SARS-CoV-2_circ_Homo_sapiens_2373 | MN908947.3 | 23556 | 24377 | + | S | 2 | circRNA_finder | SRR11550043 |
| SARS-CoV-2_circ_Homo_sapiens_2376 | MN908947.3 | 23556 | 25671 | - | ORF3a;S | 2 | find_circ | SRR11550044 |
| SARS-CoV-2_circ_Homo_sapiens_2378 | MN908947.3 | 23568 | 24139 | + | S | 2 | CIRI2 | SRR11550045 |
| SARS-CoV-2_circ_Homo_sapiens_2378 | MN908947.3 | 23568 | 24139 | + | S | 2 | find_circ | SRR11550045 |
| SARS-CoV-2_circ_Homo_sapiens_2378 | MN908947.3 | 23568 | 24139 | + | S | 2 | find_circ | SRR11550046 |
| SARS-CoV-2_circ_Homo_sapiens_2378 | MN908947.3 | 23568 | 24139 | + | S | 2 | circRNA_finder | SRR11550046 |
| SARS-CoV-2_circ_Homo_sapiens_2379 | MN908947.3 | 23568 | 24192 | + | S | 2 | find_circ | SRR11550046 |
| SARS-CoV-2_circ_Homo_sapiens_2380 | MN908947.3 | 23568 | 24412 | + | S | 2 | find_circ | SRR11550044 |
| SARS-CoV-2_circ_Homo_sapiens_2381 | MN908947.3 | 23568 | 24448 | + | S | 2 | find_circ | SRR11550045 |
| SARS-CoV-2_circ_Homo_sapiens_2381 | MN908947.3 | 23568 | 24448 | + | S | 2 | circRNA_finder | SRR11550045 |
| SARS-CoV-2_circ_Homo_sapiens_2383 | MN908947.3 | 23568 | 25203 | + | S | 2 | CIRI2 | SRR11550046 |
| SARS-CoV-2_circ_Homo_sapiens_2384 | MN908947.3 | 23568 | 25243 | + | S | 2 | find_circ | SRR11550045 |
| SARS-CoV-2_circ_Homo_sapiens_2389 | MN908947.3 | 23568 | 25653 | + | ORF3a;S | 2 | CIRI2 | SRR11550046 |
| SARS-CoV-2_circ_Homo_sapiens_239 | MN908947.3 | 1009 | 2866 | + | ORF1ab | 2 | circRNA_finder | SRR11550046 |
| SARS-CoV-2_circ_Homo_sapiens_239 | MN908947.3 | 1009 | 2866 | + | ORF1ab | 2 | find_circ | SRR11550046 |
| SARS-CoV-2_circ_Homo_sapiens_2394 | MN908947.3 | 23568 | 26783 | + | ORF3a;S;M;E | 2 | CIRI2 | SRR11550043 |
| SARS-CoV-2_circ_Homo_sapiens_2397 | MN908947.3 | 23568 | 27187 | + | ORF3a;S;M;E | 2 | circRNA_finder | SRR11550046 |
| SARS-CoV-2_circ_Homo_sapiens_2397 | MN908947.3 | 23568 | 27187 | + | ORF3a;S;M;E | 2 | find_circ | SRR11550046 |
| SARS-CoV-2_circ_Homo_sapiens_2398 | MN908947.3 | 23568 | 27620 | + | ORF6;M;E;ORF3a;S;ORF7a | 2 | find_circ | SRR11550044 |
| SARS-CoV-2_circ_Homo_sapiens_2399 | MN908947.3 | 23568 | 27636 | + | ORF6;M;E;ORF3a;S;ORF7a | 2 | find_circ | SRR11550045 |
| SARS-CoV-2_circ_Homo_sapiens_2399 | MN908947.3 | 23568 | 27636 | + | ORF6;M;E;ORF3a;S;ORF7a | 2 | circRNA_finder | SRR11550045 |
| SARS-CoV-2_circ_Homo_sapiens_24 | MN908947.3 | 8 | 22609 | + | S;ORF1ab | 2 | CIRI2 | SRR11550044 |
| SARS-CoV-2_circ_Homo_sapiens_24 | MN908947.3 | 8 | 22609 | + | S;ORF1ab | 2 | find_circ | SRR11550043 |
| SARS-CoV-2_circ_Homo_sapiens_2400 | MN908947.3 | 23581 | 24192 | + | S | 2 | find_circ | SRR11550046 |
| SARS-CoV-2_circ_Homo_sapiens_2400 | MN908947.3 | 23581 | 24192 | + | S | 2 | find_circ | SRR11550045 |
| SARS-CoV-2_circ_Homo_sapiens_241 | MN908947.3 | 1009 | 3350 | + | ORF1ab | 2 | find_circ | SRR11550045 |
| SARS-CoV-2_circ_Homo_sapiens_2410 | MN908947.3 | 23581 | 26783 | + | ORF3a;S;M;E | 2 | circRNA_finder | SRR11550044 |
| SARS-CoV-2_circ_Homo_sapiens_2410 | MN908947.3 | 23581 | 26783 | + | ORF3a;S;M;E | 2 | find_circ | SRR11550044 |
| SARS-CoV-2_circ_Homo_sapiens_2411 | MN908947.3 | 23588 | 24200 | + | S | 2 | find_circ | SRR11550044 |
| SARS-CoV-2_circ_Homo_sapiens_2414 | MN908947.3 | 23594 | 24763 | + | S | 2 | find_circ | SRR11550043 |
| SARS-CoV-2_circ_Homo_sapiens_2414 | MN908947.3 | 23594 | 24763 | + | S | 2 | circRNA_finder | SRR11550043 |
| SARS-CoV-2_circ_Homo_sapiens_2416 | MN908947.3 | 23596 | 24377 | - | S | 2 | find_circ | SRR11550045 |
| SARS-CoV-2_circ_Homo_sapiens_2420 | MN908947.3 | 23625 | 24192 | + | S | 2 | find_circ | SRR11550046 |
| SARS-CoV-2_circ_Homo_sapiens_2421 | MN908947.3 | 23625 | 24946 | + | S | 2 | circRNA_finder | SRR11550045 |
| SARS-CoV-2_circ_Homo_sapiens_2421 | MN908947.3 | 23625 | 24946 | + | S | 2 | find_circ | SRR11550045 |
| SARS-CoV-2_circ_Homo_sapiens_2425 | MN908947.3 | 23625 | 25444 | + | ORF3a;S | 2 | find_circ | SRR11550046 |
| SARS-CoV-2_circ_Homo_sapiens_2426 | MN908947.3 | 23625 | 26154 | + | ORF3a;S | 2 | find_circ | SRR11550043 |
| SARS-CoV-2_circ_Homo_sapiens_2430 | MN908947.3 | 23629 | 25653 | + | ORF3a;S | 2 | circRNA_finder | SRR11550044 |
| SARS-CoV-2_circ_Homo_sapiens_2432 | MN908947.3 | 23629 | 28428 | + | ORF6;M;E;ORF3a;S;N;ORF8;ORF7a;ORF7b | 2 | CIRI2 | SRR11550046 |
| SARS-CoV-2_circ_Homo_sapiens_2433 | MN908947.3 | 23658 | 24222 | - | S | 2 | find_circ | SRR11550044 |
| SARS-CoV-2_circ_Homo_sapiens_2434 | MN908947.3 | 23658 | 24989 | - | S | 2 | find_circ | SRR11550043 |
| SARS-CoV-2_circ_Homo_sapiens_2435 | MN908947.3 | 23658 | 24989 | + | S | 2 | circRNA_finder | SRR11550043 |
| SARS-CoV-2_circ_Homo_sapiens_2436 | MN908947.3 | 23658 | 27678 | - | ORF6;M;E;ORF3a;S;ORF7a | 2 | find_circ | SRR11550044 |
| SARS-CoV-2_circ_Homo_sapiens_2439 | MN908947.3 | 23684 | 24908 | - | S | 2 | find_circ | SRR11550046 |
| SARS-CoV-2_circ_Homo_sapiens_2443 | MN908947.3 | 23779 | 24137 | - | S | 2 | find_circ | SRR11550043 |
| SARS-CoV-2_circ_Homo_sapiens_2444 | MN908947.3 | 23779 | 24137 | + | S | 2 | circRNA_finder | SRR11550043 |
| SARS-CoV-2_circ_Homo_sapiens_2446 | MN908947.3 | 23855 | 25432 | - | ORF3a;S | 2 | find_circ | SRR11550045 |
| SARS-CoV-2_circ_Homo_sapiens_2447 | MN908947.3 | 23874 | 24276 | + | S | 2 | CIRI2 | SRR11550045 |
| SARS-CoV-2_circ_Homo_sapiens_2449 | MN908947.3 | 24021 | 26778 | + | ORF3a;S;M;E | 2 | find_circ | SRR11550044 |
| SARS-CoV-2_circ_Homo_sapiens_2450 | MN908947.3 | 24027 | 24137 | - | S | 2 | find_circ | SRR11550045 |
| SARS-CoV-2_circ_Homo_sapiens_2452 | MN908947.3 | 24027 | 24457 | - | S | 2 | find_circ | SRR11550044 |
| SARS-CoV-2_circ_Homo_sapiens_2453 | MN908947.3 | 24027 | 24457 | + | S | 2 | circRNA_finder | SRR11550044 |
| SARS-CoV-2_circ_Homo_sapiens_2454 | MN908947.3 | 24027 | 24462 | - | S | 2 | find_circ | SRR11550045 |
| SARS-CoV-2_circ_Homo_sapiens_2455 | MN908947.3 | 24027 | 24462 | + | S | 2 | circRNA_finder | SRR11550045 |
| SARS-CoV-2_circ_Homo_sapiens_2457 | MN908947.3 | 24027 | 24620 | + | S | 2 | circRNA_finder | SRR11550045 |
| SARS-CoV-2_circ_Homo_sapiens_2461 | MN908947.3 | 24027 | 24989 | - | S | 2 | find_circ | SRR11550046 |
| SARS-CoV-2_circ_Homo_sapiens_2465 | MN908947.3 | 24027 | 25516 | - | ORF3a;S | 2 | find_circ | SRR11550045 |
| SARS-CoV-2_circ_Homo_sapiens_2468 | MN908947.3 | 24027 | 25552 | - | ORF3a;S | 2 | find_circ | SRR11550045 |
| SARS-CoV-2_circ_Homo_sapiens_2474 | MN908947.3 | 24027 | 26775 | - | ORF3a;S;M;E | 2 | find_circ | SRR11550044 |
| SARS-CoV-2_circ_Homo_sapiens_2475 | MN908947.3 | 24027 | 26775 | + | ORF3a;S;M;E | 2 | circRNA_finder | SRR11550044 |
| SARS-CoV-2_circ_Homo_sapiens_2476 | MN908947.3 | 24027 | 27015 | - | ORF3a;S;M;E | 2 | find_circ | SRR11550046 |
| SARS-CoV-2_circ_Homo_sapiens_2478 | MN908947.3 | 24027 | 28092 | - | ORF6;M;E;ORF3a;S;ORF8;ORF7a;ORF7b | 2 | find_circ | SRR11550046 |
| SARS-CoV-2_circ_Homo_sapiens_2479 | MN908947.3 | 24125 | 25428 | + | ORF3a;S | 2 | find_circ | SRR11550044 |
| SARS-CoV-2_circ_Homo_sapiens_248 | MN908947.3 | 1026 | 1403 | - | ORF1ab | 2 | find_circ | SRR11550046 |
| SARS-CoV-2_circ_Homo_sapiens_2480 | MN908947.3 | 24125 | 25659 | + | ORF3a;S | 2 | find_circ | SRR11550046 |
| SARS-CoV-2_circ_Homo_sapiens_2486 | MN908947.3 | 24192 | 28059 | - | ORF6;M;E;ORF3a;S;ORF8;ORF7a;ORF7b | 2 | find_circ | SRR11550045 |
| SARS-CoV-2_circ_Homo_sapiens_2487 | MN908947.3 | 24198 | 24711 | + | S | 2 | circRNA_finder | SRR11550046 |
| SARS-CoV-2_circ_Homo_sapiens_2488 | MN908947.3 | 24198 | 24763 | + | S | 2 | find_circ | SRR11550046 |
| SARS-CoV-2_circ_Homo_sapiens_249 | MN908947.3 | 1026 | 1403 | + | ORF1ab | 2 | circRNA_finder | SRR11550046 |
| SARS-CoV-2_circ_Homo_sapiens_2494 | MN908947.3 | 24198 | 26216 | + | ORF3a;S | 2 | find_circ | SRR11550046 |
| SARS-CoV-2_circ_Homo_sapiens_2494 | MN908947.3 | 24198 | 26216 | + | ORF3a;S | 2 | circRNA_finder | SRR11550046 |
| SARS-CoV-2_circ_Homo_sapiens_2496 | MN908947.3 | 24277 | 24925 | + | S | 2 | circRNA_finder | SRR11550046 |
| SARS-CoV-2_circ_Homo_sapiens_2496 | MN908947.3 | 24277 | 24925 | + | S | 2 | find_circ | SRR11550046 |
| SARS-CoV-2_circ_Homo_sapiens_25 | MN908947.3 | 8 | 23066 | + | S;ORF1ab | 2 | find_circ | SRR11550043 |
| SARS-CoV-2_circ_Homo_sapiens_2501 | MN908947.3 | 24349 | 24932 | + | S | 2 | find_circ | SRR11550045 |
| SARS-CoV-2_circ_Homo_sapiens_2501 | MN908947.3 | 24349 | 24932 | + | S | 2 | circRNA_finder | SRR11550045 |
| SARS-CoV-2_circ_Homo_sapiens_2502 | MN908947.3 | 24375 | 24864 | - | S | 2 | find_circ | SRR11550045 |
| SARS-CoV-2_circ_Homo_sapiens_2504 | MN908947.3 | 24375 | 24989 | + | S | 2 | circRNA_finder | SRR11550045 |
| SARS-CoV-2_circ_Homo_sapiens_2506 | MN908947.3 | 24375 | 25548 | - | ORF3a;S | 2 | find_circ | SRR11550046 |
| SARS-CoV-2_circ_Homo_sapiens_2510 | MN908947.3 | 24456 | 25727 | - | ORF3a;S | 2 | find_circ | SRR11550046 |
| SARS-CoV-2_circ_Homo_sapiens_2512 | MN908947.3 | 24456 | 29268 | - | ORF6;M;E;ORF3a;S;N;ORF8;ORF7a;ORF7b | 2 | find_circ | SRR11550045 |
| SARS-CoV-2_circ_Homo_sapiens_2516 | MN908947.3 | 24463 | 24925 | + | S | 2 | circRNA_finder | SRR11550046 |
| SARS-CoV-2_circ_Homo_sapiens_2516 | MN908947.3 | 24463 | 24925 | + | S | 2 | find_circ | SRR11550045 |
| SARS-CoV-2_circ_Homo_sapiens_2516 | MN908947.3 | 24463 | 24925 | + | S | 2 | find_circ | SRR11550046 |
| SARS-CoV-2_circ_Homo_sapiens_2517 | MN908947.3 | 24463 | 24927 | + | S | 2 | find_circ | SRR11550046 |
| SARS-CoV-2_circ_Homo_sapiens_2518 | MN908947.3 | 24463 | 25061 | + | S | 2 | circRNA_finder | SRR11550043 |
| SARS-CoV-2_circ_Homo_sapiens_2518 | MN908947.3 | 24463 | 25061 | + | S | 2 | find_circ | SRR11550043 |
| SARS-CoV-2_circ_Homo_sapiens_2519 | MN908947.3 | 24463 | 25304 | + | S | 2 | find_circ | SRR11550043 |
| SARS-CoV-2_circ_Homo_sapiens_252 | MN908947.3 | 1026 | 5054 | - | ORF1ab | 2 | CIRI2 | SRR11550046 |
| SARS-CoV-2_circ_Homo_sapiens_2521 | MN908947.3 | 24463 | 25533 | + | ORF3a;S | 2 | find_circ | SRR11550046 |
| SARS-CoV-2_circ_Homo_sapiens_2522 | MN908947.3 | 24463 | 26246 | + | ORF3a;S;E | 2 | find_circ | SRR11550043 |
| SARS-CoV-2_circ_Homo_sapiens_2522 | MN908947.3 | 24463 | 26246 | + | ORF3a;S;E | 2 | circRNA_finder | SRR11550043 |
| SARS-CoV-2_circ_Homo_sapiens_2524 | MN908947.3 | 24487 | 24871 | + | S | 2 | find_circ | SRR11550045 |
| SARS-CoV-2_circ_Homo_sapiens_2532 | MN908947.3 | 24698 | 26778 | + | ORF3a;S;M;E | 2 | find_circ | SRR11550045 |
| SARS-CoV-2_circ_Homo_sapiens_2534 | MN908947.3 | 24760 | 25516 | - | ORF3a;S | 2 | find_circ | SRR11550045 |
| SARS-CoV-2_circ_Homo_sapiens_2540 | MN908947.3 | 24913 | 25281 | - | S | 2 | find_circ | SRR11550044 |
| SARS-CoV-2_circ_Homo_sapiens_2541 | MN908947.3 | 24913 | 25281 | + | S | 2 | circRNA_finder | SRR11550044 |
| SARS-CoV-2_circ_Homo_sapiens_2543 | MN908947.3 | 24922 | 25389 | - | S | 2 | find_circ | SRR11550045 |
| SARS-CoV-2_circ_Homo_sapiens_2544 | MN908947.3 | 24922 | 25389 | + | S | 2 | circRNA_finder | SRR11550045 |
| SARS-CoV-2_circ_Homo_sapiens_255 | MN908947.3 | 1162 | 17260 | + | ORF1ab | 2 | find_circ | SRR11550043 |
| SARS-CoV-2_circ_Homo_sapiens_2558 | MN908947.3 | 24972 | 25243 | + | S | 2 | find_circ | SRR11550046 |
| SARS-CoV-2_circ_Homo_sapiens_2561 | MN908947.3 | 24990 | 25516 | - | ORF3a;S | 2 | find_circ | SRR11550045 |
| SARS-CoV-2_circ_Homo_sapiens_2564 | MN908947.3 | 24990 | 25548 | + | ORF3a;S | 2 | circRNA_finder | SRR11550045 |
| SARS-CoV-2_circ_Homo_sapiens_2564 | MN908947.3 | 24990 | 25548 | + | ORF3a;S | 2 | circRNA_finder | SRR11550046 |
| SARS-CoV-2_circ_Homo_sapiens_2568 | MN908947.3 | 24990 | 26212 | - | ORF3a;S | 2 | find_circ | SRR11550046 |
| SARS-CoV-2_circ_Homo_sapiens_2568 | MN908947.3 | 24990 | 26212 | - | ORF3a;S | 2 | find_circ | SRR11550045 |
| SARS-CoV-2_circ_Homo_sapiens_2569 | MN908947.3 | 24990 | 26212 | + | ORF3a;S | 2 | circRNA_finder | SRR11550045 |
| SARS-CoV-2_circ_Homo_sapiens_257 | MN908947.3 | 1315 | 2155 | - | ORF1ab | 2 | find_circ | SRR11550045 |
| SARS-CoV-2_circ_Homo_sapiens_2575 | MN908947.3 | 24999 | 25476 | + | ORF3a;S | 2 | circRNA_finder | SRR11550046 |
| SARS-CoV-2_circ_Homo_sapiens_2575 | MN908947.3 | 24999 | 25476 | + | ORF3a;S | 2 | find_circ | SRR11550045 |
| SARS-CoV-2_circ_Homo_sapiens_2577 | MN908947.3 | 25001 | 25307 | - | S | 2 | find_circ | SRR11550045 |
| SARS-CoV-2_circ_Homo_sapiens_2578 | MN908947.3 | 25001 | 25426 | - | ORF3a;S | 2 | find_circ | SRR11550045 |
| SARS-CoV-2_circ_Homo_sapiens_2579 | MN908947.3 | 25001 | 25432 | - | ORF3a;S | 2 | find_circ | SRR11550046 |
| SARS-CoV-2_circ_Homo_sapiens_258 | MN908947.3 | 1320 | 3337 | + | ORF1ab | 2 | find_circ | SRR11550044 |
| SARS-CoV-2_circ_Homo_sapiens_258 | MN908947.3 | 1320 | 3337 | + | ORF1ab | 2 | find_circ | SRR11550046 |
| SARS-CoV-2_circ_Homo_sapiens_2581 | MN908947.3 | 25016 | 25554 | + | ORF3a;S | 2 | find_circ | SRR11550043 |
| SARS-CoV-2_circ_Homo_sapiens_2582 | MN908947.3 | 25016 | 26157 | + | ORF3a;S | 2 | find_circ | SRR11550045 |
| SARS-CoV-2_circ_Homo_sapiens_2587 | MN908947.3 | 25020 | 25533 | + | ORF3a;S | 2 | find_circ | SRR11550043 |
| SARS-CoV-2_circ_Homo_sapiens_2588 | MN908947.3 | 25020 | 26154 | + | ORF3a;S | 2 | find_circ | SRR11550045 |
| SARS-CoV-2_circ_Homo_sapiens_259 | MN908947.3 | 1323 | 1523 | + | ORF1ab | 2 | find_circ | SRR11550044 |
| SARS-CoV-2_circ_Homo_sapiens_2590 | MN908947.3 | 25034 | 25476 | + | ORF3a;S | 2 | find_circ | SRR11550046 |
| SARS-CoV-2_circ_Homo_sapiens_2591 | MN908947.3 | 25047 | 26298 | + | ORF3a;S;E | 2 | circRNA_finder | SRR11550046 |
| SARS-CoV-2_circ_Homo_sapiens_2597 | MN908947.3 | 25255 | 25516 | - | ORF3a;S | 2 | find_circ | SRR11550044 |
| SARS-CoV-2_circ_Homo_sapiens_2598 | MN908947.3 | 25276 | 25641 | - | ORF3a;S | 2 | find_circ | SRR11550045 |
| SARS-CoV-2_circ_Homo_sapiens_2599 | MN908947.3 | 25276 | 26946 | - | ORF3a;S;M;E | 2 | find_circ | SRR11550043 |
| SARS-CoV-2_circ_Homo_sapiens_2600 | MN908947.3 | 25288 | 26316 | + | ORF3a;S;E | 2 | circRNA_finder | SRR11550044 |
| SARS-CoV-2_circ_Homo_sapiens_2601 | MN908947.3 | 25374 | 26780 | - | ORF3a;S;M;E | 2 | find_circ | SRR11550043 |
| SARS-CoV-2_circ_Homo_sapiens_2602 | MN908947.3 | 25374 | 26780 | + | ORF3a;S;M;E | 2 | circRNA_finder | SRR11550043 |
| SARS-CoV-2_circ_Homo_sapiens_2604 | MN908947.3 | 25391 | 25516 | - | ORF3a | 2 | find_circ | SRR11550044 |
| SARS-CoV-2_circ_Homo_sapiens_2605 | MN908947.3 | 25391 | 25823 | - | ORF3a | 2 | find_circ | SRR11550045 |
| SARS-CoV-2_circ_Homo_sapiens_2606 | MN908947.3 | 25391 | 25823 | + | ORF3a | 2 | circRNA_finder | SRR11550045 |
| SARS-CoV-2_circ_Homo_sapiens_2608 | MN908947.3 | 25391 | 26241 | - | ORF3a | 2 | find_circ | SRR11550046 |
| SARS-CoV-2_circ_Homo_sapiens_2609 | MN908947.3 | 25391 | 26541 | - | ORF3a;M;E | 2 | find_circ | SRR11550044 |
| SARS-CoV-2_circ_Homo_sapiens_2609 | MN908947.3 | 25391 | 26541 | - | ORF3a;M;E | 2 | find_circ | SRR11550043 |
| SARS-CoV-2_circ_Homo_sapiens_2610 | MN908947.3 | 25391 | 26541 | + | ORF3a;M;E | 2 | circRNA_finder | SRR11550044 |
| SARS-CoV-2_circ_Homo_sapiens_2611 | MN908947.3 | 25391 | 26780 | - | ORF3a;M;E | 2 | find_circ | SRR11550046 |
| SARS-CoV-2_circ_Homo_sapiens_2612 | MN908947.3 | 25391 | 26817 | - | ORF3a;M;E | 2 | find_circ | SRR11550044 |
| SARS-CoV-2_circ_Homo_sapiens_2612 | MN908947.3 | 25391 | 26817 | - | ORF3a;M;E | 2 | find_circ | SRR11550045 |
| SARS-CoV-2_circ_Homo_sapiens_2612 | MN908947.3 | 25391 | 26817 | - | ORF3a;M;E | 2 | find_circ | SRR11550043 |
| SARS-CoV-2_circ_Homo_sapiens_2614 | MN908947.3 | 25391 | 27015 | - | ORF3a;M;E | 2 | find_circ | SRR11550046 |
| SARS-CoV-2_circ_Homo_sapiens_2614 | MN908947.3 | 25391 | 27015 | - | ORF3a;M;E | 2 | find_circ | SRR11550045 |
| SARS-CoV-2_circ_Homo_sapiens_2615 | MN908947.3 | 25391 | 27015 | + | ORF3a;M;E | 2 | circRNA_finder | SRR11550045 |
| SARS-CoV-2_circ_Homo_sapiens_2618 | MN908947.3 | 25391 | 27262 | - | ORF6;ORF3a;M;E | 2 | find_circ | SRR11550045 |
| SARS-CoV-2_circ_Homo_sapiens_2626 | MN908947.3 | 25441 | 27702 | + | ORF6;M;E;ORF3a;ORF7a | 2 | find_circ | SRR11550046 |
| SARS-CoV-2_circ_Homo_sapiens_2628 | MN908947.3 | 25456 | 26246 | + | ORF3a;E | 2 | circRNA_finder | SRR11550045 |
| SARS-CoV-2_circ_Homo_sapiens_2628 | MN908947.3 | 25456 | 26246 | + | ORF3a;E | 2 | find_circ | SRR11550045 |
| SARS-CoV-2_circ_Homo_sapiens_2629 | MN908947.3 | 25464 | 27541 | - | ORF6;M;E;ORF3a;ORF7a | 2 | find_circ | SRR11550045 |
| SARS-CoV-2_circ_Homo_sapiens_2630 | MN908947.3 | 25464 | 27541 | + | ORF6;M;E;ORF3a;ORF7a | 2 | circRNA_finder | SRR11550045 |
| SARS-CoV-2_circ_Homo_sapiens_2631 | MN908947.3 | 25464 | 29481 | - | ORF6;M;E;ORF3a;N;ORF8;ORF7a;ORF7b | 2 | find_circ | SRR11550046 |
| SARS-CoV-2_circ_Homo_sapiens_2632 | MN908947.3 | 25464 | 29481 | + | ORF6;M;E;ORF3a;N;ORF8;ORF7a;ORF7b | 2 | circRNA_finder | SRR11550046 |
| SARS-CoV-2_circ_Homo_sapiens_2633 | MN908947.3 | 25472 | 26157 | + | ORF3a | 2 | find_circ | SRR11550044 |
| SARS-CoV-2_circ_Homo_sapiens_2633 | MN908947.3 | 25472 | 26157 | + | ORF3a | 2 | circRNA_finder | SRR11550044 |
| SARS-CoV-2_circ_Homo_sapiens_2635 | MN908947.3 | 25547 | 25781 | - | ORF3a | 2 | find_circ | SRR11550046 |
| SARS-CoV-2_circ_Homo_sapiens_2637 | MN908947.3 | 25547 | 26298 | - | ORF3a;E | 2 | find_circ | SRR11550046 |
| SARS-CoV-2_circ_Homo_sapiens_2639 | MN908947.3 | 25547 | 26541 | - | ORF3a;M;E | 2 | find_circ | SRR11550046 |
| SARS-CoV-2_circ_Homo_sapiens_2640 | MN908947.3 | 25547 | 27790 | - | ORF6;M;E;ORF3a;ORF7a;ORF7b | 2 | find_circ | SRR11550046 |
| SARS-CoV-2_circ_Homo_sapiens_2642 | MN908947.3 | 25584 | 26788 | - | ORF3a;M;E | 2 | find_circ | SRR11550045 |
| SARS-CoV-2_circ_Homo_sapiens_2643 | MN908947.3 | 25607 | 26234 | + | ORF3a | 2 | find_circ | SRR11550046 |
| SARS-CoV-2_circ_Homo_sapiens_2651 | MN908947.3 | 25627 | 26814 | - | ORF3a;M;E | 2 | find_circ | SRR11550045 |
| SARS-CoV-2_circ_Homo_sapiens_2653 | MN908947.3 | 25627 | 29444 | - | ORF6;M;E;ORF3a;N;ORF8;ORF7a;ORF7b | 2 | find_circ | SRR11550043 |
| SARS-CoV-2_circ_Homo_sapiens_2654 | MN908947.3 | 25659 | 26212 | - | ORF3a | 2 | find_circ | SRR11550043 |
| SARS-CoV-2_circ_Homo_sapiens_2655 | MN908947.3 | 25659 | 26621 | - | ORF3a;M;E | 2 | find_circ | SRR11550044 |
| SARS-CoV-2_circ_Homo_sapiens_2656 | MN908947.3 | 25659 | 26788 | - | ORF3a;M;E | 2 | find_circ | SRR11550045 |
| SARS-CoV-2_circ_Homo_sapiens_2665 | MN908947.3 | 25672 | 26203 | - | ORF3a | 2 | find_circ | SRR11550043 |
| SARS-CoV-2_circ_Homo_sapiens_2666 | MN908947.3 | 25672 | 26203 | + | ORF3a | 2 | circRNA_finder | SRR11550043 |
| SARS-CoV-2_circ_Homo_sapiens_2666 | MN908947.3 | 25672 | 26203 | + | ORF3a | 2 | circRNA_finder | SRR11550045 |
| SARS-CoV-2_circ_Homo_sapiens_2667 | MN908947.3 | 25672 | 26212 | - | ORF3a | 2 | find_circ | SRR11550043 |
| SARS-CoV-2_circ_Homo_sapiens_2668 | MN908947.3 | 25672 | 26212 | + | ORF3a | 2 | circRNA_finder | SRR11550044 |
| SARS-CoV-2_circ_Homo_sapiens_2677 | MN908947.3 | 25672 | 26343 | + | ORF3a;E | 2 | circRNA_finder | SRR11550046 |
| SARS-CoV-2_circ_Homo_sapiens_2682 | MN908947.3 | 25672 | 26407 | - | ORF3a;E | 2 | CIRI2 | SRR11550046 |
| SARS-CoV-2_circ_Homo_sapiens_2683 | MN908947.3 | 25672 | 26541 | - | ORF3a;M;E | 2 | find_circ | SRR11550043 |
| SARS-CoV-2_circ_Homo_sapiens_2683 | MN908947.3 | 25672 | 26541 | - | ORF3a;M;E | 2 | find_circ | SRR11550044 |
| SARS-CoV-2_circ_Homo_sapiens_2684 | MN908947.3 | 25672 | 26541 | + | ORF3a;M;E | 2 | circRNA_finder | SRR11550043 |
| SARS-CoV-2_circ_Homo_sapiens_2684 | MN908947.3 | 25672 | 26541 | + | ORF3a;M;E | 2 | circRNA_finder | SRR11550044 |
| SARS-CoV-2_circ_Homo_sapiens_2690 | MN908947.3 | 25672 | 26775 | - | ORF3a;M;E | 2 | find_circ | SRR11550043 |
| SARS-CoV-2_circ_Homo_sapiens_2690 | MN908947.3 | 25672 | 26775 | - | ORF3a;M;E | 2 | find_circ | SRR11550044 |
| SARS-CoV-2_circ_Homo_sapiens_2693 | MN908947.3 | 25672 | 26780 | + | ORF3a;M;E | 2 | circRNA_finder | SRR11550043 |
| SARS-CoV-2_circ_Homo_sapiens_2698 | MN908947.3 | 25672 | 26868 | + | ORF3a;M;E | 2 | circRNA_finder | SRR11550045 |
| SARS-CoV-2_circ_Homo_sapiens_27 | MN908947.3 | 8 | 24139 | + | S;ORF1ab | 2 | find_circ | SRR11550043 |
| SARS-CoV-2_circ_Homo_sapiens_2700 | MN908947.3 | 25672 | 26906 | - | ORF3a;M;E | 2 | find_circ | SRR11550043 |
| SARS-CoV-2_circ_Homo_sapiens_2701 | MN908947.3 | 25672 | 26906 | + | ORF3a;M;E | 2 | circRNA_finder | SRR11550043 |
| SARS-CoV-2_circ_Homo_sapiens_2703 | MN908947.3 | 25672 | 27015 | - | ORF3a;M;E | 2 | find_circ | SRR11550043 |
| SARS-CoV-2_circ_Homo_sapiens_2703 | MN908947.3 | 25672 | 27015 | - | ORF3a;M;E | 2 | find_circ | SRR11550044 |
| SARS-CoV-2_circ_Homo_sapiens_2705 | MN908947.3 | 25672 | 27047 | - | ORF3a;M;E | 2 | find_circ | SRR11550044 |
| SARS-CoV-2_circ_Homo_sapiens_2706 | MN908947.3 | 25672 | 27047 | + | ORF3a;M;E | 2 | circRNA_finder | SRR11550044 |
| SARS-CoV-2_circ_Homo_sapiens_271 | MN908947.3 | 1414 | 1771 | + | ORF1ab | 2 | find_circ | SRR11550045 |
| SARS-CoV-2_circ_Homo_sapiens_272 | MN908947.3 | 1427 | 1600 | - | ORF1ab | 2 | find_circ | SRR11550045 |
| SARS-CoV-2_circ_Homo_sapiens_2723 | MN908947.3 | 25727 | 26216 | + | ORF3a | 2 | find_circ | SRR11550046 |
| SARS-CoV-2_circ_Homo_sapiens_2724 | MN908947.3 | 25727 | 26246 | + | ORF3a;E | 2 | find_circ | SRR11550046 |
| SARS-CoV-2_circ_Homo_sapiens_2724 | MN908947.3 | 25727 | 26246 | + | ORF3a;E | 2 | circRNA_finder | SRR11550046 |
| SARS-CoV-2_circ_Homo_sapiens_2724 | MN908947.3 | 25727 | 26246 | + | ORF3a;E | 2 | find_circ | SRR11550045 |
| SARS-CoV-2_circ_Homo_sapiens_2724 | MN908947.3 | 25727 | 26246 | + | ORF3a;E | 2 | circRNA_finder | SRR11550043 |
| SARS-CoV-2_circ_Homo_sapiens_2740 | MN908947.3 | 25811 | 27130 | - | ORF3a;M;E | 2 | find_circ | SRR11550044 |
| SARS-CoV-2_circ_Homo_sapiens_2742 | MN908947.3 | 25825 | 26298 | - | ORF3a;E | 2 | find_circ | SRR11550044 |
| SARS-CoV-2_circ_Homo_sapiens_2749 | MN908947.3 | 26092 | 26558 | - | ORF3a;M;E | 2 | find_circ | SRR11550045 |
| SARS-CoV-2_circ_Homo_sapiens_2749 | MN908947.3 | 26092 | 26558 | - | ORF3a;M;E | 2 | find_circ | SRR11550046 |
| SARS-CoV-2_circ_Homo_sapiens_2750 | MN908947.3 | 26092 | 26570 | - | ORF3a;M;E | 2 | find_circ | SRR11550046 |
| SARS-CoV-2_circ_Homo_sapiens_2751 | MN908947.3 | 26156 | 26538 | + | ORF3a;M;E | 2 | find_circ | SRR11550046 |
| SARS-CoV-2_circ_Homo_sapiens_2752 | MN908947.3 | 26156 | 26778 | + | ORF3a;M;E | 2 | find_circ | SRR11550043 |
| SARS-CoV-2_circ_Homo_sapiens_2758 | MN908947.3 | 26243 | 26780 | - | M;E | 2 | find_circ | SRR11550043 |
| SARS-CoV-2_circ_Homo_sapiens_2762 | MN908947.3 | 26285 | 26778 | + | M;E | 2 | find_circ | SRR11550046 |
| SARS-CoV-2_circ_Homo_sapiens_2763 | MN908947.3 | 26285 | 26783 | + | M;E | 2 | find_circ | SRR11550045 |
| SARS-CoV-2_circ_Homo_sapiens_2763 | MN908947.3 | 26285 | 26783 | + | M;E | 2 | circRNA_finder | SRR11550045 |
| SARS-CoV-2_circ_Homo_sapiens_2764 | MN908947.3 | 26285 | 26948 | + | M;E | 2 | find_circ | SRR11550044 |
| SARS-CoV-2_circ_Homo_sapiens_2768 | MN908947.3 | 26292 | 26671 | + | M;E | 2 | find_circ | SRR11550043 |
| SARS-CoV-2_circ_Homo_sapiens_2769 | MN908947.3 | 26405 | 26567 | - | M;E | 2 | find_circ | SRR11550043 |
| SARS-CoV-2_circ_Homo_sapiens_2772 | MN908947.3 | 26510 | 26876 | - | M | 2 | find_circ | SRR11550044 |
| SARS-CoV-2_circ_Homo_sapiens_2773 | MN908947.3 | 26510 | 26876 | + | M | 2 | circRNA_finder | SRR11550044 |
| SARS-CoV-2_circ_Homo_sapiens_2777 | MN908947.3 | 26578 | 27210 | - | ORF6;M | 2 | find_circ | SRR11550046 |
| SARS-CoV-2_circ_Homo_sapiens_278 | MN908947.3 | 1463 | 24473 | + | S;ORF1ab | 2 | find_circ | SRR11550044 |
| SARS-CoV-2_circ_Homo_sapiens_278 | MN908947.3 | 1463 | 24473 | + | S;ORF1ab | 2 | circRNA_finder | SRR11550044 |
| SARS-CoV-2_circ_Homo_sapiens_2780 | MN908947.3 | 26612 | 27130 | - | M | 2 | find_circ | SRR11550043 |
| SARS-CoV-2_circ_Homo_sapiens_2788 | MN908947.3 | 26705 | 29787 | - | ORF6;ORF10;M;N;ORF8;ORF7a;ORF7b | 2 | find_circ | SRR11550043 |
| SARS-CoV-2_circ_Homo_sapiens_279 | MN908947.3 | 1520 | 2090 | - | ORF1ab | 2 | find_circ | SRR11550046 |
| SARS-CoV-2_circ_Homo_sapiens_2790 | MN908947.3 | 26710 | 27669 | + | ORF7a;ORF6;M | 2 | find_circ | SRR11550043 |
| SARS-CoV-2_circ_Homo_sapiens_2790 | MN908947.3 | 26710 | 27669 | + | ORF7a;ORF6;M | 2 | circRNA_finder | SRR11550043 |
| SARS-CoV-2_circ_Homo_sapiens_2791 | MN908947.3 | 26738 | 27225 | + | ORF6;M | 2 | circRNA_finder | SRR11550043 |
| SARS-CoV-2_circ_Homo_sapiens_2791 | MN908947.3 | 26738 | 27225 | + | ORF6;M | 2 | find_circ | SRR11550043 |
| SARS-CoV-2_circ_Homo_sapiens_2792 | MN908947.3 | 26788 | 27191 | + | M | 2 | find_circ | SRR11550045 |
| SARS-CoV-2_circ_Homo_sapiens_2794 | MN908947.3 | 26808 | 27051 | - | M | 2 | find_circ | SRR11550046 |
| SARS-CoV-2_circ_Homo_sapiens_2799 | MN908947.3 | 26827 | 27047 | - | M | 2 | find_circ | SRR11550043 |
| SARS-CoV-2_circ_Homo_sapiens_28 | MN908947.3 | 8 | 25554 | + | ORF3a;S;ORF1ab | 2 | find_circ | SRR11550045 |
| SARS-CoV-2_circ_Homo_sapiens_280 | MN908947.3 | 1520 | 2155 | - | ORF1ab | 2 | find_circ | SRR11550043 |
| SARS-CoV-2_circ_Homo_sapiens_280 | MN908947.3 | 1520 | 2155 | - | ORF1ab | 2 | find_circ | SRR11550046 |
| SARS-CoV-2_circ_Homo_sapiens_2800 | MN908947.3 | 26827 | 27174 | - | M | 2 | find_circ | SRR11550045 |
| SARS-CoV-2_circ_Homo_sapiens_2801 | MN908947.3 | 26827 | 27218 | - | ORF6;M | 2 | find_circ | SRR11550045 |
| SARS-CoV-2_circ_Homo_sapiens_2803 | MN908947.3 | 26827 | 27480 | - | ORF7a;ORF6;M | 2 | find_circ | SRR11550044 |
| SARS-CoV-2_circ_Homo_sapiens_2804 | MN908947.3 | 26827 | 27541 | - | ORF7a;ORF6;M | 2 | find_circ | SRR11550043 |
| SARS-CoV-2_circ_Homo_sapiens_2805 | MN908947.3 | 26827 | 27541 | + | ORF7a;ORF6;M | 2 | circRNA_finder | SRR11550043 |
| SARS-CoV-2_circ_Homo_sapiens_2806 | MN908947.3 | 26827 | 27566 | - | ORF7a;ORF6;M | 2 | find_circ | SRR11550044 |
| SARS-CoV-2_circ_Homo_sapiens_2807 | MN908947.3 | 26827 | 27678 | - | ORF7a;ORF6;M | 2 | find_circ | SRR11550045 |
| SARS-CoV-2_circ_Homo_sapiens_2811 | MN908947.3 | 26870 | 27285 | - | ORF6;M | 2 | find_circ | SRR11550046 |
| SARS-CoV-2_circ_Homo_sapiens_2812 | MN908947.3 | 26874 | 27218 | - | ORF6;M | 2 | find_circ | SRR11550046 |
| SARS-CoV-2_circ_Homo_sapiens_2815 | MN908947.3 | 26874 | 27583 | - | ORF7a;ORF6;M | 2 | find_circ | SRR11550045 |
| SARS-CoV-2_circ_Homo_sapiens_2816 | MN908947.3 | 26874 | 27678 | - | ORF7a;ORF6;M | 2 | find_circ | SRR11550045 |
| SARS-CoV-2_circ_Homo_sapiens_2817 | MN908947.3 | 26874 | 27678 | + | ORF7a;ORF6;M | 2 | circRNA_finder | SRR11550045 |
| SARS-CoV-2_circ_Homo_sapiens_2819 | MN908947.3 | 26874 | 27696 | - | ORF7a;ORF6;M | 2 | find_circ | SRR11550046 |
| SARS-CoV-2_circ_Homo_sapiens_282 | MN908947.3 | 1520 | 2484 | + | ORF1ab | 2 | circRNA_finder | SRR11550045 |
| SARS-CoV-2_circ_Homo_sapiens_283 | MN908947.3 | 1520 | 2535 | - | ORF1ab | 2 | find_circ | SRR11550044 |
| SARS-CoV-2_circ_Homo_sapiens_2834 | MN908947.3 | 27060 | 27229 | - | ORF6;M | 2 | find_circ | SRR11550046 |
| SARS-CoV-2_circ_Homo_sapiens_2835 | MN908947.3 | 27060 | 27541 | + | ORF7a;ORF6;M | 2 | circRNA_finder | SRR11550045 |
| SARS-CoV-2_circ_Homo_sapiens_2836 | MN908947.3 | 27060 | 27583 | - | ORF7a;ORF6;M | 2 | find_circ | SRR11550043 |
| SARS-CoV-2_circ_Homo_sapiens_2836 | MN908947.3 | 27060 | 27583 | - | ORF7a;ORF6;M | 2 | find_circ | SRR11550046 |
| SARS-CoV-2_circ_Homo_sapiens_2838 | MN908947.3 | 27060 | 27683 | - | ORF7a;ORF6;M | 2 | find_circ | SRR11550044 |
| SARS-CoV-2_circ_Homo_sapiens_2840 | MN908947.3 | 27060 | 27696 | - | ORF7a;ORF6;M | 2 | find_circ | SRR11550043 |
| SARS-CoV-2_circ_Homo_sapiens_2840 | MN908947.3 | 27060 | 27696 | - | ORF7a;ORF6;M | 2 | find_circ | SRR11550044 |
| SARS-CoV-2_circ_Homo_sapiens_2843 | MN908947.3 | 27060 | 28146 | - | ORF6;M;ORF8;ORF7a;ORF7b | 2 | find_circ | SRR11550043 |
| SARS-CoV-2_circ_Homo_sapiens_2844 | MN908947.3 | 27060 | 28146 | + | ORF6;M;ORF8;ORF7a;ORF7b | 2 | circRNA_finder | SRR11550043 |
| SARS-CoV-2_circ_Homo_sapiens_285 | MN908947.3 | 1520 | 3014 | - | ORF1ab | 2 | find_circ | SRR11550046 |
| SARS-CoV-2_circ_Homo_sapiens_2850 | MN908947.3 | 27093 | 27696 | - | ORF7a;ORF6;M | 2 | find_circ | SRR11550045 |
| SARS-CoV-2_circ_Homo_sapiens_2856 | MN908947.3 | 27144 | 28343 | - | ORF6;M;N;ORF8;ORF7a;ORF7b | 2 | find_circ | SRR11550044 |
| SARS-CoV-2_circ_Homo_sapiens_2857 | MN908947.3 | 27144 | 28418 | - | ORF6;M;N;ORF8;ORF7a;ORF7b | 2 | find_circ | SRR11550046 |
| SARS-CoV-2_circ_Homo_sapiens_2860 | MN908947.3 | 27144 | 29408 | - | ORF6;M;N;ORF8;ORF7a;ORF7b | 2 | find_circ | SRR11550044 |
| SARS-CoV-2_circ_Homo_sapiens_2865 | MN908947.3 | 27193 | 27625 | + | ORF7a;ORF6 | 2 | circRNA_finder | SRR11550043 |
| SARS-CoV-2_circ_Homo_sapiens_2865 | MN908947.3 | 27193 | 27625 | + | ORF7a;ORF6 | 2 | find_circ | SRR11550043 |
| SARS-CoV-2_circ_Homo_sapiens_2866 | MN908947.3 | 27193 | 29746 | + | ORF6;ORF10;N;ORF8;ORF7a;ORF7b | 2 | find_circ | SRR11550045 |
| SARS-CoV-2_circ_Homo_sapiens_2867 | MN908947.3 | 27202 | 27620 | + | ORF7a;ORF6 | 2 | find_circ | SRR11550043 |
| SARS-CoV-2_circ_Homo_sapiens_287 | MN908947.3 | 1520 | 4814 | - | ORF1ab | 2 | find_circ | SRR11550045 |
| SARS-CoV-2_circ_Homo_sapiens_2870 | MN908947.3 | 27220 | 27558 | - | ORF7a;ORF6 | 2 | find_circ | SRR11550043 |
| SARS-CoV-2_circ_Homo_sapiens_2874 | MN908947.3 | 27220 | 27696 | + | ORF7a;ORF6 | 2 | circRNA_finder | SRR11550045 |
| SARS-CoV-2_circ_Homo_sapiens_2875 | MN908947.3 | 27220 | 27781 | - | ORF7a;ORF6;ORF7b | 2 | find_circ | SRR11550046 |
| SARS-CoV-2_circ_Homo_sapiens_2876 | MN908947.3 | 27220 | 28059 | - | ORF7a;ORF6;ORF8;ORF7b | 2 | find_circ | SRR11550043 |
| SARS-CoV-2_circ_Homo_sapiens_2877 | MN908947.3 | 27220 | 29707 | - | ORF6;ORF10;N;ORF8;ORF7a;ORF7b | 2 | find_circ | SRR11550044 |
| SARS-CoV-2_circ_Homo_sapiens_2879 | MN908947.3 | 27226 | 27603 | + | ORF7a;ORF6 | 2 | circRNA_finder | SRR11550045 |
| SARS-CoV-2_circ_Homo_sapiens_2889 | MN908947.3 | 27231 | 27683 | - | ORF7a;ORF6 | 2 | find_circ | SRR11550043 |
| SARS-CoV-2_circ_Homo_sapiens_2891 | MN908947.3 | 27264 | 27535 | - | ORF7a;ORF6 | 2 | find_circ | SRR11550044 |
| SARS-CoV-2_circ_Homo_sapiens_2892 | MN908947.3 | 27264 | 27537 | - | ORF7a;ORF6 | 2 | find_circ | SRR11550045 |
| SARS-CoV-2_circ_Homo_sapiens_2896 | MN908947.3 | 27264 | 27678 | - | ORF7a;ORF6 | 2 | find_circ | SRR11550046 |
| SARS-CoV-2_circ_Homo_sapiens_2897 | MN908947.3 | 27264 | 27678 | + | ORF7a;ORF6 | 2 | circRNA_finder | SRR11550046 |
| SARS-CoV-2_circ_Homo_sapiens_2898 | MN908947.3 | 27264 | 27696 | - | ORF7a;ORF6 | 2 | find_circ | SRR11550043 |
| SARS-CoV-2_circ_Homo_sapiens_290 | MN908947.3 | 1542 | 2570 | - | ORF1ab | 2 | find_circ | SRR11550045 |
| SARS-CoV-2_circ_Homo_sapiens_2902 | MN908947.3 | 27264 | 28727 | - | ORF6;N;ORF8;ORF7a;ORF7b | 2 | find_circ | SRR11550044 |
| SARS-CoV-2_circ_Homo_sapiens_2904 | MN908947.3 | 27272 | 27650 | + | ORF7a;ORF6 | 2 | find_circ | SRR11550046 |
| SARS-CoV-2_circ_Homo_sapiens_2905 | MN908947.3 | 27295 | 27583 | - | ORF7a;ORF6 | 2 | find_circ | SRR11550043 |
| SARS-CoV-2_circ_Homo_sapiens_2907 | MN908947.3 | 27295 | 27683 | - | ORF7a;ORF6 | 2 | find_circ | SRR11550045 |
| SARS-CoV-2_circ_Homo_sapiens_2907 | MN908947.3 | 27295 | 27683 | - | ORF7a;ORF6 | 2 | find_circ | SRR11550046 |
| SARS-CoV-2_circ_Homo_sapiens_2909 | MN908947.3 | 27295 | 27696 | - | ORF7a;ORF6 | 2 | find_circ | SRR11550043 |
| SARS-CoV-2_circ_Homo_sapiens_2909 | MN908947.3 | 27295 | 27696 | - | ORF7a;ORF6 | 2 | find_circ | SRR11550044 |
| SARS-CoV-2_circ_Homo_sapiens_2909 | MN908947.3 | 27295 | 27696 | - | ORF7a;ORF6 | 2 | find_circ | SRR11550046 |
| SARS-CoV-2_circ_Homo_sapiens_2911 | MN908947.3 | 27295 | 28527 | - | ORF6;N;ORF8;ORF7a;ORF7b | 2 | find_circ | SRR11550045 |
| SARS-CoV-2_circ_Homo_sapiens_2913 | MN908947.3 | 27295 | 29450 | - | ORF6;N;ORF8;ORF7a;ORF7b | 2 | find_circ | SRR11550045 |
| SARS-CoV-2_circ_Homo_sapiens_2914 | MN908947.3 | 27328 | 27702 | + | ORF7a;ORF6 | 2 | find_circ | SRR11550046 |
| SARS-CoV-2_circ_Homo_sapiens_292 | MN908947.3 | 1549 | 3350 | + | ORF1ab | 2 | circRNA_finder | SRR11550046 |
| SARS-CoV-2_circ_Homo_sapiens_292 | MN908947.3 | 1549 | 3350 | + | ORF1ab | 2 | find_circ | SRR11550046 |
| SARS-CoV-2_circ_Homo_sapiens_2920 | MN908947.3 | 27390 | 29268 | - | ORF7a;ORF8;ORF7b;N | 2 | find_circ | SRR11550043 |
| SARS-CoV-2_circ_Homo_sapiens_2923 | MN908947.3 | 27394 | 28092 | - | ORF7a;ORF8;ORF7b | 2 | find_circ | SRR11550046 |
| SARS-CoV-2_circ_Homo_sapiens_293 | MN908947.3 | 1549 | 23561 | + | S;ORF1ab | 2 | circRNA_finder | SRR11550043 |
| SARS-CoV-2_circ_Homo_sapiens_2932 | MN908947.3 | 27564 | 28892 | - | ORF7a;ORF8;ORF7b;N | 2 | find_circ | SRR11550045 |
| SARS-CoV-2_circ_Homo_sapiens_2933 | MN908947.3 | 27576 | 28092 | - | ORF7a;ORF8;ORF7b | 2 | find_circ | SRR11550043 |
| SARS-CoV-2_circ_Homo_sapiens_2934 | MN908947.3 | 27576 | 28602 | - | ORF7a;ORF8;ORF7b;N | 2 | find_circ | SRR11550045 |
| SARS-CoV-2_circ_Homo_sapiens_2936 | MN908947.3 | 27576 | 28727 | - | ORF7a;ORF8;ORF7b;N | 2 | find_circ | SRR11550043 |
| SARS-CoV-2_circ_Homo_sapiens_2936 | MN908947.3 | 27576 | 28727 | - | ORF7a;ORF8;ORF7b;N | 2 | find_circ | SRR11550045 |
| SARS-CoV-2_circ_Homo_sapiens_2937 | MN908947.3 | 27576 | 28895 | - | ORF7a;ORF8;ORF7b;N | 2 | find_circ | SRR11550046 |
| SARS-CoV-2_circ_Homo_sapiens_2938 | MN908947.3 | 27576 | 29012 | - | ORF7a;ORF8;ORF7b;N | 2 | find_circ | SRR11550046 |
| SARS-CoV-2_circ_Homo_sapiens_294 | MN908947.3 | 1556 | 2090 | - | ORF1ab | 2 | find_circ | SRR11550045 |
| SARS-CoV-2_circ_Homo_sapiens_2940 | MN908947.3 | 27576 | 29268 | - | ORF7a;ORF8;ORF7b;N | 2 | find_circ | SRR11550045 |
| SARS-CoV-2_circ_Homo_sapiens_2942 | MN908947.3 | 27576 | 29787 | - | ORF10;N;ORF8;ORF7a;ORF7b | 2 | find_circ | SRR11550044 |
| SARS-CoV-2_circ_Homo_sapiens_2947 | MN908947.3 | 27622 | 29572 | + | ORF10;N;ORF8;ORF7a;ORF7b | 2 | find_circ | SRR11550043 |
| SARS-CoV-2_circ_Homo_sapiens_295 | MN908947.3 | 1556 | 2484 | - | ORF1ab | 2 | find_circ | SRR11550046 |
| SARS-CoV-2_circ_Homo_sapiens_2952 | MN908947.3 | 27644 | 28092 | - | ORF7a;ORF8;ORF7b | 2 | CIRI2 | SRR11550046 |
| SARS-CoV-2_circ_Homo_sapiens_2952 | MN908947.3 | 27644 | 28092 | - | ORF7a;ORF8;ORF7b | 2 | CIRI2 | SRR11550044 |
| SARS-CoV-2_circ_Homo_sapiens_2952 | MN908947.3 | 27644 | 28092 | - | ORF7a;ORF8;ORF7b | 2 | find_circ | SRR11550043 |
| SARS-CoV-2_circ_Homo_sapiens_2953 | MN908947.3 | 27644 | 28092 | + | ORF7a;ORF8;ORF7b | 2 | circRNA_finder | SRR11550043 |
| SARS-CoV-2_circ_Homo_sapiens_2959 | MN908947.3 | 27644 | 29066 | - | ORF7a;ORF8;ORF7b;N | 2 | find_circ | SRR11550044 |
| SARS-CoV-2_circ_Homo_sapiens_296 | MN908947.3 | 1556 | 2484 | + | ORF1ab | 2 | circRNA_finder | SRR11550046 |
| SARS-CoV-2_circ_Homo_sapiens_2960 | MN908947.3 | 27644 | 29066 | + | ORF7a;ORF8;ORF7b;N | 2 | circRNA_finder | SRR11550044 |
| SARS-CoV-2_circ_Homo_sapiens_2967 | MN908947.3 | 27726 | 29450 | - | ORF7a;ORF8;ORF7b;N | 2 | find_circ | SRR11550044 |
| SARS-CoV-2_circ_Homo_sapiens_2968 | MN908947.3 | 27728 | 28092 | - | ORF7a;ORF8;ORF7b | 2 | find_circ | SRR11550046 |
| SARS-CoV-2_circ_Homo_sapiens_2970 | MN908947.3 | 27751 | 28325 | + | ORF7a;ORF8;ORF7b;N | 2 | circRNA_finder | SRR11550045 |
| SARS-CoV-2_circ_Homo_sapiens_2970 | MN908947.3 | 27751 | 28325 | + | ORF7a;ORF8;ORF7b;N | 2 | find_circ | SRR11550045 |
| SARS-CoV-2_circ_Homo_sapiens_2971 | MN908947.3 | 27766 | 28418 | - | ORF8;ORF7b;N | 2 | find_circ | SRR11550046 |
| SARS-CoV-2_circ_Homo_sapiens_2974 | MN908947.3 | 27780 | 28343 | - | ORF8;ORF7b;N | 2 | find_circ | SRR11550045 |
| SARS-CoV-2_circ_Homo_sapiens_2975 | MN908947.3 | 27780 | 29159 | - | ORF8;ORF7b;N | 2 | find_circ | SRR11550044 |
| SARS-CoV-2_circ_Homo_sapiens_2976 | MN908947.3 | 27780 | 29268 | - | ORF8;ORF7b;N | 2 | find_circ | SRR11550046 |
| SARS-CoV-2_circ_Homo_sapiens_2977 | MN908947.3 | 27780 | 29268 | + | ORF8;ORF7b;N | 2 | circRNA_finder | SRR11550046 |
| SARS-CoV-2_circ_Homo_sapiens_2978 | MN908947.3 | 27780 | 29408 | - | ORF8;ORF7b;N | 2 | find_circ | SRR11550045 |
| SARS-CoV-2_circ_Homo_sapiens_2978 | MN908947.3 | 27780 | 29408 | - | ORF8;ORF7b;N | 2 | find_circ | SRR11550046 |
| SARS-CoV-2_circ_Homo_sapiens_2983 | MN908947.3 | 27799 | 29211 | + | ORF8;ORF7b;N | 2 | find_circ | SRR11550045 |
| SARS-CoV-2_circ_Homo_sapiens_2984 | MN908947.3 | 27799 | 29586 | + | ORF10;ORF8;ORF7b;N | 2 | find_circ | SRR11550044 |
| SARS-CoV-2_circ_Homo_sapiens_2985 | MN908947.3 | 27850 | 28462 | - | ORF8;ORF7b;N | 2 | find_circ | SRR11550045 |
| SARS-CoV-2_circ_Homo_sapiens_2986 | MN908947.3 | 27875 | 28418 | - | ORF8;ORF7b;N | 2 | find_circ | SRR11550044 |
| SARS-CoV-2_circ_Homo_sapiens_2988 | MN908947.3 | 27894 | 28895 | - | ORF8;N | 2 | find_circ | SRR11550046 |
| SARS-CoV-2_circ_Homo_sapiens_2989 | MN908947.3 | 27916 | 28320 | + | ORF8;N | 2 | circRNA_finder | SRR11550045 |
| SARS-CoV-2_circ_Homo_sapiens_2989 | MN908947.3 | 27916 | 28320 | + | ORF8;N | 2 | find_circ | SRR11550045 |
| SARS-CoV-2_circ_Homo_sapiens_2991 | MN908947.3 | 27971 | 28602 | - | ORF8;N | 2 | find_circ | SRR11550043 |
| SARS-CoV-2_circ_Homo_sapiens_2998 | MN908947.3 | 28102 | 28472 | - | ORF8;N | 2 | find_circ | SRR11550045 |
| SARS-CoV-2_circ_Homo_sapiens_2999 | MN908947.3 | 28147 | 28462 | - | ORF8;N | 2 | find_circ | SRR11550045 |
| SARS-CoV-2_circ_Homo_sapiens_300 | MN908947.3 | 1599 | 2122 | + | ORF1ab | 2 | find_circ | SRR11550046 |
| SARS-CoV-2_circ_Homo_sapiens_3000 | MN908947.3 | 28147 | 28462 | + | ORF8;N | 2 | circRNA_finder | SRR11550045 |
| SARS-CoV-2_circ_Homo_sapiens_3001 | MN908947.3 | 28147 | 28895 | - | ORF8;N | 2 | find_circ | SRR11550046 |
| SARS-CoV-2_circ_Homo_sapiens_3005 | MN908947.3 | 28154 | 28343 | - | ORF8;N | 2 | find_circ | SRR11550045 |
| SARS-CoV-2_circ_Homo_sapiens_3007 | MN908947.3 | 28249 | 28596 | + | ORF8;N | 2 | find_circ | SRR11550045 |
| SARS-CoV-2_circ_Homo_sapiens_3010 | MN908947.3 | 28262 | 29408 | - | N | 2 | find_circ | SRR11550045 |
| SARS-CoV-2_circ_Homo_sapiens_3012 | MN908947.3 | 28270 | 29159 | - | N | 2 | find_circ | SRR11550046 |
| SARS-CoV-2_circ_Homo_sapiens_3013 | MN908947.3 | 28290 | 28602 | - | N | 2 | find_circ | SRR11550046 |
| SARS-CoV-2_circ_Homo_sapiens_3014 | MN908947.3 | 28301 | 28556 | + | N | 2 | find_circ | SRR11550045 |
| SARS-CoV-2_circ_Homo_sapiens_3014 | MN908947.3 | 28301 | 28556 | + | N | 2 | find_circ | SRR11550044 |
| SARS-CoV-2_circ_Homo_sapiens_3014 | MN908947.3 | 28301 | 28556 | + | N | 2 | circRNA_finder | SRR11550045 |
| SARS-CoV-2_circ_Homo_sapiens_3014 | MN908947.3 | 28301 | 28556 | + | N | 2 | circRNA_finder | SRR11550044 |
| SARS-CoV-2_circ_Homo_sapiens_3015 | MN908947.3 | 28301 | 29057 | + | N | 2 | find_circ | SRR11550046 |
| SARS-CoV-2_circ_Homo_sapiens_3016 | MN908947.3 | 28321 | 28602 | - | N | 2 | find_circ | SRR11550046 |
| SARS-CoV-2_circ_Homo_sapiens_3017 | MN908947.3 | 28321 | 28602 | + | N | 2 | circRNA_finder | SRR11550043 |
| SARS-CoV-2_circ_Homo_sapiens_3018 | MN908947.3 | 28321 | 28609 | - | N | 2 | find_circ | SRR11550043 |
| SARS-CoV-2_circ_Homo_sapiens_3019 | MN908947.3 | 28321 | 28609 | + | N | 2 | circRNA_finder | SRR11550043 |
| SARS-CoV-2_circ_Homo_sapiens_302 | MN908947.3 | 1599 | 12906 | + | ORF1ab | 2 | find_circ | SRR11550045 |
| SARS-CoV-2_circ_Homo_sapiens_3020 | MN908947.3 | 28321 | 28727 | - | N | 2 | find_circ | SRR11550045 |
| SARS-CoV-2_circ_Homo_sapiens_3020 | MN908947.3 | 28321 | 28727 | - | N | 2 | find_circ | SRR11550046 |
| SARS-CoV-2_circ_Homo_sapiens_3021 | MN908947.3 | 28321 | 28886 | - | N | 2 | find_circ | SRR11550045 |
| SARS-CoV-2_circ_Homo_sapiens_3025 | MN908947.3 | 28321 | 29006 | - | N | 2 | find_circ | SRR11550046 |
| SARS-CoV-2_circ_Homo_sapiens_3030 | MN908947.3 | 28321 | 29329 | - | N | 2 | find_circ | SRR11550045 |
| SARS-CoV-2_circ_Homo_sapiens_3030 | MN908947.3 | 28321 | 29329 | - | N | 2 | find_circ | SRR11550044 |
| SARS-CoV-2_circ_Homo_sapiens_3031 | MN908947.3 | 28321 | 29444 | - | N | 2 | find_circ | SRR11550044 |
| SARS-CoV-2_circ_Homo_sapiens_3032 | MN908947.3 | 28321 | 29534 | - | N | 2 | find_circ | SRR11550045 |
| SARS-CoV-2_circ_Homo_sapiens_3035 | MN908947.3 | 28332 | 28895 | - | N | 2 | find_circ | SRR11550046 |
| SARS-CoV-2_circ_Homo_sapiens_3036 | MN908947.3 | 28332 | 29204 | - | N | 2 | find_circ | SRR11550045 |
| SARS-CoV-2_circ_Homo_sapiens_3037 | MN908947.3 | 28332 | 29204 | + | N | 2 | circRNA_finder | SRR11550045 |
| SARS-CoV-2_circ_Homo_sapiens_304 | MN908947.3 | 1646 | 3098 | - | ORF1ab | 2 | find_circ | SRR11550046 |
| SARS-CoV-2_circ_Homo_sapiens_3040 | MN908947.3 | 28338 | 28974 | + | N | 2 | find_circ | SRR11550045 |
| SARS-CoV-2_circ_Homo_sapiens_3040 | MN908947.3 | 28338 | 28974 | + | N | 2 | circRNA_finder | SRR11550045 |
| SARS-CoV-2_circ_Homo_sapiens_3041 | MN908947.3 | 28338 | 28979 | + | N | 2 | find_circ | SRR11550046 |
| SARS-CoV-2_circ_Homo_sapiens_3042 | MN908947.3 | 28338 | 29080 | + | N | 2 | circRNA_finder | SRR11550045 |
| SARS-CoV-2_circ_Homo_sapiens_3044 | MN908947.3 | 28351 | 28974 | + | N | 2 | find_circ | SRR11550045 |
| SARS-CoV-2_circ_Homo_sapiens_3045 | MN908947.3 | 28355 | 29522 | - | N | 2 | find_circ | SRR11550043 |
| SARS-CoV-2_circ_Homo_sapiens_3046 | MN908947.3 | 28355 | 29522 | + | N | 2 | circRNA_finder | SRR11550043 |
| SARS-CoV-2_circ_Homo_sapiens_3048 | MN908947.3 | 28404 | 28669 | + | N | 2 | circRNA_finder | SRR11550043 |
| SARS-CoV-2_circ_Homo_sapiens_3052 | MN908947.3 | 28404 | 28877 | + | N | 2 | find_circ | SRR11550043 |
| SARS-CoV-2_circ_Homo_sapiens_3053 | MN908947.3 | 28404 | 28916 | + | N | 2 | find_circ | SRR11550045 |
| SARS-CoV-2_circ_Homo_sapiens_3054 | MN908947.3 | 28404 | 28974 | + | N | 2 | circRNA_finder | SRR11550046 |
| SARS-CoV-2_circ_Homo_sapiens_3054 | MN908947.3 | 28404 | 28974 | + | N | 2 | find_circ | SRR11550046 |
| SARS-CoV-2_circ_Homo_sapiens_3055 | MN908947.3 | 28404 | 28979 | + | N | 2 | find_circ | SRR11550044 |
| SARS-CoV-2_circ_Homo_sapiens_3056 | MN908947.3 | 28404 | 29008 | + | N | 2 | find_circ | SRR11550045 |
| SARS-CoV-2_circ_Homo_sapiens_3057 | MN908947.3 | 28404 | 29057 | + | N | 2 | find_circ | SRR11550046 |
| SARS-CoV-2_circ_Homo_sapiens_3059 | MN908947.3 | 28404 | 29259 | + | N | 2 | circRNA_finder | SRR11550043 |
| SARS-CoV-2_circ_Homo_sapiens_3059 | MN908947.3 | 28404 | 29259 | + | N | 2 | circRNA_finder | SRR11550046 |
| SARS-CoV-2_circ_Homo_sapiens_3059 | MN908947.3 | 28404 | 29259 | + | N | 2 | find_circ | SRR11550043 |
| SARS-CoV-2_circ_Homo_sapiens_3059 | MN908947.3 | 28404 | 29259 | + | N | 2 | find_circ | SRR11550046 |
| SARS-CoV-2_circ_Homo_sapiens_3060 | MN908947.3 | 28404 | 29262 | + | N | 2 | circRNA_finder | SRR11550045 |
| SARS-CoV-2_circ_Homo_sapiens_3064 | MN908947.3 | 28410 | 28602 | - | N | 2 | find_circ | SRR11550045 |
| SARS-CoV-2_circ_Homo_sapiens_3065 | MN908947.3 | 28410 | 28646 | - | N | 2 | find_circ | SRR11550043 |
| SARS-CoV-2_circ_Homo_sapiens_3066 | MN908947.3 | 28410 | 28646 | + | N | 2 | circRNA_finder | SRR11550044 |
| SARS-CoV-2_circ_Homo_sapiens_3067 | MN908947.3 | 28410 | 28724 | - | N | 2 | find_circ | SRR11550045 |
| SARS-CoV-2_circ_Homo_sapiens_3069 | MN908947.3 | 28410 | 28727 | - | N | 2 | find_circ | SRR11550043 |
| SARS-CoV-2_circ_Homo_sapiens_3069 | MN908947.3 | 28410 | 28727 | - | N | 2 | find_circ | SRR11550044 |
| SARS-CoV-2_circ_Homo_sapiens_3070 | MN908947.3 | 28410 | 28727 | + | N | 2 | circRNA_finder | SRR11550046 |
| SARS-CoV-2_circ_Homo_sapiens_3071 | MN908947.3 | 28410 | 28753 | - | N | 2 | find_circ | SRR11550043 |
| SARS-CoV-2_circ_Homo_sapiens_3072 | MN908947.3 | 28410 | 28757 | - | N | 2 | find_circ | SRR11550044 |
| SARS-CoV-2_circ_Homo_sapiens_3075 | MN908947.3 | 28410 | 28865 | - | N | 2 | find_circ | SRR11550044 |
| SARS-CoV-2_circ_Homo_sapiens_3075 | MN908947.3 | 28410 | 28865 | - | N | 2 | find_circ | SRR11550043 |
| SARS-CoV-2_circ_Homo_sapiens_3078 | MN908947.3 | 28410 | 28904 | - | N | 2 | find_circ | SRR11550043 |
| SARS-CoV-2_circ_Homo_sapiens_3080 | MN908947.3 | 28410 | 28936 | - | N | 2 | find_circ | SRR11550045 |
| SARS-CoV-2_circ_Homo_sapiens_3082 | MN908947.3 | 28410 | 29012 | - | N | 2 | find_circ | SRR11550043 |
| SARS-CoV-2_circ_Homo_sapiens_3082 | MN908947.3 | 28410 | 29012 | - | N | 2 | find_circ | SRR11550044 |
| SARS-CoV-2_circ_Homo_sapiens_3086 | MN908947.3 | 28410 | 29268 | - | N | 2 | find_circ | SRR11550043 |
| SARS-CoV-2_circ_Homo_sapiens_3087 | MN908947.3 | 28410 | 29268 | + | N | 2 | circRNA_finder | SRR11550043 |
| SARS-CoV-2_circ_Homo_sapiens_3088 | MN908947.3 | 28410 | 29329 | - | N | 2 | find_circ | SRR11550046 |
| SARS-CoV-2_circ_Homo_sapiens_3089 | MN908947.3 | 28410 | 29408 | - | N | 2 | find_circ | SRR11550044 |
| SARS-CoV-2_circ_Homo_sapiens_3089 | MN908947.3 | 28410 | 29408 | - | N | 2 | find_circ | SRR11550045 |
| SARS-CoV-2_circ_Homo_sapiens_3090 | MN908947.3 | 28410 | 29450 | - | N | 2 | find_circ | SRR11550045 |
| SARS-CoV-2_circ_Homo_sapiens_3095 | MN908947.3 | 28420 | 28602 | - | N | 2 | find_circ | SRR11550044 |
| SARS-CoV-2_circ_Homo_sapiens_3099 | MN908947.3 | 28420 | 28976 | - | N | 2 | find_circ | SRR11550045 |
| SARS-CoV-2_circ_Homo_sapiens_3100 | MN908947.3 | 28420 | 28976 | + | N | 2 | circRNA_finder | SRR11550045 |
| SARS-CoV-2_circ_Homo_sapiens_3107 | MN908947.3 | 28444 | 28895 | - | N | 2 | find_circ | SRR11550046 |
| SARS-CoV-2_circ_Homo_sapiens_3112 | MN908947.3 | 28449 | 28960 | - | N | 2 | find_circ | SRR11550045 |
| SARS-CoV-2_circ_Homo_sapiens_3113 | MN908947.3 | 28449 | 29024 | - | N | 2 | find_circ | SRR11550044 |
| SARS-CoV-2_circ_Homo_sapiens_3114 | MN908947.3 | 28449 | 29066 | - | N | 2 | find_circ | SRR11550043 |
| SARS-CoV-2_circ_Homo_sapiens_3115 | MN908947.3 | 28457 | 28850 | + | N | 2 | CIRI2 | SRR11550044 |
| SARS-CoV-2_circ_Homo_sapiens_3115 | MN908947.3 | 28457 | 28850 | + | N | 2 | find_circ | SRR11550043 |
| SARS-CoV-2_circ_Homo_sapiens_3116 | MN908947.3 | 28463 | 28676 | - | N | 2 | find_circ | SRR11550044 |
| SARS-CoV-2_circ_Homo_sapiens_3118 | MN908947.3 | 28463 | 28686 | - | N | 2 | find_circ | SRR11550043 |
| SARS-CoV-2_circ_Homo_sapiens_3118 | MN908947.3 | 28463 | 28686 | - | N | 2 | find_circ | SRR11550046 |
| SARS-CoV-2_circ_Homo_sapiens_3119 | MN908947.3 | 28463 | 28724 | - | N | 2 | find_circ | SRR11550045 |
| SARS-CoV-2_circ_Homo_sapiens_3122 | MN908947.3 | 28463 | 28736 | - | N | 2 | CIRI2 | SRR11550045 |
| SARS-CoV-2_circ_Homo_sapiens_3125 | MN908947.3 | 28463 | 28753 | - | N | 2 | find_circ | SRR11550043 |
| SARS-CoV-2_circ_Homo_sapiens_3128 | MN908947.3 | 28463 | 28785 | - | N | 2 | find_circ | SRR11550044 |
| SARS-CoV-2_circ_Homo_sapiens_313 | MN908947.3 | 1844 | 2425 | + | ORF1ab | 2 | circRNA_finder | SRR11550043 |
| SARS-CoV-2_circ_Homo_sapiens_313 | MN908947.3 | 1844 | 2425 | + | ORF1ab | 2 | find_circ | SRR11550043 |
| SARS-CoV-2_circ_Homo_sapiens_3130 | MN908947.3 | 28463 | 28830 | - | N | 2 | find_circ | SRR11550045 |
| SARS-CoV-2_circ_Homo_sapiens_3130 | MN908947.3 | 28463 | 28830 | - | N | 2 | find_circ | SRR11550046 |
| SARS-CoV-2_circ_Homo_sapiens_3131 | MN908947.3 | 28463 | 28830 | + | N | 2 | circRNA_finder | SRR11550046 |
| SARS-CoV-2_circ_Homo_sapiens_3132 | MN908947.3 | 28463 | 28886 | - | N | 2 | CIRI2 | SRR11550044 |
| SARS-CoV-2_circ_Homo_sapiens_3136 | MN908947.3 | 28463 | 28927 | - | N | 2 | find_circ | SRR11550044 |
| SARS-CoV-2_circ_Homo_sapiens_3140 | MN908947.3 | 28463 | 28942 | - | N | 2 | find_circ | SRR11550046 |
| SARS-CoV-2_circ_Homo_sapiens_3141 | MN908947.3 | 28463 | 28942 | + | N | 2 | circRNA_finder | SRR11550046 |
| SARS-CoV-2_circ_Homo_sapiens_3143 | MN908947.3 | 28463 | 28960 | + | N | 2 | circRNA_finder | SRR11550045 |
| SARS-CoV-2_circ_Homo_sapiens_3144 | MN908947.3 | 28463 | 29006 | - | N | 2 | find_circ | SRR11550046 |
| SARS-CoV-2_circ_Homo_sapiens_3145 | MN908947.3 | 28463 | 29006 | + | N | 2 | circRNA_finder | SRR11550046 |
| SARS-CoV-2_circ_Homo_sapiens_3148 | MN908947.3 | 28463 | 29036 | - | N | 2 | find_circ | SRR11550043 |
| SARS-CoV-2_circ_Homo_sapiens_3149 | MN908947.3 | 28463 | 29036 | + | N | 2 | circRNA_finder | SRR11550043 |
| SARS-CoV-2_circ_Homo_sapiens_3150 | MN908947.3 | 28463 | 29066 | - | N | 2 | find_circ | SRR11550046 |
| SARS-CoV-2_circ_Homo_sapiens_3151 | MN908947.3 | 28463 | 29066 | + | N | 2 | circRNA_finder | SRR11550046 |
| SARS-CoV-2_circ_Homo_sapiens_3157 | MN908947.3 | 28463 | 29268 | - | N | 2 | CIRI2 | SRR11550044 |
| SARS-CoV-2_circ_Homo_sapiens_3160 | MN908947.3 | 28463 | 29444 | - | N | 2 | find_circ | SRR11550044 |
| SARS-CoV-2_circ_Homo_sapiens_3160 | MN908947.3 | 28463 | 29444 | - | N | 2 | CIRI2 | SRR11550043 |
| SARS-CoV-2_circ_Homo_sapiens_3163 | MN908947.3 | 28463 | 29579 | - | ORF10;N | 2 | find_circ | SRR11550043 |
| SARS-CoV-2_circ_Homo_sapiens_3168 | MN908947.3 | 28463 | 29678 | - | ORF10;N | 2 | find_circ | SRR11550046 |
| SARS-CoV-2_circ_Homo_sapiens_3169 | MN908947.3 | 28463 | 29678 | + | ORF10;N | 2 | circRNA_finder | SRR11550046 |
| SARS-CoV-2_circ_Homo_sapiens_317 | MN908947.3 | 1953 | 2459 | + | ORF1ab | 2 | circRNA_finder | SRR11550046 |
| SARS-CoV-2_circ_Homo_sapiens_3170 | MN908947.3 | 28463 | 29802 | - | ORF10;N | 2 | find_circ | SRR11550043 |
| SARS-CoV-2_circ_Homo_sapiens_3172 | MN908947.3 | 28499 | 28818 | - | N | 2 | find_circ | SRR11550044 |
| SARS-CoV-2_circ_Homo_sapiens_3173 | MN908947.3 | 28499 | 28818 | + | N | 2 | circRNA_finder | SRR11550044 |
| SARS-CoV-2_circ_Homo_sapiens_3174 | MN908947.3 | 28515 | 28850 | + | N | 2 | find_circ | SRR11550046 |
| SARS-CoV-2_circ_Homo_sapiens_3178 | MN908947.3 | 28532 | 28904 | - | N | 2 | find_circ | SRR11550046 |
| SARS-CoV-2_circ_Homo_sapiens_3179 | MN908947.3 | 28535 | 28904 | + | N | 2 | circRNA_finder | SRR11550043 |
| SARS-CoV-2_circ_Homo_sapiens_3181 | MN908947.3 | 28542 | 29080 | + | N | 2 | circRNA_finder | SRR11550043 |
| SARS-CoV-2_circ_Homo_sapiens_3181 | MN908947.3 | 28542 | 29080 | + | N | 2 | find_circ | SRR11550043 |
| SARS-CoV-2_circ_Homo_sapiens_3184 | MN908947.3 | 28551 | 29066 | - | N | 2 | find_circ | SRR11550046 |
| SARS-CoV-2_circ_Homo_sapiens_3185 | MN908947.3 | 28594 | 28979 | + | N | 2 | find_circ | SRR11550046 |
| SARS-CoV-2_circ_Homo_sapiens_3195 | MN908947.3 | 28607 | 28976 | - | N | 2 | find_circ | SRR11550043 |
| SARS-CoV-2_circ_Homo_sapiens_3196 | MN908947.3 | 28607 | 28976 | + | N | 2 | circRNA_finder | SRR11550043 |
| SARS-CoV-2_circ_Homo_sapiens_320 | MN908947.3 | 1958 | 2421 | + | ORF1ab | 2 | find_circ | SRR11550046 |
| SARS-CoV-2_circ_Homo_sapiens_3209 | MN908947.3 | 28610 | 28925 | - | N | 2 | find_circ | SRR11550043 |
| SARS-CoV-2_circ_Homo_sapiens_3218 | MN908947.3 | 28614 | 29008 | + | N | 2 | CIRI2 | SRR11550046 |
| SARS-CoV-2_circ_Homo_sapiens_3218 | MN908947.3 | 28614 | 29008 | + | N | 2 | find_circ | SRR11550046 |
| SARS-CoV-2_circ_Homo_sapiens_3224 | MN908947.3 | 28652 | 29036 | - | N | 2 | find_circ | SRR11550045 |
| SARS-CoV-2_circ_Homo_sapiens_3224 | MN908947.3 | 28652 | 29036 | - | N | 2 | find_circ | SRR11550046 |
| SARS-CoV-2_circ_Homo_sapiens_3226 | MN908947.3 | 28652 | 29143 | - | N | 2 | find_circ | SRR11550044 |
| SARS-CoV-2_circ_Homo_sapiens_3227 | MN908947.3 | 28652 | 29268 | - | N | 2 | find_circ | SRR11550044 |
| SARS-CoV-2_circ_Homo_sapiens_3229 | MN908947.3 | 28652 | 29562 | - | ORF10;N | 2 | find_circ | SRR11550044 |
| SARS-CoV-2_circ_Homo_sapiens_3230 | MN908947.3 | 28656 | 28979 | + | N | 2 | CIRI2 | SRR11550043 |
| SARS-CoV-2_circ_Homo_sapiens_3234 | MN908947.3 | 28658 | 28892 | - | N | 2 | find_circ | SRR11550046 |
| SARS-CoV-2_circ_Homo_sapiens_3239 | MN908947.3 | 28696 | 28976 | - | N | 2 | find_circ | SRR11550045 |
| SARS-CoV-2_circ_Homo_sapiens_3244 | MN908947.3 | 28698 | 29066 | - | N | 2 | find_circ | SRR11550046 |
| SARS-CoV-2_circ_Homo_sapiens_3245 | MN908947.3 | 28698 | 29066 | + | N | 2 | circRNA_finder | SRR11550046 |
| SARS-CoV-2_circ_Homo_sapiens_3247 | MN908947.3 | 28704 | 29262 | + | N | 2 | find_circ | SRR11550044 |
| SARS-CoV-2_circ_Homo_sapiens_3249 | MN908947.3 | 28709 | 29012 | - | N | 2 | find_circ | SRR11550043 |
| SARS-CoV-2_circ_Homo_sapiens_3259 | MN908947.3 | 28733 | 29024 | - | N | 2 | find_circ | SRR11550046 |
| SARS-CoV-2_circ_Homo_sapiens_3261 | MN908947.3 | 28733 | 29268 | + | N | 2 | circRNA_finder | SRR11550043 |
| SARS-CoV-2_circ_Homo_sapiens_3262 | MN908947.3 | 28733 | 29450 | - | N | 2 | find_circ | SRR11550044 |
| SARS-CoV-2_circ_Homo_sapiens_3263 | MN908947.3 | 28733 | 29534 | - | N | 2 | find_circ | SRR11550046 |
| SARS-CoV-2_circ_Homo_sapiens_3269 | MN908947.3 | 28771 | 29066 | - | N | 2 | find_circ | SRR11550046 |
| SARS-CoV-2_circ_Homo_sapiens_3271 | MN908947.3 | 28771 | 29159 | - | N | 2 | find_circ | SRR11550046 |
| SARS-CoV-2_circ_Homo_sapiens_3275 | MN908947.3 | 28790 | 29066 | - | N | 2 | find_circ | SRR11550043 |
| SARS-CoV-2_circ_Homo_sapiens_3275 | MN908947.3 | 28790 | 29066 | - | N | 2 | find_circ | SRR11550045 |
| SARS-CoV-2_circ_Homo_sapiens_3286 | MN908947.3 | 28951 | 29756 | + | ORF10;N | 2 | find_circ | SRR11550044 |
| SARS-CoV-2_circ_Homo_sapiens_3288 | MN908947.3 | 28961 | 29259 | + | N | 2 | find_circ | SRR11550045 |
| SARS-CoV-2_circ_Homo_sapiens_3288 | MN908947.3 | 28961 | 29259 | + | N | 2 | find_circ | SRR11550043 |
| SARS-CoV-2_circ_Homo_sapiens_3289 | MN908947.3 | 28961 | 29262 | + | N | 2 | circRNA_finder | SRR11550046 |
| SARS-CoV-2_circ_Homo_sapiens_3289 | MN908947.3 | 28961 | 29262 | + | N | 2 | find_circ | SRR11550046 |
| SARS-CoV-2_circ_Homo_sapiens_3290 | MN908947.3 | 28961 | 29572 | + | ORF10;N | 2 | circRNA_finder | SRR11550045 |
| SARS-CoV-2_circ_Homo_sapiens_3290 | MN908947.3 | 28961 | 29572 | + | ORF10;N | 2 | find_circ | SRR11550045 |
| SARS-CoV-2_circ_Homo_sapiens_3291 | MN908947.3 | 28967 | 29211 | + | N | 2 | find_circ | SRR11550046 |
| SARS-CoV-2_circ_Homo_sapiens_3293 | MN908947.3 | 28967 | 29572 | + | ORF10;N | 2 | find_circ | SRR11550044 |
| SARS-CoV-2_circ_Homo_sapiens_3295 | MN908947.3 | 29018 | 29211 | + | N | 2 | find_circ | SRR11550043 |
| SARS-CoV-2_circ_Homo_sapiens_3295 | MN908947.3 | 29018 | 29211 | + | N | 2 | find_circ | SRR11550044 |
| SARS-CoV-2_circ_Homo_sapiens_3297 | MN908947.3 | 29068 | 29444 | - | N | 2 | find_circ | SRR11550046 |
| SARS-CoV-2_circ_Homo_sapiens_3297 | MN908947.3 | 29068 | 29444 | - | N | 2 | find_circ | SRR11550044 |
| SARS-CoV-2_circ_Homo_sapiens_3298 | MN908947.3 | 29073 | 29242 | + | N | 2 | find_circ | SRR11550043 |
| SARS-CoV-2_circ_Homo_sapiens_3299 | MN908947.3 | 29073 | 29446 | + | N | 2 | find_circ | SRR11550043 |
| SARS-CoV-2_circ_Homo_sapiens_3299 | MN908947.3 | 29073 | 29446 | + | N | 2 | find_circ | SRR11550044 |
| SARS-CoV-2_circ_Homo_sapiens_330 | MN908947.3 | 2163 | 2351 | + | ORF1ab | 2 | find_circ | SRR11550045 |
| SARS-CoV-2_circ_Homo_sapiens_3302 | MN908947.3 | 29078 | 29329 | - | N | 2 | find_circ | SRR11550045 |
| SARS-CoV-2_circ_Homo_sapiens_3303 | MN908947.3 | 29078 | 29459 | - | N | 2 | find_circ | SRR11550044 |
| SARS-CoV-2_circ_Homo_sapiens_3304 | MN908947.3 | 29078 | 29459 | + | N | 2 | circRNA_finder | SRR11550044 |
| SARS-CoV-2_circ_Homo_sapiens_3305 | MN908947.3 | 29078 | 29481 | - | N | 2 | find_circ | SRR11550045 |
| SARS-CoV-2_circ_Homo_sapiens_3310 | MN908947.3 | 29086 | 29408 | - | N | 2 | find_circ | SRR11550045 |
| SARS-CoV-2_circ_Homo_sapiens_3314 | MN908947.3 | 29086 | 29522 | - | N | 2 | find_circ | SRR11550046 |
| SARS-CoV-2_circ_Homo_sapiens_3315 | MN908947.3 | 29086 | 29522 | + | N | 2 | circRNA_finder | SRR11550046 |
| SARS-CoV-2_circ_Homo_sapiens_3316 | MN908947.3 | 29086 | 29529 | - | N | 2 | find_circ | SRR11550045 |
| SARS-CoV-2_circ_Homo_sapiens_3319 | MN908947.3 | 29086 | 29579 | - | ORF10;N | 2 | find_circ | SRR11550044 |
| SARS-CoV-2_circ_Homo_sapiens_332 | MN908947.3 | 2282 | 5122 | + | ORF1ab | 2 | circRNA_finder | SRR11550043 |
| SARS-CoV-2_circ_Homo_sapiens_332 | MN908947.3 | 2282 | 5122 | + | ORF1ab | 2 | find_circ | SRR11550043 |
| SARS-CoV-2_circ_Homo_sapiens_3325 | MN908947.3 | 29091 | 29446 | + | N | 2 | find_circ | SRR11550044 |
| SARS-CoV-2_circ_Homo_sapiens_3326 | MN908947.3 | 29091 | 29572 | + | ORF10;N | 2 | circRNA_finder | SRR11550045 |
| SARS-CoV-2_circ_Homo_sapiens_3326 | MN908947.3 | 29091 | 29572 | + | ORF10;N | 2 | find_circ | SRR11550046 |
| SARS-CoV-2_circ_Homo_sapiens_3326 | MN908947.3 | 29091 | 29572 | + | ORF10;N | 2 | find_circ | SRR11550045 |
| SARS-CoV-2_circ_Homo_sapiens_3328 | MN908947.3 | 29103 | 29415 | - | N | 2 | find_circ | SRR11550046 |
| SARS-CoV-2_circ_Homo_sapiens_3329 | MN908947.3 | 29103 | 29444 | - | N | 2 | find_circ | SRR11550046 |
| SARS-CoV-2_circ_Homo_sapiens_3334 | MN908947.3 | 29152 | 29262 | + | N | 2 | find_circ | SRR11550045 |
| SARS-CoV-2_circ_Homo_sapiens_3335 | MN908947.3 | 29157 | 29586 | + | ORF10;N | 2 | find_circ | SRR11550045 |
| SARS-CoV-2_circ_Homo_sapiens_3335 | MN908947.3 | 29157 | 29586 | + | ORF10;N | 2 | circRNA_finder | SRR11550045 |
| SARS-CoV-2_circ_Homo_sapiens_3336 | MN908947.3 | 29168 | 29450 | - | N | 2 | find_circ | SRR11550046 |
| SARS-CoV-2_circ_Homo_sapiens_3339 | MN908947.3 | 29168 | 29534 | - | N | 2 | find_circ | SRR11550046 |
| SARS-CoV-2_circ_Homo_sapiens_334 | MN908947.3 | 2389 | 27051 | - | M;E;ORF3a;S;ORF1ab | 2 | find_circ | SRR11550045 |
| SARS-CoV-2_circ_Homo_sapiens_3340 | MN908947.3 | 29168 | 29534 | + | N | 2 | circRNA_finder | SRR11550046 |
| SARS-CoV-2_circ_Homo_sapiens_3345 | MN908947.3 | 29172 | 29455 | - | N | 2 | find_circ | SRR11550045 |
| SARS-CoV-2_circ_Homo_sapiens_3346 | MN908947.3 | 29172 | 29455 | + | N | 2 | circRNA_finder | SRR11550045 |
| SARS-CoV-2_circ_Homo_sapiens_3347 | MN908947.3 | 29172 | 29517 | - | N | 2 | find_circ | SRR11550045 |
| SARS-CoV-2_circ_Homo_sapiens_3348 | MN908947.3 | 29172 | 29529 | - | N | 2 | find_circ | SRR11550043 |
| SARS-CoV-2_circ_Homo_sapiens_335 | MN908947.3 | 2389 | 27051 | + | M;E;ORF3a;S;ORF1ab | 2 | circRNA_finder | SRR11550045 |
| SARS-CoV-2_circ_Homo_sapiens_3351 | MN908947.3 | 29172 | 29579 | + | ORF10;N | 2 | circRNA_finder | SRR11550044 |
| SARS-CoV-2_circ_Homo_sapiens_3352 | MN908947.3 | 29172 | 29607 | - | ORF10;N | 2 | find_circ | SRR11550045 |
| SARS-CoV-2_circ_Homo_sapiens_3353 | MN908947.3 | 29172 | 29607 | + | ORF10;N | 2 | circRNA_finder | SRR11550045 |
| SARS-CoV-2_circ_Homo_sapiens_3355 | MN908947.3 | 29172 | 29658 | - | ORF10;N | 2 | find_circ | SRR11550046 |
| SARS-CoV-2_circ_Homo_sapiens_3355 | MN908947.3 | 29172 | 29658 | - | ORF10;N | 2 | find_circ | SRR11550044 |
| SARS-CoV-2_circ_Homo_sapiens_3356 | MN908947.3 | 29172 | 29658 | + | ORF10;N | 2 | circRNA_finder | SRR11550044 |
| SARS-CoV-2_circ_Homo_sapiens_3357 | MN908947.3 | 29172 | 29678 | + | ORF10;N | 2 | circRNA_finder | SRR11550044 |
| SARS-CoV-2_circ_Homo_sapiens_3358 | MN908947.3 | 29172 | 29707 | - | ORF10;N | 2 | find_circ | SRR11550043 |
| SARS-CoV-2_circ_Homo_sapiens_3358 | MN908947.3 | 29172 | 29707 | - | ORF10;N | 2 | find_circ | SRR11550044 |
| SARS-CoV-2_circ_Homo_sapiens_3358 | MN908947.3 | 29172 | 29707 | - | ORF10;N | 2 | find_circ | SRR11550046 |
| SARS-CoV-2_circ_Homo_sapiens_3359 | MN908947.3 | 29172 | 29707 | + | ORF10;N | 2 | circRNA_finder | SRR11550044 |
| SARS-CoV-2_circ_Homo_sapiens_3359 | MN908947.3 | 29172 | 29707 | + | ORF10;N | 2 | circRNA_finder | SRR11550043 |
| SARS-CoV-2_circ_Homo_sapiens_3360 | MN908947.3 | 29190 | 29481 | - | N | 2 | find_circ | SRR11550046 |
| SARS-CoV-2_circ_Homo_sapiens_3361 | MN908947.3 | 29190 | 29481 | + | N | 2 | circRNA_finder | SRR11550046 |
| SARS-CoV-2_circ_Homo_sapiens_3364 | MN908947.3 | 29190 | 29678 | - | ORF10;N | 2 | find_circ | SRR11550043 |
| SARS-CoV-2_circ_Homo_sapiens_3364 | MN908947.3 | 29190 | 29678 | - | ORF10;N | 2 | find_circ | SRR11550046 |
| SARS-CoV-2_circ_Homo_sapiens_3364 | MN908947.3 | 29190 | 29678 | - | ORF10;N | 2 | find_circ | SRR11550045 |
| SARS-CoV-2_circ_Homo_sapiens_3366 | MN908947.3 | 29211 | 29644 | + | ORF10;N | 2 | find_circ | SRR11550044 |
| SARS-CoV-2_circ_Homo_sapiens_3369 | MN908947.3 | 29250 | 29534 | - | N | 2 | find_circ | SRR11550046 |
| SARS-CoV-2_circ_Homo_sapiens_3370 | MN908947.3 | 29250 | 29534 | + | N | 2 | circRNA_finder | SRR11550046 |
| SARS-CoV-2_circ_Homo_sapiens_3372 | MN908947.3 | 29250 | 29579 | - | ORF10;N | 2 | CIRI2 | SRR11550044 |
| SARS-CoV-2_circ_Homo_sapiens_3379 | MN908947.3 | 29250 | 29773 | - | ORF10;N | 2 | find_circ | SRR11550045 |
| SARS-CoV-2_circ_Homo_sapiens_3380 | MN908947.3 | 29250 | 29773 | + | ORF10;N | 2 | circRNA_finder | SRR11550045 |
| SARS-CoV-2_circ_Homo_sapiens_3381 | MN908947.3 | 29260 | 29444 | - | N | 2 | find_circ | SRR11550046 |
| SARS-CoV-2_circ_Homo_sapiens_3383 | MN908947.3 | 29260 | 29579 | - | ORF10;N | 2 | find_circ | SRR11550044 |
| SARS-CoV-2_circ_Homo_sapiens_3384 | MN908947.3 | 29260 | 29607 | - | ORF10;N | 2 | find_circ | SRR11550043 |
| SARS-CoV-2_circ_Homo_sapiens_3386 | MN908947.3 | 29260 | 29678 | - | ORF10;N | 2 | find_circ | SRR11550043 |
| SARS-CoV-2_circ_Homo_sapiens_3387 | MN908947.3 | 29269 | 29444 | - | N | 2 | find_circ | SRR11550044 |
| SARS-CoV-2_circ_Homo_sapiens_3389 | MN908947.3 | 29269 | 29455 | - | N | 2 | CIRI2 | SRR11550046 |
| SARS-CoV-2_circ_Homo_sapiens_339 | MN908947.3 | 2493 | 3029 | + | ORF1ab | 2 | find_circ | SRR11550043 |
| SARS-CoV-2_circ_Homo_sapiens_3390 | MN908947.3 | 29269 | 29513 | - | N | 2 | find_circ | SRR11550044 |
| SARS-CoV-2_circ_Homo_sapiens_3390 | MN908947.3 | 29269 | 29513 | - | N | 2 | find_circ | SRR11550045 |
| SARS-CoV-2_circ_Homo_sapiens_3393 | MN908947.3 | 29269 | 29529 | - | N | 2 | find_circ | SRR11550045 |
| SARS-CoV-2_circ_Homo_sapiens_3394 | MN908947.3 | 29269 | 29529 | + | N | 2 | circRNA_finder | SRR11550045 |
| SARS-CoV-2_circ_Homo_sapiens_3395 | MN908947.3 | 29269 | 29534 | - | N | 2 | find_circ | SRR11550044 |
| SARS-CoV-2_circ_Homo_sapiens_3397 | MN908947.3 | 29269 | 29562 | - | ORF10;N | 2 | find_circ | SRR11550046 |
| SARS-CoV-2_circ_Homo_sapiens_3399 | MN908947.3 | 29269 | 29579 | - | ORF10;N | 2 | find_circ | SRR11550043 |
| SARS-CoV-2_circ_Homo_sapiens_34 | MN908947.3 | 8 | 29572 | + | ORF6;ORF10;M;E;ORF3a;S;N;ORF8;ORF7a;ORF7b;ORF1ab | 2 | find_circ | SRR11550044 |
| SARS-CoV-2_circ_Homo_sapiens_340 | MN908947.3 | 2493 | 3350 | + | ORF1ab | 2 | circRNA_finder | SRR11550044 |
| SARS-CoV-2_circ_Homo_sapiens_340 | MN908947.3 | 2493 | 3350 | + | ORF1ab | 2 | find_circ | SRR11550044 |
| SARS-CoV-2_circ_Homo_sapiens_3406 | MN908947.3 | 29273 | 29481 | - | N | 2 | find_circ | SRR11550046 |
| SARS-CoV-2_circ_Homo_sapiens_3409 | MN908947.3 | 29273 | 29787 | - | ORF10;N | 2 | find_circ | SRR11550044 |
| SARS-CoV-2_circ_Homo_sapiens_3413 | MN908947.3 | 29316 | 29758 | + | ORF10;N | 2 | circRNA_finder | SRR11550046 |
| SARS-CoV-2_circ_Homo_sapiens_3413 | MN908947.3 | 29316 | 29758 | + | ORF10;N | 2 | find_circ | SRR11550046 |
| SARS-CoV-2_circ_Homo_sapiens_3416 | MN908947.3 | 29322 | 29758 | + | ORF10;N | 2 | find_circ | SRR11550043 |
| SARS-CoV-2_circ_Homo_sapiens_3418 | MN908947.3 | 29339 | 29653 | + | ORF10;N | 2 | find_circ | SRR11550046 |
| SARS-CoV-2_circ_Homo_sapiens_3420 | MN908947.3 | 29348 | 29529 | - | N | 2 | find_circ | SRR11550044 |
| SARS-CoV-2_circ_Homo_sapiens_3422 | MN908947.3 | 29348 | 29624 | - | ORF10;N | 2 | find_circ | SRR11550043 |
| SARS-CoV-2_circ_Homo_sapiens_3424 | MN908947.3 | 29348 | 29665 | + | ORF10;N | 2 | circRNA_finder | SRR11550046 |
| SARS-CoV-2_circ_Homo_sapiens_3430 | MN908947.3 | 29426 | 29840 | + | ORF10;N | 2 | circRNA_finder | SRR11550044 |
| SARS-CoV-2_circ_Homo_sapiens_3432 | MN908947.3 | 29469 | 29572 | + | ORF10;N | 2 | find_circ | SRR11550043 |
| SARS-CoV-2_circ_Homo_sapiens_3434 | MN908947.3 | 29469 | 29809 | + | ORF10;N | 2 | find_circ | SRR11550043 |
| SARS-CoV-2_circ_Homo_sapiens_3435 | MN908947.3 | 29636 | 29856 | - | ORF10 | 2 | find_circ | SRR11550045 |
| SARS-CoV-2_circ_Homo_sapiens_3437 | MN908947.3 | 29709 | 29787 | - | . | 2 | find_circ | SRR11550043 |
| SARS-CoV-2_circ_Homo_sapiens_346 | MN908947.3 | 2495 | 3337 | + | ORF1ab | 2 | find_circ | SRR11550045 |
| SARS-CoV-2_circ_Homo_sapiens_35 | MN908947.3 | 8 | 29756 | + | ORF6;ORF10;M;E;ORF3a;S;N;ORF8;ORF7a;ORF7b;ORF1ab | 2 | find_circ | SRR11550044 |
| SARS-CoV-2_circ_Homo_sapiens_350 | MN908947.3 | 2524 | 4814 | - | ORF1ab | 2 | find_circ | SRR11550045 |
| SARS-CoV-2_circ_Homo_sapiens_352 | MN908947.3 | 2559 | 3457 | - | ORF1ab | 2 | find_circ | SRR11550046 |
| SARS-CoV-2_circ_Homo_sapiens_353 | MN908947.3 | 2559 | 3665 | - | ORF1ab | 2 | find_circ | SRR11550046 |
| SARS-CoV-2_circ_Homo_sapiens_358 | MN908947.3 | 2565 | 3029 | + | ORF1ab | 2 | find_circ | SRR11550045 |
| SARS-CoV-2_circ_Homo_sapiens_361 | MN908947.3 | 2571 | 3176 | - | ORF1ab | 2 | find_circ | SRR11550046 |
| SARS-CoV-2_circ_Homo_sapiens_362 | MN908947.3 | 2571 | 3176 | + | ORF1ab | 2 | circRNA_finder | SRR11550046 |
| SARS-CoV-2_circ_Homo_sapiens_363 | MN908947.3 | 2571 | 3263 | - | ORF1ab | 2 | find_circ | SRR11550043 |
| SARS-CoV-2_circ_Homo_sapiens_364 | MN908947.3 | 2571 | 3263 | + | ORF1ab | 2 | circRNA_finder | SRR11550043 |
| SARS-CoV-2_circ_Homo_sapiens_368 | MN908947.3 | 2571 | 3539 | - | ORF1ab | 2 | find_circ | SRR11550046 |
| SARS-CoV-2_circ_Homo_sapiens_368 | MN908947.3 | 2571 | 3539 | - | ORF1ab | 2 | find_circ | SRR11550044 |
| SARS-CoV-2_circ_Homo_sapiens_369 | MN908947.3 | 2571 | 3539 | + | ORF1ab | 2 | circRNA_finder | SRR11550044 |
| SARS-CoV-2_circ_Homo_sapiens_372 | MN908947.3 | 2571 | 16288 | - | ORF1ab | 2 | find_circ | SRR11550046 |
| SARS-CoV-2_circ_Homo_sapiens_374 | MN908947.3 | 2578 | 3024 | + | ORF1ab | 2 | find_circ | SRR11550045 |
| SARS-CoV-2_circ_Homo_sapiens_374 | MN908947.3 | 2578 | 3024 | + | ORF1ab | 2 | circRNA_finder | SRR11550045 |
| SARS-CoV-2_circ_Homo_sapiens_375 | MN908947.3 | 2578 | 3350 | + | ORF1ab | 2 | find_circ | SRR11550045 |
| SARS-CoV-2_circ_Homo_sapiens_375 | MN908947.3 | 2578 | 3350 | + | ORF1ab | 2 | circRNA_finder | SRR11550045 |
| SARS-CoV-2_circ_Homo_sapiens_378 | MN908947.3 | 2592 | 3071 | + | ORF1ab | 2 | find_circ | SRR11550045 |
| SARS-CoV-2_circ_Homo_sapiens_383 | MN908947.3 | 2676 | 3329 | - | ORF1ab | 2 | CIRI2 | SRR11550044 |
| SARS-CoV-2_circ_Homo_sapiens_383 | MN908947.3 | 2676 | 3329 | - | ORF1ab | 2 | find_circ | SRR11550044 |
| SARS-CoV-2_circ_Homo_sapiens_384 | MN908947.3 | 2676 | 3329 | + | ORF1ab | 2 | circRNA_finder | SRR11550044 |
| SARS-CoV-2_circ_Homo_sapiens_385 | MN908947.3 | 2676 | 3542 | - | ORF1ab | 2 | find_circ | SRR11550045 |
| SARS-CoV-2_circ_Homo_sapiens_386 | MN908947.3 | 2676 | 3542 | + | ORF1ab | 2 | circRNA_finder | SRR11550045 |
| SARS-CoV-2_circ_Homo_sapiens_388 | MN908947.3 | 2715 | 2920 | + | ORF1ab | 2 | find_circ | SRR11550045 |
| SARS-CoV-2_circ_Homo_sapiens_39 | MN908947.3 | 16 | 526 | - | ORF1ab | 2 | find_circ | SRR11550043 |
| SARS-CoV-2_circ_Homo_sapiens_394 | MN908947.3 | 2791 | 3329 | - | ORF1ab | 2 | find_circ | SRR11550045 |
| SARS-CoV-2_circ_Homo_sapiens_394 | MN908947.3 | 2791 | 3329 | - | ORF1ab | 2 | find_circ | SRR11550046 |
| SARS-CoV-2_circ_Homo_sapiens_396 | MN908947.3 | 2799 | 3329 | - | ORF1ab | 2 | find_circ | SRR11550045 |
| SARS-CoV-2_circ_Homo_sapiens_398 | MN908947.3 | 2799 | 3737 | - | ORF1ab | 2 | find_circ | SRR11550043 |
| SARS-CoV-2_circ_Homo_sapiens_399 | MN908947.3 | 2799 | 5906 | - | ORF1ab | 2 | find_circ | SRR11550045 |
| SARS-CoV-2_circ_Homo_sapiens_40 | MN908947.3 | 16 | 590 | - | ORF1ab | 2 | find_circ | SRR11550045 |
| SARS-CoV-2_circ_Homo_sapiens_405 | MN908947.3 | 2865 | 3337 | + | ORF1ab | 2 | find_circ | SRR11550046 |
| SARS-CoV-2_circ_Homo_sapiens_406 | MN908947.3 | 2911 | 3329 | - | ORF1ab | 2 | find_circ | SRR11550045 |
| SARS-CoV-2_circ_Homo_sapiens_406 | MN908947.3 | 2911 | 3329 | - | ORF1ab | 2 | find_circ | SRR11550046 |
| SARS-CoV-2_circ_Homo_sapiens_41 | MN908947.3 | 16 | 1925 | - | ORF1ab | 2 | CIRI2 | SRR11550045 |
| SARS-CoV-2_circ_Homo_sapiens_410 | MN908947.3 | 2934 | 3457 | - | ORF1ab | 2 | find_circ | SRR11550046 |
| SARS-CoV-2_circ_Homo_sapiens_412 | MN908947.3 | 2958 | 3337 | + | ORF1ab | 2 | find_circ | SRR11550045 |
| SARS-CoV-2_circ_Homo_sapiens_414 | MN908947.3 | 3021 | 3542 | - | ORF1ab | 2 | find_circ | SRR11550045 |
| SARS-CoV-2_circ_Homo_sapiens_415 | MN908947.3 | 3021 | 3542 | + | ORF1ab | 2 | circRNA_finder | SRR11550045 |
| SARS-CoV-2_circ_Homo_sapiens_427 | MN908947.3 | 3177 | 3500 | - | ORF1ab | 2 | find_circ | SRR11550045 |
| SARS-CoV-2_circ_Homo_sapiens_428 | MN908947.3 | 3177 | 3542 | - | ORF1ab | 2 | find_circ | SRR11550045 |
| SARS-CoV-2_circ_Homo_sapiens_429 | MN908947.3 | 3177 | 3542 | + | ORF1ab | 2 | circRNA_finder | SRR11550045 |
| SARS-CoV-2_circ_Homo_sapiens_432 | MN908947.3 | 3177 | 4831 | - | ORF1ab | 2 | find_circ | SRR11550044 |
| SARS-CoV-2_circ_Homo_sapiens_433 | MN908947.3 | 3177 | 4831 | + | ORF1ab | 2 | circRNA_finder | SRR11550044 |
| SARS-CoV-2_circ_Homo_sapiens_436 | MN908947.3 | 3177 | 22376 | - | S;ORF1ab | 2 | find_circ | SRR11550046 |
| SARS-CoV-2_circ_Homo_sapiens_439 | MN908947.3 | 3294 | 4523 | - | ORF1ab | 2 | find_circ | SRR11550044 |
| SARS-CoV-2_circ_Homo_sapiens_44 | MN908947.3 | 16 | 5564 | - | ORF1ab | 2 | find_circ | SRR11550044 |
| SARS-CoV-2_circ_Homo_sapiens_443 | MN908947.3 | 3346 | 5624 | + | ORF1ab | 2 | find_circ | SRR11550043 |
| SARS-CoV-2_circ_Homo_sapiens_445 | MN908947.3 | 3383 | 3665 | - | ORF1ab | 2 | find_circ | SRR11550043 |
| SARS-CoV-2_circ_Homo_sapiens_446 | MN908947.3 | 3383 | 3665 | + | ORF1ab | 2 | circRNA_finder | SRR11550043 |
| SARS-CoV-2_circ_Homo_sapiens_448 | MN908947.3 | 3383 | 17110 | - | ORF1ab | 2 | find_circ | SRR11550045 |
| SARS-CoV-2_circ_Homo_sapiens_449 | MN908947.3 | 3383 | 28462 | - | ORF6;M;E;ORF3a;S;N;ORF8;ORF7a;ORF7b;ORF1ab | 2 | find_circ | SRR11550044 |
| SARS-CoV-2_circ_Homo_sapiens_450 | MN908947.3 | 3383 | 28462 | + | ORF6;M;E;ORF3a;S;N;ORF8;ORF7a;ORF7b;ORF1ab | 2 | circRNA_finder | SRR11550044 |
| SARS-CoV-2_circ_Homo_sapiens_457 | MN908947.3 | 3540 | 4520 | + | ORF1ab | 2 | find_circ | SRR11550046 |
| SARS-CoV-2_circ_Homo_sapiens_457 | MN908947.3 | 3540 | 4520 | + | ORF1ab | 2 | find_circ | SRR11550044 |
| SARS-CoV-2_circ_Homo_sapiens_459 | MN908947.3 | 3540 | 26538 | + | M;E;ORF3a;S;ORF1ab | 2 | find_circ | SRR11550045 |
| SARS-CoV-2_circ_Homo_sapiens_459 | MN908947.3 | 3540 | 26538 | + | M;E;ORF3a;S;ORF1ab | 2 | circRNA_finder | SRR11550045 |
| SARS-CoV-2_circ_Homo_sapiens_46 | MN908947.3 | 16 | 6632 | - | ORF1ab | 2 | find_circ | SRR11550044 |
| SARS-CoV-2_circ_Homo_sapiens_464 | MN908947.3 | 3552 | 5525 | - | ORF1ab | 2 | find_circ | SRR11550045 |
| SARS-CoV-2_circ_Homo_sapiens_465 | MN908947.3 | 3552 | 5783 | - | ORF1ab | 2 | find_circ | SRR11550046 |
| SARS-CoV-2_circ_Homo_sapiens_467 | MN908947.3 | 3552 | 29665 | - | ORF6;ORF10;M;E;ORF3a;S;N;ORF8;ORF7a;ORF7b;ORF1ab | 2 | find_circ | SRR11550043 |
| SARS-CoV-2_circ_Homo_sapiens_468 | MN908947.3 | 3555 | 4193 | - | ORF1ab | 2 | find_circ | SRR11550045 |
| SARS-CoV-2_circ_Homo_sapiens_473 | MN908947.3 | 3555 | 4585 | + | ORF1ab | 2 | circRNA_finder | SRR11550044 |
| SARS-CoV-2_circ_Homo_sapiens_473 | MN908947.3 | 3555 | 4585 | + | ORF1ab | 2 | circRNA_finder | SRR11550046 |
| SARS-CoV-2_circ_Homo_sapiens_475 | MN908947.3 | 3555 | 5183 | - | ORF1ab | 2 | find_circ | SRR11550045 |
| SARS-CoV-2_circ_Homo_sapiens_476 | MN908947.3 | 3555 | 5821 | - | ORF1ab | 2 | find_circ | SRR11550044 |
| SARS-CoV-2_circ_Homo_sapiens_476 | MN908947.3 | 3555 | 5821 | - | ORF1ab | 2 | find_circ | SRR11550045 |
| SARS-CoV-2_circ_Homo_sapiens_48 | MN908947.3 | 16 | 6999 | - | ORF1ab | 2 | find_circ | SRR11550043 |
| SARS-CoV-2_circ_Homo_sapiens_484 | MN908947.3 | 3583 | 4560 | + | ORF1ab | 2 | find_circ | SRR11550046 |
| SARS-CoV-2_circ_Homo_sapiens_488 | MN908947.3 | 3717 | 29276 | + | ORF6;M;E;ORF3a;S;N;ORF8;ORF7a;ORF7b;ORF1ab | 2 | find_circ | SRR11550043 |
| SARS-CoV-2_circ_Homo_sapiens_49 | MN908947.3 | 16 | 7278 | - | ORF1ab | 2 | find_circ | SRR11550045 |
| SARS-CoV-2_circ_Homo_sapiens_490 | MN908947.3 | 3737 | 4379 | - | ORF1ab | 2 | find_circ | SRR11550045 |
| SARS-CoV-2_circ_Homo_sapiens_491 | MN908947.3 | 3737 | 4379 | + | ORF1ab | 2 | circRNA_finder | SRR11550045 |
| SARS-CoV-2_circ_Homo_sapiens_492 | MN908947.3 | 3737 | 5525 | - | ORF1ab | 2 | find_circ | SRR11550045 |
| SARS-CoV-2_circ_Homo_sapiens_494 | MN908947.3 | 3744 | 4154 | - | ORF1ab | 2 | find_circ | SRR11550046 |
| SARS-CoV-2_circ_Homo_sapiens_499 | MN908947.3 | 3756 | 4308 | + | ORF1ab | 2 | find_circ | SRR11550043 |
| SARS-CoV-2_circ_Homo_sapiens_5 | MN908947.3 | 8 | 442 | + | ORF1ab | 2 | find_circ | SRR11550043 |
| SARS-CoV-2_circ_Homo_sapiens_502 | MN908947.3 | 3788 | 4157 | - | ORF1ab | 2 | find_circ | SRR11550044 |
| SARS-CoV-2_circ_Homo_sapiens_505 | MN908947.3 | 3818 | 4154 | - | ORF1ab | 2 | find_circ | SRR11550044 |
| SARS-CoV-2_circ_Homo_sapiens_51 | MN908947.3 | 16 | 10457 | - | ORF1ab | 2 | find_circ | SRR11550044 |
| SARS-CoV-2_circ_Homo_sapiens_517 | MN908947.3 | 4125 | 4520 | + | ORF1ab | 2 | find_circ | SRR11550046 |
| SARS-CoV-2_circ_Homo_sapiens_518 | MN908947.3 | 4125 | 5048 | + | ORF1ab | 2 | circRNA_finder | SRR11550046 |
| SARS-CoV-2_circ_Homo_sapiens_518 | MN908947.3 | 4125 | 5048 | + | ORF1ab | 2 | find_circ | SRR11550046 |
| SARS-CoV-2_circ_Homo_sapiens_52 | MN908947.3 | 16 | 22746 | - | S;ORF1ab | 2 | find_circ | SRR11550046 |
| SARS-CoV-2_circ_Homo_sapiens_522 | MN908947.3 | 4170 | 4585 | - | ORF1ab | 2 | CIRI2 | SRR11550045 |
| SARS-CoV-2_circ_Homo_sapiens_528 | MN908947.3 | 4219 | 9190 | + | ORF1ab | 2 | find_circ | SRR11550045 |
| SARS-CoV-2_circ_Homo_sapiens_528 | MN908947.3 | 4219 | 9190 | + | ORF1ab | 2 | circRNA_finder | SRR11550045 |
| SARS-CoV-2_circ_Homo_sapiens_529 | MN908947.3 | 4284 | 4441 | + | ORF1ab | 2 | find_circ | SRR11550045 |
| SARS-CoV-2_circ_Homo_sapiens_53 | MN908947.3 | 16 | 26775 | - | M;E;ORF3a;S;ORF1ab | 2 | find_circ | SRR11550045 |
| SARS-CoV-2_circ_Homo_sapiens_530 | MN908947.3 | 4284 | 4835 | + | ORF1ab | 2 | circRNA_finder | SRR11550045 |
| SARS-CoV-2_circ_Homo_sapiens_532 | MN908947.3 | 4286 | 4835 | + | ORF1ab | 2 | find_circ | SRR11550045 |
| SARS-CoV-2_circ_Homo_sapiens_532 | MN908947.3 | 4286 | 4835 | + | ORF1ab | 2 | circRNA_finder | SRR11550045 |
| SARS-CoV-2_circ_Homo_sapiens_536 | MN908947.3 | 4362 | 8900 | + | ORF1ab | 2 | CIRI2 | SRR11550043 |
| SARS-CoV-2_circ_Homo_sapiens_538 | MN908947.3 | 4473 | 5525 | - | ORF1ab | 2 | find_circ | SRR11550046 |
| SARS-CoV-2_circ_Homo_sapiens_54 | MN908947.3 | 16 | 26868 | - | M;E;ORF3a;S;ORF1ab | 2 | CIRI2 | SRR11550046 |
| SARS-CoV-2_circ_Homo_sapiens_543 | MN908947.3 | 4535 | 4794 | - | ORF1ab | 2 | find_circ | SRR11550046 |
| SARS-CoV-2_circ_Homo_sapiens_545 | MN908947.3 | 4535 | 4831 | - | ORF1ab | 2 | find_circ | SRR11550043 |
| SARS-CoV-2_circ_Homo_sapiens_55 | MN908947.3 | 16 | 26954 | - | M;E;ORF3a;S;ORF1ab | 2 | find_circ | SRR11550046 |
| SARS-CoV-2_circ_Homo_sapiens_557 | MN908947.3 | 4543 | 27678 | - | ORF6;M;E;ORF3a;S;ORF7a;ORF1ab | 2 | find_circ | SRR11550045 |
| SARS-CoV-2_circ_Homo_sapiens_561 | MN908947.3 | 4560 | 4835 | + | ORF1ab | 2 | CIRI2 | SRR11550046 |
| SARS-CoV-2_circ_Homo_sapiens_567 | MN908947.3 | 4578 | 5821 | - | ORF1ab | 2 | find_circ | SRR11550046 |
| SARS-CoV-2_circ_Homo_sapiens_578 | MN908947.3 | 4629 | 4831 | - | ORF1ab | 2 | find_circ | SRR11550046 |
| SARS-CoV-2_circ_Homo_sapiens_579 | MN908947.3 | 4629 | 4964 | - | ORF1ab | 2 | find_circ | SRR11550046 |
| SARS-CoV-2_circ_Homo_sapiens_580 | MN908947.3 | 4704 | 14575 | - | ORF1ab | 2 | find_circ | SRR11550046 |
| SARS-CoV-2_circ_Homo_sapiens_581 | MN908947.3 | 4704 | 14575 | + | ORF1ab | 2 | circRNA_finder | SRR11550046 |
| SARS-CoV-2_circ_Homo_sapiens_582 | MN908947.3 | 4828 | 5391 | - | ORF1ab | 2 | find_circ | SRR11550046 |
| SARS-CoV-2_circ_Homo_sapiens_583 | MN908947.3 | 4828 | 5391 | + | ORF1ab | 2 | circRNA_finder | SRR11550046 |
| SARS-CoV-2_circ_Homo_sapiens_587 | MN908947.3 | 4836 | 5462 | + | ORF1ab | 2 | find_circ | SRR11550045 |
| SARS-CoV-2_circ_Homo_sapiens_59 | MN908947.3 | 16 | 29631 | - | ORF6;ORF10;M;E;ORF3a;S;N;ORF8;ORF7a;ORF7b;ORF1ab | 2 | find_circ | SRR11550045 |
| SARS-CoV-2_circ_Homo_sapiens_591 | MN908947.3 | 4842 | 5512 | + | ORF1ab | 2 | find_circ | SRR11550046 |
| SARS-CoV-2_circ_Homo_sapiens_594 | MN908947.3 | 4853 | 7760 | + | ORF1ab | 2 | circRNA_finder | SRR11550045 |
| SARS-CoV-2_circ_Homo_sapiens_594 | MN908947.3 | 4853 | 7760 | + | ORF1ab | 2 | find_circ | SRR11550045 |
| SARS-CoV-2_circ_Homo_sapiens_595 | MN908947.3 | 4877 | 5710 | - | ORF1ab | 2 | find_circ | SRR11550045 |
| SARS-CoV-2_circ_Homo_sapiens_596 | MN908947.3 | 4882 | 5186 | + | ORF1ab | 2 | find_circ | SRR11550045 |
| SARS-CoV-2_circ_Homo_sapiens_597 | MN908947.3 | 4882 | 5396 | + | ORF1ab | 2 | find_circ | SRR11550045 |
| SARS-CoV-2_circ_Homo_sapiens_597 | MN908947.3 | 4882 | 5396 | + | ORF1ab | 2 | circRNA_finder | SRR11550045 |
| SARS-CoV-2_circ_Homo_sapiens_599 | MN908947.3 | 4891 | 5387 | - | ORF1ab | 2 | find_circ | SRR11550044 |
| SARS-CoV-2_circ_Homo_sapiens_6 | MN908947.3 | 8 | 514 | + | ORF1ab | 2 | find_circ | SRR11550046 |
| SARS-CoV-2_circ_Homo_sapiens_60 | MN908947.3 | 16 | 29665 | - | ORF6;ORF10;M;E;ORF3a;S;N;ORF8;ORF7a;ORF7b;ORF1ab | 2 | CIRI2 | SRR11550043 |
| SARS-CoV-2_circ_Homo_sapiens_600 | MN908947.3 | 4891 | 5387 | + | ORF1ab | 2 | circRNA_finder | SRR11550044 |
| SARS-CoV-2_circ_Homo_sapiens_601 | MN908947.3 | 4891 | 5783 | - | ORF1ab | 2 | find_circ | SRR11550045 |
| SARS-CoV-2_circ_Homo_sapiens_602 | MN908947.3 | 4891 | 5783 | + | ORF1ab | 2 | circRNA_finder | SRR11550045 |
| SARS-CoV-2_circ_Homo_sapiens_605 | MN908947.3 | 4901 | 5387 | - | ORF1ab | 2 | find_circ | SRR11550045 |
| SARS-CoV-2_circ_Homo_sapiens_605 | MN908947.3 | 4901 | 5387 | - | ORF1ab | 2 | CIRI2 | SRR11550045 |
| SARS-CoV-2_circ_Homo_sapiens_606 | MN908947.3 | 4901 | 5391 | - | ORF1ab | 2 | find_circ | SRR11550045 |
| SARS-CoV-2_circ_Homo_sapiens_61 | MN908947.3 | 24 | 509 | + | ORF1ab | 2 | CIRI2 | SRR11550045 |
| SARS-CoV-2_circ_Homo_sapiens_617 | MN908947.3 | 4914 | 5462 | + | ORF1ab | 2 | find_circ | SRR11550044 |
| SARS-CoV-2_circ_Homo_sapiens_62 | MN908947.3 | 24 | 514 | + | ORF1ab | 2 | find_circ | SRR11550045 |
| SARS-CoV-2_circ_Homo_sapiens_622 | MN908947.3 | 4921 | 5525 | + | ORF1ab | 2 | circRNA_finder | SRR11550045 |
| SARS-CoV-2_circ_Homo_sapiens_623 | MN908947.3 | 4921 | 5588 | - | ORF1ab | 2 | find_circ | SRR11550046 |
| SARS-CoV-2_circ_Homo_sapiens_624 | MN908947.3 | 4921 | 5588 | + | ORF1ab | 2 | circRNA_finder | SRR11550046 |
| SARS-CoV-2_circ_Homo_sapiens_625 | MN908947.3 | 4921 | 5621 | - | ORF1ab | 2 | CIRI2 | SRR11550043 |
| SARS-CoV-2_circ_Homo_sapiens_63 | MN908947.3 | 24 | 1360 | + | ORF1ab | 2 | find_circ | SRR11550046 |
| SARS-CoV-2_circ_Homo_sapiens_632 | MN908947.3 | 4941 | 5090 | - | ORF1ab | 2 | find_circ | SRR11550043 |
| SARS-CoV-2_circ_Homo_sapiens_634 | MN908947.3 | 4963 | 5331 | + | ORF1ab | 2 | find_circ | SRR11550046 |
| SARS-CoV-2_circ_Homo_sapiens_635 | MN908947.3 | 4963 | 5503 | + | ORF1ab | 2 | find_circ | SRR11550046 |
| SARS-CoV-2_circ_Homo_sapiens_636 | MN908947.3 | 4963 | 5555 | + | ORF1ab | 2 | find_circ | SRR11550043 |
| SARS-CoV-2_circ_Homo_sapiens_639 | MN908947.3 | 4994 | 5357 | - | ORF1ab | 2 | find_circ | SRR11550044 |
| SARS-CoV-2_circ_Homo_sapiens_64 | MN908947.3 | 24 | 3134 | + | ORF1ab | 2 | find_circ | SRR11550045 |
| SARS-CoV-2_circ_Homo_sapiens_641 | MN908947.3 | 4994 | 5525 | - | ORF1ab | 2 | find_circ | SRR11550045 |
| SARS-CoV-2_circ_Homo_sapiens_642 | MN908947.3 | 4994 | 5816 | - | ORF1ab | 2 | find_circ | SRR11550044 |
| SARS-CoV-2_circ_Homo_sapiens_645 | MN908947.3 | 5021 | 5821 | - | ORF1ab | 2 | find_circ | SRR11550043 |
| SARS-CoV-2_circ_Homo_sapiens_646 | MN908947.3 | 5116 | 5525 | - | ORF1ab | 2 | find_circ | SRR11550043 |
| SARS-CoV-2_circ_Homo_sapiens_646 | MN908947.3 | 5116 | 5525 | - | ORF1ab | 2 | find_circ | SRR11550046 |
| SARS-CoV-2_circ_Homo_sapiens_651 | MN908947.3 | 5116 | 8292 | - | ORF1ab | 2 | find_circ | SRR11550046 |
| SARS-CoV-2_circ_Homo_sapiens_653 | MN908947.3 | 5130 | 5357 | - | ORF1ab | 2 | find_circ | SRR11550045 |
| SARS-CoV-2_circ_Homo_sapiens_654 | MN908947.3 | 5130 | 5391 | - | ORF1ab | 2 | find_circ | SRR11550046 |
| SARS-CoV-2_circ_Homo_sapiens_655 | MN908947.3 | 5130 | 5391 | + | ORF1ab | 2 | circRNA_finder | SRR11550046 |
| SARS-CoV-2_circ_Homo_sapiens_659 | MN908947.3 | 5130 | 5588 | - | ORF1ab | 2 | find_circ | SRR11550046 |
| SARS-CoV-2_circ_Homo_sapiens_659 | MN908947.3 | 5130 | 5588 | - | ORF1ab | 2 | find_circ | SRR11550043 |
| SARS-CoV-2_circ_Homo_sapiens_660 | MN908947.3 | 5130 | 5588 | + | ORF1ab | 2 | circRNA_finder | SRR11550043 |
| SARS-CoV-2_circ_Homo_sapiens_660 | MN908947.3 | 5130 | 5588 | + | ORF1ab | 2 | circRNA_finder | SRR11550045 |
| SARS-CoV-2_circ_Homo_sapiens_661 | MN908947.3 | 5130 | 5621 | - | ORF1ab | 2 | find_circ | SRR11550045 |
| SARS-CoV-2_circ_Homo_sapiens_662 | MN908947.3 | 5130 | 5621 | + | ORF1ab | 2 | circRNA_finder | SRR11550045 |
| SARS-CoV-2_circ_Homo_sapiens_669 | MN908947.3 | 5141 | 5525 | - | ORF1ab | 2 | find_circ | SRR11550046 |
| SARS-CoV-2_circ_Homo_sapiens_67 | MN908947.3 | 37 | 3539 | - | ORF1ab | 2 | find_circ | SRR11550044 |
| SARS-CoV-2_circ_Homo_sapiens_67 | MN908947.3 | 37 | 3539 | - | ORF1ab | 2 | find_circ | SRR11550043 |
| SARS-CoV-2_circ_Homo_sapiens_672 | MN908947.3 | 5148 | 6632 | - | ORF1ab | 2 | find_circ | SRR11550045 |
| SARS-CoV-2_circ_Homo_sapiens_673 | MN908947.3 | 5148 | 6632 | + | ORF1ab | 2 | circRNA_finder | SRR11550045 |
| SARS-CoV-2_circ_Homo_sapiens_674 | MN908947.3 | 5188 | 5555 | + | ORF1ab | 2 | find_circ | SRR11550045 |
| SARS-CoV-2_circ_Homo_sapiens_676 | MN908947.3 | 5188 | 5703 | + | ORF1ab | 2 | find_circ | SRR11550045 |
| SARS-CoV-2_circ_Homo_sapiens_681 | MN908947.3 | 5366 | 5710 | - | ORF1ab | 2 | find_circ | SRR11550044 |
| SARS-CoV-2_circ_Homo_sapiens_681 | MN908947.3 | 5366 | 5710 | - | ORF1ab | 2 | find_circ | SRR11550045 |
| SARS-CoV-2_circ_Homo_sapiens_682 | MN908947.3 | 5366 | 5710 | + | ORF1ab | 2 | circRNA_finder | SRR11550045 |
| SARS-CoV-2_circ_Homo_sapiens_685 | MN908947.3 | 5366 | 5821 | + | ORF1ab | 2 | circRNA_finder | SRR11550046 |
| SARS-CoV-2_circ_Homo_sapiens_689 | MN908947.3 | 5368 | 8336 | + | ORF1ab | 2 | find_circ | SRR11550043 |
| SARS-CoV-2_circ_Homo_sapiens_69 | MN908947.3 | 37 | 26814 | - | M;E;ORF3a;S;ORF1ab | 2 | find_circ | SRR11550046 |
| SARS-CoV-2_circ_Homo_sapiens_692 | MN908947.3 | 5374 | 5720 | + | ORF1ab | 2 | find_circ | SRR11550045 |
| SARS-CoV-2_circ_Homo_sapiens_694 | MN908947.3 | 5426 | 28325 | + | ORF6;M;E;ORF3a;S;N;ORF8;ORF7a;ORF7b;ORF1ab | 2 | find_circ | SRR11550045 |
| SARS-CoV-2_circ_Homo_sapiens_694 | MN908947.3 | 5426 | 28325 | + | ORF6;M;E;ORF3a;S;N;ORF8;ORF7a;ORF7b;ORF1ab | 2 | circRNA_finder | SRR11550045 |
| SARS-CoV-2_circ_Homo_sapiens_698 | MN908947.3 | 5490 | 23339 | + | S;ORF1ab | 2 | circRNA_finder | SRR11550046 |
| SARS-CoV-2_circ_Homo_sapiens_698 | MN908947.3 | 5490 | 23339 | + | S;ORF1ab | 2 | find_circ | SRR11550046 |
| SARS-CoV-2_circ_Homo_sapiens_700 | MN908947.3 | 5527 | 5906 | - | ORF1ab | 2 | find_circ | SRR11550046 |
| SARS-CoV-2_circ_Homo_sapiens_702 | MN908947.3 | 5527 | 5978 | + | ORF1ab | 2 | circRNA_finder | SRR11550045 |
| SARS-CoV-2_circ_Homo_sapiens_705 | MN908947.3 | 5527 | 26780 | - | M;E;ORF3a;S;ORF1ab | 2 | find_circ | SRR11550045 |
| SARS-CoV-2_circ_Homo_sapiens_706 | MN908947.3 | 5527 | 29607 | - | ORF6;ORF10;M;E;ORF3a;S;N;ORF8;ORF7a;ORF7b;ORF1ab | 2 | find_circ | SRR11550044 |
| SARS-CoV-2_circ_Homo_sapiens_711 | MN908947.3 | 5622 | 6680 | - | ORF1ab | 2 | find_circ | SRR11550044 |
| SARS-CoV-2_circ_Homo_sapiens_717 | MN908947.3 | 5622 | 9953 | - | ORF1ab | 2 | CIRI2 | SRR11550043 |
| SARS-CoV-2_circ_Homo_sapiens_72 | MN908947.3 | 41 | 400 | + | ORF1ab | 2 | circRNA_finder | SRR11550046 |
| SARS-CoV-2_circ_Homo_sapiens_725 | MN908947.3 | 5658 | 24806 | + | S;ORF1ab | 2 | find_circ | SRR11550045 |
| SARS-CoV-2_circ_Homo_sapiens_725 | MN908947.3 | 5658 | 24806 | + | S;ORF1ab | 2 | circRNA_finder | SRR11550045 |
| SARS-CoV-2_circ_Homo_sapiens_737 | MN908947.3 | 5823 | 6680 | - | ORF1ab | 2 | find_circ | SRR11550045 |
| SARS-CoV-2_circ_Homo_sapiens_737 | MN908947.3 | 5823 | 6680 | - | ORF1ab | 2 | find_circ | SRR11550046 |
| SARS-CoV-2_circ_Homo_sapiens_738 | MN908947.3 | 5823 | 6680 | + | ORF1ab | 2 | circRNA_finder | SRR11550046 |
| SARS-CoV-2_circ_Homo_sapiens_741 | MN908947.3 | 5823 | 14617 | - | ORF1ab | 2 | find_circ | SRR11550044 |
| SARS-CoV-2_circ_Homo_sapiens_742 | MN908947.3 | 5823 | 14617 | + | ORF1ab | 2 | circRNA_finder | SRR11550044 |
| SARS-CoV-2_circ_Homo_sapiens_744 | MN908947.3 | 5823 | 29444 | - | ORF6;M;E;ORF3a;S;N;ORF8;ORF7a;ORF7b;ORF1ab | 2 | find_circ | SRR11550043 |
| SARS-CoV-2_circ_Homo_sapiens_750 | MN908947.3 | 5827 | 7169 | - | ORF1ab | 2 | find_circ | SRR11550046 |
| SARS-CoV-2_circ_Homo_sapiens_753 | MN908947.3 | 5827 | 8921 | - | ORF1ab | 2 | find_circ | SRR11550043 |
| SARS-CoV-2_circ_Homo_sapiens_754 | MN908947.3 | 5827 | 8921 | + | ORF1ab | 2 | circRNA_finder | SRR11550043 |
| SARS-CoV-2_circ_Homo_sapiens_759 | MN908947.3 | 5908 | 6170 | - | ORF1ab | 2 | find_circ | SRR11550045 |
| SARS-CoV-2_circ_Homo_sapiens_761 | MN908947.3 | 5908 | 6557 | - | ORF1ab | 2 | find_circ | SRR11550046 |
| SARS-CoV-2_circ_Homo_sapiens_762 | MN908947.3 | 5908 | 6572 | - | ORF1ab | 2 | find_circ | SRR11550045 |
| SARS-CoV-2_circ_Homo_sapiens_763 | MN908947.3 | 5908 | 6572 | + | ORF1ab | 2 | circRNA_finder | SRR11550045 |
| SARS-CoV-2_circ_Homo_sapiens_766 | MN908947.3 | 5908 | 6680 | - | ORF1ab | 2 | find_circ | SRR11550044 |
| SARS-CoV-2_circ_Homo_sapiens_766 | MN908947.3 | 5908 | 6680 | - | ORF1ab | 2 | find_circ | SRR11550043 |
| SARS-CoV-2_circ_Homo_sapiens_767 | MN908947.3 | 5908 | 6680 | + | ORF1ab | 2 | circRNA_finder | SRR11550044 |
| SARS-CoV-2_circ_Homo_sapiens_767 | MN908947.3 | 5908 | 6680 | + | ORF1ab | 2 | circRNA_finder | SRR11550043 |
| SARS-CoV-2_circ_Homo_sapiens_77 | MN908947.3 | 41 | 856 | - | ORF1ab | 2 | find_circ | SRR11550046 |
| SARS-CoV-2_circ_Homo_sapiens_773 | MN908947.3 | 5908 | 7172 | - | ORF1ab | 2 | find_circ | SRR11550044 |
| SARS-CoV-2_circ_Homo_sapiens_775 | MN908947.3 | 5908 | 8292 | + | ORF1ab | 2 | circRNA_finder | SRR11550045 |
| SARS-CoV-2_circ_Homo_sapiens_776 | MN908947.3 | 5908 | 11961 | - | ORF1ab | 2 | find_circ | SRR11550045 |
| SARS-CoV-2_circ_Homo_sapiens_777 | MN908947.3 | 5908 | 17517 | - | ORF1ab | 2 | find_circ | SRR11550043 |
| SARS-CoV-2_circ_Homo_sapiens_78 | MN908947.3 | 41 | 856 | + | ORF1ab | 2 | circRNA_finder | SRR11550046 |
| SARS-CoV-2_circ_Homo_sapiens_781 | MN908947.3 | 5989 | 6577 | - | ORF1ab | 2 | find_circ | SRR11550044 |
| SARS-CoV-2_circ_Homo_sapiens_787 | MN908947.3 | 6037 | 6512 | + | ORF1ab | 2 | find_circ | SRR11550045 |
| SARS-CoV-2_circ_Homo_sapiens_787 | MN908947.3 | 6037 | 6512 | + | ORF1ab | 2 | circRNA_finder | SRR11550045 |
| SARS-CoV-2_circ_Homo_sapiens_788 | MN908947.3 | 6053 | 6512 | + | ORF1ab | 2 | circRNA_finder | SRR11550046 |
| SARS-CoV-2_circ_Homo_sapiens_788 | MN908947.3 | 6053 | 6512 | + | ORF1ab | 2 | find_circ | SRR11550046 |
| SARS-CoV-2_circ_Homo_sapiens_789 | MN908947.3 | 6053 | 6562 | + | ORF1ab | 2 | find_circ | SRR11550044 |
| SARS-CoV-2_circ_Homo_sapiens_789 | MN908947.3 | 6053 | 6562 | + | ORF1ab | 2 | circRNA_finder | SRR11550044 |
| SARS-CoV-2_circ_Homo_sapiens_789 | MN908947.3 | 6053 | 6562 | + | ORF1ab | 2 | find_circ | SRR11550045 |
| SARS-CoV-2_circ_Homo_sapiens_796 | MN908947.3 | 6225 | 6581 | - | ORF1ab | 2 | find_circ | SRR11550044 |
| SARS-CoV-2_circ_Homo_sapiens_80 | MN908947.3 | 41 | 2535 | - | ORF1ab | 2 | find_circ | SRR11550045 |
| SARS-CoV-2_circ_Homo_sapiens_800 | MN908947.3 | 6590 | 7026 | + | ORF1ab | 2 | find_circ | SRR11550043 |
| SARS-CoV-2_circ_Homo_sapiens_800 | MN908947.3 | 6590 | 7026 | + | ORF1ab | 2 | circRNA_finder | SRR11550043 |
| SARS-CoV-2_circ_Homo_sapiens_807 | MN908947.3 | 6648 | 29586 | + | ORF6;ORF10;M;E;ORF3a;S;N;ORF8;ORF7a;ORF7b;ORF1ab | 2 | find_circ | SRR11550046 |
| SARS-CoV-2_circ_Homo_sapiens_808 | MN908947.3 | 6681 | 7272 | + | ORF1ab | 2 | find_circ | SRR11550046 |
| SARS-CoV-2_circ_Homo_sapiens_810 | MN908947.3 | 6681 | 7692 | + | ORF1ab | 2 | find_circ | SRR11550044 |
| SARS-CoV-2_circ_Homo_sapiens_815 | MN908947.3 | 6681 | 9002 | + | ORF1ab | 2 | find_circ | SRR11550043 |
| SARS-CoV-2_circ_Homo_sapiens_817 | MN908947.3 | 6731 | 7164 | - | ORF1ab | 2 | find_circ | SRR11550043 |
| SARS-CoV-2_circ_Homo_sapiens_823 | MN908947.3 | 6814 | 10043 | - | ORF1ab | 2 | find_circ | SRR11550046 |
| SARS-CoV-2_circ_Homo_sapiens_829 | MN908947.3 | 7636 | 8280 | - | ORF1ab | 2 | find_circ | SRR11550046 |
| SARS-CoV-2_circ_Homo_sapiens_83 | MN908947.3 | 41 | 26946 | - | M;E;ORF3a;S;ORF1ab | 2 | find_circ | SRR11550046 |
| SARS-CoV-2_circ_Homo_sapiens_834 | MN908947.3 | 7778 | 8292 | - | ORF1ab | 2 | find_circ | SRR11550045 |
| SARS-CoV-2_circ_Homo_sapiens_835 | MN908947.3 | 7778 | 8759 | - | ORF1ab | 2 | find_circ | SRR11550046 |
| SARS-CoV-2_circ_Homo_sapiens_838 | MN908947.3 | 7778 | 10031 | - | ORF1ab | 2 | find_circ | SRR11550044 |
| SARS-CoV-2_circ_Homo_sapiens_84 | MN908947.3 | 41 | 27493 | - | ORF6;M;E;ORF3a;S;ORF7a;ORF1ab | 2 | CIRI2 | SRR11550043 |
| SARS-CoV-2_circ_Homo_sapiens_841 | MN908947.3 | 7946 | 8921 | - | ORF1ab | 2 | find_circ | SRR11550046 |
| SARS-CoV-2_circ_Homo_sapiens_842 | MN908947.3 | 7955 | 10395 | + | ORF1ab | 2 | find_circ | SRR11550045 |
| SARS-CoV-2_circ_Homo_sapiens_842 | MN908947.3 | 7955 | 10395 | + | ORF1ab | 2 | circRNA_finder | SRR11550045 |
| SARS-CoV-2_circ_Homo_sapiens_843 | MN908947.3 | 7968 | 8504 | - | ORF1ab | 2 | CIRI2 | SRR11550043 |
| SARS-CoV-2_circ_Homo_sapiens_848 | MN908947.3 | 8091 | 11918 | - | ORF1ab | 2 | find_circ | SRR11550044 |
| SARS-CoV-2_circ_Homo_sapiens_849 | MN908947.3 | 8161 | 16678 | - | ORF1ab | 2 | find_circ | SRR11550045 |
| SARS-CoV-2_circ_Homo_sapiens_854 | MN908947.3 | 8272 | 12110 | + | ORF1ab | 2 | find_circ | SRR11550043 |
| SARS-CoV-2_circ_Homo_sapiens_854 | MN908947.3 | 8272 | 12110 | + | ORF1ab | 2 | circRNA_finder | SRR11550043 |
| SARS-CoV-2_circ_Homo_sapiens_859 | MN908947.3 | 8293 | 8916 | - | ORF1ab | 2 | find_circ | SRR11550046 |
| SARS-CoV-2_circ_Homo_sapiens_86 | MN908947.3 | 41 | 28602 | - | ORF6;M;E;ORF3a;S;N;ORF8;ORF7a;ORF7b;ORF1ab | 2 | find_circ | SRR11550046 |
| SARS-CoV-2_circ_Homo_sapiens_860 | MN908947.3 | 8293 | 8916 | + | ORF1ab | 2 | circRNA_finder | SRR11550046 |
| SARS-CoV-2_circ_Homo_sapiens_861 | MN908947.3 | 8293 | 8921 | - | ORF1ab | 2 | find_circ | SRR11550044 |
| SARS-CoV-2_circ_Homo_sapiens_867 | MN908947.3 | 8293 | 10031 | - | ORF1ab | 2 | CIRI2 | SRR11550046 |
| SARS-CoV-2_circ_Homo_sapiens_869 | MN908947.3 | 8293 | 10038 | - | ORF1ab | 2 | find_circ | SRR11550046 |
| SARS-CoV-2_circ_Homo_sapiens_870 | MN908947.3 | 8293 | 10038 | + | ORF1ab | 2 | circRNA_finder | SRR11550046 |
| SARS-CoV-2_circ_Homo_sapiens_882 | MN908947.3 | 8300 | 26078 | - | ORF3a;S;ORF1ab | 2 | find_circ | SRR11550045 |
| SARS-CoV-2_circ_Homo_sapiens_883 | MN908947.3 | 8300 | 26078 | + | ORF3a;S;ORF1ab | 2 | circRNA_finder | SRR11550045 |
| SARS-CoV-2_circ_Homo_sapiens_884 | MN908947.3 | 8304 | 8717 | + | ORF1ab | 2 | find_circ | SRR11550045 |
| SARS-CoV-2_circ_Homo_sapiens_884 | MN908947.3 | 8304 | 8717 | + | ORF1ab | 2 | circRNA_finder | SRR11550045 |
| SARS-CoV-2_circ_Homo_sapiens_885 | MN908947.3 | 8312 | 11108 | - | ORF1ab | 2 | find_circ | SRR11550045 |
| SARS-CoV-2_circ_Homo_sapiens_887 | MN908947.3 | 8327 | 8921 | - | ORF1ab | 2 | find_circ | SRR11550045 |
| SARS-CoV-2_circ_Homo_sapiens_888 | MN908947.3 | 8327 | 8921 | + | ORF1ab | 2 | circRNA_finder | SRR11550045 |
| SARS-CoV-2_circ_Homo_sapiens_890 | MN908947.3 | 8372 | 8792 | + | ORF1ab | 2 | find_circ | SRR11550046 |
| SARS-CoV-2_circ_Homo_sapiens_890 | MN908947.3 | 8372 | 8792 | + | ORF1ab | 2 | circRNA_finder | SRR11550046 |
| SARS-CoV-2_circ_Homo_sapiens_891 | MN908947.3 | 8415 | 9269 | + | ORF1ab | 2 | find_circ | SRR11550046 |
| SARS-CoV-2_circ_Homo_sapiens_895 | MN908947.3 | 8669 | 9569 | - | ORF1ab | 2 | find_circ | SRR11550046 |
| SARS-CoV-2_circ_Homo_sapiens_896 | MN908947.3 | 8696 | 9574 | + | ORF1ab | 2 | find_circ | SRR11550046 |
| SARS-CoV-2_circ_Homo_sapiens_898 | MN908947.3 | 8723 | 10439 | - | ORF1ab | 2 | find_circ | SRR11550044 |
| SARS-CoV-2_circ_Homo_sapiens_899 | MN908947.3 | 8723 | 10439 | + | ORF1ab | 2 | circRNA_finder | SRR11550044 |
| SARS-CoV-2_circ_Homo_sapiens_9 | MN908947.3 | 8 | 1357 | + | ORF1ab | 2 | find_circ | SRR11550043 |
| SARS-CoV-2_circ_Homo_sapiens_902 | MN908947.3 | 8771 | 10038 | - | ORF1ab | 2 | find_circ | SRR11550045 |
| SARS-CoV-2_circ_Homo_sapiens_904 | MN908947.3 | 8771 | 10400 | - | ORF1ab | 2 | find_circ | SRR11550046 |
| SARS-CoV-2_circ_Homo_sapiens_906 | MN908947.3 | 8773 | 10031 | - | ORF1ab | 2 | find_circ | SRR11550044 |
| SARS-CoV-2_circ_Homo_sapiens_910 | MN908947.3 | 8773 | 10581 | - | ORF1ab | 2 | find_circ | SRR11550045 |
| SARS-CoV-2_circ_Homo_sapiens_911 | MN908947.3 | 8773 | 23423 | - | S;ORF1ab | 2 | find_circ | SRR11550046 |
| SARS-CoV-2_circ_Homo_sapiens_916 | MN908947.3 | 8782 | 10532 | + | ORF1ab | 2 | find_circ | SRR11550046 |
| SARS-CoV-2_circ_Homo_sapiens_922 | MN908947.3 | 8810 | 10009 | - | ORF1ab | 2 | find_circ | SRR11550046 |
| SARS-CoV-2_circ_Homo_sapiens_924 | MN908947.3 | 8922 | 9558 | - | ORF1ab | 2 | find_circ | SRR11550045 |
| SARS-CoV-2_circ_Homo_sapiens_93 | MN908947.3 | 72 | 1436 | - | ORF1ab | 2 | find_circ | SRR11550046 |
| SARS-CoV-2_circ_Homo_sapiens_931 | MN908947.3 | 8928 | 9583 | + | ORF1ab | 2 | circRNA_finder | SRR11550046 |
| SARS-CoV-2_circ_Homo_sapiens_931 | MN908947.3 | 8928 | 9583 | + | ORF1ab | 2 | find_circ | SRR11550046 |
| SARS-CoV-2_circ_Homo_sapiens_934 | MN908947.3 | 8928 | 10663 | + | ORF1ab | 2 | find_circ | SRR11550046 |
| SARS-CoV-2_circ_Homo_sapiens_936 | MN908947.3 | 8935 | 9583 | + | ORF1ab | 2 | find_circ | SRR11550045 |
| SARS-CoV-2_circ_Homo_sapiens_938 | MN908947.3 | 8957 | 13828 | - | ORF1ab | 2 | find_circ | SRR11550043 |
| SARS-CoV-2_circ_Homo_sapiens_94 | MN908947.3 | 72 | 1436 | + | ORF1ab | 2 | circRNA_finder | SRR11550046 |
| SARS-CoV-2_circ_Homo_sapiens_941 | MN908947.3 | 9057 | 9558 | - | ORF1ab | 2 | find_circ | SRR11550045 |
| SARS-CoV-2_circ_Homo_sapiens_942 | MN908947.3 | 9087 | 9176 | + | ORF1ab | 2 | find_circ | SRR11550044 |
| SARS-CoV-2_circ_Homo_sapiens_943 | MN908947.3 | 9106 | 9542 | + | ORF1ab | 2 | circRNA_finder | SRR11550045 |
| SARS-CoV-2_circ_Homo_sapiens_943 | MN908947.3 | 9106 | 9542 | + | ORF1ab | 2 | find_circ | SRR11550045 |
| SARS-CoV-2_circ_Homo_sapiens_944 | MN908947.3 | 9121 | 10043 | - | ORF1ab | 2 | find_circ | SRR11550044 |
| SARS-CoV-2_circ_Homo_sapiens_949 | MN908947.3 | 9123 | 10754 | - | ORF1ab | 2 | find_circ | SRR11550046 |
| SARS-CoV-2_circ_Homo_sapiens_95 | MN908947.3 | 72 | 29268 | - | ORF6;M;E;ORF3a;S;N;ORF8;ORF7a;ORF7b;ORF1ab | 2 | find_circ | SRR11550046 |
| SARS-CoV-2_circ_Homo_sapiens_951 | MN908947.3 | 9166 | 29631 | - | ORF6;ORF10;M;E;ORF3a;S;N;ORF8;ORF7a;ORF7b;ORF1ab | 2 | find_circ | SRR11550044 |
| SARS-CoV-2_circ_Homo_sapiens_953 | MN908947.3 | 9170 | 9450 | - | ORF1ab | 2 | find_circ | SRR11550045 |
| SARS-CoV-2_circ_Homo_sapiens_956 | MN908947.3 | 9170 | 11918 | - | ORF1ab | 2 | find_circ | SRR11550045 |
| SARS-CoV-2_circ_Homo_sapiens_959 | MN908947.3 | 9220 | 11178 | + | ORF1ab | 2 | circRNA_finder | SRR11550043 |
| SARS-CoV-2_circ_Homo_sapiens_959 | MN908947.3 | 9220 | 11178 | + | ORF1ab | 2 | find_circ | SRR11550043 |
| SARS-CoV-2_circ_Homo_sapiens_964 | MN908947.3 | 9426 | 10100 | + | ORF1ab | 2 | find_circ | SRR11550043 |
| SARS-CoV-2_circ_Homo_sapiens_967 | MN908947.3 | 9555 | 10045 | + | ORF1ab | 2 | find_circ | SRR11550045 |
| SARS-CoV-2_circ_Homo_sapiens_968 | MN908947.3 | 9560 | 10031 | - | ORF1ab | 2 | find_circ | SRR11550045 |
| SARS-CoV-2_circ_Homo_sapiens_969 | MN908947.3 | 9604 | 13357 | - | ORF1ab | 2 | find_circ | SRR11550046 |
| SARS-CoV-2_circ_Homo_sapiens_970 | MN908947.3 | 9604 | 13357 | + | ORF1ab | 2 | circRNA_finder | SRR11550046 |
| SARS-CoV-2_circ_Homo_sapiens_973 | MN908947.3 | 9636 | 10663 | + | ORF1ab | 2 | find_circ | SRR11550045 |
| SARS-CoV-2_circ_Homo_sapiens_974 | MN908947.3 | 9639 | 10009 | - | ORF1ab | 2 | find_circ | SRR11550045 |
| SARS-CoV-2_circ_Homo_sapiens_976 | MN908947.3 | 9639 | 10031 | + | ORF1ab | 2 | circRNA_finder | SRR11550043 |
| SARS-CoV-2_circ_Homo_sapiens_977 | MN908947.3 | 9639 | 10038 | - | ORF1ab | 2 | find_circ | SRR11550043 |
| SARS-CoV-2_circ_Homo_sapiens_978 | MN908947.3 | 9639 | 10038 | + | ORF1ab | 2 | circRNA_finder | SRR11550043 |
| SARS-CoV-2_circ_Homo_sapiens_979 | MN908947.3 | 9639 | 10147 | - | ORF1ab | 2 | find_circ | SRR11550043 |
| SARS-CoV-2_circ_Homo_sapiens_983 | MN908947.3 | 9746 | 10201 | - | ORF1ab | 2 | find_circ | SRR11550046 |
| SARS-CoV-2_circ_Homo_sapiens_984 | MN908947.3 | 9756 | 10043 | - | ORF1ab | 2 | find_circ | SRR11550043 |
| SARS-CoV-2_circ_Homo_sapiens_986 | MN908947.3 | 9762 | 13389 | + | ORF1ab | 2 | circRNA_finder | SRR11550045 |
| SARS-CoV-2_circ_Homo_sapiens_986 | MN908947.3 | 9762 | 13389 | + | ORF1ab | 2 | find_circ | SRR11550045 |
| SARS-CoV-2_circ_Homo_sapiens_991 | MN908947.3 | 9887 | 10164 | - | ORF1ab | 2 | find_circ | SRR11550045 |
| SARS-CoV-2_circ_Homo_sapiens_992 | MN908947.3 | 9891 | 10663 | + | ORF1ab | 2 | find_circ | SRR11550043 |
| SARS-CoV-2_circ_Homo_sapiens_992 | MN908947.3 | 9891 | 10663 | + | ORF1ab | 2 | circRNA_finder | SRR11550043 |
| SARS-CoV-2_circ_Homo_sapiens_999 | MN908947.3 | 9944 | 10439 | - | ORF1ab | 2 | find_circ | SRR11550045 |
| SARS-CoV-2_circ_Homo_sapiens_1001 | MN908947.3 | 9946 | 10395 | + | ORF1ab | 1 | circRNA_finder | SRR11550044 |
| SARS-CoV-2_circ_Homo_sapiens_1002 | MN908947.3 | 9946 | 10436 | + | ORF1ab | 1 | circRNA_finder | SRR11550044 |
| SARS-CoV-2_circ_Homo_sapiens_1003 | MN908947.3 | 9946 | 11286 | + | ORF1ab | 1 | circRNA_finder | SRR11550046 |
| SARS-CoV-2_circ_Homo_sapiens_1004 | MN908947.3 | 9946 | 23627 | + | S;ORF1ab | 1 | circRNA_finder | SRR11550045 |
| SARS-CoV-2_circ_Homo_sapiens_1005 | MN908947.3 | 9948 | 10324 | + | ORF1ab | 1 | circRNA_finder | SRR11550045 |
| SARS-CoV-2_circ_Homo_sapiens_1007 | MN908947.3 | 9948 | 13308 | + | ORF1ab | 1 | circRNA_finder | SRR11550045 |
| SARS-CoV-2_circ_Homo_sapiens_1008 | MN908947.3 | 9948 | 13389 | + | ORF1ab | 1 | circRNA_finder | SRR11550045 |
| SARS-CoV-2_circ_Homo_sapiens_1009 | MN908947.3 | 9948 | 27462 | + | ORF6;M;E;ORF3a;S;ORF7a;ORF1ab | 1 | circRNA_finder | SRR11550044 |
| SARS-CoV-2_circ_Homo_sapiens_101 | MN908947.3 | 237 | 668 | + | ORF1ab | 1 | circRNA_finder | SRR11550046 |
| SARS-CoV-2_circ_Homo_sapiens_1010 | MN908947.3 | 9951 | 10589 | + | ORF1ab | 1 | circRNA_finder | SRR11550044 |
| SARS-CoV-2_circ_Homo_sapiens_1017 | MN908947.3 | 10030 | 10524 | + | ORF1ab | 1 | circRNA_finder | SRR11550046 |
| SARS-CoV-2_circ_Homo_sapiens_1019 | MN908947.3 | 10030 | 10526 | + | ORF1ab | 1 | circRNA_finder | SRR11550045 |
| SARS-CoV-2_circ_Homo_sapiens_102 | MN908947.3 | 237 | 776 | + | ORF1ab | 1 | circRNA_finder | SRR11550043 |
| SARS-CoV-2_circ_Homo_sapiens_1023 | MN908947.3 | 10044 | 10393 | + | ORF1ab | 1 | circRNA_finder | SRR11550043 |
| SARS-CoV-2_circ_Homo_sapiens_1024 | MN908947.3 | 10044 | 10424 | + | ORF1ab | 1 | circRNA_finder | SRR11550046 |
| SARS-CoV-2_circ_Homo_sapiens_1025 | MN908947.3 | 10044 | 10493 | + | ORF1ab | 1 | circRNA_finder | SRR11550045 |
| SARS-CoV-2_circ_Homo_sapiens_1025 | MN908947.3 | 10044 | 10493 | + | ORF1ab | 1 | circRNA_finder | SRR11550044 |
| SARS-CoV-2_circ_Homo_sapiens_1026 | MN908947.3 | 10044 | 10499 | + | ORF1ab | 1 | circRNA_finder | SRR11550045 |
| SARS-CoV-2_circ_Homo_sapiens_1028 | MN908947.3 | 10044 | 29242 | + | ORF6;M;E;ORF3a;S;N;ORF8;ORF7a;ORF7b;ORF1ab | 1 | circRNA_finder | SRR11550044 |
| SARS-CoV-2_circ_Homo_sapiens_103 | MN908947.3 | 237 | 20442 | + | ORF1ab | 1 | circRNA_finder | SRR11550044 |
| SARS-CoV-2_circ_Homo_sapiens_1031 | MN908947.3 | 10066 | 10499 | + | ORF1ab | 1 | circRNA_finder | SRR11550046 |
| SARS-CoV-2_circ_Homo_sapiens_1033 | MN908947.3 | 10066 | 10663 | + | ORF1ab | 1 | circRNA_finder | SRR11550044 |
| SARS-CoV-2_circ_Homo_sapiens_1034 | MN908947.3 | 10066 | 16911 | + | ORF1ab | 1 | circRNA_finder | SRR11550046 |
| SARS-CoV-2_circ_Homo_sapiens_1035 | MN908947.3 | 10066 | 28403 | + | ORF6;M;E;ORF3a;S;N;ORF8;ORF7a;ORF7b;ORF1ab | 1 | circRNA_finder | SRR11550046 |
| SARS-CoV-2_circ_Homo_sapiens_1037 | MN908947.3 | 10066 | 29691 | + | ORF6;ORF10;M;E;ORF3a;S;N;ORF8;ORF7a;ORF7b;ORF1ab | 1 | circRNA_finder | SRR11550046 |
| SARS-CoV-2_circ_Homo_sapiens_1038 | MN908947.3 | 10126 | 10808 | + | ORF1ab | 1 | circRNA_finder | SRR11550046 |
| SARS-CoV-2_circ_Homo_sapiens_1039 | MN908947.3 | 10134 | 10439 | + | ORF1ab | 1 | circRNA_finder | SRR11550046 |
| SARS-CoV-2_circ_Homo_sapiens_104 | MN908947.3 | 237 | 23383 | + | S;ORF1ab | 1 | circRNA_finder | SRR11550045 |
| SARS-CoV-2_circ_Homo_sapiens_1040 | MN908947.3 | 10134 | 10649 | + | ORF1ab | 1 | circRNA_finder | SRR11550043 |
| SARS-CoV-2_circ_Homo_sapiens_1041 | MN908947.3 | 10157 | 10439 | + | ORF1ab | 1 | circRNA_finder | SRR11550045 |
| SARS-CoV-2_circ_Homo_sapiens_1043 | MN908947.3 | 10157 | 11918 | + | ORF1ab | 1 | circRNA_finder | SRR11550046 |
| SARS-CoV-2_circ_Homo_sapiens_1044 | MN908947.3 | 10157 | 13139 | + | ORF1ab | 1 | circRNA_finder | SRR11550045 |
| SARS-CoV-2_circ_Homo_sapiens_1045 | MN908947.3 | 10157 | 20659 | + | ORF1ab | 1 | circRNA_finder | SRR11550046 |
| SARS-CoV-2_circ_Homo_sapiens_1046 | MN908947.3 | 10157 | 27015 | + | M;E;ORF3a;S;ORF1ab | 1 | circRNA_finder | SRR11550045 |
| SARS-CoV-2_circ_Homo_sapiens_1047 | MN908947.3 | 10161 | 10395 | + | ORF1ab | 1 | circRNA_finder | SRR11550046 |
| SARS-CoV-2_circ_Homo_sapiens_105 | MN908947.3 | 255 | 28686 | + | ORF6;M;E;ORF3a;S;N;ORF8;ORF7a;ORF7b;ORF1ab | 1 | circRNA_finder | SRR11550044 |
| SARS-CoV-2_circ_Homo_sapiens_1050 | MN908947.3 | 10161 | 10792 | + | ORF1ab | 1 | circRNA_finder | SRR11550046 |
| SARS-CoV-2_circ_Homo_sapiens_1051 | MN908947.3 | 10161 | 11615 | + | ORF1ab | 1 | circRNA_finder | SRR11550046 |
| SARS-CoV-2_circ_Homo_sapiens_1052 | MN908947.3 | 10161 | 20869 | + | ORF1ab | 1 | circRNA_finder | SRR11550045 |
| SARS-CoV-2_circ_Homo_sapiens_1053 | MN908947.3 | 10161 | 23579 | + | S;ORF1ab | 1 | circRNA_finder | SRR11550045 |
| SARS-CoV-2_circ_Homo_sapiens_1054 | MN908947.3 | 10161 | 26064 | + | ORF3a;S;ORF1ab | 1 | circRNA_finder | SRR11550046 |
| SARS-CoV-2_circ_Homo_sapiens_1055 | MN908947.3 | 10161 | 26154 | + | ORF3a;S;ORF1ab | 1 | circRNA_finder | SRR11550046 |
| SARS-CoV-2_circ_Homo_sapiens_1059 | MN908947.3 | 10197 | 10589 | + | ORF1ab | 1 | circRNA_finder | SRR11550045 |
| SARS-CoV-2_circ_Homo_sapiens_1060 | MN908947.3 | 10314 | 11486 | + | ORF1ab | 1 | circRNA_finder | SRR11550046 |
| SARS-CoV-2_circ_Homo_sapiens_1061 | MN908947.3 | 10314 | 14177 | + | ORF1ab | 1 | circRNA_finder | SRR11550044 |
| SARS-CoV-2_circ_Homo_sapiens_1062 | MN908947.3 | 10319 | 10657 | + | ORF1ab | 1 | circRNA_finder | SRR11550043 |
| SARS-CoV-2_circ_Homo_sapiens_1062 | MN908947.3 | 10319 | 10657 | + | ORF1ab | 1 | circRNA_finder | SRR11550044 |
| SARS-CoV-2_circ_Homo_sapiens_1063 | MN908947.3 | 10319 | 11482 | + | ORF1ab | 1 | circRNA_finder | SRR11550046 |
| SARS-CoV-2_circ_Homo_sapiens_1064 | MN908947.3 | 10325 | 28403 | + | ORF6;M;E;ORF3a;S;N;ORF8;ORF7a;ORF7b;ORF1ab | 1 | circRNA_finder | SRR11550046 |
| SARS-CoV-2_circ_Homo_sapiens_1065 | MN908947.3 | 10346 | 10973 | + | ORF1ab | 1 | circRNA_finder | SRR11550044 |
| SARS-CoV-2_circ_Homo_sapiens_1068 | MN908947.3 | 10350 | 10649 | + | ORF1ab | 1 | circRNA_finder | SRR11550046 |
| SARS-CoV-2_circ_Homo_sapiens_1069 | MN908947.3 | 10350 | 10670 | + | ORF1ab | 1 | circRNA_finder | SRR11550043 |
| SARS-CoV-2_circ_Homo_sapiens_1070 | MN908947.3 | 10350 | 12879 | + | ORF1ab | 1 | circRNA_finder | SRR11550043 |
| SARS-CoV-2_circ_Homo_sapiens_1072 | MN908947.3 | 10401 | 11482 | + | ORF1ab | 1 | circRNA_finder | SRR11550045 |
| SARS-CoV-2_circ_Homo_sapiens_1074 | MN908947.3 | 10508 | 15298 | + | ORF1ab | 1 | circRNA_finder | SRR11550046 |
| SARS-CoV-2_circ_Homo_sapiens_1077 | MN908947.3 | 10566 | 11178 | + | ORF1ab | 1 | circRNA_finder | SRR11550045 |
| SARS-CoV-2_circ_Homo_sapiens_1080 | MN908947.3 | 10595 | 10931 | + | ORF1ab | 1 | circRNA_finder | SRR11550046 |
| SARS-CoV-2_circ_Homo_sapiens_1081 | MN908947.3 | 10595 | 10964 | + | ORF1ab | 1 | circRNA_finder | SRR11550046 |
| SARS-CoV-2_circ_Homo_sapiens_1082 | MN908947.3 | 10595 | 11035 | + | ORF1ab | 1 | circRNA_finder | SRR11550045 |
| SARS-CoV-2_circ_Homo_sapiens_1083 | MN908947.3 | 10595 | 11066 | + | ORF1ab | 1 | circRNA_finder | SRR11550045 |
| SARS-CoV-2_circ_Homo_sapiens_1084 | MN908947.3 | 10595 | 11094 | + | ORF1ab | 1 | circRNA_finder | SRR11550046 |
| SARS-CoV-2_circ_Homo_sapiens_1084 | MN908947.3 | 10595 | 11094 | + | ORF1ab | 1 | circRNA_finder | SRR11550045 |
| SARS-CoV-2_circ_Homo_sapiens_1085 | MN908947.3 | 10595 | 11132 | + | ORF1ab | 1 | circRNA_finder | SRR11550046 |
| SARS-CoV-2_circ_Homo_sapiens_1086 | MN908947.3 | 10595 | 12084 | + | ORF1ab | 1 | circRNA_finder | SRR11550046 |
| SARS-CoV-2_circ_Homo_sapiens_1087 | MN908947.3 | 10595 | 13357 | + | ORF1ab | 1 | circRNA_finder | SRR11550046 |
| SARS-CoV-2_circ_Homo_sapiens_1096 | MN908947.3 | 10605 | 11066 | + | ORF1ab | 1 | circRNA_finder | SRR11550045 |
| SARS-CoV-2_circ_Homo_sapiens_1099 | MN908947.3 | 10605 | 12004 | + | ORF1ab | 1 | circRNA_finder | SRR11550044 |
| SARS-CoV-2_circ_Homo_sapiens_1102 | MN908947.3 | 10605 | 25516 | + | ORF3a;S;ORF1ab | 1 | circRNA_finder | SRR11550046 |
| SARS-CoV-2_circ_Homo_sapiens_1104 | MN908947.3 | 10605 | 26775 | + | M;E;ORF3a;S;ORF1ab | 1 | circRNA_finder | SRR11550046 |
| SARS-CoV-2_circ_Homo_sapiens_1106 | MN908947.3 | 10605 | 26780 | + | M;E;ORF3a;S;ORF1ab | 1 | circRNA_finder | SRR11550045 |
| SARS-CoV-2_circ_Homo_sapiens_1109 | MN908947.3 | 10616 | 14177 | + | ORF1ab | 1 | circRNA_finder | SRR11550044 |
| SARS-CoV-2_circ_Homo_sapiens_1110 | MN908947.3 | 10618 | 27087 | + | M;E;ORF3a;S;ORF1ab | 1 | circRNA_finder | SRR11550043 |
| SARS-CoV-2_circ_Homo_sapiens_1115 | MN908947.3 | 10659 | 11203 | + | ORF1ab | 1 | circRNA_finder | SRR11550046 |
| SARS-CoV-2_circ_Homo_sapiens_1118 | MN908947.3 | 10659 | 12260 | + | ORF1ab | 1 | circRNA_finder | SRR11550045 |
| SARS-CoV-2_circ_Homo_sapiens_1119 | MN908947.3 | 10659 | 13084 | + | ORF1ab | 1 | circRNA_finder | SRR11550045 |
| SARS-CoV-2_circ_Homo_sapiens_1120 | MN908947.3 | 10659 | 13385 | + | ORF1ab | 1 | circRNA_finder | SRR11550045 |
| SARS-CoV-2_circ_Homo_sapiens_1120 | MN908947.3 | 10659 | 13385 | + | ORF1ab | 1 | circRNA_finder | SRR11550043 |
| SARS-CoV-2_circ_Homo_sapiens_1122 | MN908947.3 | 10659 | 25243 | + | S;ORF1ab | 1 | circRNA_finder | SRR11550045 |
| SARS-CoV-2_circ_Homo_sapiens_1123 | MN908947.3 | 10659 | 25980 | + | ORF3a;S;ORF1ab | 1 | circRNA_finder | SRR11550046 |
| SARS-CoV-2_circ_Homo_sapiens_1124 | MN908947.3 | 10659 | 27187 | + | M;E;ORF3a;S;ORF1ab | 1 | circRNA_finder | SRR11550046 |
| SARS-CoV-2_circ_Homo_sapiens_1129 | MN908947.3 | 10671 | 11010 | + | ORF1ab | 1 | circRNA_finder | SRR11550045 |
| SARS-CoV-2_circ_Homo_sapiens_1131 | MN908947.3 | 10671 | 11145 | + | ORF1ab | 1 | circRNA_finder | SRR11550043 |
| SARS-CoV-2_circ_Homo_sapiens_1132 | MN908947.3 | 10671 | 11482 | + | ORF1ab | 1 | circRNA_finder | SRR11550044 |
| SARS-CoV-2_circ_Homo_sapiens_1133 | MN908947.3 | 10671 | 11520 | + | ORF1ab | 1 | circRNA_finder | SRR11550044 |
| SARS-CoV-2_circ_Homo_sapiens_1135 | MN908947.3 | 10671 | 12110 | + | ORF1ab | 1 | circRNA_finder | SRR11550043 |
| SARS-CoV-2_circ_Homo_sapiens_1136 | MN908947.3 | 10671 | 12768 | + | ORF1ab | 1 | circRNA_finder | SRR11550044 |
| SARS-CoV-2_circ_Homo_sapiens_1137 | MN908947.3 | 10671 | 13389 | + | ORF1ab | 1 | circRNA_finder | SRR11550045 |
| SARS-CoV-2_circ_Homo_sapiens_1138 | MN908947.3 | 10671 | 13458 | + | ORF1ab | 1 | circRNA_finder | SRR11550044 |
| SARS-CoV-2_circ_Homo_sapiens_1139 | MN908947.3 | 10671 | 14947 | + | ORF1ab | 1 | circRNA_finder | SRR11550046 |
| SARS-CoV-2_circ_Homo_sapiens_114 | MN908947.3 | 316 | 687 | + | ORF1ab | 1 | circRNA_finder | SRR11550045 |
| SARS-CoV-2_circ_Homo_sapiens_1140 | MN908947.3 | 10671 | 21685 | + | S;ORF1ab | 1 | circRNA_finder | SRR11550044 |
| SARS-CoV-2_circ_Homo_sapiens_1141 | MN908947.3 | 10748 | 11360 | + | ORF1ab | 1 | circRNA_finder | SRR11550044 |
| SARS-CoV-2_circ_Homo_sapiens_1142 | MN908947.3 | 10748 | 27535 | + | ORF6;M;E;ORF3a;S;ORF7a;ORF1ab | 1 | circRNA_finder | SRR11550046 |
| SARS-CoV-2_circ_Homo_sapiens_1145 | MN908947.3 | 10809 | 12131 | + | ORF1ab | 1 | circRNA_finder | SRR11550046 |
| SARS-CoV-2_circ_Homo_sapiens_1146 | MN908947.3 | 10890 | 14177 | + | ORF1ab | 1 | circRNA_finder | SRR11550043 |
| SARS-CoV-2_circ_Homo_sapiens_1149 | MN908947.3 | 10932 | 11480 | + | ORF1ab | 1 | circRNA_finder | SRR11550043 |
| SARS-CoV-2_circ_Homo_sapiens_115 | MN908947.3 | 316 | 836 | + | ORF1ab | 1 | circRNA_finder | SRR11550045 |
| SARS-CoV-2_circ_Homo_sapiens_1150 | MN908947.3 | 10932 | 11579 | + | ORF1ab | 1 | circRNA_finder | SRR11550046 |
| SARS-CoV-2_circ_Homo_sapiens_1151 | MN908947.3 | 10932 | 17830 | + | ORF1ab | 1 | circRNA_finder | SRR11550043 |
| SARS-CoV-2_circ_Homo_sapiens_1153 | MN908947.3 | 10948 | 11286 | + | ORF1ab | 1 | circRNA_finder | SRR11550046 |
| SARS-CoV-2_circ_Homo_sapiens_1154 | MN908947.3 | 10948 | 12056 | + | ORF1ab | 1 | circRNA_finder | SRR11550046 |
| SARS-CoV-2_circ_Homo_sapiens_1155 | MN908947.3 | 10948 | 13389 | + | ORF1ab | 1 | circRNA_finder | SRR11550046 |
| SARS-CoV-2_circ_Homo_sapiens_1156 | MN908947.3 | 10948 | 13935 | + | ORF1ab | 1 | circRNA_finder | SRR11550046 |
| SARS-CoV-2_circ_Homo_sapiens_1158 | MN908947.3 | 11019 | 11299 | + | ORF1ab | 1 | circRNA_finder | SRR11550043 |
| SARS-CoV-2_circ_Homo_sapiens_1160 | MN908947.3 | 11103 | 11480 | + | ORF1ab | 1 | circRNA_finder | SRR11550044 |
| SARS-CoV-2_circ_Homo_sapiens_1161 | MN908947.3 | 11157 | 14644 | + | ORF1ab | 1 | circRNA_finder | SRR11550046 |
| SARS-CoV-2_circ_Homo_sapiens_1162 | MN908947.3 | 11190 | 11513 | + | ORF1ab | 1 | circRNA_finder | SRR11550045 |
| SARS-CoV-2_circ_Homo_sapiens_1164 | MN908947.3 | 11190 | 11843 | + | ORF1ab | 1 | circRNA_finder | SRR11550045 |
| SARS-CoV-2_circ_Homo_sapiens_117 | MN908947.3 | 316 | 24867 | + | S;ORF1ab | 1 | circRNA_finder | SRR11550046 |
| SARS-CoV-2_circ_Homo_sapiens_1170 | MN908947.3 | 11190 | 26780 | + | M;E;ORF3a;S;ORF1ab | 1 | circRNA_finder | SRR11550045 |
| SARS-CoV-2_circ_Homo_sapiens_1171 | MN908947.3 | 11190 | 28686 | + | ORF6;M;E;ORF3a;S;N;ORF8;ORF7a;ORF7b;ORF1ab | 1 | circRNA_finder | SRR11550044 |
| SARS-CoV-2_circ_Homo_sapiens_1174 | MN908947.3 | 11284 | 13458 | + | ORF1ab | 1 | circRNA_finder | SRR11550044 |
| SARS-CoV-2_circ_Homo_sapiens_1176 | MN908947.3 | 11300 | 11905 | + | ORF1ab | 1 | circRNA_finder | SRR11550044 |
| SARS-CoV-2_circ_Homo_sapiens_1178 | MN908947.3 | 11300 | 13385 | + | ORF1ab | 1 | circRNA_finder | SRR11550043 |
| SARS-CoV-2_circ_Homo_sapiens_118 | MN908947.3 | 330 | 2484 | + | ORF1ab | 1 | circRNA_finder | SRR11550046 |
| SARS-CoV-2_circ_Homo_sapiens_1180 | MN908947.3 | 11307 | 12201 | + | ORF1ab | 1 | circRNA_finder | SRR11550044 |
| SARS-CoV-2_circ_Homo_sapiens_1181 | MN908947.3 | 11334 | 13389 | + | ORF1ab | 1 | circRNA_finder | SRR11550046 |
| SARS-CoV-2_circ_Homo_sapiens_1182 | MN908947.3 | 11340 | 11918 | + | ORF1ab | 1 | circRNA_finder | SRR11550043 |
| SARS-CoV-2_circ_Homo_sapiens_1183 | MN908947.3 | 11340 | 11966 | + | ORF1ab | 1 | circRNA_finder | SRR11550046 |
| SARS-CoV-2_circ_Homo_sapiens_1184 | MN908947.3 | 11340 | 26343 | + | ORF1ab;ORF3a;S;E | 1 | circRNA_finder | SRR11550044 |
| SARS-CoV-2_circ_Homo_sapiens_1185 | MN908947.3 | 11362 | 11816 | + | ORF1ab | 1 | circRNA_finder | SRR11550044 |
| SARS-CoV-2_circ_Homo_sapiens_1186 | MN908947.3 | 11488 | 11918 | + | ORF1ab | 1 | circRNA_finder | SRR11550043 |
| SARS-CoV-2_circ_Homo_sapiens_1186 | MN908947.3 | 11488 | 11918 | + | ORF1ab | 1 | circRNA_finder | SRR11550045 |
| SARS-CoV-2_circ_Homo_sapiens_1189 | MN908947.3 | 11488 | 11966 | + | ORF1ab | 1 | circRNA_finder | SRR11550045 |
| SARS-CoV-2_circ_Homo_sapiens_1189 | MN908947.3 | 11488 | 11966 | + | ORF1ab | 1 | circRNA_finder | SRR11550044 |
| SARS-CoV-2_circ_Homo_sapiens_1189 | MN908947.3 | 11488 | 11966 | + | ORF1ab | 1 | circRNA_finder | SRR11550046 |
| SARS-CoV-2_circ_Homo_sapiens_119 | MN908947.3 | 341 | 25548 | + | ORF3a;S;ORF1ab | 1 | circRNA_finder | SRR11550046 |
| SARS-CoV-2_circ_Homo_sapiens_1192 | MN908947.3 | 11488 | 12019 | + | ORF1ab | 1 | circRNA_finder | SRR11550043 |
| SARS-CoV-2_circ_Homo_sapiens_1193 | MN908947.3 | 11488 | 12099 | + | ORF1ab | 1 | circRNA_finder | SRR11550045 |
| SARS-CoV-2_circ_Homo_sapiens_1195 | MN908947.3 | 11488 | 12137 | + | ORF1ab | 1 | circRNA_finder | SRR11550046 |
| SARS-CoV-2_circ_Homo_sapiens_1195 | MN908947.3 | 11488 | 12137 | + | ORF1ab | 1 | circRNA_finder | SRR11550045 |
| SARS-CoV-2_circ_Homo_sapiens_1195 | MN908947.3 | 11488 | 12137 | + | ORF1ab | 1 | circRNA_finder | SRR11550044 |
| SARS-CoV-2_circ_Homo_sapiens_1196 | MN908947.3 | 11488 | 12773 | + | ORF1ab | 1 | circRNA_finder | SRR11550045 |
| SARS-CoV-2_circ_Homo_sapiens_1197 | MN908947.3 | 11488 | 12777 | + | ORF1ab | 1 | circRNA_finder | SRR11550046 |
| SARS-CoV-2_circ_Homo_sapiens_120 | MN908947.3 | 363 | 1835 | + | ORF1ab | 1 | circRNA_finder | SRR11550043 |
| SARS-CoV-2_circ_Homo_sapiens_1202 | MN908947.3 | 11488 | 14572 | + | ORF1ab | 1 | circRNA_finder | SRR11550046 |
| SARS-CoV-2_circ_Homo_sapiens_1204 | MN908947.3 | 11488 | 15852 | + | ORF1ab | 1 | circRNA_finder | SRR11550046 |
| SARS-CoV-2_circ_Homo_sapiens_1209 | MN908947.3 | 11502 | 13312 | + | ORF1ab | 1 | circRNA_finder | SRR11550045 |
| SARS-CoV-2_circ_Homo_sapiens_121 | MN908947.3 | 377 | 3344 | + | ORF1ab | 1 | circRNA_finder | SRR11550045 |
| SARS-CoV-2_circ_Homo_sapiens_1210 | MN908947.3 | 11502 | 13389 | + | ORF1ab | 1 | circRNA_finder | SRR11550045 |
| SARS-CoV-2_circ_Homo_sapiens_1212 | MN908947.3 | 11508 | 12037 | + | ORF1ab | 1 | circRNA_finder | SRR11550043 |
| SARS-CoV-2_circ_Homo_sapiens_1213 | MN908947.3 | 11508 | 13389 | + | ORF1ab | 1 | circRNA_finder | SRR11550045 |
| SARS-CoV-2_circ_Homo_sapiens_1214 | MN908947.3 | 11512 | 11966 | + | ORF1ab | 1 | circRNA_finder | SRR11550045 |
| SARS-CoV-2_circ_Homo_sapiens_1215 | MN908947.3 | 11512 | 14177 | + | ORF1ab | 1 | circRNA_finder | SRR11550045 |
| SARS-CoV-2_circ_Homo_sapiens_1216 | MN908947.3 | 11512 | 14575 | + | ORF1ab | 1 | circRNA_finder | SRR11550043 |
| SARS-CoV-2_circ_Homo_sapiens_1218 | MN908947.3 | 11535 | 11936 | + | ORF1ab | 1 | circRNA_finder | SRR11550046 |
| SARS-CoV-2_circ_Homo_sapiens_1221 | MN908947.3 | 11638 | 11966 | + | ORF1ab | 1 | circRNA_finder | SRR11550045 |
| SARS-CoV-2_circ_Homo_sapiens_1225 | MN908947.3 | 11638 | 12084 | + | ORF1ab | 1 | circRNA_finder | SRR11550045 |
| SARS-CoV-2_circ_Homo_sapiens_1227 | MN908947.3 | 11638 | 12814 | + | ORF1ab | 1 | circRNA_finder | SRR11550045 |
| SARS-CoV-2_circ_Homo_sapiens_1228 | MN908947.3 | 11638 | 23519 | + | S;ORF1ab | 1 | circRNA_finder | SRR11550045 |
| SARS-CoV-2_circ_Homo_sapiens_1229 | MN908947.3 | 11645 | 11961 | + | ORF1ab | 1 | circRNA_finder | SRR11550046 |
| SARS-CoV-2_circ_Homo_sapiens_1232 | MN908947.3 | 11645 | 12084 | + | ORF1ab | 1 | circRNA_finder | SRR11550045 |
| SARS-CoV-2_circ_Homo_sapiens_1233 | MN908947.3 | 11645 | 12099 | + | ORF1ab | 1 | circRNA_finder | SRR11550043 |
| SARS-CoV-2_circ_Homo_sapiens_1234 | MN908947.3 | 11645 | 12115 | + | ORF1ab | 1 | circRNA_finder | SRR11550046 |
| SARS-CoV-2_circ_Homo_sapiens_1235 | MN908947.3 | 11645 | 12131 | + | ORF1ab | 1 | circRNA_finder | SRR11550043 |
| SARS-CoV-2_circ_Homo_sapiens_1237 | MN908947.3 | 11645 | 12137 | + | ORF1ab | 1 | circRNA_finder | SRR11550045 |
| SARS-CoV-2_circ_Homo_sapiens_1238 | MN908947.3 | 11645 | 12341 | + | ORF1ab | 1 | circRNA_finder | SRR11550043 |
| SARS-CoV-2_circ_Homo_sapiens_1244 | MN908947.3 | 11645 | 14177 | + | ORF1ab | 1 | circRNA_finder | SRR11550043 |
| SARS-CoV-2_circ_Homo_sapiens_1247 | MN908947.3 | 11664 | 11975 | + | ORF1ab | 1 | circRNA_finder | SRR11550045 |
| SARS-CoV-2_circ_Homo_sapiens_1249 | MN908947.3 | 11686 | 18355 | + | ORF1ab | 1 | circRNA_finder | SRR11550044 |
| SARS-CoV-2_circ_Homo_sapiens_125 | MN908947.3 | 429 | 2246 | + | ORF1ab | 1 | circRNA_finder | SRR11550043 |
| SARS-CoV-2_circ_Homo_sapiens_1251 | MN908947.3 | 11705 | 11975 | + | ORF1ab | 1 | circRNA_finder | SRR11550045 |
| SARS-CoV-2_circ_Homo_sapiens_1253 | MN908947.3 | 11705 | 12004 | + | ORF1ab | 1 | circRNA_finder | SRR11550046 |
| SARS-CoV-2_circ_Homo_sapiens_1253 | MN908947.3 | 11705 | 12004 | + | ORF1ab | 1 | circRNA_finder | SRR11550044 |
| SARS-CoV-2_circ_Homo_sapiens_1257 | MN908947.3 | 11705 | 12137 | + | ORF1ab | 1 | circRNA_finder | SRR11550043 |
| SARS-CoV-2_circ_Homo_sapiens_1259 | MN908947.3 | 11705 | 12170 | + | ORF1ab | 1 | circRNA_finder | SRR11550045 |
| SARS-CoV-2_circ_Homo_sapiens_126 | MN908947.3 | 429 | 2866 | + | ORF1ab | 1 | circRNA_finder | SRR11550045 |
| SARS-CoV-2_circ_Homo_sapiens_1260 | MN908947.3 | 11705 | 12492 | + | ORF1ab | 1 | circRNA_finder | SRR11550046 |
| SARS-CoV-2_circ_Homo_sapiens_1261 | MN908947.3 | 11705 | 12623 | + | ORF1ab | 1 | circRNA_finder | SRR11550043 |
| SARS-CoV-2_circ_Homo_sapiens_1263 | MN908947.3 | 11705 | 13139 | + | ORF1ab | 1 | circRNA_finder | SRR11550044 |
| SARS-CoV-2_circ_Homo_sapiens_1263 | MN908947.3 | 11705 | 13139 | + | ORF1ab | 1 | circRNA_finder | SRR11550046 |
| SARS-CoV-2_circ_Homo_sapiens_1264 | MN908947.3 | 11705 | 13516 | + | ORF1ab | 1 | circRNA_finder | SRR11550045 |
| SARS-CoV-2_circ_Homo_sapiens_1264 | MN908947.3 | 11705 | 13516 | + | ORF1ab | 1 | circRNA_finder | SRR11550044 |
| SARS-CoV-2_circ_Homo_sapiens_1265 | MN908947.3 | 11705 | 13729 | + | ORF1ab | 1 | circRNA_finder | SRR11550043 |
| SARS-CoV-2_circ_Homo_sapiens_1266 | MN908947.3 | 11705 | 14785 | + | ORF1ab | 1 | circRNA_finder | SRR11550046 |
| SARS-CoV-2_circ_Homo_sapiens_1267 | MN908947.3 | 11705 | 14847 | + | ORF1ab | 1 | circRNA_finder | SRR11550046 |
| SARS-CoV-2_circ_Homo_sapiens_1268 | MN908947.3 | 11705 | 15856 | + | ORF1ab | 1 | circRNA_finder | SRR11550043 |
| SARS-CoV-2_circ_Homo_sapiens_1269 | MN908947.3 | 11709 | 11938 | + | ORF1ab | 1 | circRNA_finder | SRR11550046 |
| SARS-CoV-2_circ_Homo_sapiens_1269 | MN908947.3 | 11709 | 11938 | + | ORF1ab | 1 | circRNA_finder | SRR11550045 |
| SARS-CoV-2_circ_Homo_sapiens_1272 | MN908947.3 | 11709 | 12110 | + | ORF1ab | 1 | circRNA_finder | SRR11550043 |
| SARS-CoV-2_circ_Homo_sapiens_1272 | MN908947.3 | 11709 | 12110 | + | ORF1ab | 1 | circRNA_finder | SRR11550046 |
| SARS-CoV-2_circ_Homo_sapiens_1275 | MN908947.3 | 11709 | 13458 | + | ORF1ab | 1 | circRNA_finder | SRR11550045 |
| SARS-CoV-2_circ_Homo_sapiens_1276 | MN908947.3 | 11709 | 14711 | + | ORF1ab | 1 | circRNA_finder | SRR11550045 |
| SARS-CoV-2_circ_Homo_sapiens_1277 | MN908947.3 | 11709 | 17095 | + | ORF1ab | 1 | circRNA_finder | SRR11550043 |
| SARS-CoV-2_circ_Homo_sapiens_1279 | MN908947.3 | 11817 | 12084 | + | ORF1ab | 1 | circRNA_finder | SRR11550044 |
| SARS-CoV-2_circ_Homo_sapiens_1281 | MN908947.3 | 11817 | 12115 | + | ORF1ab | 1 | circRNA_finder | SRR11550046 |
| SARS-CoV-2_circ_Homo_sapiens_1282 | MN908947.3 | 11817 | 12170 | + | ORF1ab | 1 | circRNA_finder | SRR11550045 |
| SARS-CoV-2_circ_Homo_sapiens_1283 | MN908947.3 | 11817 | 13139 | + | ORF1ab | 1 | circRNA_finder | SRR11550045 |
| SARS-CoV-2_circ_Homo_sapiens_1285 | MN908947.3 | 11817 | 26570 | + | M;E;ORF3a;S;ORF1ab | 1 | circRNA_finder | SRR11550043 |
| SARS-CoV-2_circ_Homo_sapiens_1286 | MN908947.3 | 11817 | 28895 | + | ORF6;M;E;ORF3a;S;N;ORF8;ORF7a;ORF7b;ORF1ab | 1 | circRNA_finder | SRR11550043 |
| SARS-CoV-2_circ_Homo_sapiens_1287 | MN908947.3 | 11877 | 13389 | + | ORF1ab | 1 | circRNA_finder | SRR11550043 |
| SARS-CoV-2_circ_Homo_sapiens_1288 | MN908947.3 | 11877 | 24276 | + | S;ORF1ab | 1 | circRNA_finder | SRR11550044 |
| SARS-CoV-2_circ_Homo_sapiens_1295 | MN908947.3 | 12009 | 13004 | + | ORF1ab | 1 | circRNA_finder | SRR11550044 |
| SARS-CoV-2_circ_Homo_sapiens_1296 | MN908947.3 | 12044 | 12287 | + | ORF1ab | 1 | circRNA_finder | SRR11550045 |
| SARS-CoV-2_circ_Homo_sapiens_1297 | MN908947.3 | 12044 | 12737 | + | ORF1ab | 1 | circRNA_finder | SRR11550046 |
| SARS-CoV-2_circ_Homo_sapiens_130 | MN908947.3 | 527 | 995 | + | ORF1ab | 1 | circRNA_finder | SRR11550044 |
| SARS-CoV-2_circ_Homo_sapiens_1300 | MN908947.3 | 12044 | 27130 | + | M;E;ORF3a;S;ORF1ab | 1 | circRNA_finder | SRR11550044 |
| SARS-CoV-2_circ_Homo_sapiens_1302 | MN908947.3 | 12053 | 13725 | + | ORF1ab | 1 | circRNA_finder | SRR11550045 |
| SARS-CoV-2_circ_Homo_sapiens_1306 | MN908947.3 | 12085 | 12341 | + | ORF1ab | 1 | circRNA_finder | SRR11550045 |
| SARS-CoV-2_circ_Homo_sapiens_1307 | MN908947.3 | 12085 | 12438 | + | ORF1ab | 1 | circRNA_finder | SRR11550045 |
| SARS-CoV-2_circ_Homo_sapiens_1308 | MN908947.3 | 12085 | 13139 | + | ORF1ab | 1 | circRNA_finder | SRR11550045 |
| SARS-CoV-2_circ_Homo_sapiens_1308 | MN908947.3 | 12085 | 13139 | + | ORF1ab | 1 | circRNA_finder | SRR11550043 |
| SARS-CoV-2_circ_Homo_sapiens_1309 | MN908947.3 | 12085 | 13357 | + | ORF1ab | 1 | circRNA_finder | SRR11550046 |
| SARS-CoV-2_circ_Homo_sapiens_131 | MN908947.3 | 527 | 1232 | + | ORF1ab | 1 | circRNA_finder | SRR11550045 |
| SARS-CoV-2_circ_Homo_sapiens_1310 | MN908947.3 | 12085 | 14146 | + | ORF1ab | 1 | circRNA_finder | SRR11550046 |
| SARS-CoV-2_circ_Homo_sapiens_1311 | MN908947.3 | 12085 | 14575 | + | ORF1ab | 1 | circRNA_finder | SRR11550044 |
| SARS-CoV-2_circ_Homo_sapiens_1313 | MN908947.3 | 12085 | 14876 | + | ORF1ab | 1 | circRNA_finder | SRR11550045 |
| SARS-CoV-2_circ_Homo_sapiens_1314 | MN908947.3 | 12085 | 15018 | + | ORF1ab | 1 | circRNA_finder | SRR11550046 |
| SARS-CoV-2_circ_Homo_sapiens_1315 | MN908947.3 | 12085 | 15279 | + | ORF1ab | 1 | circRNA_finder | SRR11550044 |
| SARS-CoV-2_circ_Homo_sapiens_1317 | MN908947.3 | 12085 | 23225 | + | S;ORF1ab | 1 | circRNA_finder | SRR11550045 |
| SARS-CoV-2_circ_Homo_sapiens_1318 | MN908947.3 | 12085 | 25602 | + | ORF3a;S;ORF1ab | 1 | circRNA_finder | SRR11550044 |
| SARS-CoV-2_circ_Homo_sapiens_1319 | MN908947.3 | 12090 | 12694 | + | ORF1ab | 1 | circRNA_finder | SRR11550043 |
| SARS-CoV-2_circ_Homo_sapiens_132 | MN908947.3 | 527 | 1244 | + | ORF1ab | 1 | circRNA_finder | SRR11550046 |
| SARS-CoV-2_circ_Homo_sapiens_132 | MN908947.3 | 527 | 1244 | + | ORF1ab | 1 | circRNA_finder | SRR11550043 |
| SARS-CoV-2_circ_Homo_sapiens_1320 | MN908947.3 | 12090 | 12773 | + | ORF1ab | 1 | circRNA_finder | SRR11550046 |
| SARS-CoV-2_circ_Homo_sapiens_1321 | MN908947.3 | 12093 | 12797 | + | ORF1ab | 1 | circRNA_finder | SRR11550045 |
| SARS-CoV-2_circ_Homo_sapiens_1322 | MN908947.3 | 12093 | 13385 | + | ORF1ab | 1 | circRNA_finder | SRR11550046 |
| SARS-CoV-2_circ_Homo_sapiens_1324 | MN908947.3 | 12093 | 17063 | + | ORF1ab | 1 | circRNA_finder | SRR11550046 |
| SARS-CoV-2_circ_Homo_sapiens_1325 | MN908947.3 | 12093 | 27187 | + | M;E;ORF3a;S;ORF1ab | 1 | circRNA_finder | SRR11550043 |
| SARS-CoV-2_circ_Homo_sapiens_1326 | MN908947.3 | 12107 | 14862 | + | ORF1ab | 1 | circRNA_finder | SRR11550045 |
| SARS-CoV-2_circ_Homo_sapiens_1327 | MN908947.3 | 12112 | 14413 | + | ORF1ab | 1 | circRNA_finder | SRR11550046 |
| SARS-CoV-2_circ_Homo_sapiens_1328 | MN908947.3 | 12112 | 15918 | + | ORF1ab | 1 | circRNA_finder | SRR11550045 |
| SARS-CoV-2_circ_Homo_sapiens_133 | MN908947.3 | 527 | 1337 | + | ORF1ab | 1 | circRNA_finder | SRR11550043 |
| SARS-CoV-2_circ_Homo_sapiens_133 | MN908947.3 | 527 | 1337 | + | ORF1ab | 1 | circRNA_finder | SRR11550044 |
| SARS-CoV-2_circ_Homo_sapiens_1330 | MN908947.3 | 12132 | 12768 | + | ORF1ab | 1 | circRNA_finder | SRR11550043 |
| SARS-CoV-2_circ_Homo_sapiens_1331 | MN908947.3 | 12132 | 12801 | + | ORF1ab | 1 | circRNA_finder | SRR11550045 |
| SARS-CoV-2_circ_Homo_sapiens_1334 | MN908947.3 | 12132 | 13458 | + | ORF1ab | 1 | circRNA_finder | SRR11550044 |
| SARS-CoV-2_circ_Homo_sapiens_1334 | MN908947.3 | 12132 | 13458 | + | ORF1ab | 1 | circRNA_finder | SRR11550046 |
| SARS-CoV-2_circ_Homo_sapiens_1337 | MN908947.3 | 12132 | 14862 | + | ORF1ab | 1 | circRNA_finder | SRR11550045 |
| SARS-CoV-2_circ_Homo_sapiens_1338 | MN908947.3 | 12132 | 15242 | + | ORF1ab | 1 | circRNA_finder | SRR11550045 |
| SARS-CoV-2_circ_Homo_sapiens_1339 | MN908947.3 | 12132 | 18390 | + | ORF1ab | 1 | circRNA_finder | SRR11550045 |
| SARS-CoV-2_circ_Homo_sapiens_134 | MN908947.3 | 527 | 1492 | + | ORF1ab | 1 | circRNA_finder | SRR11550044 |
| SARS-CoV-2_circ_Homo_sapiens_1340 | MN908947.3 | 12132 | 21667 | + | S;ORF1ab | 1 | circRNA_finder | SRR11550044 |
| SARS-CoV-2_circ_Homo_sapiens_1342 | MN908947.3 | 12150 | 12805 | + | ORF1ab | 1 | circRNA_finder | SRR11550045 |
| SARS-CoV-2_circ_Homo_sapiens_1345 | MN908947.3 | 12153 | 12768 | + | ORF1ab | 1 | circRNA_finder | SRR11550046 |
| SARS-CoV-2_circ_Homo_sapiens_1346 | MN908947.3 | 12153 | 12801 | + | ORF1ab | 1 | circRNA_finder | SRR11550044 |
| SARS-CoV-2_circ_Homo_sapiens_1346 | MN908947.3 | 12153 | 12801 | + | ORF1ab | 1 | circRNA_finder | SRR11550043 |
| SARS-CoV-2_circ_Homo_sapiens_1347 | MN908947.3 | 12153 | 12816 | + | ORF1ab | 1 | circRNA_finder | SRR11550046 |
| SARS-CoV-2_circ_Homo_sapiens_1347 | MN908947.3 | 12153 | 12816 | + | ORF1ab | 1 | circRNA_finder | SRR11550045 |
| SARS-CoV-2_circ_Homo_sapiens_1348 | MN908947.3 | 12153 | 12910 | + | ORF1ab | 1 | circRNA_finder | SRR11550043 |
| SARS-CoV-2_circ_Homo_sapiens_135 | MN908947.3 | 527 | 1534 | + | ORF1ab | 1 | circRNA_finder | SRR11550044 |
| SARS-CoV-2_circ_Homo_sapiens_1352 | MN908947.3 | 12153 | 13458 | + | ORF1ab | 1 | circRNA_finder | SRR11550043 |
| SARS-CoV-2_circ_Homo_sapiens_1354 | MN908947.3 | 12153 | 14413 | + | ORF1ab | 1 | circRNA_finder | SRR11550045 |
| SARS-CoV-2_circ_Homo_sapiens_1357 | MN908947.3 | 12153 | 15242 | + | ORF1ab | 1 | circRNA_finder | SRR11550046 |
| SARS-CoV-2_circ_Homo_sapiens_1357 | MN908947.3 | 12153 | 15242 | + | ORF1ab | 1 | circRNA_finder | SRR11550043 |
| SARS-CoV-2_circ_Homo_sapiens_1363 | MN908947.3 | 12189 | 13458 | + | ORF1ab | 1 | circRNA_finder | SRR11550043 |
| SARS-CoV-2_circ_Homo_sapiens_1364 | MN908947.3 | 12189 | 14719 | + | ORF1ab | 1 | circRNA_finder | SRR11550046 |
| SARS-CoV-2_circ_Homo_sapiens_1365 | MN908947.3 | 12203 | 13412 | + | ORF1ab | 1 | circRNA_finder | SRR11550043 |
| SARS-CoV-2_circ_Homo_sapiens_1367 | MN908947.3 | 12203 | 14208 | + | ORF1ab | 1 | circRNA_finder | SRR11550045 |
| SARS-CoV-2_circ_Homo_sapiens_1369 | MN908947.3 | 12209 | 12478 | + | ORF1ab | 1 | circRNA_finder | SRR11550046 |
| SARS-CoV-2_circ_Homo_sapiens_137 | MN908947.3 | 527 | 2515 | + | ORF1ab | 1 | circRNA_finder | SRR11550046 |
| SARS-CoV-2_circ_Homo_sapiens_1370 | MN908947.3 | 12209 | 14719 | + | ORF1ab | 1 | circRNA_finder | SRR11550045 |
| SARS-CoV-2_circ_Homo_sapiens_1375 | MN908947.3 | 12261 | 13139 | + | ORF1ab | 1 | circRNA_finder | SRR11550044 |
| SARS-CoV-2_circ_Homo_sapiens_1377 | MN908947.3 | 12261 | 28727 | + | ORF6;M;E;ORF3a;S;N;ORF8;ORF7a;ORF7b;ORF1ab | 1 | circRNA_finder | SRR11550046 |
| SARS-CoV-2_circ_Homo_sapiens_138 | MN908947.3 | 527 | 2761 | + | ORF1ab | 1 | circRNA_finder | SRR11550046 |
| SARS-CoV-2_circ_Homo_sapiens_1382 | MN908947.3 | 12288 | 12797 | + | ORF1ab | 1 | circRNA_finder | SRR11550045 |
| SARS-CoV-2_circ_Homo_sapiens_1386 | MN908947.3 | 12288 | 15264 | + | ORF1ab | 1 | circRNA_finder | SRR11550045 |
| SARS-CoV-2_circ_Homo_sapiens_1387 | MN908947.3 | 12288 | 15574 | + | ORF1ab | 1 | circRNA_finder | SRR11550046 |
| SARS-CoV-2_circ_Homo_sapiens_1390 | MN908947.3 | 12295 | 15298 | + | ORF1ab | 1 | circRNA_finder | SRR11550045 |
| SARS-CoV-2_circ_Homo_sapiens_1392 | MN908947.3 | 12309 | 12777 | + | ORF1ab | 1 | circRNA_finder | SRR11550046 |
| SARS-CoV-2_circ_Homo_sapiens_1395 | MN908947.3 | 12311 | 12768 | + | ORF1ab | 1 | circRNA_finder | SRR11550045 |
| SARS-CoV-2_circ_Homo_sapiens_1396 | MN908947.3 | 12311 | 13470 | + | ORF1ab | 1 | circRNA_finder | SRR11550046 |
| SARS-CoV-2_circ_Homo_sapiens_1397 | MN908947.3 | 12316 | 12768 | + | ORF1ab | 1 | circRNA_finder | SRR11550045 |
| SARS-CoV-2_circ_Homo_sapiens_1398 | MN908947.3 | 12339 | 13373 | + | ORF1ab | 1 | circRNA_finder | SRR11550044 |
| SARS-CoV-2_circ_Homo_sapiens_140 | MN908947.3 | 527 | 3353 | + | ORF1ab | 1 | circRNA_finder | SRR11550043 |
| SARS-CoV-2_circ_Homo_sapiens_1400 | MN908947.3 | 12343 | 12773 | + | ORF1ab | 1 | circRNA_finder | SRR11550045 |
| SARS-CoV-2_circ_Homo_sapiens_1405 | MN908947.3 | 12502 | 13139 | + | ORF1ab | 1 | circRNA_finder | SRR11550043 |
| SARS-CoV-2_circ_Homo_sapiens_1405 | MN908947.3 | 12502 | 13139 | + | ORF1ab | 1 | circRNA_finder | SRR11550044 |
| SARS-CoV-2_circ_Homo_sapiens_1406 | MN908947.3 | 12502 | 14167 | + | ORF1ab | 1 | circRNA_finder | SRR11550045 |
| SARS-CoV-2_circ_Homo_sapiens_1409 | MN908947.3 | 12535 | 13067 | + | ORF1ab | 1 | circRNA_finder | SRR11550044 |
| SARS-CoV-2_circ_Homo_sapiens_141 | MN908947.3 | 527 | 4972 | + | ORF1ab | 1 | circRNA_finder | SRR11550046 |
| SARS-CoV-2_circ_Homo_sapiens_1410 | MN908947.3 | 12597 | 13139 | + | ORF1ab | 1 | circRNA_finder | SRR11550043 |
| SARS-CoV-2_circ_Homo_sapiens_1411 | MN908947.3 | 12601 | 15037 | + | ORF1ab | 1 | circRNA_finder | SRR11550046 |
| SARS-CoV-2_circ_Homo_sapiens_1414 | MN908947.3 | 12633 | 12937 | + | ORF1ab | 1 | circRNA_finder | SRR11550045 |
| SARS-CoV-2_circ_Homo_sapiens_1415 | MN908947.3 | 12654 | 13304 | + | ORF1ab | 1 | circRNA_finder | SRR11550043 |
| SARS-CoV-2_circ_Homo_sapiens_1416 | MN908947.3 | 12654 | 13389 | + | ORF1ab | 1 | circRNA_finder | SRR11550045 |
| SARS-CoV-2_circ_Homo_sapiens_1417 | MN908947.3 | 12654 | 13458 | + | ORF1ab | 1 | circRNA_finder | SRR11550043 |
| SARS-CoV-2_circ_Homo_sapiens_1418 | MN908947.3 | 12654 | 14947 | + | ORF1ab | 1 | circRNA_finder | SRR11550043 |
| SARS-CoV-2_circ_Homo_sapiens_142 | MN908947.3 | 527 | 5633 | + | ORF1ab | 1 | circRNA_finder | SRR11550045 |
| SARS-CoV-2_circ_Homo_sapiens_1420 | MN908947.3 | 12695 | 13458 | + | ORF1ab | 1 | circRNA_finder | SRR11550043 |
| SARS-CoV-2_circ_Homo_sapiens_1421 | MN908947.3 | 12700 | 12937 | + | ORF1ab | 1 | circRNA_finder | SRR11550046 |
| SARS-CoV-2_circ_Homo_sapiens_1422 | MN908947.3 | 12774 | 13312 | + | ORF1ab | 1 | circRNA_finder | SRR11550045 |
| SARS-CoV-2_circ_Homo_sapiens_1423 | MN908947.3 | 12774 | 13389 | + | ORF1ab | 1 | circRNA_finder | SRR11550045 |
| SARS-CoV-2_circ_Homo_sapiens_1425 | MN908947.3 | 12794 | 13312 | + | ORF1ab | 1 | circRNA_finder | SRR11550046 |
| SARS-CoV-2_circ_Homo_sapiens_1426 | MN908947.3 | 12802 | 14652 | + | ORF1ab | 1 | circRNA_finder | SRR11550046 |
| SARS-CoV-2_circ_Homo_sapiens_1427 | MN908947.3 | 12831 | 13567 | + | ORF1ab | 1 | circRNA_finder | SRR11550045 |
| SARS-CoV-2_circ_Homo_sapiens_1429 | MN908947.3 | 12831 | 14177 | + | ORF1ab | 1 | circRNA_finder | SRR11550046 |
| SARS-CoV-2_circ_Homo_sapiens_1433 | MN908947.3 | 12831 | 16798 | + | ORF1ab | 1 | circRNA_finder | SRR11550044 |
| SARS-CoV-2_circ_Homo_sapiens_1435 | MN908947.3 | 12833 | 13304 | + | ORF1ab | 1 | circRNA_finder | SRR11550043 |
| SARS-CoV-2_circ_Homo_sapiens_1437 | MN908947.3 | 12850 | 13292 | + | ORF1ab | 1 | circRNA_finder | SRR11550046 |
| SARS-CoV-2_circ_Homo_sapiens_1438 | MN908947.3 | 12850 | 16312 | + | ORF1ab | 1 | circRNA_finder | SRR11550045 |
| SARS-CoV-2_circ_Homo_sapiens_1439 | MN908947.3 | 12860 | 13389 | + | ORF1ab | 1 | circRNA_finder | SRR11550045 |
| SARS-CoV-2_circ_Homo_sapiens_1440 | MN908947.3 | 12862 | 13292 | + | ORF1ab | 1 | circRNA_finder | SRR11550048 |
| SARS-CoV-2_circ_Homo_sapiens_1441 | MN908947.3 | 12886 | 13445 | + | ORF1ab | 1 | circRNA_finder | SRR11550046 |
| SARS-CoV-2_circ_Homo_sapiens_1442 | MN908947.3 | 12900 | 13435 | + | ORF1ab | 1 | circRNA_finder | SRR11550044 |
| SARS-CoV-2_circ_Homo_sapiens_1443 | MN908947.3 | 12900 | 14847 | + | ORF1ab | 1 | circRNA_finder | SRR11550046 |
| SARS-CoV-2_circ_Homo_sapiens_1444 | MN908947.3 | 12924 | 13296 | + | ORF1ab | 1 | circRNA_finder | SRR11550043 |
| SARS-CoV-2_circ_Homo_sapiens_1449 | MN908947.3 | 12939 | 15304 | + | ORF1ab | 1 | circRNA_finder | SRR11550043 |
| SARS-CoV-2_circ_Homo_sapiens_145 | MN908947.3 | 527 | 19299 | + | ORF1ab | 1 | circRNA_finder | SRR11550044 |
| SARS-CoV-2_circ_Homo_sapiens_1452 | MN908947.3 | 12953 | 25713 | + | ORF3a;S;ORF1ab | 1 | circRNA_finder | SRR11550046 |
| SARS-CoV-2_circ_Homo_sapiens_1454 | MN908947.3 | 12974 | 13334 | + | ORF1ab | 1 | circRNA_finder | SRR11550045 |
| SARS-CoV-2_circ_Homo_sapiens_1455 | MN908947.3 | 12974 | 13357 | + | ORF1ab | 1 | circRNA_finder | SRR11550045 |
| SARS-CoV-2_circ_Homo_sapiens_1457 | MN908947.3 | 12974 | 13823 | + | ORF1ab | 1 | circRNA_finder | SRR11550046 |
| SARS-CoV-2_circ_Homo_sapiens_146 | MN908947.3 | 527 | 26246 | + | ORF1ab;ORF3a;S;E | 1 | circRNA_finder | SRR11550045 |
| SARS-CoV-2_circ_Homo_sapiens_1460 | MN908947.3 | 12974 | 14575 | + | ORF1ab | 1 | circRNA_finder | SRR11550044 |
| SARS-CoV-2_circ_Homo_sapiens_1462 | MN908947.3 | 12974 | 14598 | + | ORF1ab | 1 | circRNA_finder | SRR11550046 |
| SARS-CoV-2_circ_Homo_sapiens_1465 | MN908947.3 | 12974 | 15856 | + | ORF1ab | 1 | circRNA_finder | SRR11550043 |
| SARS-CoV-2_circ_Homo_sapiens_1466 | MN908947.3 | 12974 | 17899 | + | ORF1ab | 1 | circRNA_finder | SRR11550045 |
| SARS-CoV-2_circ_Homo_sapiens_1468 | MN908947.3 | 12982 | 14240 | + | ORF1ab | 1 | circRNA_finder | SRR11550044 |
| SARS-CoV-2_circ_Homo_sapiens_1469 | MN908947.3 | 12984 | 13345 | + | ORF1ab | 1 | circRNA_finder | SRR11550046 |
| SARS-CoV-2_circ_Homo_sapiens_147 | MN908947.3 | 534 | 1273 | + | ORF1ab | 1 | circRNA_finder | SRR11550046 |
| SARS-CoV-2_circ_Homo_sapiens_1474 | MN908947.3 | 12984 | 15242 | + | ORF1ab | 1 | circRNA_finder | SRR11550045 |
| SARS-CoV-2_circ_Homo_sapiens_1476 | MN908947.3 | 12984 | 29746 | + | ORF6;ORF10;M;E;ORF3a;S;N;ORF8;ORF7a;ORF7b;ORF1ab | 1 | circRNA_finder | SRR11550043 |
| SARS-CoV-2_circ_Homo_sapiens_1479 | MN908947.3 | 13000 | 13389 | + | ORF1ab | 1 | circRNA_finder | SRR11550046 |
| SARS-CoV-2_circ_Homo_sapiens_148 | MN908947.3 | 534 | 1574 | + | ORF1ab | 1 | circRNA_finder | SRR11550045 |
| SARS-CoV-2_circ_Homo_sapiens_1480 | MN908947.3 | 13000 | 13397 | + | ORF1ab | 1 | circRNA_finder | SRR11550045 |
| SARS-CoV-2_circ_Homo_sapiens_1483 | MN908947.3 | 13005 | 13397 | + | ORF1ab | 1 | circRNA_finder | SRR11550043 |
| SARS-CoV-2_circ_Homo_sapiens_1484 | MN908947.3 | 13005 | 14240 | + | ORF1ab | 1 | circRNA_finder | SRR11550045 |
| SARS-CoV-2_circ_Homo_sapiens_1485 | MN908947.3 | 13005 | 14499 | + | ORF1ab | 1 | circRNA_finder | SRR11550045 |
| SARS-CoV-2_circ_Homo_sapiens_1486 | MN908947.3 | 13017 | 17162 | + | ORF1ab | 1 | circRNA_finder | SRR11550045 |
| SARS-CoV-2_circ_Homo_sapiens_1487 | MN908947.3 | 13017 | 29481 | + | ORF6;M;E;ORF3a;S;N;ORF8;ORF7a;ORF7b;ORF1ab | 1 | circRNA_finder | SRR11550044 |
| SARS-CoV-2_circ_Homo_sapiens_1488 | MN908947.3 | 13023 | 13729 | + | ORF1ab | 1 | circRNA_finder | SRR11550045 |
| SARS-CoV-2_circ_Homo_sapiens_149 | MN908947.3 | 572 | 25620 | + | ORF3a;S;ORF1ab | 1 | circRNA_finder | SRR11550045 |
| SARS-CoV-2_circ_Homo_sapiens_1490 | MN908947.3 | 13023 | 18400 | + | ORF1ab | 1 | circRNA_finder | SRR11550045 |
| SARS-CoV-2_circ_Homo_sapiens_1491 | MN908947.3 | 13089 | 13389 | + | ORF1ab | 1 | circRNA_finder | SRR11550043 |
| SARS-CoV-2_circ_Homo_sapiens_1492 | MN908947.3 | 13089 | 13458 | + | ORF1ab | 1 | circRNA_finder | SRR11550043 |
| SARS-CoV-2_circ_Homo_sapiens_1493 | MN908947.3 | 13089 | 14240 | + | ORF1ab | 1 | circRNA_finder | SRR11550046 |
| SARS-CoV-2_circ_Homo_sapiens_1494 | MN908947.3 | 13089 | 14539 | + | ORF1ab | 1 | circRNA_finder | SRR11550046 |
| SARS-CoV-2_circ_Homo_sapiens_1495 | MN908947.3 | 13089 | 16297 | + | ORF1ab | 1 | circRNA_finder | SRR11550045 |
| SARS-CoV-2_circ_Homo_sapiens_1496 | MN908947.3 | 13119 | 24925 | + | S;ORF1ab | 1 | circRNA_finder | SRR11550044 |
| SARS-CoV-2_circ_Homo_sapiens_1502 | MN908947.3 | 13154 | 17922 | + | ORF1ab | 1 | circRNA_finder | SRR11550044 |
| SARS-CoV-2_circ_Homo_sapiens_1506 | MN908947.3 | 13265 | 14598 | + | ORF1ab | 1 | circRNA_finder | SRR11550045 |
| SARS-CoV-2_circ_Homo_sapiens_1507 | MN908947.3 | 13287 | 26157 | + | ORF3a;S;ORF1ab | 1 | circRNA_finder | SRR11550046 |
| SARS-CoV-2_circ_Homo_sapiens_1508 | MN908947.3 | 13298 | 14575 | + | ORF1ab | 1 | circRNA_finder | SRR11550044 |
| SARS-CoV-2_circ_Homo_sapiens_1509 | MN908947.3 | 13359 | 14598 | + | ORF1ab | 1 | circRNA_finder | SRR11550046 |
| SARS-CoV-2_circ_Homo_sapiens_1510 | MN908947.3 | 13398 | 15242 | + | ORF1ab | 1 | circRNA_finder | SRR11550045 |
| SARS-CoV-2_circ_Homo_sapiens_1511 | MN908947.3 | 13431 | 14572 | + | ORF1ab | 1 | circRNA_finder | SRR11550045 |
| SARS-CoV-2_circ_Homo_sapiens_1513 | MN908947.3 | 13485 | 14593 | + | ORF1ab | 1 | circRNA_finder | SRR11550045 |
| SARS-CoV-2_circ_Homo_sapiens_1515 | MN908947.3 | 13500 | 14177 | + | ORF1ab | 1 | circRNA_finder | SRR11550045 |
| SARS-CoV-2_circ_Homo_sapiens_1519 | MN908947.3 | 13500 | 14598 | + | ORF1ab | 1 | circRNA_finder | SRR11550045 |
| SARS-CoV-2_circ_Homo_sapiens_152 | MN908947.3 | 689 | 1463 | + | ORF1ab | 1 | circRNA_finder | SRR11550043 |
| SARS-CoV-2_circ_Homo_sapiens_1520 | MN908947.3 | 13500 | 14713 | + | ORF1ab | 1 | circRNA_finder | SRR11550045 |
| SARS-CoV-2_circ_Homo_sapiens_1522 | MN908947.3 | 13500 | 16690 | + | ORF1ab | 1 | circRNA_finder | SRR11550043 |
| SARS-CoV-2_circ_Homo_sapiens_1523 | MN908947.3 | 13500 | 17009 | + | ORF1ab | 1 | circRNA_finder | SRR11550045 |
| SARS-CoV-2_circ_Homo_sapiens_1524 | MN908947.3 | 13500 | 17162 | + | ORF1ab | 1 | circRNA_finder | SRR11550046 |
| SARS-CoV-2_circ_Homo_sapiens_1526 | MN908947.3 | 13500 | 18355 | + | ORF1ab | 1 | circRNA_finder | SRR11550043 |
| SARS-CoV-2_circ_Homo_sapiens_1527 | MN908947.3 | 13500 | 25748 | + | ORF3a;S;ORF1ab | 1 | circRNA_finder | SRR11550045 |
| SARS-CoV-2_circ_Homo_sapiens_1528 | MN908947.3 | 13500 | 27102 | + | M;E;ORF3a;S;ORF1ab | 1 | circRNA_finder | SRR11550044 |
| SARS-CoV-2_circ_Homo_sapiens_1529 | MN908947.3 | 13502 | 14598 | + | ORF1ab | 1 | circRNA_finder | SRR11550045 |
| SARS-CoV-2_circ_Homo_sapiens_1535 | MN908947.3 | 13521 | 13935 | + | ORF1ab | 1 | circRNA_finder | SRR11550045 |
| SARS-CoV-2_circ_Homo_sapiens_1537 | MN908947.3 | 13521 | 14122 | + | ORF1ab | 1 | circRNA_finder | SRR11550044 |
| SARS-CoV-2_circ_Homo_sapiens_1538 | MN908947.3 | 13521 | 14460 | + | ORF1ab | 1 | circRNA_finder | SRR11550043 |
| SARS-CoV-2_circ_Homo_sapiens_1539 | MN908947.3 | 13521 | 14853 | + | ORF1ab | 1 | circRNA_finder | SRR11550045 |
| SARS-CoV-2_circ_Homo_sapiens_154 | MN908947.3 | 689 | 1598 | + | ORF1ab | 1 | circRNA_finder | SRR11550046 |
| SARS-CoV-2_circ_Homo_sapiens_1541 | MN908947.3 | 13521 | 16312 | + | ORF1ab | 1 | circRNA_finder | SRR11550044 |
| SARS-CoV-2_circ_Homo_sapiens_1543 | MN908947.3 | 13521 | 16942 | + | ORF1ab | 1 | circRNA_finder | SRR11550043 |
| SARS-CoV-2_circ_Homo_sapiens_1543 | MN908947.3 | 13521 | 16942 | + | ORF1ab | 1 | circRNA_finder | SRR11550045 |
| SARS-CoV-2_circ_Homo_sapiens_1544 | MN908947.3 | 13521 | 19295 | + | ORF1ab | 1 | circRNA_finder | SRR11550046 |
| SARS-CoV-2_circ_Homo_sapiens_1546 | MN908947.3 | 13648 | 13993 | + | ORF1ab | 1 | circRNA_finder | SRR11550046 |
| SARS-CoV-2_circ_Homo_sapiens_1551 | MN908947.3 | 13736 | 14283 | + | ORF1ab | 1 | circRNA_finder | SRR11550044 |
| SARS-CoV-2_circ_Homo_sapiens_1551 | MN908947.3 | 13736 | 14283 | + | ORF1ab | 1 | circRNA_finder | SRR11550046 |
| SARS-CoV-2_circ_Homo_sapiens_1553 | MN908947.3 | 13836 | 14556 | + | ORF1ab | 1 | circRNA_finder | SRR11550046 |
| SARS-CoV-2_circ_Homo_sapiens_1554 | MN908947.3 | 13850 | 15969 | + | ORF1ab | 1 | circRNA_finder | SRR11550043 |
| SARS-CoV-2_circ_Homo_sapiens_1556 | MN908947.3 | 13949 | 15198 | + | ORF1ab | 1 | circRNA_finder | SRR11550045 |
| SARS-CoV-2_circ_Homo_sapiens_1557 | MN908947.3 | 13972 | 14407 | + | ORF1ab | 1 | circRNA_finder | SRR11550046 |
| SARS-CoV-2_circ_Homo_sapiens_1558 | MN908947.3 | 13972 | 14530 | + | ORF1ab | 1 | circRNA_finder | SRR11550044 |
| SARS-CoV-2_circ_Homo_sapiens_1560 | MN908947.3 | 13972 | 14572 | + | ORF1ab | 1 | circRNA_finder | SRR11550045 |
| SARS-CoV-2_circ_Homo_sapiens_1563 | MN908947.3 | 13972 | 14598 | + | ORF1ab | 1 | circRNA_finder | SRR11550046 |
| SARS-CoV-2_circ_Homo_sapiens_1565 | MN908947.3 | 13972 | 14644 | + | ORF1ab | 1 | circRNA_finder | SRR11550044 |
| SARS-CoV-2_circ_Homo_sapiens_1566 | MN908947.3 | 13972 | 25924 | + | ORF3a;S;ORF1ab | 1 | circRNA_finder | SRR11550046 |
| SARS-CoV-2_circ_Homo_sapiens_1567 | MN908947.3 | 13976 | 14460 | + | ORF1ab | 1 | circRNA_finder | SRR11550043 |
| SARS-CoV-2_circ_Homo_sapiens_1568 | MN908947.3 | 13976 | 14514 | + | ORF1ab | 1 | circRNA_finder | SRR11550043 |
| SARS-CoV-2_circ_Homo_sapiens_157 | MN908947.3 | 689 | 4520 | + | ORF1ab | 1 | circRNA_finder | SRR11550045 |
| SARS-CoV-2_circ_Homo_sapiens_1571 | MN908947.3 | 13976 | 26851 | + | M;E;ORF3a;S;ORF1ab | 1 | circRNA_finder | SRR11550045 |
| SARS-CoV-2_circ_Homo_sapiens_1572 | MN908947.3 | 14127 | 14539 | + | ORF1ab | 1 | circRNA_finder | SRR11550045 |
| SARS-CoV-2_circ_Homo_sapiens_1573 | MN908947.3 | 14178 | 14490 | + | ORF1ab | 1 | circRNA_finder | SRR11550046 |
| SARS-CoV-2_circ_Homo_sapiens_1575 | MN908947.3 | 14178 | 14535 | + | ORF1ab | 1 | circRNA_finder | SRR11550044 |
| SARS-CoV-2_circ_Homo_sapiens_1576 | MN908947.3 | 14178 | 14553 | + | ORF1ab | 1 | circRNA_finder | SRR11550044 |
| SARS-CoV-2_circ_Homo_sapiens_158 | MN908947.3 | 689 | 10663 | + | ORF1ab | 1 | circRNA_finder | SRR11550045 |
| SARS-CoV-2_circ_Homo_sapiens_1581 | MN908947.3 | 14178 | 14584 | + | ORF1ab | 1 | circRNA_finder | SRR11550043 |
| SARS-CoV-2_circ_Homo_sapiens_1583 | MN908947.3 | 14178 | 14590 | + | ORF1ab | 1 | circRNA_finder | SRR11550044 |
| SARS-CoV-2_circ_Homo_sapiens_1583 | MN908947.3 | 14178 | 14590 | + | ORF1ab | 1 | circRNA_finder | SRR11550046 |
| SARS-CoV-2_circ_Homo_sapiens_1586 | MN908947.3 | 14178 | 14696 | + | ORF1ab | 1 | circRNA_finder | SRR11550044 |
| SARS-CoV-2_circ_Homo_sapiens_1587 | MN908947.3 | 14178 | 14785 | + | ORF1ab | 1 | circRNA_finder | SRR11550046 |
| SARS-CoV-2_circ_Homo_sapiens_1588 | MN908947.3 | 14178 | 14804 | + | ORF1ab | 1 | circRNA_finder | SRR11550045 |
| SARS-CoV-2_circ_Homo_sapiens_1590 | MN908947.3 | 14178 | 14847 | + | ORF1ab | 1 | circRNA_finder | SRR11550046 |
| SARS-CoV-2_circ_Homo_sapiens_1591 | MN908947.3 | 14178 | 15319 | + | ORF1ab | 1 | circRNA_finder | SRR11550046 |
| SARS-CoV-2_circ_Homo_sapiens_1593 | MN908947.3 | 14178 | 16422 | + | ORF1ab | 1 | circRNA_finder | SRR11550045 |
| SARS-CoV-2_circ_Homo_sapiens_1596 | MN908947.3 | 14178 | 20404 | + | ORF1ab | 1 | circRNA_finder | SRR11550043 |
| SARS-CoV-2_circ_Homo_sapiens_1599 | MN908947.3 | 14178 | 28686 | + | ORF6;M;E;ORF3a;S;N;ORF8;ORF7a;ORF7b;ORF1ab | 1 | circRNA_finder | SRR11550046 |
| SARS-CoV-2_circ_Homo_sapiens_1601 | MN908947.3 | 14178 | 29579 | + | ORF6;ORF10;M;E;ORF3a;S;N;ORF8;ORF7a;ORF7b;ORF1ab | 1 | circRNA_finder | SRR11550044 |
| SARS-CoV-2_circ_Homo_sapiens_1602 | MN908947.3 | 14178 | 29773 | + | ORF6;ORF10;M;E;ORF3a;S;N;ORF8;ORF7a;ORF7b;ORF1ab | 1 | circRNA_finder | SRR11550043 |
| SARS-CoV-2_circ_Homo_sapiens_1604 | MN908947.3 | 14184 | 29268 | + | ORF6;M;E;ORF3a;S;N;ORF8;ORF7a;ORF7b;ORF1ab | 1 | circRNA_finder | SRR11550046 |
| SARS-CoV-2_circ_Homo_sapiens_1606 | MN908947.3 | 14207 | 14572 | + | ORF1ab | 1 | circRNA_finder | SRR11550045 |
| SARS-CoV-2_circ_Homo_sapiens_161 | MN908947.3 | 689 | 25249 | + | S;ORF1ab | 1 | circRNA_finder | SRR11550043 |
| SARS-CoV-2_circ_Homo_sapiens_1613 | MN908947.3 | 14408 | 14785 | + | ORF1ab | 1 | circRNA_finder | SRR11550046 |
| SARS-CoV-2_circ_Homo_sapiens_1615 | MN908947.3 | 14408 | 14804 | + | ORF1ab | 1 | circRNA_finder | SRR11550045 |
| SARS-CoV-2_circ_Homo_sapiens_1617 | MN908947.3 | 14408 | 15406 | + | ORF1ab | 1 | circRNA_finder | SRR11550046 |
| SARS-CoV-2_circ_Homo_sapiens_1618 | MN908947.3 | 14408 | 15856 | + | ORF1ab | 1 | circRNA_finder | SRR11550045 |
| SARS-CoV-2_circ_Homo_sapiens_1619 | MN908947.3 | 14408 | 16750 | + | ORF1ab | 1 | circRNA_finder | SRR11550046 |
| SARS-CoV-2_circ_Homo_sapiens_1621 | MN908947.3 | 14408 | 20269 | + | ORF1ab | 1 | circRNA_finder | SRR11550046 |
| SARS-CoV-2_circ_Homo_sapiens_1622 | MN908947.3 | 14412 | 17009 | + | ORF1ab | 1 | circRNA_finder | SRR11550045 |
| SARS-CoV-2_circ_Homo_sapiens_1623 | MN908947.3 | 14412 | 27683 | + | ORF6;M;E;ORF3a;S;ORF7a;ORF1ab | 1 | circRNA_finder | SRR11550046 |
| SARS-CoV-2_circ_Homo_sapiens_1624 | MN908947.3 | 14415 | 16500 | + | ORF1ab | 1 | circRNA_finder | SRR11550043 |
| SARS-CoV-2_circ_Homo_sapiens_1628 | MN908947.3 | 14504 | 14804 | + | ORF1ab | 1 | circRNA_finder | SRR11550046 |
| SARS-CoV-2_circ_Homo_sapiens_1629 | MN908947.3 | 14541 | 15198 | + | ORF1ab | 1 | circRNA_finder | SRR11550044 |
| SARS-CoV-2_circ_Homo_sapiens_1630 | MN908947.3 | 14609 | 15037 | + | ORF1ab | 1 | circRNA_finder | SRR11550043 |
| SARS-CoV-2_circ_Homo_sapiens_1632 | MN908947.3 | 14609 | 15201 | + | ORF1ab | 1 | circRNA_finder | SRR11550044 |
| SARS-CoV-2_circ_Homo_sapiens_1638 | MN908947.3 | 14646 | 15351 | + | ORF1ab | 1 | circRNA_finder | SRR11550044 |
| SARS-CoV-2_circ_Homo_sapiens_1639 | MN908947.3 | 14650 | 15852 | + | ORF1ab | 1 | circRNA_finder | SRR11550045 |
| SARS-CoV-2_circ_Homo_sapiens_1644 | MN908947.3 | 14758 | 16761 | + | ORF1ab | 1 | circRNA_finder | SRR11550044 |
| SARS-CoV-2_circ_Homo_sapiens_1646 | MN908947.3 | 14858 | 15731 | + | ORF1ab | 1 | circRNA_finder | SRR11550044 |
| SARS-CoV-2_circ_Homo_sapiens_1647 | MN908947.3 | 14858 | 15969 | + | ORF1ab | 1 | circRNA_finder | SRR11550044 |
| SARS-CoV-2_circ_Homo_sapiens_1648 | MN908947.3 | 14858 | 16088 | + | ORF1ab | 1 | circRNA_finder | SRR11550043 |
| SARS-CoV-2_circ_Homo_sapiens_1649 | MN908947.3 | 14858 | 16486 | + | ORF1ab | 1 | circRNA_finder | SRR11550045 |
| SARS-CoV-2_circ_Homo_sapiens_1650 | MN908947.3 | 14858 | 16911 | + | ORF1ab | 1 | circRNA_finder | SRR11550045 |
| SARS-CoV-2_circ_Homo_sapiens_1651 | MN908947.3 | 14878 | 15782 | + | ORF1ab | 1 | circRNA_finder | SRR11550045 |
| SARS-CoV-2_circ_Homo_sapiens_1653 | MN908947.3 | 14914 | 18880 | + | ORF1ab | 1 | circRNA_finder | SRR11550043 |
| SARS-CoV-2_circ_Homo_sapiens_1654 | MN908947.3 | 14932 | 19549 | + | ORF1ab | 1 | circRNA_finder | SRR11550043 |
| SARS-CoV-2_circ_Homo_sapiens_1655 | MN908947.3 | 14932 | 19982 | + | ORF1ab | 1 | circRNA_finder | SRR11550045 |
| SARS-CoV-2_circ_Homo_sapiens_1656 | MN908947.3 | 14936 | 16088 | + | ORF1ab | 1 | circRNA_finder | SRR11550046 |
| SARS-CoV-2_circ_Homo_sapiens_1657 | MN908947.3 | 14936 | 16297 | + | ORF1ab | 1 | circRNA_finder | SRR11550043 |
| SARS-CoV-2_circ_Homo_sapiens_1658 | MN908947.3 | 15020 | 15618 | + | ORF1ab | 1 | circRNA_finder | SRR11550045 |
| SARS-CoV-2_circ_Homo_sapiens_1659 | MN908947.3 | 15020 | 25702 | + | ORF3a;S;ORF1ab | 1 | circRNA_finder | SRR11550045 |
| SARS-CoV-2_circ_Homo_sapiens_1662 | MN908947.3 | 15038 | 15319 | + | ORF1ab | 1 | circRNA_finder | SRR11550046 |
| SARS-CoV-2_circ_Homo_sapiens_1664 | MN908947.3 | 15108 | 15745 | + | ORF1ab | 1 | circRNA_finder | SRR11550046 |
| SARS-CoV-2_circ_Homo_sapiens_1665 | MN908947.3 | 15113 | 16699 | + | ORF1ab | 1 | circRNA_finder | SRR11550045 |
| SARS-CoV-2_circ_Homo_sapiens_1666 | MN908947.3 | 15113 | 16942 | + | ORF1ab | 1 | circRNA_finder | SRR11550044 |
| SARS-CoV-2_circ_Homo_sapiens_1667 | MN908947.3 | 15194 | 16744 | + | ORF1ab | 1 | circRNA_finder | SRR11550045 |
| SARS-CoV-2_circ_Homo_sapiens_1668 | MN908947.3 | 15203 | 15788 | + | ORF1ab | 1 | circRNA_finder | SRR11550044 |
| SARS-CoV-2_circ_Homo_sapiens_1669 | MN908947.3 | 15203 | 16312 | + | ORF1ab | 1 | circRNA_finder | SRR11550044 |
| SARS-CoV-2_circ_Homo_sapiens_167 | MN908947.3 | 698 | 1313 | + | ORF1ab | 1 | circRNA_finder | SRR11550045 |
| SARS-CoV-2_circ_Homo_sapiens_1670 | MN908947.3 | 15203 | 16359 | + | ORF1ab | 1 | circRNA_finder | SRR11550043 |
| SARS-CoV-2_circ_Homo_sapiens_1671 | MN908947.3 | 15203 | 16474 | + | ORF1ab | 1 | circRNA_finder | SRR11550046 |
| SARS-CoV-2_circ_Homo_sapiens_1674 | MN908947.3 | 15241 | 17452 | + | ORF1ab | 1 | circRNA_finder | SRR11550046 |
| SARS-CoV-2_circ_Homo_sapiens_1675 | MN908947.3 | 15261 | 16312 | + | ORF1ab | 1 | circRNA_finder | SRR11550046 |
| SARS-CoV-2_circ_Homo_sapiens_1676 | MN908947.3 | 15269 | 15593 | + | ORF1ab | 1 | circRNA_finder | SRR11550044 |
| SARS-CoV-2_circ_Homo_sapiens_1679 | MN908947.3 | 15280 | 15852 | + | ORF1ab | 1 | circRNA_finder | SRR11550045 |
| SARS-CoV-2_circ_Homo_sapiens_168 | MN908947.3 | 698 | 1328 | + | ORF1ab | 1 | circRNA_finder | SRR11550045 |
| SARS-CoV-2_circ_Homo_sapiens_1680 | MN908947.3 | 15280 | 16027 | + | ORF1ab | 1 | circRNA_finder | SRR11550043 |
| SARS-CoV-2_circ_Homo_sapiens_1684 | MN908947.3 | 15280 | 16761 | + | ORF1ab | 1 | circRNA_finder | SRR11550045 |
| SARS-CoV-2_circ_Homo_sapiens_1685 | MN908947.3 | 15280 | 17092 | + | ORF1ab | 1 | circRNA_finder | SRR11550046 |
| SARS-CoV-2_circ_Homo_sapiens_1691 | MN908947.3 | 15333 | 15663 | + | ORF1ab | 1 | circRNA_finder | SRR11550044 |
| SARS-CoV-2_circ_Homo_sapiens_1692 | MN908947.3 | 15333 | 15731 | + | ORF1ab | 1 | circRNA_finder | SRR11550043 |
| SARS-CoV-2_circ_Homo_sapiens_1693 | MN908947.3 | 15333 | 16088 | + | ORF1ab | 1 | circRNA_finder | SRR11550045 |
| SARS-CoV-2_circ_Homo_sapiens_1694 | MN908947.3 | 15333 | 16297 | + | ORF1ab | 1 | circRNA_finder | SRR11550045 |
| SARS-CoV-2_circ_Homo_sapiens_1695 | MN908947.3 | 15333 | 16312 | + | ORF1ab | 1 | circRNA_finder | SRR11550046 |
| SARS-CoV-2_circ_Homo_sapiens_1695 | MN908947.3 | 15333 | 16312 | + | ORF1ab | 1 | circRNA_finder | SRR11550043 |
| SARS-CoV-2_circ_Homo_sapiens_1696 | MN908947.3 | 15333 | 16474 | + | ORF1ab | 1 | circRNA_finder | SRR11550045 |
| SARS-CoV-2_circ_Homo_sapiens_1698 | MN908947.3 | 15333 | 17593 | + | ORF1ab | 1 | circRNA_finder | SRR11550045 |
| SARS-CoV-2_circ_Homo_sapiens_1699 | MN908947.3 | 15333 | 18972 | + | ORF1ab | 1 | circRNA_finder | SRR11550046 |
| SARS-CoV-2_circ_Homo_sapiens_170 | MN908947.3 | 698 | 1472 | + | ORF1ab | 1 | circRNA_finder | SRR11550046 |
| SARS-CoV-2_circ_Homo_sapiens_1700 | MN908947.3 | 15333 | 23561 | + | S;ORF1ab | 1 | circRNA_finder | SRR11550045 |
| SARS-CoV-2_circ_Homo_sapiens_1701 | MN908947.3 | 15347 | 15724 | + | ORF1ab | 1 | circRNA_finder | SRR11550045 |
| SARS-CoV-2_circ_Homo_sapiens_1703 | MN908947.3 | 15347 | 16615 | + | ORF1ab | 1 | circRNA_finder | SRR11550046 |
| SARS-CoV-2_circ_Homo_sapiens_1706 | MN908947.3 | 15402 | 15731 | + | ORF1ab | 1 | circRNA_finder | SRR11550046 |
| SARS-CoV-2_circ_Homo_sapiens_1707 | MN908947.3 | 15402 | 15733 | + | ORF1ab | 1 | circRNA_finder | SRR11550045 |
| SARS-CoV-2_circ_Homo_sapiens_1708 | MN908947.3 | 15402 | 15788 | + | ORF1ab | 1 | circRNA_finder | SRR11550045 |
| SARS-CoV-2_circ_Homo_sapiens_1709 | MN908947.3 | 15402 | 15835 | + | ORF1ab | 1 | circRNA_finder | SRR11550043 |
| SARS-CoV-2_circ_Homo_sapiens_1711 | MN908947.3 | 15402 | 16312 | + | ORF1ab | 1 | circRNA_finder | SRR11550044 |
| SARS-CoV-2_circ_Homo_sapiens_1712 | MN908947.3 | 15402 | 16474 | + | ORF1ab | 1 | circRNA_finder | SRR11550046 |
| SARS-CoV-2_circ_Homo_sapiens_1714 | MN908947.3 | 15407 | 15788 | + | ORF1ab | 1 | circRNA_finder | SRR11550046 |
| SARS-CoV-2_circ_Homo_sapiens_1715 | MN908947.3 | 15407 | 15920 | + | ORF1ab | 1 | circRNA_finder | SRR11550046 |
| SARS-CoV-2_circ_Homo_sapiens_1716 | MN908947.3 | 15407 | 15969 | + | ORF1ab | 1 | circRNA_finder | SRR11550044 |
| SARS-CoV-2_circ_Homo_sapiens_1717 | MN908947.3 | 15407 | 16297 | + | ORF1ab | 1 | circRNA_finder | SRR11550043 |
| SARS-CoV-2_circ_Homo_sapiens_1719 | MN908947.3 | 15407 | 18016 | + | ORF1ab | 1 | circRNA_finder | SRR11550043 |
| SARS-CoV-2_circ_Homo_sapiens_1720 | MN908947.3 | 15415 | 16399 | + | ORF1ab | 1 | circRNA_finder | SRR11550045 |
| SARS-CoV-2_circ_Homo_sapiens_1721 | MN908947.3 | 15526 | 16307 | + | ORF1ab | 1 | circRNA_finder | SRR11550046 |
| SARS-CoV-2_circ_Homo_sapiens_1722 | MN908947.3 | 15536 | 16297 | + | ORF1ab | 1 | circRNA_finder | SRR11550046 |
| SARS-CoV-2_circ_Homo_sapiens_1725 | MN908947.3 | 15560 | 19585 | + | ORF1ab | 1 | circRNA_finder | SRR11550046 |
| SARS-CoV-2_circ_Homo_sapiens_1726 | MN908947.3 | 15580 | 16603 | + | ORF1ab | 1 | circRNA_finder | SRR11550046 |
| SARS-CoV-2_circ_Homo_sapiens_1727 | MN908947.3 | 15580 | 16674 | + | ORF1ab | 1 | circRNA_finder | SRR11550044 |
| SARS-CoV-2_circ_Homo_sapiens_1728 | MN908947.3 | 15595 | 16312 | + | ORF1ab | 1 | circRNA_finder | SRR11550046 |
| SARS-CoV-2_circ_Homo_sapiens_1728 | MN908947.3 | 15595 | 16312 | + | ORF1ab | 1 | circRNA_finder | SRR11550045 |
| SARS-CoV-2_circ_Homo_sapiens_1730 | MN908947.3 | 15721 | 18355 | + | ORF1ab | 1 | circRNA_finder | SRR11550046 |
| SARS-CoV-2_circ_Homo_sapiens_1732 | MN908947.3 | 15744 | 16312 | + | ORF1ab | 1 | circRNA_finder | SRR11550046 |
| SARS-CoV-2_circ_Homo_sapiens_1733 | MN908947.3 | 15744 | 18890 | + | ORF1ab | 1 | circRNA_finder | SRR11550045 |
| SARS-CoV-2_circ_Homo_sapiens_1735 | MN908947.3 | 15784 | 16307 | + | ORF1ab | 1 | circRNA_finder | SRR11550043 |
| SARS-CoV-2_circ_Homo_sapiens_1736 | MN908947.3 | 15784 | 18003 | + | ORF1ab | 1 | circRNA_finder | SRR11550045 |
| SARS-CoV-2_circ_Homo_sapiens_1744 | MN908947.3 | 15853 | 16307 | + | ORF1ab | 1 | circRNA_finder | SRR11550044 |
| SARS-CoV-2_circ_Homo_sapiens_1746 | MN908947.3 | 15858 | 17009 | + | ORF1ab | 1 | circRNA_finder | SRR11550045 |
| SARS-CoV-2_circ_Homo_sapiens_1747 | MN908947.3 | 15866 | 16270 | + | ORF1ab | 1 | circRNA_finder | SRR11550044 |
| SARS-CoV-2_circ_Homo_sapiens_1749 | MN908947.3 | 15925 | 16270 | + | ORF1ab | 1 | circRNA_finder | SRR11550045 |
| SARS-CoV-2_circ_Homo_sapiens_1750 | MN908947.3 | 15956 | 16312 | + | ORF1ab | 1 | circRNA_finder | SRR11550045 |
| SARS-CoV-2_circ_Homo_sapiens_1750 | MN908947.3 | 15956 | 16312 | + | ORF1ab | 1 | circRNA_finder | SRR11550046 |
| SARS-CoV-2_circ_Homo_sapiens_1751 | MN908947.3 | 15956 | 16359 | + | ORF1ab | 1 | circRNA_finder | SRR11550046 |
| SARS-CoV-2_circ_Homo_sapiens_1752 | MN908947.3 | 16028 | 16297 | + | ORF1ab | 1 | circRNA_finder | SRR11550043 |
| SARS-CoV-2_circ_Homo_sapiens_1753 | MN908947.3 | 16028 | 16315 | + | ORF1ab | 1 | circRNA_finder | SRR11550045 |
| SARS-CoV-2_circ_Homo_sapiens_1753 | MN908947.3 | 16028 | 16315 | + | ORF1ab | 1 | circRNA_finder | SRR11550046 |
| SARS-CoV-2_circ_Homo_sapiens_1755 | MN908947.3 | 16028 | 16794 | + | ORF1ab | 1 | circRNA_finder | SRR11550044 |
| SARS-CoV-2_circ_Homo_sapiens_1756 | MN908947.3 | 16028 | 16929 | + | ORF1ab | 1 | circRNA_finder | SRR11550044 |
| SARS-CoV-2_circ_Homo_sapiens_1758 | MN908947.3 | 16050 | 16603 | + | ORF1ab | 1 | circRNA_finder | SRR11550044 |
| SARS-CoV-2_circ_Homo_sapiens_176 | MN908947.3 | 702 | 909 | + | ORF1ab | 1 | circRNA_finder | SRR11550046 |
| SARS-CoV-2_circ_Homo_sapiens_1760 | MN908947.3 | 16198 | 22858 | + | S;ORF1ab | 1 | circRNA_finder | SRR11550044 |
| SARS-CoV-2_circ_Homo_sapiens_1761 | MN908947.3 | 16298 | 19521 | + | ORF1ab | 1 | circRNA_finder | SRR11550046 |
| SARS-CoV-2_circ_Homo_sapiens_1762 | MN908947.3 | 16302 | 16942 | + | ORF1ab | 1 | circRNA_finder | SRR11550043 |
| SARS-CoV-2_circ_Homo_sapiens_1763 | MN908947.3 | 16330 | 17326 | + | ORF1ab | 1 | circRNA_finder | SRR11550044 |
| SARS-CoV-2_circ_Homo_sapiens_1764 | MN908947.3 | 16330 | 24462 | + | S;ORF1ab | 1 | circRNA_finder | SRR11550045 |
| SARS-CoV-2_circ_Homo_sapiens_1768 | MN908947.3 | 16361 | 16942 | + | ORF1ab | 1 | circRNA_finder | SRR11550045 |
| SARS-CoV-2_circ_Homo_sapiens_1769 | MN908947.3 | 16397 | 16695 | + | ORF1ab | 1 | circRNA_finder | SRR11550044 |
| SARS-CoV-2_circ_Homo_sapiens_177 | MN908947.3 | 702 | 1534 | + | ORF1ab | 1 | circRNA_finder | SRR11550046 |
| SARS-CoV-2_circ_Homo_sapiens_177 | MN908947.3 | 702 | 1534 | + | ORF1ab | 1 | circRNA_finder | SRR11550044 |
| SARS-CoV-2_circ_Homo_sapiens_1770 | MN908947.3 | 16397 | 16794 | + | ORF1ab | 1 | circRNA_finder | SRR11550043 |
| SARS-CoV-2_circ_Homo_sapiens_1771 | MN908947.3 | 16397 | 16929 | + | ORF1ab | 1 | circRNA_finder | SRR11550046 |
| SARS-CoV-2_circ_Homo_sapiens_1772 | MN908947.3 | 16397 | 16974 | + | ORF1ab | 1 | circRNA_finder | SRR11550046 |
| SARS-CoV-2_circ_Homo_sapiens_1773 | MN908947.3 | 16397 | 17018 | + | ORF1ab | 1 | circRNA_finder | SRR11550046 |
| SARS-CoV-2_circ_Homo_sapiens_1774 | MN908947.3 | 16397 | 17763 | + | ORF1ab | 1 | circRNA_finder | SRR11550045 |
| SARS-CoV-2_circ_Homo_sapiens_1777 | MN908947.3 | 16397 | 19413 | + | ORF1ab | 1 | circRNA_finder | SRR11550046 |
| SARS-CoV-2_circ_Homo_sapiens_1778 | MN908947.3 | 16424 | 18492 | + | ORF1ab | 1 | circRNA_finder | SRR11550046 |
| SARS-CoV-2_circ_Homo_sapiens_1779 | MN908947.3 | 16429 | 17009 | + | ORF1ab | 1 | circRNA_finder | SRR11550045 |
| SARS-CoV-2_circ_Homo_sapiens_178 | MN908947.3 | 702 | 28320 | + | ORF6;M;E;ORF3a;S;N;ORF8;ORF7a;ORF7b;ORF1ab | 1 | circRNA_finder | SRR11550044 |
| SARS-CoV-2_circ_Homo_sapiens_1781 | MN908947.3 | 16443 | 16929 | + | ORF1ab | 1 | circRNA_finder | SRR11550046 |
| SARS-CoV-2_circ_Homo_sapiens_179 | MN908947.3 | 707 | 1937 | + | ORF1ab | 1 | circRNA_finder | SRR11550045 |
| SARS-CoV-2_circ_Homo_sapiens_1790 | MN908947.3 | 16552 | 27218 | + | ORF6;M;E;ORF3a;S;ORF1ab | 1 | circRNA_finder | SRR11550044 |
| SARS-CoV-2_circ_Homo_sapiens_1793 | MN908947.3 | 16611 | 18394 | + | ORF1ab | 1 | circRNA_finder | SRR11550043 |
| SARS-CoV-2_circ_Homo_sapiens_1795 | MN908947.3 | 16630 | 16974 | + | ORF1ab | 1 | circRNA_finder | SRR11550046 |
| SARS-CoV-2_circ_Homo_sapiens_1795 | MN908947.3 | 16630 | 16974 | + | ORF1ab | 1 | circRNA_finder | SRR11550043 |
| SARS-CoV-2_circ_Homo_sapiens_1796 | MN908947.3 | 16659 | 18355 | + | ORF1ab | 1 | circRNA_finder | SRR11550044 |
| SARS-CoV-2_circ_Homo_sapiens_1798 | MN908947.3 | 16676 | 26954 | + | M;E;ORF3a;S;ORF1ab | 1 | circRNA_finder | SRR11550043 |
| SARS-CoV-2_circ_Homo_sapiens_1799 | MN908947.3 | 16700 | 28892 | + | ORF6;M;E;ORF3a;S;N;ORF8;ORF7a;ORF7b;ORF1ab | 1 | circRNA_finder | SRR11550046 |
| SARS-CoV-2_circ_Homo_sapiens_180 | MN908947.3 | 710 | 1273 | + | ORF1ab | 1 | circRNA_finder | SRR11550044 |
| SARS-CoV-2_circ_Homo_sapiens_1802 | MN908947.3 | 16727 | 18355 | + | ORF1ab | 1 | circRNA_finder | SRR11550043 |
| SARS-CoV-2_circ_Homo_sapiens_1803 | MN908947.3 | 16751 | 17126 | + | ORF1ab | 1 | circRNA_finder | SRR11550045 |
| SARS-CoV-2_circ_Homo_sapiens_1805 | MN908947.3 | 16751 | 18325 | + | ORF1ab | 1 | circRNA_finder | SRR11550045 |
| SARS-CoV-2_circ_Homo_sapiens_1806 | MN908947.3 | 16751 | 18558 | + | ORF1ab | 1 | circRNA_finder | SRR11550045 |
| SARS-CoV-2_circ_Homo_sapiens_1808 | MN908947.3 | 16751 | 18876 | + | ORF1ab | 1 | circRNA_finder | SRR11550043 |
| SARS-CoV-2_circ_Homo_sapiens_1808 | MN908947.3 | 16751 | 18876 | + | ORF1ab | 1 | circRNA_finder | SRR11550045 |
| SARS-CoV-2_circ_Homo_sapiens_1809 | MN908947.3 | 16751 | 19549 | + | ORF1ab | 1 | circRNA_finder | SRR11550045 |
| SARS-CoV-2_circ_Homo_sapiens_181 | MN908947.3 | 710 | 1357 | + | ORF1ab | 1 | circRNA_finder | SRR11550045 |
| SARS-CoV-2_circ_Homo_sapiens_1811 | MN908947.3 | 16751 | 28015 | + | ORF6;M;E;ORF3a;S;ORF8;ORF7a;ORF7b;ORF1ab | 1 | circRNA_finder | SRR11550045 |
| SARS-CoV-2_circ_Homo_sapiens_1813 | MN908947.3 | 16755 | 18497 | + | ORF1ab | 1 | circRNA_finder | SRR11550043 |
| SARS-CoV-2_circ_Homo_sapiens_1814 | MN908947.3 | 16757 | 18355 | + | ORF1ab | 1 | circRNA_finder | SRR11550043 |
| SARS-CoV-2_circ_Homo_sapiens_1815 | MN908947.3 | 16800 | 17569 | + | ORF1ab | 1 | circRNA_finder | SRR11550044 |
| SARS-CoV-2_circ_Homo_sapiens_1817 | MN908947.3 | 16800 | 28472 | + | ORF6;M;E;ORF3a;S;N;ORF8;ORF7a;ORF7b;ORF1ab | 1 | circRNA_finder | SRR11550044 |
| SARS-CoV-2_circ_Homo_sapiens_1818 | MN908947.3 | 16823 | 17308 | + | ORF1ab | 1 | circRNA_finder | SRR11550046 |
| SARS-CoV-2_circ_Homo_sapiens_182 | MN908947.3 | 710 | 1360 | + | ORF1ab | 1 | circRNA_finder | SRR11550044 |
| SARS-CoV-2_circ_Homo_sapiens_1823 | MN908947.3 | 16980 | 17410 | + | ORF1ab | 1 | circRNA_finder | SRR11550046 |
| SARS-CoV-2_circ_Homo_sapiens_1826 | MN908947.3 | 17020 | 18897 | + | ORF1ab | 1 | circRNA_finder | SRR11550045 |
| SARS-CoV-2_circ_Homo_sapiens_1827 | MN908947.3 | 17020 | 25649 | + | ORF3a;S;ORF1ab | 1 | circRNA_finder | SRR11550044 |
| SARS-CoV-2_circ_Homo_sapiens_183 | MN908947.3 | 710 | 1534 | + | ORF1ab | 1 | circRNA_finder | SRR11550046 |
| SARS-CoV-2_circ_Homo_sapiens_1831 | MN908947.3 | 17232 | 23561 | + | S;ORF1ab | 1 | circRNA_finder | SRR11550043 |
| SARS-CoV-2_circ_Homo_sapiens_1832 | MN908947.3 | 17342 | 18016 | + | ORF1ab | 1 | circRNA_finder | SRR11550044 |
| SARS-CoV-2_circ_Homo_sapiens_1836 | MN908947.3 | 17342 | 23092 | + | S;ORF1ab | 1 | circRNA_finder | SRR11550044 |
| SARS-CoV-2_circ_Homo_sapiens_1837 | MN908947.3 | 17342 | 23567 | + | S;ORF1ab | 1 | circRNA_finder | SRR11550045 |
| SARS-CoV-2_circ_Homo_sapiens_184 | MN908947.3 | 732 | 1337 | + | ORF1ab | 1 | circRNA_finder | SRR11550045 |
| SARS-CoV-2_circ_Homo_sapiens_1840 | MN908947.3 | 17406 | 18254 | + | ORF1ab | 1 | circRNA_finder | SRR11550045 |
| SARS-CoV-2_circ_Homo_sapiens_1842 | MN908947.3 | 17406 | 18379 | + | ORF1ab | 1 | circRNA_finder | SRR11550044 |
| SARS-CoV-2_circ_Homo_sapiens_1843 | MN908947.3 | 17406 | 18444 | + | ORF1ab | 1 | circRNA_finder | SRR11550046 |
| SARS-CoV-2_circ_Homo_sapiens_1845 | MN908947.3 | 17406 | 18897 | + | ORF1ab | 1 | circRNA_finder | SRR11550044 |
| SARS-CoV-2_circ_Homo_sapiens_1846 | MN908947.3 | 17406 | 19347 | + | ORF1ab | 1 | circRNA_finder | SRR11550045 |
| SARS-CoV-2_circ_Homo_sapiens_1847 | MN908947.3 | 17406 | 20144 | + | ORF1ab | 1 | circRNA_finder | SRR11550044 |
| SARS-CoV-2_circ_Homo_sapiens_1848 | MN908947.3 | 17406 | 21607 | + | S;ORF1ab | 1 | circRNA_finder | SRR11550046 |
| SARS-CoV-2_circ_Homo_sapiens_1849 | MN908947.3 | 17406 | 21700 | + | S;ORF1ab | 1 | circRNA_finder | SRR11550045 |
| SARS-CoV-2_circ_Homo_sapiens_185 | MN908947.3 | 732 | 2425 | + | ORF1ab | 1 | circRNA_finder | SRR11550043 |
| SARS-CoV-2_circ_Homo_sapiens_1850 | MN908947.3 | 17406 | 26256 | + | ORF1ab;ORF3a;S;E | 1 | circRNA_finder | SRR11550044 |
| SARS-CoV-2_circ_Homo_sapiens_1852 | MN908947.3 | 17411 | 18355 | + | ORF1ab | 1 | circRNA_finder | SRR11550043 |
| SARS-CoV-2_circ_Homo_sapiens_1853 | MN908947.3 | 17411 | 18813 | + | ORF1ab | 1 | circRNA_finder | SRR11550044 |
| SARS-CoV-2_circ_Homo_sapiens_1855 | MN908947.3 | 17431 | 17765 | + | ORF1ab | 1 | circRNA_finder | SRR11550043 |
| SARS-CoV-2_circ_Homo_sapiens_1855 | MN908947.3 | 17431 | 17765 | + | ORF1ab | 1 | circRNA_finder | SRR11550044 |
| SARS-CoV-2_circ_Homo_sapiens_1857 | MN908947.3 | 17431 | 18558 | + | ORF1ab | 1 | circRNA_finder | SRR11550044 |
| SARS-CoV-2_circ_Homo_sapiens_1858 | MN908947.3 | 17431 | 18880 | + | ORF1ab | 1 | circRNA_finder | SRR11550045 |
| SARS-CoV-2_circ_Homo_sapiens_1859 | MN908947.3 | 17453 | 18175 | + | ORF1ab | 1 | circRNA_finder | SRR11550043 |
| SARS-CoV-2_circ_Homo_sapiens_1860 | MN908947.3 | 17466 | 17733 | + | ORF1ab | 1 | circRNA_finder | SRR11550046 |
| SARS-CoV-2_circ_Homo_sapiens_1864 | MN908947.3 | 17617 | 17963 | + | ORF1ab | 1 | circRNA_finder | SRR11550045 |
| SARS-CoV-2_circ_Homo_sapiens_1866 | MN908947.3 | 17627 | 18130 | + | ORF1ab | 1 | circRNA_finder | SRR11550043 |
| SARS-CoV-2_circ_Homo_sapiens_1867 | MN908947.3 | 17633 | 18744 | + | ORF1ab | 1 | circRNA_finder | SRR11550045 |
| SARS-CoV-2_circ_Homo_sapiens_1869 | MN908947.3 | 17741 | 18876 | + | ORF1ab | 1 | circRNA_finder | SRR11550044 |
| SARS-CoV-2_circ_Homo_sapiens_187 | MN908947.3 | 732 | 3517 | + | ORF1ab | 1 | circRNA_finder | SRR11550044 |
| SARS-CoV-2_circ_Homo_sapiens_1870 | MN908947.3 | 17777 | 18142 | + | ORF1ab | 1 | circRNA_finder | SRR11550046 |
| SARS-CoV-2_circ_Homo_sapiens_1871 | MN908947.3 | 17777 | 18166 | + | ORF1ab | 1 | circRNA_finder | SRR11550045 |
| SARS-CoV-2_circ_Homo_sapiens_1872 | MN908947.3 | 17777 | 18646 | + | ORF1ab | 1 | circRNA_finder | SRR11550043 |
| SARS-CoV-2_circ_Homo_sapiens_1873 | MN908947.3 | 17809 | 18148 | + | ORF1ab | 1 | circRNA_finder | SRR11550046 |
| SARS-CoV-2_circ_Homo_sapiens_1874 | MN908947.3 | 17809 | 18444 | + | ORF1ab | 1 | circRNA_finder | SRR11550046 |
| SARS-CoV-2_circ_Homo_sapiens_1875 | MN908947.3 | 17809 | 23161 | + | S;ORF1ab | 1 | circRNA_finder | SRR11550044 |
| SARS-CoV-2_circ_Homo_sapiens_1876 | MN908947.3 | 17809 | 24943 | + | S;ORF1ab | 1 | circRNA_finder | SRR11550043 |
| SARS-CoV-2_circ_Homo_sapiens_1877 | MN908947.3 | 17848 | 18820 | + | ORF1ab | 1 | circRNA_finder | SRR11550044 |
| SARS-CoV-2_circ_Homo_sapiens_1881 | MN908947.3 | 17916 | 18214 | + | ORF1ab | 1 | circRNA_finder | SRR11550045 |
| SARS-CoV-2_circ_Homo_sapiens_1885 | MN908947.3 | 18017 | 18247 | + | ORF1ab | 1 | circRNA_finder | SRR11550043 |
| SARS-CoV-2_circ_Homo_sapiens_1887 | MN908947.3 | 18017 | 18274 | + | ORF1ab | 1 | circRNA_finder | SRR11550046 |
| SARS-CoV-2_circ_Homo_sapiens_1888 | MN908947.3 | 18017 | 18328 | + | ORF1ab | 1 | circRNA_finder | SRR11550046 |
| SARS-CoV-2_circ_Homo_sapiens_1888 | MN908947.3 | 18017 | 18328 | + | ORF1ab | 1 | circRNA_finder | SRR11550044 |
| SARS-CoV-2_circ_Homo_sapiens_1888 | MN908947.3 | 18017 | 18328 | + | ORF1ab | 1 | circRNA_finder | SRR11550045 |
| SARS-CoV-2_circ_Homo_sapiens_1889 | MN908947.3 | 18017 | 18373 | + | ORF1ab | 1 | circRNA_finder | SRR11550043 |
| SARS-CoV-2_circ_Homo_sapiens_1889 | MN908947.3 | 18017 | 18373 | + | ORF1ab | 1 | circRNA_finder | SRR11550044 |
| SARS-CoV-2_circ_Homo_sapiens_189 | MN908947.3 | 759 | 1436 | + | ORF1ab | 1 | circRNA_finder | SRR11550046 |
| SARS-CoV-2_circ_Homo_sapiens_1890 | MN908947.3 | 18017 | 18387 | + | ORF1ab | 1 | circRNA_finder | SRR11550046 |
| SARS-CoV-2_circ_Homo_sapiens_1890 | MN908947.3 | 18017 | 18387 | + | ORF1ab | 1 | circRNA_finder | SRR11550045 |
| SARS-CoV-2_circ_Homo_sapiens_1894 | MN908947.3 | 18017 | 18451 | + | ORF1ab | 1 | circRNA_finder | SRR11550046 |
| SARS-CoV-2_circ_Homo_sapiens_1894 | MN908947.3 | 18017 | 18451 | + | ORF1ab | 1 | circRNA_finder | SRR11550043 |
| SARS-CoV-2_circ_Homo_sapiens_1898 | MN908947.3 | 18017 | 18727 | + | ORF1ab | 1 | circRNA_finder | SRR11550044 |
| SARS-CoV-2_circ_Homo_sapiens_1899 | MN908947.3 | 18017 | 18746 | + | ORF1ab | 1 | circRNA_finder | SRR11550046 |
| SARS-CoV-2_circ_Homo_sapiens_190 | MN908947.3 | 794 | 1357 | + | ORF1ab | 1 | circRNA_finder | SRR11550046 |
| SARS-CoV-2_circ_Homo_sapiens_1903 | MN908947.3 | 18017 | 19048 | + | ORF1ab | 1 | circRNA_finder | SRR11550046 |
| SARS-CoV-2_circ_Homo_sapiens_1908 | MN908947.3 | 18017 | 20098 | + | ORF1ab | 1 | circRNA_finder | SRR11550046 |
| SARS-CoV-2_circ_Homo_sapiens_1909 | MN908947.3 | 18017 | 20428 | + | ORF1ab | 1 | circRNA_finder | SRR11550043 |
| SARS-CoV-2_circ_Homo_sapiens_1910 | MN908947.3 | 18017 | 20843 | + | ORF1ab | 1 | circRNA_finder | SRR11550044 |
| SARS-CoV-2_circ_Homo_sapiens_1910 | MN908947.3 | 18017 | 20843 | + | ORF1ab | 1 | circRNA_finder | SRR11550043 |
| SARS-CoV-2_circ_Homo_sapiens_1911 | MN908947.3 | 18017 | 21058 | + | ORF1ab | 1 | circRNA_finder | SRR11550045 |
| SARS-CoV-2_circ_Homo_sapiens_1912 | MN908947.3 | 18017 | 21761 | + | S;ORF1ab | 1 | circRNA_finder | SRR11550045 |
| SARS-CoV-2_circ_Homo_sapiens_1913 | MN908947.3 | 18017 | 23480 | + | S;ORF1ab | 1 | circRNA_finder | SRR11550045 |
| SARS-CoV-2_circ_Homo_sapiens_1914 | MN908947.3 | 18017 | 23624 | + | S;ORF1ab | 1 | circRNA_finder | SRR11550043 |
| SARS-CoV-2_circ_Homo_sapiens_1915 | MN908947.3 | 18017 | 24022 | + | S;ORF1ab | 1 | circRNA_finder | SRR11550045 |
| SARS-CoV-2_circ_Homo_sapiens_1916 | MN908947.3 | 18017 | 27130 | + | M;E;ORF3a;S;ORF1ab | 1 | circRNA_finder | SRR11550045 |
| SARS-CoV-2_circ_Homo_sapiens_1917 | MN908947.3 | 18021 | 18309 | + | ORF1ab | 1 | circRNA_finder | SRR11550045 |
| SARS-CoV-2_circ_Homo_sapiens_1919 | MN908947.3 | 18021 | 18390 | + | ORF1ab | 1 | circRNA_finder | SRR11550046 |
| SARS-CoV-2_circ_Homo_sapiens_192 | MN908947.3 | 830 | 2449 | + | ORF1ab | 1 | circRNA_finder | SRR11550045 |
| SARS-CoV-2_circ_Homo_sapiens_1920 | MN908947.3 | 18021 | 18448 | + | ORF1ab | 1 | circRNA_finder | SRR11550045 |
| SARS-CoV-2_circ_Homo_sapiens_1921 | MN908947.3 | 18021 | 18497 | + | ORF1ab | 1 | circRNA_finder | SRR11550044 |
| SARS-CoV-2_circ_Homo_sapiens_1921 | MN908947.3 | 18021 | 18497 | + | ORF1ab | 1 | circRNA_finder | SRR11550045 |
| SARS-CoV-2_circ_Homo_sapiens_1921 | MN908947.3 | 18021 | 18497 | + | ORF1ab | 1 | circRNA_finder | SRR11550043 |
| SARS-CoV-2_circ_Homo_sapiens_1923 | MN908947.3 | 18021 | 18815 | + | ORF1ab | 1 | circRNA_finder | SRR11550046 |
| SARS-CoV-2_circ_Homo_sapiens_1928 | MN908947.3 | 18021 | 19347 | + | ORF1ab | 1 | circRNA_finder | SRR11550045 |
| SARS-CoV-2_circ_Homo_sapiens_1930 | MN908947.3 | 18021 | 27191 | + | M;E;ORF3a;S;ORF1ab | 1 | circRNA_finder | SRR11550046 |
| SARS-CoV-2_circ_Homo_sapiens_1933 | MN908947.3 | 18038 | 20404 | + | ORF1ab | 1 | circRNA_finder | SRR11550046 |
| SARS-CoV-2_circ_Homo_sapiens_1936 | MN908947.3 | 18068 | 21700 | + | S;ORF1ab | 1 | circRNA_finder | SRR11550043 |
| SARS-CoV-2_circ_Homo_sapiens_1937 | MN908947.3 | 18075 | 19299 | + | ORF1ab | 1 | circRNA_finder | SRR11550046 |
| SARS-CoV-2_circ_Homo_sapiens_1939 | MN908947.3 | 18163 | 18876 | + | ORF1ab | 1 | circRNA_finder | SRR11550045 |
| SARS-CoV-2_circ_Homo_sapiens_194 | MN908947.3 | 863 | 1244 | + | ORF1ab | 1 | circRNA_finder | SRR11550045 |
| SARS-CoV-2_circ_Homo_sapiens_1940 | MN908947.3 | 18167 | 18876 | + | ORF1ab | 1 | circRNA_finder | SRR11550044 |
| SARS-CoV-2_circ_Homo_sapiens_1942 | MN908947.3 | 18253 | 18656 | + | ORF1ab | 1 | circRNA_finder | SRR11550044 |
| SARS-CoV-2_circ_Homo_sapiens_1944 | MN908947.3 | 18253 | 18813 | + | ORF1ab | 1 | circRNA_finder | SRR11550046 |
| SARS-CoV-2_circ_Homo_sapiens_1945 | MN908947.3 | 18275 | 18582 | + | ORF1ab | 1 | circRNA_finder | SRR11550045 |
| SARS-CoV-2_circ_Homo_sapiens_1949 | MN908947.3 | 18282 | 18744 | + | ORF1ab | 1 | circRNA_finder | SRR11550044 |
| SARS-CoV-2_circ_Homo_sapiens_1952 | MN908947.3 | 18284 | 18646 | + | ORF1ab | 1 | circRNA_finder | SRR11550044 |
| SARS-CoV-2_circ_Homo_sapiens_1953 | MN908947.3 | 18348 | 18727 | + | ORF1ab | 1 | circRNA_finder | SRR11550043 |
| SARS-CoV-2_circ_Homo_sapiens_1954 | MN908947.3 | 18356 | 18813 | + | ORF1ab | 1 | circRNA_finder | SRR11550043 |
| SARS-CoV-2_circ_Homo_sapiens_1957 | MN908947.3 | 18356 | 19342 | + | ORF1ab | 1 | circRNA_finder | SRR11550044 |
| SARS-CoV-2_circ_Homo_sapiens_1959 | MN908947.3 | 18356 | 20198 | + | ORF1ab | 1 | circRNA_finder | SRR11550043 |
| SARS-CoV-2_circ_Homo_sapiens_1962 | MN908947.3 | 18356 | 23060 | + | S;ORF1ab | 1 | circRNA_finder | SRR11550044 |
| SARS-CoV-2_circ_Homo_sapiens_1964 | MN908947.3 | 18356 | 25548 | + | ORF3a;S;ORF1ab | 1 | circRNA_finder | SRR11550045 |
| SARS-CoV-2_circ_Homo_sapiens_1967 | MN908947.3 | 18378 | 18876 | + | ORF1ab | 1 | circRNA_finder | SRR11550046 |
| SARS-CoV-2_circ_Homo_sapiens_1969 | MN908947.3 | 18380 | 18897 | + | ORF1ab | 1 | circRNA_finder | SRR11550043 |
| SARS-CoV-2_circ_Homo_sapiens_197 | MN908947.3 | 873 | 5186 | + | ORF1ab | 1 | circRNA_finder | SRR11550045 |
| SARS-CoV-2_circ_Homo_sapiens_1970 | MN908947.3 | 18392 | 18904 | + | ORF1ab | 1 | circRNA_finder | SRR11550045 |
| SARS-CoV-2_circ_Homo_sapiens_1971 | MN908947.3 | 18401 | 18656 | + | ORF1ab | 1 | circRNA_finder | SRR11550043 |
| SARS-CoV-2_circ_Homo_sapiens_1973 | MN908947.3 | 18401 | 20269 | + | ORF1ab | 1 | circRNA_finder | SRR11550043 |
| SARS-CoV-2_circ_Homo_sapiens_1974 | MN908947.3 | 18407 | 18815 | + | ORF1ab | 1 | circRNA_finder | SRR11550045 |
| SARS-CoV-2_circ_Homo_sapiens_1978 | MN908947.3 | 18422 | 18876 | + | ORF1ab | 1 | circRNA_finder | SRR11550045 |
| SARS-CoV-2_circ_Homo_sapiens_1979 | MN908947.3 | 18434 | 18781 | + | ORF1ab | 1 | circRNA_finder | SRR11550044 |
| SARS-CoV-2_circ_Homo_sapiens_198 | MN908947.3 | 873 | 10997 | + | ORF1ab | 1 | circRNA_finder | SRR11550043 |
| SARS-CoV-2_circ_Homo_sapiens_1980 | MN908947.3 | 18450 | 18744 | + | ORF1ab | 1 | circRNA_finder | SRR11550044 |
| SARS-CoV-2_circ_Homo_sapiens_1980 | MN908947.3 | 18450 | 18744 | + | ORF1ab | 1 | circRNA_finder | SRR11550045 |
| SARS-CoV-2_circ_Homo_sapiens_1985 | MN908947.3 | 18494 | 20581 | + | ORF1ab | 1 | circRNA_finder | SRR11550045 |
| SARS-CoV-2_circ_Homo_sapiens_1986 | MN908947.3 | 18494 | 21241 | + | ORF1ab | 1 | circRNA_finder | SRR11550044 |
| SARS-CoV-2_circ_Homo_sapiens_1987 | MN908947.3 | 18494 | 28602 | + | ORF6;M;E;ORF3a;S;N;ORF8;ORF7a;ORF7b;ORF1ab | 1 | circRNA_finder | SRR11550045 |
| SARS-CoV-2_circ_Homo_sapiens_1990 | MN908947.3 | 18524 | 20341 | + | ORF1ab | 1 | circRNA_finder | SRR11550043 |
| SARS-CoV-2_circ_Homo_sapiens_1992 | MN908947.3 | 18560 | 19042 | + | ORF1ab | 1 | circRNA_finder | SRR11550044 |
| SARS-CoV-2_circ_Homo_sapiens_1994 | MN908947.3 | 18647 | 19708 | + | ORF1ab | 1 | circRNA_finder | SRR11550043 |
| SARS-CoV-2_circ_Homo_sapiens_1997 | MN908947.3 | 18657 | 18916 | + | ORF1ab | 1 | circRNA_finder | SRR11550045 |
| SARS-CoV-2_circ_Homo_sapiens_1997 | MN908947.3 | 18657 | 18916 | + | ORF1ab | 1 | circRNA_finder | SRR11550043 |
| SARS-CoV-2_circ_Homo_sapiens_1998 | MN908947.3 | 18657 | 18928 | + | ORF1ab | 1 | circRNA_finder | SRR11550046 |
| SARS-CoV-2_circ_Homo_sapiens_1999 | MN908947.3 | 18657 | 19042 | + | ORF1ab | 1 | circRNA_finder | SRR11550046 |
[truncated: 135,575 more chars]
