# Supplementary material for: Competing endogenous RNA network mediated by circ_3205 in SARS-CoV-2 infected cells
Source: Cell Mol Life Sci. 2022 Jan 17;79(2):75. doi: 10.1007/s00018-021-04119-8 (PMC8763136; doi:10.1007/s00018-021-04119-8)
Supplement: Supplementary file 3 — Supplementary file3 (DOCX 18 KB) [file 18_2021_4119_MOESM3_ESM.docx]

| **#** | **Hsa-mir-298 target** | **Gene Ensembl id** | **Score** |
| --- | --- | --- | --- |
| 1 | MYB | ENSG00000118513 | 0.823 |
| 2 | NRAS | ENSG00000213281 | 0.83 |
| 3 | SERPINB2 | ENSG00000197632 | 0.803 |
| 4 | MERTK | ENSG00000153208 | 0.829 |
| 5 | PRKCE | ENSG00000171132 | 0.861 |
| 6 | APP | ENSG00000142192 | 0.871 |
| 7 | ATP2B1 | ENSG00000070961 | 0.825 |
| 8 | PDE1A | ENSG00000115252 | 0.806 |
| 9 | RAB5A | ENSG00000144566 | 0.918 |
| 10 | CPB2 | ENSG00000080618 | 0.828 |
| 11 | SLC8A1 | ENSG00000183023 | 0.863 |
| 12 | KCNMB4 | ENSG00000135643 | 0.864 |
| 13 | PIK3CA | ENSG00000121879 | 0.884 |
| 14 | AKAP10 | ENSG00000108599 | 0.858 |
| 15 | IRF2 | ENSG00000168310 | 0.907 |
| 16 | FN1 | ENSG00000115414 | 0.942 |
| 17 | RAP1B | ENSG00000127314 | 0.91 |
| 18 | CBX5 | ENSG00000094916 | 0.948 |
| 19 | PIK3C3 | ENSG00000078142 | 0.866 |
| 20 | CIITA | ENSG00000179583 | 0.947 |
| 21 | ADCY5 | ENSG00000173175 | 0.834 |
| 22 | NRAS | ENSG00000213281 | 0.83 |
| 23 | MERTK | ENSG00000153208 | 0.829 |
| 24 | TCF12 | ENSG00000140262 | 0.904 |
| 25 | PPP3R1 | ENSG00000221823 | 0.97 |
| 26 | HLA-DRA | ENSG00000204287 | 0.855 |
| 27 | MYO1C | ENSG00000197879 | 0.864 |
| 28 | SPTBN2 | ENSG00000173898 | 0.885 |
| 29 | PRKCE | ENSG00000171132 | 0.861 |
| 30 | APP | ENSG00000142192 | 0.871 |
| 31 | CREB1 | ENSG00000118260 | 0.812 |
| 32 | PDE1A | ENSG00000115252 | 0.806 |
| 33 | APOBEC3F | ENSG00000128394 | 0.904 |
| 34 | ILF3 | ENSG00000129351 | 0.889 |
| 35 | DCTN3 | ENSG00000137100 | 0.848 |
| 36 | APOBEC3C | ENSG00000244509 | 0.883 |
| 37 | DCTN1 | ENSG00000204843 | 0.926 |
| 38 | CYBB | ENSG00000165168 | 0.923 |
| 39 | FNIP1 | ENSG00000217128 | 0.814 |
| 40 | PIK3CA | ENSG00000121879 | 0.884 |
| 41 | SKAP2 | ENSG00000005020 | 0.844 |
| 42 | FN1 | ENSG00000115414 | 0.942 |
| 43 | OSBPL1A | ENSG00000141447 | 0.921 |
| 44 | VSIG4 | ENSG00000155659 | 0.825 |
| 45 | AP1G1 | ENSG00000166747 | 0.805 |
| 46 | ABCB5 | ENSG00000004846 | 0.863 |
| 47 | TRIM32 | ENSG00000119401 | 0.894 |
| 48 | ENAH | ENSG00000154380 | 0.967 |

**Supplemental table 3.** Hsa-miR-298 targets predicted by microT-CDS. Targets involved in blood coagulation and immune response are highlighted in light blue and yellow, respectively.
