# Supplementary material for: Competing endogenous RNA network mediated by circ_3205 in SARS-CoV-2 infected cells
Source: Cell Mol Life Sci. 2022 Jan 17;79(2):75. doi: 10.1007/s00018-021-04119-8 (PMC8763136; doi:10.1007/s00018-021-04119-8)
Supplement: Supplementary file 4 — Supplementary file4 (DOCX 14 KB) [file 18_2021_4119_MOESM4_ESM.docx]

| **Hsa-miR-298 target gene** | **COVID-19 *vs* CTRL** | **COVID-19 *vs* CTRL (Log2 Fold Change)** | **PMID** |
| --- | --- | --- | --- |
| CPB2 | Upregulated | 6.508533382 | 33425248 |
| DCTN1 | Upregulated | 1.247449949 | 33425248 |
| FN1 | Upregulated | 1.748603748 | 33425248 |
| KCNMB4 | Upregulated | 4.513820527 | 33425248 |
| MERTK | Upregulated | 3.245951009 | 33425248 |
| MYO1C | Upregulated | 1.356084129 | 33425248 |
| PDE1A | Upregulated | 3.130815146 | 33425248 |
| PIK3C3 | Upregulated | 2.131980345 | 33425248 |
| PRKCE | Upregulated | 1.057945026 | 33425248 |
| SERPINB2 | Upregulated | 8.008380407 | 33740387 |

**Supplemental table 4**. List of predicted hsa-miR-298 targets known to be upregulated in biological specimens of COVID-19 patients as compared to unaffected controls (CTRL). Targets are arranged in alphabetical order. Literature references are indicated as PubMed IDs (PMID).
