# Supplementary figures and images for: Competing endogenous RNA network mediated by circ_3205 in SARS-CoV-2 infected cells
Source: Cell Mol Life Sci. 2022 Jan 17;79(2):75. doi: 10.1007/s00018-021-04119-8 (PMC8763136; doi:10.1007/s00018-021-04119-8)

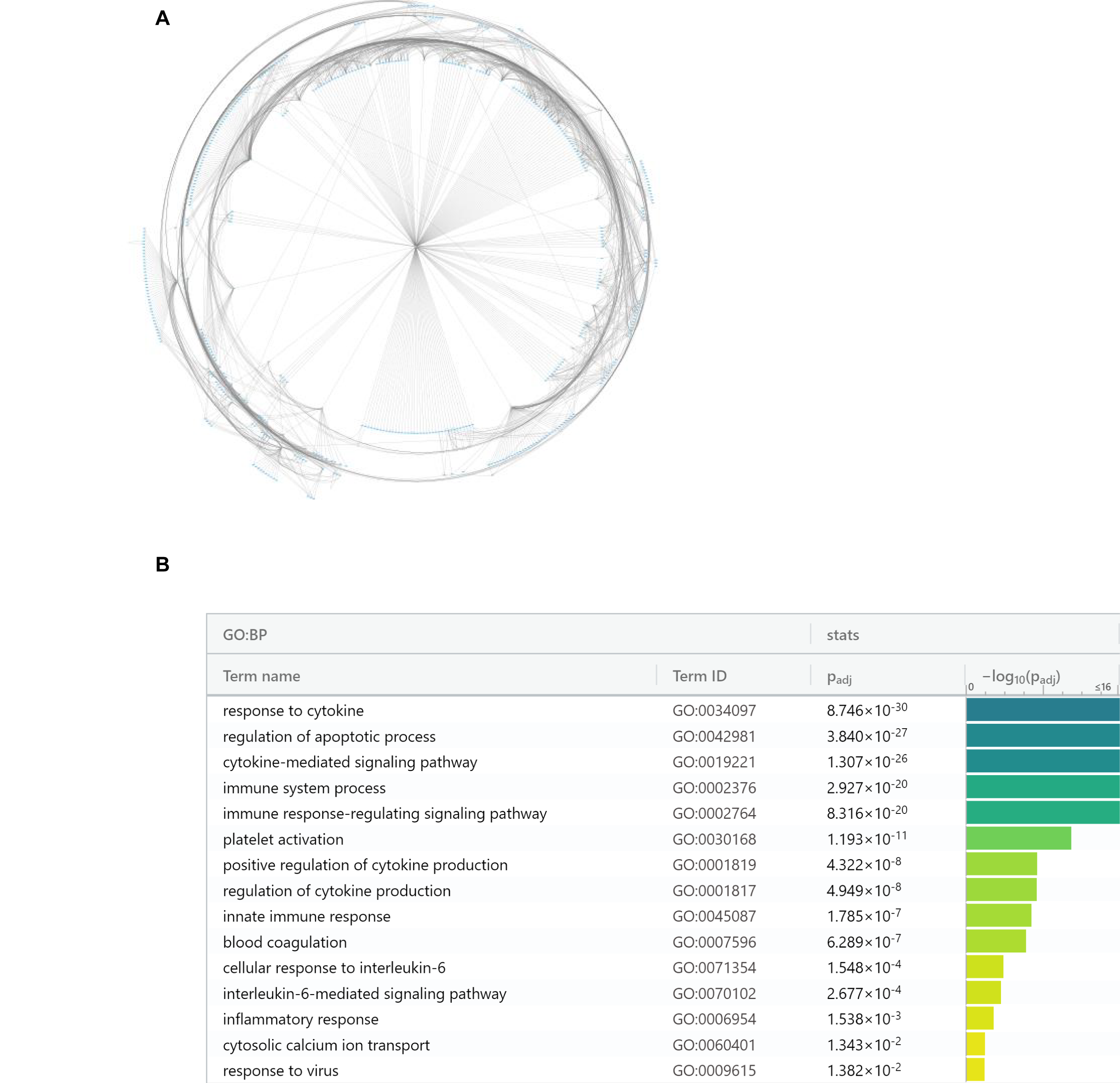

Supplement: Supplementary file 5 — Supplementary file5 (TIF 2275 KB) [file 18_2021_4119_MOESM5_ESM.tif]
